# Gene Expression Changes Associated with the Airway Wall Response to Injury

**DOI:** 10.1371/journal.pone.0058930

**Published:** 2013-04-09

**Authors:** Badrul Yahaya, Gerry McLachlan, Caroline McCorquodale, David Collie

**Affiliations:** 1 Cluster for Regenerative Medicine, Advanced Medical and Dental Institute (AMDI), Universiti Sains Malaysia, Bandar Putra Bertam, Kepala Batas, Penang, Malaysia; 2 The Roslin Institute & Royal (Dick) School of Veterinary Studies, Easter Bush Veterinary Centre, University of Edinburgh, Roslin, Midlothian, Edinburgh, Scotland, United Kingdom; Vanderbilt University Medical Center, United States of America

## Abstract

**Background:**

Understanding the way in which the airway heals in response to injury is fundamental to dissecting the mechanisms underlying airway disease pathology. As only limited data is available in relation to the in vivo characterisation of the molecular features of repair in the airway we sought to characterise the dynamic changes in gene expression that are associated with the early response to physical injury in the airway wall.

**Methodology/Principal Findings:**

We profiled gene expression changes in the airway wall using a large animal model of physical injury comprising bronchial brush biopsy in anaesthetised sheep. The experimental design featured sequential studies in the same animals over the course of a week and yielded data relating to the response at 6 hours, and 1, 3 and 7 days after injury. Notable features of the transcriptional response included the early and sustained preponderance of down-regulated genes associated with angiogenesis and immune cell activation, selection and differentiation. Later features of the response included the up-regulation of cell cycle genes at d1 and d3, and the latter pronounced up-regulation of extracellular matrix-related genes at d3 and d7.

**Conclusions/Significance:**

It is possible to follow the airway wall response to physical injury in the same animal over the course of time. Transcriptional changes featured coordinate expression of functionally related genes in a reproducible manner both within and between animals. This characterisation will provide a foundation against which to assess the perturbations that accompany airway disease pathologies of comparative relevance.

## Introduction

Understanding the mechanisms involved in the airway response to injury is fundamental to the process of piecing together the pathways and mechanisms that underlie more complex respiratory diseases such as asthma and chronic obstructive pulmonary disease (COPD). Indeed the process of wound healing in response to physical injury of the airways has been examined using a variety of experimental animal models ranging from hamsters [1], rats [2]–[4], rabbits [1] and guinea pigs 5–8, to sheep [9] and calves [10], with characterisation at gross, light and electron microscopic resolution.

Such systems have demonstrated that the process of repair involves a sequence of events that appears both efficient and well conserved between species. With successful repair likely involving a series of interlinked and interdependent events, pathology may occur where aspects of sequence and/or interdependence are compromised and knowledge of where such abnormalities arise may offer the potential of modulating an abnormal repair process. Although the complexity of airway epithelial cell phenotypes and their involvement during injury and repair is well discussed, there is a lack of evidence and detailed explanation of how the repair process is regulated at the molecular level.

Expression profiling studies offers the facility of identifying novel genetic pathways involved in development and disease, and their use has extended to include studies of lung development, lung disease pathogenesis, and lung repair following either chemical or physical injury. Whilst these and other studies have offered new insights in relation to specific diseases or under specific experimental conditions, there is clearly a need to apply this technology in the context of understanding the fundamental mechanisms that underlay normal airway injury responses in vivo. This need was recognised and partly addressed by Heguy *et al* (2007) [11], who used bronchial brush biopsy in human subjects to study the airway epithelial transcriptional response at day 7 and day 14 after injury, and found that at day 7 compared with resting epithelium, there were substantial differences in gene expression pattern, with a distinctive airway epithelial “repair transcriptome” of actively proliferating cells in the process of re-differentiation.

The sheep is a popular large animal model for studying lung disease pathogenesis, with comparative relevance often assumed on the basis of similarities in disease phenotype, whether in the context of pathological or functional change. Such studies, in common with many animal models, falter on the hurdle that there are inevitable differences between the experimental and actual disease pathogenesis and it is difficult to dissect what is, or is not, relevant in the process observed. In contrast, studies wherein the challenge or treatment is essentially the same in man as in experimental animal are of value in establishing the comparative relevance of the latter. Hence the approach used by Heguy *et al* (2007) [11], in using a bronchoscopic technique that is easily applied in the context of a large animal model, provides a useful opportunity to compare and contrast responses of a model system to the system being modelled.

We therefore sought to expand on our recently developed sheep model of airway wall injury [9] through characterising the transcriptional response to bronchial brush biopsy during the first seven days after physical injury.

The key feature of the response that was shared with that seen in the human airway was the up-regulation of genes involved in cell cycle – a feature that correlated with previously defined histopathological changes [9]. However, the nature of the experimental design allowed us to expand on these observations and highlight additional potentially important features of the response. These included a pronounced rapid and sustained decrease in the expression of genes involved in angiogenesis, and in immune cell activation, selection and differentiation and a later rise in the expression of genes involved in extracellular matrix remodelling, a rise that was still evident seven days after injury.

## Results

### Changes in airway gene expression induced by bronchial brushing

Some 7786 probes were significantly differentially regulated (up or down, p<0.05) at one or more time points following the induction of physical injury. The number of probes significantly differentially regulated at each time point and their direction of change is listed in the supplementary data section (Table S1: Table 1a). The complete list of genes that were significantly differentially regulated and showed more than a two-fold change in expression relative to baseline (naïve) levels is available in the Supporting Information section (Table S1: Table 1b).

**Table 1:** The DAVID Functional Annotation Clustering tool was used to group genes demonstrating co-ordinate expression on the basis of sharing similar biological meaning.

Biolayout cluster	DAVID functional annotation cluster	Enrichment score	Term	Count	P Value	ACVRL1	AGT	ANGPT1	ANGPT2	APOE	CAV1	CDH2	CDH5	CITED2	CXCL12	DLL4	EMCN	
001	1	6.3	GO:0001568∼blood vessel development	26	3.66E-09	x	x	x	x	x	x	x	x	x	x	x	x	
			GO:0001944∼vasculature development	26	6.04E-09	x	x	x	x	x	x	x	x	x	x	x	x	
			GO:0048514∼blood vessel morphogenesis	20	1.95E-06	x	x	x	x	x	x	x		x	x	x	x	
			GO:0001525∼angiogenesis	12	0.001	x		x	x						x	x	x	
						AGT	BCL11A	CD1D	CD3D	CD3E	CD3G	CD40LG	CD74	CD8A	CD93	CXCL12	EPAS1	
	2	4.0	GO:0042110∼T cell activation	14	1.96E-05		x	x	x	x	x		x	x		x		
			GO:0030097∼hemopoiesis	19	3.55E-05		x	x	x	x		x	x	x			x	
			GO:0001775∼cell activation	21	4.81E-05	x	x	x	x	x	x	x	x	x	x	x		
			GO:0045321∼leukocyte activation	19	4.92E-05		x	x	x	x	x	x	x	x	x	x		
			GO:0046649∼lymphocyte activation	17	5.09E-05		x	x	x	x	x	x	x	x		x		
			GO:0045058∼T cell selection	6	9.07E-05			x	x	x			x					
			GO:0048534∼hemopoietic or lymphoid organ development	19	1.24E-04		x	x	x	x		x	x	x			x	
			GO:0030217∼T cell differentiation	9	2.35E-04		x	x	x	x			x	x				
			GO:0002520∼immune system development	19	2.61E-04		x	x	x	x		x	x	x			x	
			GO:0030098∼lymphocyte differentiation	11	2.94E-04		x	x	x	x		x	x	x				
			GO:0002521∼leukocyte differentiation	12	5.25E-04		x	x	x	x		x	x	x				

EPAS1	GJA5	IL18	KDR	LAMA4	LMO2	MMP19	NOS2	NTRK2	PLXND1	PSEN1	QKI	SH2D2A	TBX3			List Total	Pop Hits	Fold Enrichment	Benjamini	FDR
x	x	x	x	x	x	x	x	x	x	x	x	x	x			346	245	4.1	8.83E-06	6.43E-06
x	x	x	x	x	x	x	x	x	x	x	x	x	x			346	251	4.1	7.28E-06	1.06E-05
x		x	x			x	x	x	x		x	x				346	211	3.7	0.002	0.003
x		x	x			x			x			x				346	148	3.2	0.087	2.503
FGB	HCLS1	HDAC5	HLA-DMA	ID2	IL15	IRF8	ITGAL	KDR	LCK	LCP1	LMO2	PSEN1	RB1	SLAMF7	TLR6					
			x		x		x		x	x		x				346	126	4.3	0.012	0.034
	x	x	x	x	x	x		x	x		x	x	x			346	236	3.1	0.012	0.062
x		x	x		x		x		x	x		x		x	x	346	287	2.9	0.014	0.084
		x	x		x		x		x	x		x		x	x	346	242	3.1	0.012	0.086
		x	x		x		x		x	x		x		x		346	199	3.3	0.010	0.089
			x		x											346	19	12.3	0.014	0.159
	x	x	x	x	x	x		x	x		x	x	x			346	260	2.9	0.016	0.218
			x		x				x							346	65	5.4	0.024	0.411
	x	x	x	x	x	x		x	x		x	x	x			346	276	2.7	0.026	0.457
		x	x		x				x							346	103	4.2	0.027	0.516
		x	x		x				x			x				346	131	3.6	0.043	0.918

The discovered gene groups were ranked according to the EASE enrichment score, which is the minus log transformation of the geometric mean of P-values from the enriched annotation terms associated with the different gene group members. Only groups with EASE scores larger than or equal to 4.0 were considered (geometric mean of Benjamini corrected P-values ≤0.05). For each group the relevant GO-terms for Biological Process are given, together with a list of the genes sharing that term, the number of genes in the query list mapped to any gene set in this ontology (List Total), the number of genes annotated to this gene set on the background list (Pop Hits), the mean Fold enrichment of the genes in the group, the Benjamini-corrected P value (the Fisher Exact Probability Value (representing the “degree of enrichment” of the annotation term within the group) further corrected for false discovery rate using the Benjamini correction) and the false discovery rate (FDR). In this table the two clusters within BioLayout cluster 001 were significantly enriched for terms relating to the formation of blood vessels, and immune cell selection, differentiation and activation.

Functional annotation clustering indicated that the lists of annotated genes significantly regulated and showing a greater than two-fold change in expression at each time point were enriched for biologic process GO terms. An enrichment score of 4.0 or above, and a significance level <0.05 best discriminated the annotation data for consideration and tabulation.

The results of functional annotation clustering applied to the significantly down-regulated annotated genes showing a greater than two-fold change in expression at 6 h (n = 561) indicated that the most significantly enriched clusters were linked to terms relating to the development and formation of blood vessels (Table S2: Table 2a). The most significantly enriched cluster of similarly up-regulated genes (n = 154) at this time point contained terms relating to wounding, inflammation and defence responses and the second cluster was significantly enriched for genes involved in responses to bacteria, organic substances and lipopolysaccharide (Table S2: Table 2b).

**Table 2:** The results of DAVID functional annotation clustering applied to the genes sharing a coordinate pattern of expression as identified using BioLayout Express 3D.

Biolayout cluster	DAVID functional annotation cluster	Enrichment score	Term	ADAMTS2	CCDC80	COL12A1	COL18A1	COL1A1	COL1A2	COL3A1	COL5A1	COL5A2	CRABP2	CST6	ELN	KRT14	KRT17
002	1	12.7	GO:0030198∼extracellular matrix organization	x	x	x	x	x	x	x	x	x			x		
			GO:0043062∼extracellular structure organization	x	x	x	x	x	x	x	x	x			x		
			GO:0030199∼collagen fibril organization	x		x		x	x	x	x	x					
	2	6.9	GO:0030199∼collagen fibril organization	x		x		x	x	x	x	x					
			GO:0032963∼collagen metabolic process	x				x		x	x						
			GO:0044259∼multicellular organismal macromolecule metabolic process	x				x		x	x						
			GO:0044236∼multicellular organismal metabolic process	x				x		x	x						
			GO:0007398∼ectoderm development	x				x	x	x	x	x	x	x		x	x
			GO:0008544∼epidermis development	x				x	x	x	x	x	x	x		x	x
			GO:0043588∼skin development	x				x	x	x	x	x					
			GO:0032964∼collagen biosynthetic process					x		x	x						
			GO:0030574∼collagen catabolic process	x													
			GO:0044243∼multicellular organismal catabolic process	x													
				ADAMTS2	AEBP1	ANKH	BMP1	COL12A1	COL1A1	COL1A2	COL3A1	COL5A2	FBN1	IGFBP3	INHBA	MMP13	MMP2
002	3	4.2	GO:0001501∼skeletal system development		x	x	x	x	x	x	x	x	x	x	x	x	x
			GO:0001503∼ossification				x		x			x		x		x	x
			GO:0060348∼bone development				x		x			x		x		x	x
			GO:0048705∼skeletal system morphogenesis				x		x								x
			GO:0060349∼bone morphogenesis						x								

KRT5	KRT6A	LOX	LUM	MMP13	MMP2	MMP7	MMP9	MREG	PDGFRA	POSTN	SERPINH1	TGFBI	TNC	List Total	Pop Hits	Fold TEnrichment	Benjamini	FDR
		x	x				x		x	x	x	x		134	104	16.5	5.25E-12	6.37E-12
		x	x				x		x	x	x	x	x	134	163	11.1	2.56E-10	6.21E-10
		x	x								x			134	29	34.8	1.50E-09	9.08E-09
		x	x								x			134	29	34.8	1.50E-09	9.08E-09
				x	x	x	x				x			134	28	32.4	4.19E-08	3.05E-07
				x	x	x	x				x			134	31	29.3	7.78E-08	7.56E-07
				x	x	x	x				x			134	37	24.6	3.22E-07	3.52E-06
x	x							x						134	199	6.6	6.72E-05	9.79E-04
x								x						134	184	6.6	2.03E-04	0.003
														134	29	20.9	6.65E-04	0.014
											x			134	5	80.8	6.88E-04	0.015
				x	x	x	x							134	20	25.2	0.002	0.063
				x	x	x	x							134	26	19.4	0.006	0.185
MMP7	MMP9	PDGFRA	POSTN	RBP4	TGFB1	TGFB3	WNT5A											
	x	x	x	x	x	x	x							134	319	6.3	5.73E-08	4.87E-07
					x	x								134	115	7.0	0.007	0.225
					x	x								134	123	6.6	0.011	0.342
		x			x	x								134	112	5.4	0.119	7.800
					x	x								134	20	15.1	0.271	23.550

In this instance the functional annotation is applied to the genes of cluster 002, and the results indicate that the gene lists are enriched for terms relating to the biological processes of extracellular matrix organisation, collagen fibril organisation, collagen metabolism, and skeletal system development. See the legend for table 2 for a description of the column headings.

Functional annotation clustering applied to the genes down-regulated 1d after bronchial brushing (n = 160) generated clusters whose terms failed to reach significance after Benjamini-Hochberg correction (data not shown). Of the up-regulated genes (n = 324) at this time point, the most significantly enriched cluster was populated with highly significant terms relating to cell division and its associated processes (Table S3: Tables 3a and 3b).

**Table 3:** The results of DAVID functional annotation clustering applied to the genes sharing a coordinate pattern of expression as identified using BioLayout Express 3D.

Biolayout cluster	DAVID functional annotation cluster	Enrichment score	Term	ASF1B	ASPM	AURKA	AURKB	BIRC5	BUB1	CBX5	CCNA2	CCNB1	CCNB2	CCNB3	CDC20	CDC45	CDC6	CDCA3	CDCA5	CDKN2D	CDT1	CENPH	CEP72
003	1	31.3	GO:0000279∼M phase		x	x	x	x	x		x	x	x	x	x		x	x	x				
			GO:0022403∼cell cycle phase		x	x	x	x	x		x	x	x	x	x		x	x	x	x			
			GO:0007049∼cell cycle		x	x	x	x	x		x	x	x	x	x	x	x	x	x	x	x		x
			GO:0022402∼cell cycle process		x	x	x	x	x		x	x	x	x	x		x	x	x	x			x
			GO:0000280∼nuclear division		x	x	x	x	x		x	x	x		x		x	x	x				
			GO:0007067∼mitosis		x	x	x	x	x		x	x	x		x		x	x	x				
			GO:0000087∼M phase of mitotic cell cycle		x	x	x	x	x		x	x	x		x		x	x	x				
			GO:0048285∼organelle fission		x	x	x	x	x		x	x	x		x		x	x	x				
			GO:0000278∼mitotic cell cycle		x	x	x	x	x		x	x	x		x		x	x	x	x			
			GO:0051301∼cell division		x		x	x	x		x	x	x	x	x		x	x	x			x	
			GO:0007059∼chromosome segregation					x											x			x	
	2	12.1	GO:0007051∼spindle organization			x																	
			GO:0000226∼microtubule cytoskeleton organization			x																	x
			GO:0007017∼microtubule-based process			x																	x
			GO:0007010∼cytoskeleton organization			x																	x
	3	8.1	GO:0006259∼DNA metabolic process													x	x			x	x		
			GO:0006974∼response to DNA damage stimulus								x									x			
			GO:0006281∼DNA repair																	x			
			GO:0033554∼cellular response to stress								x									x			
	4	7.3	GO:0007059∼chromosome segregation					x											x			x	
			GO:0000070∼mitotic sister chromatid segregation																x				
			GO:0000819∼sister chromatid segregation																x				
			GO:0051276∼chromosome organization	x						x									x			x	
			GO:0007076∼mitotic chromosome condensation																x				
			GO:0030261∼chromosome condensation																x				
			GO:0006323∼DNA packaging	x															x				
	5	5.7	GO:0007126∼meiosis											x									
			GO:0051327∼M phase of meiotic cell cycle											x									
			GO:0051321∼meiotic cell cycle											x									
			GO:0007127∼meiosis I																				
			GO:0006310∼DNA recombination																				

CGREF1	CKAP2	CKS1B	CKS2	DIAPH3	DLGAP5	DTYMK	ESPL1	EZH2	FANCD2	FBXO5	FEN1	GINS4	GTF2H5	IGF1	IGFBP4	KIF22	KIF2C	KNTC1	List Total	Pop Hits	Fold Enrichment	Benjamini	FDR
			x		x		x		x	x						x	x	x	116	329	16.0	1.62E-38	2.30E-38
			x		x		x		x	x						x	x	x	116	414	13.0	1.07E-35	3.04E-35
x	x	x	x		x	x	x		x	x						x	x	x	116	776	8.4	3.18E-35	1.36E-34
x			x		x		x		x	x						x	x	x	116	565	10.1	1.16E-33	6.57E-33
					x		x			x						x	x	x	116	220	17.5	1.10E-28	7.80E-28
					x		x			x						x	x	x	116	220	17.5	1.10E-28	7.80E-28
					x		x			x						x	x	x	116	224	17.2	1.66E-28	1.42E-27
					x		x			x						x	x	x	116	229	16.8	2.95E-28	2.94E-27
					x		x			x						x	x	x	116	370	12.0	8.99E-28	1.02E-26
		x	x				x			x								x	116	295	13.4	4.28E-26	5.47E-25
					x		x												116	81	23.0	2.09E-14	3.55E-13
			x				x			x									116	45	36.3	3.89E-15	5.53E-14
			x				x			x							x		116	147	13.5	7.42E-12	1.58E-10
			x				x			x						x	x		116	253	8.8	2.85E-10	6.47E-09
			x	x			x			x							x		116	436	5.1	1.30E-06	4.26E-05
									x		x	x	x	x	x	x			116	506	6.9	1.14E-14	1.78E-13
									x		x		x			x			116	373	5.0	2.59E-05	9.58E-04
									x		x		x			x			116	284	5.7	3.28E-05	0.001
									x		x		x			x			116	566	3.3	0.003	0.140
					x		x												116	81	23.0	2.09E-14	3.55E-13
					x		x												116	36	32.4	8.70E-10	2.10E-08
					x		x												116	37	31.5	1.08E-09	2.76E-08
					x		x	x	x										116	485	4.1	1.24E-04	0.005
																			116	13	35.9	0.005	0.258
																			116	25	18.7	0.028	1.909
																			116	117	5.0	0.255	24.817
			x				x		x	x									116	98	13.1	5.33E-07	1.59E-05
			x				x		x	x									116	98	13.1	5.33E-07	1.59E-05
			x				x		x	x									116	100	12.8	6.20E-07	1.94E-05
			x				x		x										116	42	13.9	0.012	0.691
																			116	105	4.4	0.549	63.042

Biolayout cluster	DAVID functional annotation cluster	Enrichment score	Term	KPNA2	MCM10	MCM3	MCM4	MCM5	MND1	NCAPG	NCAPH	NDC80	NEK2	NUF2	ORC1L	PCNA	PRC1	PTTG1	RACGAP1P	RAD51	RAD54L	RFC2	RNASEH2A
003	1	31.3	GO:0000279∼M phase	x					x	x	x	x	x	x			x	x		x	x		
			GO:0022403∼cell cycle phase	x					x	x	x	x	x	x			x	x		x	x		
			GO:0007049∼cell cycle	x		x			x	x	x	x	x	x			x	x	x	x	x		
			GO:0022402∼cell cycle process	x					x	x	x	x	x	x			x	x	x	x	x		
			GO:0000280∼nuclear division							x	x	x	x	x				x					
			GO:0007067∼mitosis							x	x	x	x	x				x					
			GO:0000087∼M phase of mitotic cell cycle							x	x	x	x	x				x					
			GO:0048285∼organelle fission							x	x	x	x	x				x					
			GO:0000278∼mitotic cell cycle	x						x	x	x	x	x			x	x					
			GO:0051301∼cell division					x		x	x	x	x	x			x	x	x				
			GO:0007059∼chromosome segregation							x	x	x	x	x				x					
	2	12.1	GO:0007051∼spindle organization									x					x						
			GO:0000226∼microtubule cytoskeleton organization									x	x				x						
			GO:0007017∼microtubule-based process	x								x	x				x						
			GO:0007010∼cytoskeleton organization									x	x				x		x				
	3	8.1	GO:0006259∼DNA metabolic process	x	x	x	x	x	x						x	x		x		x	x	x	x
			GO:0006974∼response to DNA damage stimulus													x		x		x	x	x	
			GO:0006281∼DNA repair													x		x		x	x	x	
			GO:0033554∼cellular response to stress													x		x		x	x	x	
	4	7.3	GO:0007059∼chromosome segregation							x	x	x	x	x				x					
			GO:0000070∼mitotic sister chromatid segregation							x	x	x	x										
			GO:0000819∼sister chromatid segregation							x	x	x	x										
			GO:0051276∼chromosome organization							x	x	x	x					x			x		
			GO:0007076∼mitotic chromosome condensation							x	x												
			GO:0030261∼chromosome condensation							x	x												
			GO:0006323∼DNA packaging							x	x												
	5	5.7	GO:0007126∼meiosis						x				x							x	x		
			GO:0051327∼M phase of meiotic cell cycle						x				x							x	x		
			GO:0051321∼meiotic cell cycle						x				x							x	x		
			GO:0007127∼meiosis I																	x			
			GO:0006310∼DNA recombination						x											x	x		

RRM2	SFN	SKA1	SMC1A	SMC2	SPC25	STMN1	TACC3	TK1	TPX2	TRIP13	TTK	TUBB	TYMS	UBE2C	UHRF1	ZWILCH	ZWINT	List Total	Pop Hits	Fold Enrichment	Benjamini	FDR
		x	x	x	x	x	x		x	x	x	x		x		x	x	116	329	16.0	1.62E-38	2.30E-38
		x	x	x	x	x	x		x	x	x	x		x		x	x	116	414	13.0	1.07E-35	3.04E-35
		x	x	x	x	x	x		x	x	x	x		x	x	x	x	116	776	8.4	3.18E-35	1.36E-34
		x	x	x	x	x	x		x	x	x	x		x		x	x	116	565	10.1	1.16E-33	6.57E-33
		x	x	x	x				x			x		x		x	x	116	220	17.5	1.10E-28	7.80E-28
		x	x	x	x				x			x		x		x	x	116	220	17.5	1.10E-28	7.80E-28
		x	x	x	x				x			x		x		x	x	116	224	17.2	1.66E-28	1.42E-27
		x	x	x	x				x			x		x		x	x	116	229	16.8	2.95E-28	2.94E-27
		x	x	x	x	x			x		x	x		x		x	x	116	370	12.0	8.99E-28	1.02E-26
		x	x	x	x									x		x	x	116	295	13.4	4.28E-26	5.47E-25
		x	x	x	x												x	116	81	23.0	2.09E-14	3.55E-13
			x		x	x	x				x	x		x			x	116	45	36.3	3.89E-15	5.53E-14
			x		x	x	x				x	x		x			x	116	147	13.5	7.42E-12	1.58E-10
			x		x	x	x				x	x		x			x	116	253	8.8	2.85E-10	6.47E-09
			x		x	x	x				x	x		x			x	116	436	5.1	1.30E-06	4.26E-05
x			x					x		x			x		x			116	506	6.9	1.14E-14	1.78E-13
	x		x							x			x		x			116	373	5.0	2.59E-05	9.58E-04
			x							x			x		x			116	284	5.7	3.28E-05	0.001
	x		x							x			x		x			116	566	3.3	0.003	0.140
		x	x	x	x												x	116	81	23.0	2.09E-14	3.55E-13
			x	x													x	116	36	32.4	8.70E-10	2.10E-08
			x	x													x	116	37	31.5	1.08E-09	2.76E-08
			x	x													x	116	485	4.1	1.24E-04	0.005
				x														116	13	35.9	0.005	0.258
				x														116	25	18.7	0.028	1.909
				x														116	117	5.0	0.255	24.817
			x							x								116	98	13.1	5.33E-07	1.59E-05
			x							x								116	98	13.1	5.33E-07	1.59E-05
			x							x								116	100	12.8	6.20E-07	1.94E-05
										x								116	42	13.9	0.012	0.691
										x								116	105	4.4	0.549	63.042

In this instance the functional annotation is applied to the genes of cluster 003, and the results indicate that the gene lists are enriched for terms relating to cell division and its associated processes. See the legend for table 2 for a description of the column headings.

Three days after physical injury down-regulated annotated gene (n = 180) clusters contained terms that failed to reach significance after FDR correction (data not shown). Similarly up-regulated gene (n = 366) clusters were significantly enriched with terms relating to cell division and its associated processes (Table S4: Table 4a), and wounding, inflammatory, defense and immune responses (Table S4: Table 4b). Other clusters were significantly enriched for genes involved in ectoderm, epidermis and skin development, and for genes involved in ECM, collagen fibril, and extracellular structure organization, skin development, and response to hormone stimuli (Table S4: Tables 4b–e).

**Table 4:** The results of DAVID functional annotation clustering applied to the genes sharing a coordinate pattern of expression as identified using BioLayout Express 3D.

Biolayout cluster	DAVID functional annotation cluster	Enrichment score	Term	BYSL	FTSJ3	IMP4	METTL1	NOLC1	NPM3	RRP9	RRS1	SYNCRIP	TSR1	WDR77	List Total	Pop Hits	Fold Enrichment	Benjamini	FDR
004	1	3.5	GO:0042254∼ribosome biogenesis	x	x	x		x	x	x	x		x		65	122	13.6	0.001	0.003
			GO:0022613∼ribonucleoprotein complex biogenesis	x	x	x		x	x	x	x		x	x	65	180	10.4	8.62E-04	0.003
			GO:0006364∼rRNA processing		x	x		x	x	x					65	92	11.3	0.185	1.438
			GO:0016072∼rRNA metabolic process		x	x		x	x	x					65	96	10.8	0.174	1.683
			GO:0034470∼ncRNA processing		x	x	x	x	x	x					65	187	6.7	0.241	2.889
			GO:0034660∼ncRNA metabolic process		x	x	x	x	x	x					65	230	5.4	0.307	6.899
			GO:0006396∼RNA processing		x	x	x	x	x	x		x		x	65	547	3.0	0.571	20.178

In this instance the functional annotation is applied to the genes of cluster 004, and the results indicate that the gene lists are enriched for terms relating to the biological processes of ribosome and ribonucleoprotein biogenesis. See the legend for table 2 for a description of the column headings.

Seven days after physical injury 68 genes showed a greater than two-fold significant down-regulation in expression. GO terms relating to muscle contraction and muscle system process reached significance after FDR correction (Table S5: Table 5a). Annotation clustering applied to the similarly up-regulated genes at this time point (n = 197) generated clusters containing significant terms relating to collagen and ECM organisation, skin, ectoderm and epidermis development, to skeletal development, ossification, bone development and osteoblast differentiation, to cell migration and motility, to regeneration and growth, to cell cycle processes and wound healing, blood coagulation, hemostasis and regulation of body fluid levels (Table S4: Table S5b–f).

**Figure 1:**
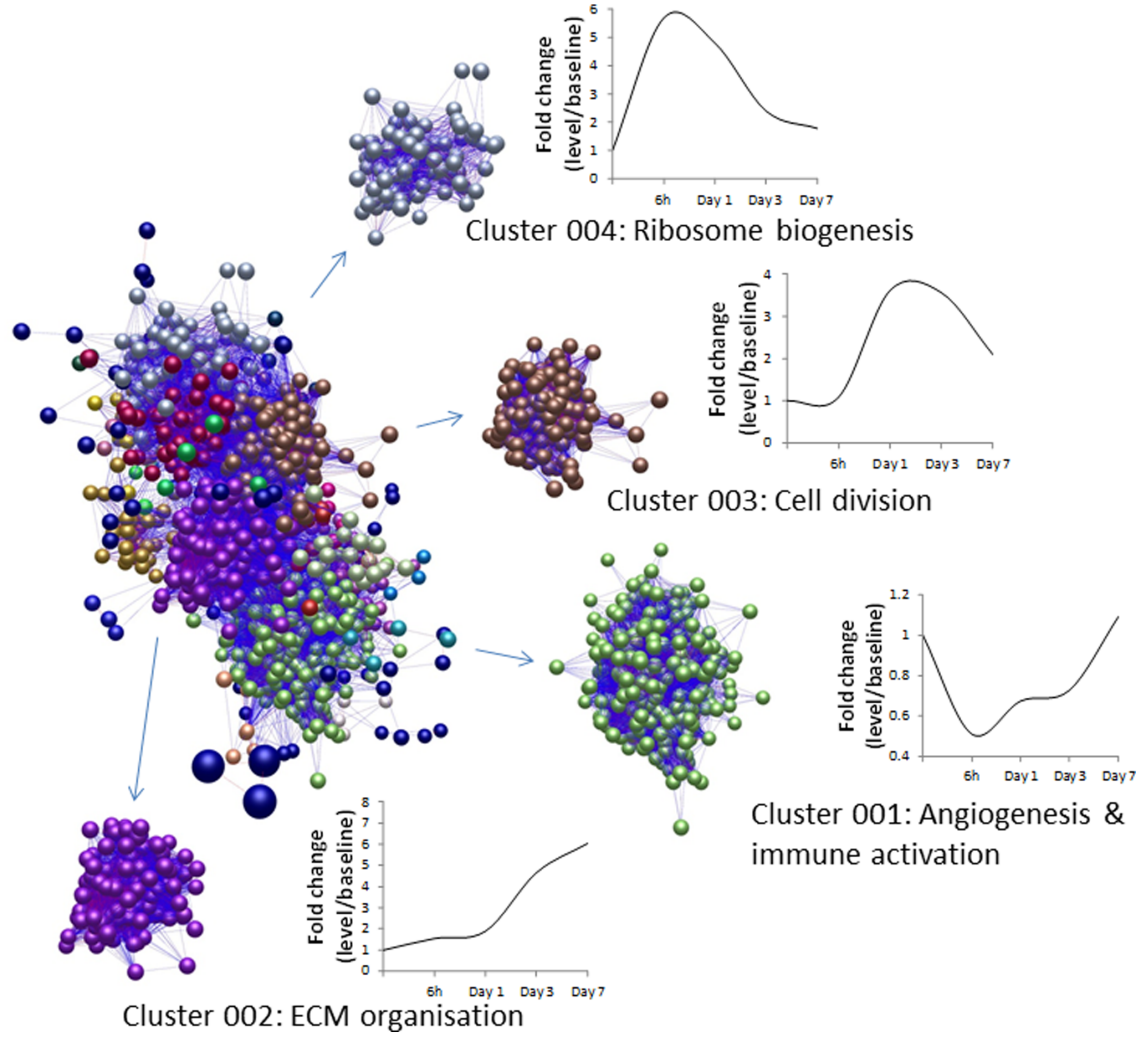
Node and edge graph used to visualise co-ordinately expressed genes from the 1145 unique genes that were significantly differentially regulated (with greater than twofold change in expression) at least once during the course of the injury response. Separate clusters of co-ordinately expressed genes identified on the basis of application of the Markov clustering algorithm are differentially coloured. Functional annotation of the genes present in the four largest clusters shown (Clusters 001–004) indicated that there was a significant enrichment for biological process terms within each cluster. The mean fold-change of gene expression during the time-course of the injury response is also shown for each cluster.

Biolayout Express was used to visualise co-ordinately expressed genes from the 1145 unique genes that were significantly differentially regulated (with greater than twofold change in expression) at least once during the response to injury (Figure 1).

The largest cluster of co-ordinately expressed genes involved 528 nodes relating to 446 unique genes (cluster 001) (Figure 1). Functional annotation of this cluster indicated that sub-clusters within this cluster were significantly enriched for terms relating to the development and formation of blood vessels, and the activation, selection and differentiation of T cells, lymphocytes and leukocytes. The dynamic change in the mean gene expression of this cluster, depicted in figure 1, indicates that there was a decrease in expression apparent at 6h that persists for at least the first three days and returns to close to baseline values by day 7 (Table 1).

The second largest cluster involved 232 nodes relating to 158 unique genes (cluster 002) (figure 1). This gene list was enriched for functional GO terms relating to the biological processes of the extracellular matrix organisation, collagen fibril organisation, collagen metabolism, and skeletal system development, predominantly the same genes that were shown to be significantly elevated at day 3 and day 7 (Table 2).

Similarly the third largest cluster of co-ordinately expressed genes, involving 167 nodes representing 151 unique genes (cluster 003) (figure 1), largely concerned those genes shown to be significantly up-regulated at day 1 and day 3 and whose functions related to cell division and its associated processes (Table 3).

Cluster 004 (figure 1), involving 93 nodes representing 79 unique genes, was significantly enriched in terms relating to ribosome and ribonucleoprotein biogenesis. Genes in this cluster demonstrated a pronounced increase at 6h, followed by a decline to baseline levels by day 7 (Table 4).

Gene ontology terms in the remaining clusters failed to reach significance after Benjamini correction.

## Discussion

In interpreting changes in gene expression in this model it is appropriate to consider whether the nature of the perturbing insult might in itself immediately change the proportions of cells present and by direct extension change the transcriptional profile. This would be particularly relevant to the airway epithelium, as the purpose of bronchial brush biopsy is normally to collect a sample of epithelial cells. A recent study by Dvorak *et al* (2011) [2] used bronchial brushing to obtain airway epithelium from healthy nonsmokers and demonstrated that the number of epithelial cells recovered ranged from 4.2–15×10^6^, with an average of 99%±1% epithelial cells. The proportion of ciliated, secretory, undifferentiated and basal cells in collected material was 57%, 11%, 11% and 21% respectively [2]. Hence, any reduction in epithelial-specific genes in the present study would have to be viewed in this context – for instance of the 23 ciliary related genes present on the array (referenced against the ciliary proteome database [3]), 14 were expressed at a lower level, 2 at a higher level and 7 unchanged relative to expression in un-brushed airway samples. This contrasts with the situation for endothelial cell associated genes (from Favre *et al* (2003) [4]) where the greatest proportion (51%; 25/49) showed unchanged levels of expression relative to control, 37% were reduced and 12% increased relative to control.

In the present study a notable feature of the early response to injury concerned the down-regulation of a number of genes involved in angiogenesis. These genes included angiopoietin-1 (ANGPT1) and -2 (ANGPT2). ANGPT1 binds to the extracellular domain of tie-2 receptors on endothelial cells, promoting vessel integrity and inhibiting vascular leakage. Notably the tie-2 receptor (TEK) is also significantly down-regulated at 6 hours. Whilst the similarly down-regulated ANGPT2 is normally considered an antagonist of ANGPT1, it may also function as a partial TEK agonist under certain conditions [5]. The receptor for vascular endothelial growth factor, the kinase insert domain receptor (KDR), is also significantly down-regulated early – further suggesting that early events negatively impact on vessel formation and maintenance. Additional down-regulated genes included the tie-1 receptor (TIE1), vascular endothelial cadherin (CDH5) which serves the purpose of maintaining newly formed vessels, and endomucin (EMCN). In demonstrating that all of these genes are enriched in the endothelial cell population in the lung, Favre *et al* (2003) commented on the implication that there may either be a statutory need for continuous remodelling of the endothelium in the adult lung, or that these genes have different functions in quiescent cells [6]. It is worth speculating on the balance between physical interactions, in terms of cell to ECM adhesions and strain, and chemical cues, in terms of the soluble angiogenic factors released into the wound, in dictating the down-regulation of angiogenesis. The early process of epithelial denudation and perturbation of mucosal and submucosal structures would likely be accompanied by the release, planned or otherwise, of a veritable cornucopia of soluble mediators and regulators. Conceptually it is hard to square the paradox of this presumed chaotic environment with the uniform epithelial response that emerges. It is perhaps easier to imagine that the overriding influence might not be chemical, but physical, in the sense of the sudden change in the way that cells would sense stress and strain in the abruptly disorganised airway wall structure. Certainly, recent reviews emphasise the importance of such mechanotransduction in both normal physiology and during the development of disease pathology [13], [14]. It is appropriate to examine some of the specific changes in expression that we have identified in light of data indicating that the Rho inhibitor, p190RhoGAP (also known as GRLF1) controls capillary network formation by modulating the balance between two antagonistic transcription factors, GTF2I, which downregulates, and GATA2,which upregulates, VEGFR2 (KDR) expression [15]. In the context of our data where KDR expression was downregulated at 6 h one might anticipate an increased expression of GTF2I. However the opposite was true, GTF2I was significantly downregulated at this time point (we were unable to determine the level of expression of GATA2). In considering this apparent contradiction it is important to acknowledge however, that GTF2I is a ubiquitous and a multifunctional transcription factor with a broad spectrum of biological roles in a variety of cell types, and that the present experiment does not allow us to separate the specific contribution of these various cell types towards the overall level of expression. Whichever the reason it is clear from this study that the immediate response to injury involves a significant suppression of vessel maintenance and/or angiogenesis in the airway wall. In the study of Roy *et al* (2008) [7], when all the genes that were significantly changed at any single time point were subject to filtering for cell type association it was apparent that endothelial-specific genes disappeared promptly (12 h onwards) on skin wounding. Amongst the genes regulated in this fashion were myocyte enhancer factor, aquaporin, tie-2 receptor, glutathione S-transferase and baculoviral IAP repeat-containing 5, all genes that were similarly down-regulated in the airway wall 6 h after physical injury in the present model. Whilst the physiological role for such down-regulation is unknown at this time it is tempting to speculate that constitutive expression of such genes does indeed reflect the need for baseline maintenance in an environment where endothelial cells are constantly subject to pulsatile forces associated with blood flow as well as the dynamics of airway wall movements linked to ventilation.

Whilst functional annotation clustering did not suggest the presence of any other functional groupings in the genes down-regulated at 6h, when the significantly regulated genes were clustered on the basis of coordinate expression over the whole time course the largest cluster identified contained a sub-cluster whose genes were significantly enriched for terms relating to the selection, differentiation and activation of T cells, lymphocytes and leukocytes. The clustered genes included crucial elements of MHC presentation by antigen presenting cells (APCs) – notably the invariant chain Ii (CD74), involved in exporting MHC II from the endoplasmic reticulum, and HLA-DM, the catalytic enzyme responsible for making available the binding groove of MHC II for binding peptides generated within the endosomal system. Other reduced expression genes included T cell receptor complex (TCR) proteins (CD3d, CD3E, CD3G), the T cell co-receptor component CD8A, and the lymphocyte-specific protein tyrosine kinase (LCK) which associates with the cytoplasmic tail of this co-receptor and assists in signal transduction. The expression of these genes decreased promptly after injury and remained at a reduced level until day 3 before returning to baseline levels by day 7 after injury indicating a reduced emphasis on antigen presentation and the selection, differentiation and activation of T cells, lymphocytes and leukocytes. Although Heguy *et al* (2007) also demonstrated differentially regulated immune response genes 7 days after physical injury, there appeared to be no consistent pattern of change – with approximately equal numbers of genes being up-, and down-, regulated [11].

**Figure 2:**
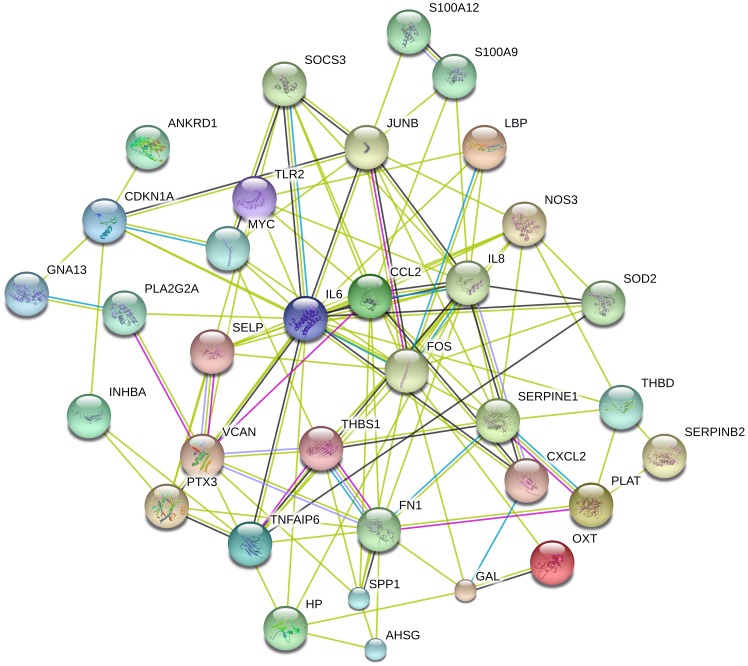
STRING-9.0 analysis (*Homo sapiens* at: (http://string-db.org/); parameters: default setting) of proteins coded by genes significantly up-regulated more than two-fold at 6 h after physical injury to the airway wall that were enriched for terms relating to the biological processes of response to wounding, and inflammatory and defence responses. The figure summarizes the network of predicted associations for this group of proteins. The network nodes are proteins. The edges which represent the predicted functional associations are drawn in coloured lines. These lines represent the existence of the types of evidence used in predicting the associations. A green line indicates the presence of neighbourhood evidence, a blue line – co-ocurrence evidence, a purple line – experimental evidence, a light blue line – database evidence and a black line – co-expression evidence.

Functional annotation clustering indicated that the genes significantly up-regulated more than two-fold at 6 h were enriched for terms relating to the biological processes of response to wounding, and inflammatory and defence responses. The subsequent expression of these genes after the 6h time point was varied and they did not cluster on the basis of coordinate expression. The up-regulated genes that showed the greatest fold-change at 6h were metallothionein 1A (MT1A, 110-fold), interleukin 6, (IL6, 54-fold), lipopolysaccharide binding protein (LBP, 45-fold), keratin 6A (KRT6A, 24-fold) and haptoglobin (HP, 20-fold). The transcription of MT1A is activated by a variety of stress stimuli, including metals, glucocorticoids, proinflammatory cytokines, and reactive oxygen species. MT1A is a scavenger of reactive oxygen species and can be induced in human small airway epithelial cells exposed to Delta9-tetrahydrocannabinol [8], and sulfur mustard [9]. Its lung-protective properties have been demonstrated in metallothionein-transgenic versus metallothionein-knockout mice in response to nickel-induced lung injury [10], bacterial endotoxin [11] and ozone [12]. Although the physiological relevance of this acute increase in expression to the subsequent response to injury remains to be elucidated in our model, the potential exists to manipulate lung levels of this protein through local lung gene therapeutic and/or modulatory strategies. Interleukin 6 is pivotal in generating the acute inflammatory response, and lies, with CCL2, IL8 and FOS, at the centre of the network of biological interactions highlighted using the STRING (Search Tool for the Retrieval of Interacting Genes/Proteins at http://string-db.org/) database [13] to predict and visualise functional interactions amongst these genes (Figure 2). Keratin 6A is also highly expressed early during the response to injury, a finding that concords with observations in relation to acute epidermal injury [14] and suggests the immediate need for cytoskeletal reorganisation, perhaps associated with de-differentiation and migration from the epithelial cells at the wound margins. Published data indicates that KRT6A, which is not normally expressed in the human bronchial epithelium, is also markedly upregulated in patients with squamous cell carcinoma of the lung, and in patients with adenocarcinoma of the lung [15].

Another observation arising out of Biolayout clustering to identify co-ordinately expressed genes across the whole time course was the identification of a cluster of genes (Figure 1, cluster 004) involved in ribosome biogenesis and metabolic processes that also peaked in expression at 6 h and thereafter declined. Cell growth is a prerequisite for cell proliferation, and ribosome biogenesis is a limiting factor for cell growth [16] – hence the increased ribosome biogenesis at 6 h is presumed necessary to furnish the proteins required for the subsequent increase in cell proliferation observed on days 1 and 3 (Figure 1, cluster 003). Indeed, our previous work determined that there was a marked increase in the proliferative activity noticeable by day 1 after injury with the proliferation extending throughout the epithelial structures of the wound margins – involving both epithelial cells and underlying basal and/or parabasal cell and also extending to the area underneath the damaged epithelium in the mucosal and submucosal compartments [9]. By day 3 extensive proliferative activity was apparent in the early granulation tissue underlying the area of mucosal disruption and by day 7, proliferative activity in the damaged area which was spread throughout organising granulation tissue had subsided in extent from that seen at day 3. The observed concordance between such evidence of proliferation and the measured increase in expression of cell cycle genes at the same time points is therefore not unexpected. Notably a number of the cell cycle genes were similarly identified by Heguy *et al* (2007) [11] as being elevated 7 days after bronchial brushing injury in human airway epithelium. These authors found that at day 7 the majority of cell cycle genes expressed in the airway epithelium were synchronised to the G2 and M phases of the cell cycle, and commented on the possibility that the epithelial cells had reached their last division at that point. We also found that the majority of genes identified (68%) in our samples of the whole airway wall belonged to the G2/M and M phases of the cell cycle – however in our instance the peak expression occurred at days 1 and 3, not at day 7 (Figure 1, cluster 003). The concept of cell cycle synchrony during the injury response of the airway wall, with its diverse complement of cell types involved in proliferation, raises questions as to whether circadian rhythms operate to synchronise cell cycle in this context. The lung has a robust peripheral circadian rhythm driven by the expression of core clock proteins in bronchiolar epithelial cells [17]. The central co-ordinating pacemaker is located in the suprachiasmatic nucleus (SCN) of the hypothalamus. Amongst the many genes influenced by circadian rhythms are those involved in cell cycle and even in the context of a wound environment can be found evidence of diurnal variation of mitotic activity [18], [19]. Whilst it is not possible within the framework of the present study to determine whether circadian rhythms influence mitotic activity following physical airway injury there is currently wider interest in the observation that airway diseases such as asthma are characterised by dyssynchrony in epithelial mitotic responses following injury [20]. Clearly there are grounds in this model system for exploiting the concept of using local lung injury protocols to dissect the influence of circadian rhythms in modulating the response to injury against a backdrop of baseline or diseased conditions.

The other major cluster of co-ordinately expressed genes (Figure 1, cluster 002) were those genes concerned with extracellular matrix organisation – with levels of expression being raised relative to baseline at d3 and d7. We previously determined that granulation tissue featured prominently in the airway wall at these times [9]. Some limited evidence for extracellular matrix-associated gene involvement at day 7 was evident in the study of Heguy *et al*, with only a few genes shared between studies (COL1A1, COL1A2, COL3A1, COL4A1, COL4A2 and SPARC). We presume that these differences reflect the respective tissues of origin – namely predominately epithelial vs whole airway wall. Clearly it would be highly informative to extend the present time course beyond 7 days and define the point at which transcriptional changes relating to the extracellular matrix revert to their baseline state. Such information would provide a basis for studying perturbation of normal healing, and understanding the pathophysiology of abnormal injury responses in the context of underlying disease pathology. Central to this cluster are collagen genes, as well as other key ECM components such as lumican (LUM; a keratan sulfate proteoglycan which is normally diffusely present in peripheral lung tissue, mainly in vessel walls [21]), tenascin C (TNC; a proteoglycan expressed in the lung parenchyma in disease states such as asthma, idiopathic pulmonary fibrosis and acute lung injury [22], where it may play a role in modulating the influx of inflammatory cells) and elastin (ELN). Amongst the remaining genes are a number of metalloproteinases (MMP-2, −7, −9 and −13). These enzymes, which broadly facilitate extracellular matrix remodeling and degradation, can also modulate the activities of many extracellular and intracellular proteins, thereby regulating cell proliferation, adhesion, migration, growth factor bioavailability, chemotaxis, and signalling. In particular, evidence suggests that MMP-7 and MMP-9 may act in concert to facilitate airway re-epithelialization [23]–[26] and the co-localization of MMP-2 and MMP-9 to the epithelial-stromal interface behind migrating epithelial cells in anterior keratectomy corneal wounds might further suggest their involvement in remodeling of the stroma and reformation of the basement membrane [27]. Excessive or inappropriate expression of metalloproteinases may contribute to the pathogenesis of lung diseases. Indeed, levels of MMP-2 and MMP-9 protein and MMP7 mRNA are elevated in the lungs of patients with human idiopathic pulmonary fibrosis (IPF) [28]–[30] and MMP-13 expression is increased in the lungs of patients with chronic obstructive pulmonary disease (COPD) [31]. Given that tissue inhibitors of metalloproteinases (TIMPs) play an important role in regulating their activity the balance between enzyme and inhibitor might also dictate whether an initial injury resolves towards repair or remodelling. In this regard it is of note that airway epithelial pseudo-stratification and surface airway epithelial differentiation in an *in vivo* humanized airway xenograft model in nude mice was associated with increased expression of MMP-7 and MMP-9, but no change in the expression of tissue inhibitor of metalloproteinase-1 (TIMP-1) [32], whereas in the present study TIMP-1 was promptly and significantly up-regulated in the airway wall more than 3–fold (relative to baseline) at 6h with expression levels peaking at approximately 5-fold on d1 and d3 before declining at d7 (Table S1b). Our findings find concordance with models of acute lung injury in mice where early up-regulation of TIMP-1 occurs and plays a regulatory role by limiting inflammation and preserving pulmonary vascular integrity [33]. Other notable genes in this cluster include lysyl oxidase (LOX) and transforming growth factor beta 1 (TGFB1). The former plays a plays a critical role in the biogenesis of connective tissue matrices by crosslinking the extracellular matrix proteins, collagen and elastin [34] and the latter plays a central role in fibrogenesis with influence over fibroblast recruitment, myofibroblast differentiation, epithelial mesenchymal transition, and extracellular matrix deposition [35]. The transcriptional response of the human airway epithelium to similar physical injury 7 days after injury was dominated by cell cycle, signal transduction, metabolism and transport, and transcription genes [11].

This study also raises an important issue in relation to selecting drug targets. In order to be able to assess the effect of any intervention it would be important that the target selected showed a consistent pattern of expression (amongst experimental subjects) during the course of phenotypic change. It is clear from the present study that amongst a small cohort of outbred experimental animals, wherein considerable biological variation would be anticipated, it is still possible to identify genes that do cluster on the basis of coordinate expression. Where such clustering can be allayed to function, there lies the greatest opportunity to select optimal targets to perturb and potentially demonstrate phenotypic effect.

In defining the airway wall response during the first seven days following physical injury we were unable to characterise the events leading to complete repair or regeneration of the airway wall. This limitation was largely a consequence of the model itself in that a balance had to be sought between the experimental objectives and the associated ethical demands, involving repeated anaesthesia, placed on the animals. Naturally there will be future merit in extending the time course in a separate cohort of animals. That injury caused by bronchial brush biopsy does indeed eventually lead to repair is evidenced by our own experience (unpublished data) and previous data from rabbits where a normal ciliated epithelium is restored within 3 weeks [1].

We have demonstrated that the airway wall response to physical injury involves significant changes in baseline gene expression, and that such changes relate to the key biological processes that include the immune response, angiogenesis, cell proliferation and ECM remodelling. By clustering significantly altered genes on the basis of shared patterns of expression it was possible to extend this information base and search for gene clusters with shared functional annotation in order that the nature of their time-dependent change be fully realised. Seven days after injury, the predominant aspect of the response appeared to relate to ECM remodelling. The utility of this model in helping define the pathophysiology of the normal response to injury in the airway has been established. The potential of this model in defining how such responses deviate under conditions relevant to human lung disease remains to be realised.

## Materials and Methods

Detailed descriptions of methodologies are provided in the online supplement (Data S1).

### Ethics Statement

All experimental procedures were subjected to ethical review at the University of Edinburgh and were performed under licence, as specified by the Animals (Scientific Procedures) Act 1986.

### Animals, experimental design and bronchial brushing

Eight sheep were subjected to endobronchial brush biopsies (BBr) on three occasions prior to euthanasia and post mortem examination (PME). Two contralateral sites in the left and right lungs were selected at the initial time point and care was taken to select spatially disparate sites during subsequent time points. The four chosen time points were allocated such that pairs of sheep were exposed to BBr according to one of four possible time point combinations: d3∶d1∶6 h, d7∶d3∶6 h, d7∶d1∶6 h or d7∶d3∶d1 – with each time-point referring to the interval to PME. At PME, in addition to sampling tissue from sites previously subjected to BBr, additional tissue was harvested from naïve sites not previously exposed to BBr. Duplicating the sites (in right and left lungs) within each occasion facilitated the collection of material for parallel histological analysis [9].

### RNA isolation and quality assessment

At PME the sites subjected to BBr were identified and the airway wall at these sites carefully dissected free of surrounding parenchyma. Total RNA was isolated from the airway wall tissue using RNeasy Mini kits (Qiagen, Crawley, West Sussex, UK).

### DNA Microarray, hybridization and analysis

RNA from 32 tissue extracts was quantified, assessed for quality using an Agilent Bioanalyser and processed according to Ark Genomics Standard Operation Procedures (http://www.ark-genomics.org/protocols/) for hybridization to Agilent 15 K sheep gene expression microarrays.

### Microarray statistical analysis

Samples were allocated at random to arrays within slides to avoid systematic variation between slides or arrays caused by the measurement process biasing the comparison of treatments. Analysis of the microarray data was performed using a linear mixed model fitted by residual maximum likelihood (REML) on the log_2_-transformed gMeanSignal. The experiment was deposited in GEO (GSE37086).

### Microarray validation study

Semi-quantitative RT-PCR assay on eight genes from the microarray was performed to validate the array approach. The expression values detected by the semi-quantitative RT-PCR assay were significantly correlated with the expression values detected by the array for all the genes in question (p<0.05).

### Array annotation

In order to extend the annotation of the probe sets contained on the array a locally installed BLAST client [16], blastcl3, was used to batch search multiple sequences for homologous manually curated transcripts in the bovine and human refseq RNA sequence databases. In addition, use was made of the Information System of AGENAE program resource (SIGENAE (http://www.sigenae.org/webcite sheep oligo annotation version 8 of February 2011) which publishes the most recent annotation for many commercially available aquaculture and farm animal species arrays. Annotation information was thus obtained for 6635 unique genes, targeted by 8729 probes. The derived gene identity information was used to query the DAVID knowledge database (http://david.abcc.ncifcrf.gov; version 2008) for functional clustering analysis using the human genome as a reference list in forward analysis.

### Visualisation of co-ordinately expressed genes

In order to visualise and facilitate functional annotation of co-ordinately expressed genes expression data relating to the genes that were significantly differentially regulated (with greater than twofold change in expression) at least once during the course of the injury response was selected for input to BioLayout Express 3D (http://www.biolayout.org) [17] and the functional annotation of derived gene clusters again queried using the DAVID knowledge database.

## Supporting Information

Data S1 (DOC)

Table S1 a: The number of probes within the microarray demonstrating significant (p<0.05) up-, or down-regulation at each time point. The number of these significantly regulated probes also demonstrating more than a two-fold change in expression relative to baseline is also depicted (>two-fold). b: Complete list of all the annotated probes that were significantly differentially regulated (P<0.05) with greater than a two-fold change in level of expression at least once during the course of the response to physical injury.
(DOC)

Table S2 a: The results of functional annotation clustering analysis, using the DAVID knowledge database (http://david.abcc.ncifcrf.gov; version 2008), applied to the significantly down-regulated annotated genes showing a greater than two-fold change in expression at 6 h (n = 561). Only clusters with an enrichment score of 4.0 or above, and a significance level <0.05 are tabulated. The complete GO gene-associations database for the human genome was used as a reference list. DAVID determined all the annotated biologic process GO terms that existed both in the background gene list and those associated with our genes of interest. The GO FAT option was chosen to select a subset of the GO term set relating only to biological process. The number of appearances of each GO term was counted and compared between the groups of interest and for the reference genes. A modified Fisher exact test was calculated for all analyses and the Benjamini-Hochberg method was used to control the false discovery rate for the enrichment p-values for the given individual term members. b: The results of functional annotation clustering analysis, using the DAVID knowledge database (http://david.abcc.ncifcrf.gov; version 2008), applied to the significantly up-regulated annotated genes showing a greater than two-fold change in expression at 6 h (n = 154). See legend for table 1a for description of table derivation.
(DOC)

Table S3 a: The results of functional annotation clustering analysis, using the DAVID knowledge database (http://david.abcc.ncifcrf.gov; version 2008), applied to the significantly up-regulated annotated genes showing a greater than two-fold change in expression at d1 (n = 324). See legend for table 1a for description of table derivation. b: The results of functional annotation clustering analysis, using the DAVID knowledge database (http://david.abcc.ncifcrf.gov; version 2008), applied to the significantly up-regulated annotated genes showing a greater than two-fold change in expression at d1 (n = 324). See legend for table 1a for description of table derivation.
(DOC)

Table S4 a: The results of functional annotation clustering analysis, using the DAVID knowledge database (http://david.abcc.ncifcrf.gov; version 2008), applied to the significantly up-regulated annotated genes showing a greater than two-fold change in expression at d3 (n = 366). See legend for table 1a for description of table derivation. b: The results of functional annotation clustering analysis, using the DAVID knowledge database (http://david.abcc.ncifcrf.gov; version 2008), applied to the significantly up-regulated annotated genes showing a greater than two-fold change in expression at d3 (n = 366). See legend for table 1a for description of table derivation. c: The results of functional annotation clustering analysis, using the DAVID knowledge database (http://david.abcc.ncifcrf.gov; version 2008), applied to the significantly up-regulated annotated genes showing a greater than two-fold change in expression at d3 (n = 366). See legend for table 1a for description of table derivation. d: The results of functional annotation clustering analysis, using the DAVID knowledge database (http://david.abcc.ncifcrf.gov; version 2008), applied to the significantly up-regulated annotated genes showing a greater than two-fold change in expression at d3 (n = 366). See legend for table 1a for description of table derivation. e: The results of functional annotation clustering analysis, using the DAVID knowledge database (http://david.abcc.ncifcrf.gov; version 2008), applied to the significantly up-regulated annotated genes showing a greater than two-fold change in expression at d3 (n = 366). See legend for table 1a for description of table derivation.
(DOC)

Table S5 a: The results of functional annotation clustering analysis, using the DAVID knowledge database (http://david.abcc.ncifcrf.gov; version 2008), applied to the significantly down-regulated annotated genes showing a greater than two-fold change in expression at d7 (n = 68). See legend for table 1a for description of table derivation. b: The results of functional annotation clustering analysis, using the DAVID knowledge database (http://david.abcc.ncifcrf.gov; version 2008), applied to the significantly up-regulated annotated genes showing a greater than two-fold change in expression at d7 (n = 197). See legend for table 1a for description of table derivation. c: The results of functional annotation clustering analysis, using the DAVID knowledge database (http://david.abcc.ncifcrf.gov; version 2008), applied to the significantly up-regulated annotated genes showing a greater than two-fold change in expression at d7 (n = 197). See legend for table 1a for description of table derivation. d: The results of functional annotation clustering analysis, using the DAVID knowledge database (http://david.abcc.ncifcrf.gov; version 2008), applied to the significantly up-regulated annotated genes showing a greater than two-fold change in expression at d7 (n = 197). See legend for table 1a for description of table derivation. e: The results of functional annotation clustering analysis, using the DAVID knowledge database (http://david.abcc.ncifcrf.gov; version 2008), applied to the significantly up-regulated annotated genes showing a greater than two-fold change in expression at d7 (n = 197). See legend for table 1a for description of table derivation. f: The results of functional annotation clustering analysis, using the DAVID knowledge database (http://david.abcc.ncifcrf.gov; version 2008), applied to the significantly up-regulated annotated genes showing a greater than two-fold change in expression at d7 (n = 197). See legend for table 1a for description of table derivation.
(DOC)
