# Supplementary material for: Gene Expression Changes Associated with the Airway Wall Response to Injury
Source: PLoS One. 2013 Apr 9;8(4):e58930. doi: 10.1371/journal.pone.0058930 (PMC3621906; doi:10.1371/journal.pone.0058930)
Supplement: Data S1 — (DOC) [file pone.0058930.s001.doc]

Gene expression changes associated with the airway wall response to injury

Badrul Yahaya, Gerry McLachlan, Caroline McCorquodale, David Collie

ONLINE DATA SUPPLEMENT

**Animals, experimental design and bronchial brushing**

Eight sheep (n=8) [(breed = Suffolk Cross), (sex; Male (neutered) = 4 (Sheep IDs: 7954, 8039, 8003, 8020), Female = 4 (Sheep IDs: 7958, 45, 8064, 8038)), (Age; means = 14.75 ± 0.45 months old), (bodyweight; means = 54.5 ± 3.74 kg)] were involved. Lung health was ascribed on the basis of bronchoscopic examination undertaken approximately 2 weeks (14.8 + 6.2 days) prior to entry into the described experimental protocol. Such visual examination, in determining the absence of airway disease, was later confirmed by necropsy examination at gross and light microscopic levels. The design of the experiment was previously described (1). In brief, bronchial brushings (BBr) were performed on anaesthetised sheep at two predefined sites within the major airways at different time-points prior to euthanasia. Those time-points were 6 hours, 1 day, 3 days and 7 days prior to euthanasia. Each of the eight sheep was subjected to a protocol which involved the two brushings being applied on only three (of the possible four) occasions. At necropsy, one site was sampled for histopathological analysis (1), and one was made available for transcriptional analysis, giving 24 samples from brushed sites for microarray analysis. In addition, a further naïve airway site, from an area of the lung not subjected to bronchoscopy was sampled from each of the eight sheep, giving a total of 32 samples for transcriptional analysis.

All experimental procedures were subjected to ethical review at the University of Edinburgh and were performed under licence, as specified by the Animals (Scientific Procedures) Act 1986.

**Tissue samples and processing**

At necropsy examination, the lung segments were carefully mapped and dissected out to identify the injured sites. Each injured airway site was immediately cut into 4 or 5 pieces and those tissues transferred to the RNA*later*® (Ambion, Inc., Austin, TX) and stored at -80^o^C.

**Total RNA isolation**

Total RNA was isolated from 100mg of cartilaginous airway tissue and homogenised using a FastPrep instrument with previously optimised time (T) and speed (S) settings. The RNA isolation procedure was performed using RNeasy Mini kits (Qiagen, Crawley, West Sussex, UK) following the manufacturer’s instructions.

**RNA quality control assessment**

The quality and yield of the extracted RNA was initially measured with a NanoDrop ND-100 Spectrophotometer (Thermo Fisher Scientific Inc, UK) to determine the RNA concentration and A260/A280 ratio. Samples were analysed using an Agilent Bioanalyzer 2100 to generate electropherogram profiles and RIN scores. RNA samples were considered to possess good quality and integrity when the RIN exceeded 6.0 and two distinct peaks for 18S and 28S ribosomal RNA were clearly observed.

**RNA Amplification, cDNA synthesis, labelling, hybridization and scanning**

For the microarray experiment, all the procedures including RNA amplification, and cDNA synthesis, labelling and hybridization, were carried out at Ark Genomics, Roslin Institute, UK following Ark Genomics Standard Operation Procedures (http://www.ark-genomics.org/protocols/).

Four slides containing eight arrays (n=8) each were used for the microarray study. As described previously, there were six replicates (n=6) for each of the four time points which were all taken from different sheep. One segment from each of the treated sheep was used as a naive control (n=8 naive controls). Sheep were allocated at random to Agilent slides and samples were allocated at random to arrays within slides so that any variation between slides or arrays caused by the measurement process should not bias the comparison of treatments. The allocated positions of each of the samples within the slides are shown in Figure 1.

The glass slides containing the RNA samples were scanned using the auto photo multiplier tube (PMT) Axon 4200AL scanner following the manufacturer's standard operation procedures. The microarray expression data was extracted using the Agilent Feature Extraction (FE) software version 9.5.3. The gMeanSignal used in this analysis is one of the basic values generated by the FE software. Raw signal is calculated, based only on the "inliers" pixels, meaning all the pixels in a spot retained after removing saturated, or not "well above background" pixels. The CookieCutter method was used to select all the pixels used in the calculation.

Quality control check were performed on the microarray data output generated from FE software. The quality control parameters followed were as recommended by the manual for the Agilent Feature Extraction Software (version 9.5.3) for one colour.

**Microarray statistical analysis**

Four microarray slides (Sheep Gene Expression Microarray, Agilent Technologies, Santa Clara, CA, USA), each containing eight arrays were used. Each of the four separate time points (6h, d1, d3 and d7) were represented by six samples from separate sheep, and a further sample was derived from naïve control airways derived from an untreated area of the lung of each sheep, giving a total of 32 samples presented for analysis. Sheep were allocated at random to each half of an Agilent slide and samples within sheep were allocated at random to arrays within slides. This approach was followed in order to avoid systematic variation between slides or arrays caused by the measurement process biasing the comparison of treatments. Accordingly, because arrays had samples from different sheep (and would therefore be subject to biological variation) it was deemed inappropriate to apply between-slide normalisation procedures.

The nature of the experimental protocol dictated that each sheep was only sampled on three occasions – thus giving rise to a possible four different sampling schedules (6h, d1 and d3; 6h, d1 and d7; 6h, d3 and d7 and d1, d3 and d7) with two sheep assigned to each. The sampling schedule itself could therefore potentially bias the nature of gene expression changes measured relative to naïve control airways. To compensate for any interaction between the time of the brushing (Time point) and the sampling schedule (combination of time points), the brushed sites were only compared to naive control airway from the same animals. Analysis of the microarray data was performed using a linear mixed model fitted by residual maximum likelihood (REML) on the log_2_-transformed gMeanSignal (2).

**Microarray validation study**

Semi-quantitative RT-PCR was performed on eight selected genes (IL6, keratin 19 (KRT19), keratin 6A (KRT6A), cell division cycle 2 (CDC2), cyclin B1 (CCNB1), Cyclin B2 (CCNB2), keratin 5 (KRT5) and tubulin beta (TUBB)) from the microarray experiment in order to validate the observed changes. Four samples for baseline and each time point (total n=20 samples) were selected and used in this validation. Criteria used in selecting the chosen genes included the necessity of the genes in question demonstrating a highly significant (p<0.001) change in expression (up or down) at one or more time points, either high or low fold change at one or more time points, and those genes playing a relevant biological role in the context of the response to physical injury such as in cell cycle, immune response, cell communication, cell adhesion and/or cellular differentiation. The full list of genes used for RT-PCR assay is listed in Table 1. Data for these genes were normalised to ATPase gene expression.

The gene annotations for the probes on the sheep array were obtained by blasting the mRNA sequences of these genes through the nucleotide mRNA database using the NCBI programme available at http://blast.ncbi.nlm.nih.gov/Blast.cgi. The selection criteria for selecting the annotations for the sequences of interest were based on a sequence similarity of more than 90% with the bovine mRNA sequence database. The mRNA sequences for these genes were used to design primers using http://biotools.umassmed.edu/bioapps/primer3_www.cgi. The list of the primers and the PCR conditions are shown in Table 1.

In order to assess the correlation between expression data obtained from the array and semi-quantitative RT-PCR, a Pearson correlation coefficient test was carried out using R (R Development core team, 2010) (3) with p<0.05 considered significant. The array data for some of the genes were log-transformed due to the fact that they were not normally distributed. The tests demonstrated that the expression values detected by the semi-quantitative RT-PCR assay were significantly correlated with the expression values detected by the array for all the genes in question (p<0.05).

**RNA quality control check**

The first quality control check for the RNA samples was carried out immediately after isolation using the conventional spectrophotometric method of RNA quantification. The A260/A280 ratios for all samples were within the recommended range of RNA purity between of 1.9 to 2.15 while the average RNA concentration was 109.2±77.67 ng/µl. The second quality control check was performed using the Agilent 2100 Bioanalyzer to assess the RNA integrity and quality. An average RIN score of 7.42 ± 0.63 was achieved (range - ).

**Quality control of the microarray output**

In order to ensure their reproducibility and reliability, the outputs generated from the Agilent Feature Extraction (FE) software were analyzed. The standard QC metrics for one colour arrays included spot finding of the four corners of each array, spatial distribution of the outliers and the histogram of the signals across all non-control probes. The FE output profiles of the arrays used in this study indicated that the microarray data generated were reliable and of good quality. In this regard the quality control reports from the feature extraction showed that there were no spatial abnormalities and the spike in control profiles in both the feature extraction and other analysis programs (i.e GeneSpring) showed that the arrays were performing normally and no array(s) were performing exceptionally. Indeed the chip spike-in control signal profiles behaved similarly and there were no obvious outliers.

Examination of the spatial distribution of all outliers on the arrays demonstrated no systematic bias within arrays. In terms of between-array and between-slide normalization, a plot of the log2gMean signals across the four slides, organised according to column and row position on the slide indicated that there was no systematic (ie shared between all slides) bias detectable.

The spread of the intensity log ratios was largely similar across slides and importantly there was no evidence of spatial bias. These observations, in conjunction with the additional knowledge that the time points were randomly allocated between rows within columns (sheep), indicate that any detected significant differences in gene expression between time points are more likely to be a true reflection of biological variation and not influenced by systematic procedural bias.

The complete data set is publicly available in the GEO (Gene Expression Omnibus) public repository (http://www.ncbi.nlm.nih.gov/geo/) (accession no. GSE37086) in a format that complies with the Minimal Information About a Microarray Experiment (MIAME) guidelines.

**Extending the annotation of the array**

Out of the 7786 differentially regulated probes on the Agilent ovine array, annotation information was only available for a limited number of targeted genes. The annotation information was therefore expanded by using a locally installed BLAST client (4), blastcl3, to batch search multiple sequences for homologous manually curated transcripts in the bovine and human refseq RNA sequence databases. The NM identifier was then used as input into IDconverter, the online tool available for conversion and annotation of gene and protein IDs (http://idconverter.bioinfo.cnio.es/), to yield the Gene Name (taken from UniGene), Ensembl transcript ID, UniGene ID, EntrezGene ID, the relevant GenBank Accession numbers and Clone IDs and information relating to chromosomal position. In addition, use was made of the Information System of AGENAE program resource (SIGENAE (http://www.sigenae.org/ webcite sheep oligo annotation version 8 of February 2011) which publishes the most recent annotation for many commercially available aquaculture and farm animal species arrays. This combined approach led to the annotation of 1434 probes out of a possible total of 2009 that were significantly differentially regulated and underwent a two-fold change in expression at one or more time points after physical injury.

The Entrez Gene IDs for these annotated genes were then queried with the DAVID knowledge database (http://david.abcc.ncifcrf.gov; version 2008) for functional clustering analysis. The complete GO gene-associations database for the human genome was used as a reference list in forward analysis and the significantly up and down regulated genes from each time point were separately queried. In the analysis, DAVID determined all the annotated biologic process GO terms that existed both in the background gene list and those associated with our genes of interest. The GO FAT option was chosen to select a subset of the GO term set relating only to biological process. The number of appearances of each GO term was counted and compared between the groups of interest and for the reference genes. A modified Fisher exact test was calculated for all analyses and the Benjamini-Hochberg method was used to control the false discovery rate for the enrichment p-values for the given individual term members.

**BioLayout Express 3D**

In order to facilitate visualisation of the dynamic changes in gene expression that occur during the airway response to physical injury, expression data relating to the 1145 unique genes that were significantly differentially regulated (with greater than twofold change in expression) at least once during the course of the response to physical injury was selected for input to BioLayout Express 3D (http://www.biolayout.org) (5) using the lowest correlation setting (0.7). Markov clustering using default settings and node grouping yielded a graph image in which the nodes sharing similar time-dependent patterns of expression were clustered into enlarged nodes, the size of which reflected the number of nodes contained therein. In order to determine whether any function was shared by the genes within each node, the relevant gene lists were again queried with the DAVID knowledge database for functional clustering analysis. Where such analysis identified significantly enriched clusters after correction for FDR (Benjamini p<0.05) the terms relating to biological process were considered.

1. Yahaya B, Baker A, Tennant P, Smith SH, Shaw DJ, McLachlan G, Collie DD. Analysis of airway epithelial regeneration and repair following endobronchial brush biopsy in sheep. *Exp Lung Res* 2011;37:519-535.

2. Patterson, Thompson R. Recovery of inter-block information when block sizes are unequal. *Biometrika* 1971;58:545-&.

3. R Development Core Team. R: A language and environment for statistical computing. Vienna, Austria: R Foundation for Statistical Computing.

4. Altschul SF, Madden TL, Schäffer AA, Zhang J, Zhang Z, Miller W, Lipman DJ. Gapped blast and psi-blast: A new generation of protein database search programs. *Nucleic Acids Res* 1997;25:3389-3402.

5. Theocharidis A, van Dongen S, Enright AJ, Freeman TC. Network visualization and analysis of gene expression data using biolayout express(3d). *Nat Protoc* 2009;4:1535-1550.
