# Supplementary material for: Gene Expression Changes Associated with the Airway Wall Response to Injury
Source: PLoS One. 2013 Apr 9;8(4):e58930. doi: 10.1371/journal.pone.0058930 (PMC3621906; doi:10.1371/journal.pone.0058930)
Supplement: Table S1 — a: The number of probes within the microarray demonstrating significant (p<0.05) up-, or down-regulation at each time point. The number of these significantly regulated probes also demonstrating more than a two-fold change in expression relative to baseline is also depicted (>two-fold). b: Complete list of all the annotated probes that were significantly differentially regulated (P<0.05) with greater than a two-fold change in level of expression at least once during the course of the response to physical injury. (DOC) [file pone.0058930.s002.doc]

Table S1a

|  | Time point | | | | | | | |
| --- | --- | --- | --- | --- | --- | --- | --- | --- |
|  | 6h | | d1 | | d3 | | d7 | |
|  | p<0.05 | >two-fold | p<0.05 | >two-fold | p<0.05 | >two-fold | p<0.05 | >two-fold |
| Up regulated | 528 | 207 | 1331 | 413 | 1599 | 536 | 857 | 335 |
| Down regulated | 4229 | 1024 | 1454 | 309 | 1957 | 354 | 1548 | 124 |

Table S1b

| **Probenames** | **Gene Symbol** | **Gene Name** | **6h vs Baseline**  **Fold**  **Change P** | | **d1 vs Baseline**  **Fold**  **Change P** | | **d3 vs Baseline**  **Fold**  **Change P** | | **d7 vs Baseline**  **Fold**  **Change P** | |
| --- | --- | --- | --- | --- | --- | --- | --- | --- | --- | --- |
| A_70_P000001 | ADA | adenosine deaminase | 1.24 | 0.248 | 1.99 | 0.002 | 2.09 | 0.001 | 1.17 | 0.392 |
| A_70_P000006 | ALCAM | activated leukocyte cell adhesion molecule | -2.42 | 0.001 | -1.86 | 0.012 | -2.44 | 0.001 | -1.58 | 0.050 |
| A_70_P000016 | KCNK1 | potassium channel, subfamily K, member 1 | 2.39 | 0.001 | 1.85 | 0.012 | 1.37 | 0.158 | -1.17 | 0.473 |
| A_70_P000061 | KRTDAP | keratinocyte differentiation-associated protein, transcript variant 2 | 1.05 | 0.859 | 1.32 | 0.339 | 8.55 | 0.000 | 2.01 | 0.027 |
| A_70_P000066 | CLEC4G | C-type lectin domain family 4, member G | -4.59 | 0.003 | -3.66 | 0.009 | -4.18 | 0.005 | -1.76 | 0.199 |
| A_70_P000116 | SULF2 | sulfatase 2 | 2.61 | 0.001 | 2.39 | 0.002 | 2.11 | 0.006 | 1.44 | 0.129 |
| A_70_P000166 | CILP | cartilage intermediate layer protein, nucleotide pyrophosphohydr | 1.50 | 0.215 | -1.56 | 0.173 | 1.40 | 0.298 | 3.86 | 0.001 |
| A_70_P000171 | LOC461186 | LIM and calponin homology domains 1 | -2.65 | 0.001 | -2.52 | 0.002 | -2.75 | 0.001 | -1.59 | 0.074 |
| A_70_P000191 | NCOA3 | nuclear receptor coactivator 3 | 3.08 | 0.000 | 2.49 | 0.000 | 1.70 | 0.015 | 1.24 | 0.277 |
| A_70_P000226 | AOX1 | aldehyde oxidase 1 | -2.17 | 0.007 | -1.73 | 0.039 | -2.74 | 0.001 | -2.02 | 0.012 |
| A_70_P000276 | SLC39A8 | solute carrier family 39 (zinc transporter), member 8 | 3.45 | 0.000 | 2.06 | 0.010 | 1.26 | 0.350 | -1.27 | 0.332 |
| A_70_P000361 | ABLIM1 | actin binding LIM protein 1 | -2.69 | 0.000 | -1.67 | 0.010 | -1.27 | 0.181 | -1.47 | 0.040 |
| A_70_P000466 | MRPL51 | mitochondrial ribosomal protein L51 | 1.47 | 0.257 | 1.59 | 0.177 | 2.10 | 0.040 | -1.41 | 0.309 |
| A_70_P000531 | MCTP1 | multiple C2 domains, transmembrane 1 | -2.25 | 0.003 | -1.34 | 0.193 | -1.06 | 0.791 | 1.18 | 0.448 |
| A_70_P000556 | LOC100140190 | Major allergen I polypeptide chain 1 precursor (Allergen Fel d 1-A) (Fel d I-A) (Allergen Cat-1) (Fel dI) (AG4) (FdI) | -1.19 | 0.666 | -1.25 | 0.581 | -1.26 | 0.562 | -3.63 | 0.006 |
| A_70_P000596 | LOC100153301 | aldehyde dehydrogenase 6A1 | -2.64 | 0.000 | -1.75 | 0.004 | -1.27 | 0.148 | -1.41 | 0.049 |
| A_70_P000741 | MTMR7 | myotubularin related protein 7 | -2.33 | 0.001 | -1.95 | 0.006 | -1.63 | 0.031 | -1.69 | 0.022 |

| **Probenames** | **Gene Symbol** | **Gene Name** | **6h vs Baseline**  **Fold**  **Change P** | | **d1 vs Baseline**  **Fold**  **Change P** | | **d3 vs Baseline**  **Fold**  **Change P** | | **d7 vs Baseline**  **Fold**  **Change P** | |
| --- | --- | --- | --- | --- | --- | --- | --- | --- | --- | --- |
| A_70_P000746 | FAM102B | family with sequence similarity 102, member B | -3.84 | 0.000 | -2.43 | 0.006 | -1.96 | 0.027 | 1.00 | 0.994 |
| A_70_P000771 | ITK | IL2-inducible T-cell kinase | -2.56 | 0.004 | -1.75 | 0.059 | -1.80 | 0.048 | 1.15 | 0.611 |
| A_70_P001351 | CDAN1 | congenital dyserythropoietic anemia, type I | -2.35 | 0.000 | -1.72 | 0.008 | -2.07 | 0.001 | -1.64 | 0.013 |
| A_70_P001431 | TXNIP | thioredoxin interacting protein | -2.12 | 0.001 | -1.75 | 0.007 | -2.05 | 0.001 | -1.50 | 0.038 |
| A_70_P001471 | GAL | galanin prepropeptide | 3.33 | 0.000 | 2.93 | 0.001 | 2.54 | 0.003 | 1.67 | 0.060 |
| A_70_P001472 | GAL | galanin prepropeptide | 3.28 | 0.000 | 2.92 | 0.001 | 2.49 | 0.003 | 1.78 | 0.035 |
| A_70_P001556 | MBP | myelin basic protein | -2.11 | 0.006 | -1.86 | 0.017 | -2.44 | 0.002 | -1.38 | 0.179 |
| A_70_P001561 | FOS | FBJ murine osteosarcoma viral oncogene homolog | 2.02 | 0.032 | 1.10 | 0.748 | -1.22 | 0.516 | -1.08 | 0.799 |
| A_70_P001658 | COL3A1 | collagen, type III, alpha 1 | -1.33 | 0.149 | -1.20 | 0.351 | 2.47 | 0.000 | 3.82 | 0.000 |
| A_70_P001691 | KRT5 | keratin 5 | 1.51 | 0.060 | 2.40 | 0.001 | 2.51 | 0.001 | 1.79 | 0.012 |
| A_70_P001746 | KRT81 | keratin 81 | 1.50 | 0.117 | 2.65 | 0.002 | 3.28 | 0.000 | 2.36 | 0.004 |
| A_70_P001766 | LUM | lumican | -1.47 | 0.094 | -1.28 | 0.271 | 2.54 | 0.001 | 2.38 | 0.001 |
| A_70_P001776 | SLC6A5 | solute carrier family 6 (neurotransmitter transporter, glycine), | -2.08 | 0.010 | -1.74 | 0.040 | -1.78 | 0.033 | -1.22 | 0.419 |
| A_70_P001791 | LOC100008589 | 28S ribosomal RNA | 1.83 | 0.018 | 2.21 | 0.004 | 1.77 | 0.023 | -1.04 | 0.876 |
| A_70_P001801 | COL3A1 | collagen, type III, alpha 1 | -1.16 | 0.353 | 1.18 | 0.304 | 2.43 | 0.000 | 2.54 | 0.000 |
| A_70_P001841 | TUBB | beta-tubulin | 1.16 | 0.149 | 2.15 | 0.000 | 1.49 | 0.001 | 1.57 | 0.001 |
| A_70_P001842 | LOC443015 | beta-tubulin | 1.15 | 0.405 | 2.38 | 0.000 | 1.61 | 0.011 | 1.49 | 0.028 |
| A_70_P001891 | COL1A1 | collagen, type I, alpha 1 | 1.17 | 0.673 | 1.97 | 0.092 | 10.81 | 0.000 | 8.26 | 0.000 |
| A_70_P001921 | COL1A2 | collagen, type I, alpha 2 | 1.01 | 0.966 | 1.29 | 0.217 | 3.46 | 0.000 | 2.89 | 0.000 |
| A_70_P001936 | COL6A3 | collagen, type VI, alpha 3 | -1.12 | 0.542 | 1.30 | 0.163 | 2.38 | 0.000 | 2.59 | 0.000 |

| **Probenames** | **Gene Symbol** | **Gene Name** | **6h vs Baseline**  **Fold**  **Change P** | | **d1 vs Baseline**  **Fold**  **Change P** | | **d3 vs Baseline**  **Fold**  **Change P** | | **d7 vs Baseline**  **Fold**  **Change P** | |
| --- | --- | --- | --- | --- | --- | --- | --- | --- | --- | --- |
| A_70_P001991 | COL1A1 | collagen, type I, alpha 1 (COL1A1) | -1.04 | 0.893 | 1.94 | 0.025 | 5.18 | 0.000 | 4.38 | 0.000 |
| A_70_P002146 | DOK2 | docking protein 2, 56kDa | -2.36 | 0.002 | -1.42 | 0.141 | -1.06 | 0.785 | 1.38 | 0.172 |
| A_70_P002156 | FAM111B | family with sequence similarity 111, member B | -1.24 | 0.155 | 6.02 | 0.000 | 3.90 | 0.000 | 2.00 | 0.000 |
| A_70_P002211 | ACE2 | angiotensin I converting enzyme (peptidyl-dipeptidase A) 2 | 1.31 | 0.184 | 2.36 | 0.001 | 1.41 | 0.094 | 1.21 | 0.331 |
| A_70_P002266 | ZBTB44 | zinc finger and BTB domain containing 44 | -2.17 | 0.004 | -1.83 | 0.016 | -1.62 | 0.044 | -1.57 | 0.057 |
| A_70_P002311 | LOC614107 | similar to Hexokinase-2 (Hexokinase type II) (HKII) | 2.18 | 0.003 | 1.82 | 0.015 | 1.60 | 0.046 | 1.23 | 0.341 |
| A_70_P002401 | CASP1 | caspase 1, apoptosis-related cysteine peptidase (interleukin 1, | -2.58 | 0.002 | -2.11 | 0.010 | -1.69 | 0.053 | -1.24 | 0.393 |
| A_70_P002456 | AC130454.1 | NULL | -2.49 | 0.001 | -2.22 | 0.002 | -1.36 | 0.144 | -1.20 | 0.374 |
| A_70_P002481 | FABP7 | fatty acid binding protein 7, brain | -1.03 | 0.925 | 1.10 | 0.753 | 7.08 | 0.000 | 2.66 | 0.007 |
| A_70_P002546 | CA12 | carbonic anhydrase XII | 1.67 | 0.142 | 1.70 | 0.131 | 2.53 | 0.015 | -1.24 | 0.522 |
| A_70_P002596 | KRT19 | keratin 19 (KRT19) | 7.33 | 0.001 | 16.99 | 0.000 | 20.43 | 0.000 | 13.51 | 0.000 |
| A_70_P002626 | CLUAP1 | clusterin associated protein 1 | -2.26 | 0.002 | -1.51 | 0.078 | -1.72 | 0.026 | -1.50 | 0.082 |
| A_70_P002641 | RIOK1 | RIO kinase 1 (yeast) | 2.24 | 0.001 | 2.40 | 0.001 | 1.76 | 0.010 | -1.12 | 0.565 |
| A_70_P002691 | EGLN3 | egl nine homolog 3 (C. elegans) | 1.87 | 0.010 | 2.01 | 0.005 | 1.98 | 0.006 | 1.89 | 0.010 |
| A_70_P002706 | FCER1G | Fc fragment of IgE, high affinity I, receptor for; gamma polypep | -2.01 | 0.035 | 1.20 | 0.542 | 1.63 | 0.120 | 1.15 | 0.647 |
| A_70_P002712 | TEK | TEK tyrosine kinase, endothelial | -2.08 | 0.019 | -1.58 | 0.117 | -1.75 | 0.061 | -1.00 | 0.990 |

| **Probenames** | **Gene Symbol** | **Gene Name** | **6h vs Baseline**  **Fold**  **Change P** | | **d1 vs Baseline**  **Fold**  **Change P** | | **d3 vs Baseline**  **Fold**  **Change P** | | **d7 vs Baseline**  **Fold**  **Change P** | |
| --- | --- | --- | --- | --- | --- | --- | --- | --- | --- | --- |
| A_70_P002716 | TIE1 | tyrosine kinase with immunoglobulin-like and EGF-like domains 1 | -2.34 | 0.001 | -1.01 | 0.953 | 1.42 | 0.116 | 1.61 | 0.040 |
| A_70_P002717 | TIE1 | tyrosine kinase with immunoglobulin-like and EGF-like domains 1 | -2.26 | 0.002 | 1.01 | 0.967 | 1.46 | 0.092 | 1.69 | 0.026 |
| A_70_P002726 | CD1D | CD1d molecule | -2.41 | 0.003 | -1.26 | 0.348 | -1.65 | 0.051 | -1.19 | 0.480 |
| A_70_P002727 | CD1D | CD1d molecule | -2.28 | 0.006 | -1.32 | 0.287 | -1.63 | 0.074 | -1.21 | 0.453 |
| A_70_P002796 | UCP3 | uncoupling protein 3 (mitochondrial, proton carrier) | -6.03 | 0.003 | -3.25 | 0.031 | -4.80 | 0.007 | -1.41 | 0.489 |
| A_70_P002797 | UCP3 | uncoupling protein 3 (mitochondrial, proton carrier) | -6.03 | 0.003 | -3.18 | 0.033 | -4.74 | 0.007 | -1.40 | 0.499 |
| A_70_P002816 | CHI3L1 | signal processing protein | 5.01 | 0.000 | 11.05 | 0.000 | 5.41 | 0.000 | 3.53 | 0.000 |
| A_70_P002876 | ASPM | asp (abnormal spindle) homolog, microcephaly associated (Drosoph | -1.38 | 0.026 | 4.02 | 0.000 | 5.53 | 0.000 | 2.41 | 0.000 |
| A_70_P002901 | ANGPT1 | angiopoietin 1 | -2.73 | 0.000 | -2.05 | 0.004 | -1.24 | 0.316 | 1.25 | 0.292 |
| A_70_P002906 | ANGPT2 | angiopoietin 2 | -2.09 | 0.027 | -1.40 | 0.273 | -1.23 | 0.491 | 1.03 | 0.918 |
| A_70_P002907 | ANGPT2 | angiopoietin 2 | -2.01 | 0.031 | -1.39 | 0.272 | -1.19 | 0.545 | 1.06 | 0.838 |
| A_70_P002921 | PMCH | pro-melanin-concentrating hormone | -1.35 | 0.031 | 4.09 | 0.000 | 4.42 | 0.000 | 2.39 | 0.000 |
| A_70_P002922 | PMCH | pro-melanin-concentrating hormone | -1.29 | 0.025 | 3.52 | 0.000 | 4.31 | 0.000 | 2.20 | 0.000 |
| A_70_P002931 | LGALS1 | lectin, galactoside-binding, soluble, 1 | -1.20 | 0.375 | 1.18 | 0.431 | 2.02 | 0.004 | -1.07 | 0.730 |
| A_70_P002991 | LOC777598 | keratin associated protein-like | 3.58 | 0.011 | 3.11 | 0.020 | 4.36 | 0.005 | -1.24 | 0.617 |
| A_70_P003056 | CHAC1 | ChaC, cation transport regulator homolog 1 (E. coli) | 2.74 | 0.002 | 1.98 | 0.023 | 2.31 | 0.008 | -1.02 | 0.945 |
| A_70_P003081 | DPYSL2 | dihydropyrimidinase-like 2 | -2.17 | 0.003 | -1.41 | 0.130 | -1.43 | 0.119 | 1.37 | 0.164 |

| **Probenames** | **Gene Symbol** | **Gene Name** | **6h vs Baseline**  **Fold**  **Change P** | | **d1 vs Baseline**  **Fold**  **Change P** | | **d3 vs Baseline**  **Fold**  **Change P** | | **d7 vs Baseline**  **Fold**  **Change P** | |
| --- | --- | --- | --- | --- | --- | --- | --- | --- | --- | --- |
| A_70_P003126 | C1S | complement component 1, s subcomponent | 1.12 | 0.611 | 1.56 | 0.057 | 2.31 | 0.002 | 3.02 | 0.000 |
| A_70_P003141 | S100A2 | S100 calcium binding protein A2 | 5.62 | 0.000 | 4.73 | 0.000 | 4.44 | 0.000 | 1.32 | 0.197 |
| A_70_P003186 | SC4MOL | sterol-C4-methyl oxidase-like | -2.25 | 0.005 | -1.10 | 0.686 | -1.61 | 0.069 | -1.55 | 0.091 |
| A_70_P003211 | POSTN | periostin, osteoblast specific factor | -1.01 | 0.970 | 1.05 | 0.858 | 2.93 | 0.001 | 7.21 | 0.000 |
| A_70_P003381 | AC004223.3 | NULL | 1.47 | 0.044 | 2.15 | 0.001 | 1.81 | 0.004 | 1.18 | 0.350 |
| A_70_P003401 | CCNB1 | cyclin B1 | -1.23 | 0.142 | 7.70 | 0.000 | 8.58 | 0.000 | 2.82 | 0.000 |
| A_70_P003441 | REPS2 | RALBP1 associated Eps domain containing 2 | -2.20 | 0.003 | -1.30 | 0.232 | -1.33 | 0.192 | -1.31 | 0.224 |
| A_70_P003501 | TNS3 | tensin 3 | -2.20 | 0.015 | -1.69 | 0.085 | -2.16 | 0.017 | -1.14 | 0.651 |
| A_70_P003521 | CLEC4E | C-type lectin domain family 4, member E | 1.31 | 0.411 | 1.59 | 0.169 | 4.27 | 0.001 | 3.36 | 0.002 |
| A_70_P003522 | CLEC4E | C-type lectin domain family 4, member E | 1.39 | 0.327 | 1.63 | 0.159 | 4.41 | 0.001 | 3.63 | 0.002 |
| A_70_P003541 | SAA3 | serum amyloid A3.2 | 7.39 | 0.000 | 7.36 | 0.000 | 5.37 | 0.000 | 5.05 | 0.000 |
| A_70_P003836 | TMOD1 | tropomodulin 1 | -1.93 | 0.008 | -1.82 | 0.013 | -2.29 | 0.002 | -1.53 | 0.059 |
| A_70_P003891 | CLDN18 | claudin 18 | -11.82 | 0.003 | -7.94 | 0.010 | -11.46 | 0.004 | -2.43 | 0.216 |
| A_70_P003946 | OFD1 | oral-facial-digital syndrome 1 | -2.02 | 0.006 | -1.50 | 0.079 | -1.63 | 0.040 | -1.30 | 0.241 |
| A_70_P003961 | ENPP5 | ectonucleotide pyrophosphatase/phosphodiesterase 5 (putative) | -2.11 | 0.005 | -1.45 | 0.114 | -1.96 | 0.009 | -1.41 | 0.139 |
| A_70_P003976 | RRS1 | RRS1 ribosome biogenesis regulator homolog (S. cerevisiae) | 2.18 | 0.001 | 1.86 | 0.003 | 1.15 | 0.426 | -1.02 | 0.921 |
| A_70_P003981 | HSPA6 | heat shock 70kDa protein 6 (HSP70B') | 1.94 | 0.040 | 1.54 | 0.161 | 2.03 | 0.031 | 1.26 | 0.435 |
| A_70_P004236 | RAMP1 | receptor (G protein-coupled) activity modifying protein 1 | -1.57 | 0.054 | -1.54 | 0.061 | -1.50 | 0.079 | -2.48 | 0.001 |
| A_70_P004271 | RRM2 | ribonucleotide reductase M2 | -1.65 | 0.014 | 13.81 | 0.000 | 9.23 | 0.000 | 4.83 | 0.000 |

| **Probenames** | **Gene Symbol** | **Gene Name** | **6h vs Baseline**  **Fold**  **Change P** | | **d1 vs Baseline**  **Fold**  **Change P** | | **d3 vs Baseline**  **Fold**  **Change P** | | **d7 vs Baseline**  **Fold**  **Change P** | |
| --- | --- | --- | --- | --- | --- | --- | --- | --- | --- | --- |
| A_70_P004326 | CETN3 | centrin, EF-hand protein, 3 | -2.33 | 0.000 | -1.26 | 0.198 | -1.02 | 0.897 | -1.19 | 0.320 |
| A_70_P004341 | DYSF | dysferlin, limb girdle muscular dystrophy 2B (autosomal recessiv | -2.44 | 0.001 | -1.46 | 0.095 | -1.59 | 0.048 | -1.10 | 0.667 |
| A_70_P004391 | SH3BGR | SH3 domain binding glutamic acid-rich protein | -1.71 | 0.057 | -2.33 | 0.006 | -2.10 | 0.013 | -2.59 | 0.003 |
| A_70_P004456 | ANTXR2 | anthrax toxin receptor 2 | -2.08 | 0.001 | -1.75 | 0.005 | -1.16 | 0.395 | -1.02 | 0.914 |
| A_70_P004466 | CKAP2 | cytoskeleton associated protein 2 | -1.14 | 0.410 | 4.60 | 0.000 | 5.26 | 0.000 | 2.85 | 0.000 |
| A_70_P004551 | GTF2H5 | general transcription factor IIH, polypeptide 5 | -1.29 | 0.230 | 1.35 | 0.164 | 2.07 | 0.004 | -1.11 | 0.622 |
| A_70_P004596 | LRG1 | leucine-rich alpha-2-glycoprotein 1 | 1.81 | 0.024 | 2.97 | 0.000 | 2.54 | 0.002 | 1.54 | 0.085 |
| A_70_P004646 | RCSD1 | RCSD domain containing 1 | -3.08 | 0.001 | -1.90 | 0.020 | -1.41 | 0.177 | 1.19 | 0.476 |
| A_70_P004806 | MAPRE3 | microtubule-associated protein, RP/EB family, member 3 | -2.51 | 0.002 | -1.67 | 0.055 | -1.53 | 0.102 | -1.70 | 0.049 |
| A_70_P004916 | MMAB | methylmalonic aciduria (cobalamin deficiency) cblB type | -2.28 | 0.000 | -1.18 | 0.320 | -1.19 | 0.299 | -1.54 | 0.018 |
| A_70_P004976 | CDKN1C | cyclin-dependent kinase inhibitor 1C (p57, Kip2) | -3.15 | 0.000 | -2.71 | 0.000 | 1.17 | 0.291 | -1.02 | 0.884 |
| A_70_P004981 | AP2A1 | adaptor-related protein complex 2, alpha 1 subunit | 2.19 | 0.212 | 1.80 | 0.343 | 3.78 | 0.045 | -1.33 | 0.641 |
| A_70_P005041 | PTTG1 | pituitary tumor-transforming 1 | -1.11 | 0.488 | 3.22 | 0.000 | 3.58 | 0.000 | 1.34 | 0.074 |
| A_70_P005076 | GPC4 | glypican 4 | -2.43 | 0.003 | -1.37 | 0.207 | -1.56 | 0.085 | -1.05 | 0.843 |
| A_70_P005081 | KIAA0020 | KIAA0020 | 1.46 | 0.229 | 2.07 | 0.032 | -1.21 | 0.536 | -1.12 | 0.710 |

| **Probenames** | **Gene Symbol** | **Gene Name** | **6h vs Baseline**  **Fold**  **Change P** | | **d1 vs Baseline**  **Fold**  **Change P** | | **d3 vs Baseline**  **Fold**  **Change P** | | **d7 vs Baseline**  **Fold**  **Change P** | |
| --- | --- | --- | --- | --- | --- | --- | --- | --- | --- | --- |
| A_70_P005141 | GNPNAT1 | glucosamine-phosphate N-acetyltransferase 1 | 1.62 | 0.020 | 2.16 | 0.001 | 1.45 | 0.064 | 1.28 | 0.200 |
| A_70_P005156 | CLPB | ClpB caseinolytic peptidase B homolog (E. coli) | 1.26 | 0.188 | 2.01 | 0.001 | 1.38 | 0.076 | 1.18 | 0.338 |
| A_70_P005186 | MOGS | mannosyl-oligosaccharide glucosidase | 1.90 | 0.008 | 2.01 | 0.005 | 1.24 | 0.302 | 1.18 | 0.428 |
| A_70_P005231 | ALDH6A1 | aldehyde dehydrogenase 6 family, member A1 | -2.06 | 0.002 | -1.59 | 0.028 | -1.33 | 0.152 | -1.20 | 0.359 |
| A_70_P005371 | FAM105A | family with sequence similarity 105, member A | -2.06 | 0.001 | -2.34 | 0.000 | -1.46 | 0.030 | 1.08 | 0.642 |
| A_70_P005501 | CDC20 | cell division cycle 20 homolog (S. cerevisiae) | -1.20 | 0.492 | 3.73 | 0.000 | 4.75 | 0.000 | 1.70 | 0.058 |
| A_70_P005511 | DIRAS3 | DIRAS family, GTP-binding RAS-like 3 | -1.29 | 0.175 | -1.64 | 0.017 | -2.75 | 0.000 | -1.20 | 0.329 |
| A_70_P005521 | RNF13 | ring finger protein 13 | -2.00 | 0.001 | -1.41 | 0.043 | -1.24 | 0.177 | -1.23 | 0.208 |
| A_70_P005526 | STMN2 | stathmin-like 2 | -1.46 | 0.358 | -4.12 | 0.004 | -3.63 | 0.007 | 1.07 | 0.860 |
| A_70_P005621 | SLC39A14 | solute carrier family 39 (zinc transporter), member 14 | 3.07 | 0.001 | 2.46 | 0.004 | 1.49 | 0.136 | 1.79 | 0.037 |
| A_70_P005636 | TFPI2 | tissue factor pathway inhibitor 2 | 2.47 | 0.003 | 1.36 | 0.227 | 1.22 | 0.420 | 1.02 | 0.935 |
| A_70_P005776 | SLC39A14 | solute carrier family 39 (zinc transporter) | 2.15 | 0.007 | 1.85 | 0.024 | 1.24 | 0.389 | 1.73 | 0.040 |
| A_70_P005851 | MYBBP1A | MYB binding protein (P160) 1a | 2.28 | 0.002 | 2.16 | 0.003 | 1.28 | 0.250 | -1.17 | 0.457 |
| A_70_P005981 | CDCA5 | cell division cycle associated 5 | -1.43 | 0.151 | 5.04 | 0.000 | 4.45 | 0.000 | 1.99 | 0.012 |
| A_70_P005986 | CYFIP2 | cytoplasmic FMR1 interacting protein 2 | -2.46 | 0.001 | -1.79 | 0.017 | -2.09 | 0.004 | -1.54 | 0.062 |
| A_70_P005991 | IL1R2 | interleukin 1 receptor, type II | 6.69 | 0.000 | 3.51 | 0.000 | 5.97 | 0.000 | 2.13 | 0.004 |
| A_70_P005996 | FGF-BP | fibroblast growth factor-binding protein | 2.99 | 0.005 | 3.33 | 0.002 | 1.77 | 0.095 | -1.76 | 0.097 |
| A_70_P006051 | PON3 | paraoxonase 3 | -2.51 | 0.006 | -2.03 | 0.025 | -3.38 | 0.001 | -3.04 | 0.002 |

| **Probenames** | **Gene Symbol** | **Gene Name** | **6h vs Baseline**  **Fold**  **Change P** | | **d1 vs Baseline**  **Fold**  **Change P** | | **d3 vs Baseline**  **Fold**  **Change P** | | **d7 vs Baseline**  **Fold**  **Change P** | |
| --- | --- | --- | --- | --- | --- | --- | --- | --- | --- | --- |
| A_70_P006071 | PLN | phospholamban | -3.83 | 0.005 | -3.39 | 0.008 | -3.46 | 0.007 | -2.74 | 0.023 |
| A_70_P006136 | FAM13A | family with sequence similarity 13, member A | -2.14 | 0.004 | -1.54 | 0.070 | -2.11 | 0.005 | -1.64 | 0.040 |
| A_70_P006211 | FBXL3 | F-box and leucine-rich repeat protein 3 | -2.44 | 0.000 | -2.05 | 0.001 | -1.68 | 0.009 | -1.38 | 0.082 |
| A_70_P006216 | LMO7 | LIM domain 7 | -2.38 | 0.000 | -1.90 | 0.004 | -2.04 | 0.002 | -1.35 | 0.125 |
| A_70_P006271 | AGXT2L2 | alanine-glyoxylate aminotransferase 2-like 2 | -2.62 | 0.000 | -1.84 | 0.007 | -1.31 | 0.176 | -1.39 | 0.108 |
| A_70_P006301 | CA8 | carbonic anhydrase VIII | -1.63 | 0.064 | -1.29 | 0.311 | -2.36 | 0.004 | -2.10 | 0.010 |
| A_70_P006326 | MEOX1 | mesenchyme homeobox 1 | 1.20 | 0.444 | 1.93 | 0.015 | 2.89 | 0.001 | 1.77 | 0.031 |
| A_70_P006401 | AMICA1 | adhesion molecule, interacts with CXADR antigen 1 | -1.18 | 0.350 | 1.29 | 0.171 | 2.17 | 0.001 | 1.75 | 0.007 |
| A_70_P006476 | FAM13C | family with sequence similarity 13, member C | -3.21 | 0.000 | -1.90 | 0.005 | -1.30 | 0.176 | 1.02 | 0.903 |
| A_70_P006536 | MGMT | O-6-methylguanine-DNA methyltransferase | -1.76 | 0.010 | -1.29 | 0.194 | -1.58 | 0.029 | -2.11 | 0.002 |
| A_70_P006606 | ZWINT | ZW10 interactor | -1.89 | 0.002 | 2.46 | 0.000 | 1.78 | 0.005 | 1.19 | 0.320 |
| A_70_P006651 | C8orf58 | chromosome 8 open reading frame 58 | -2.69 | 0.002 | -1.85 | 0.026 | -2.24 | 0.006 | -1.50 | 0.117 |
| A_70_P006751 | MFSD2A | major facilitator superfamily domain containing 2A | 3.60 | 0.000 | 4.38 | 0.000 | 3.11 | 0.000 | 1.74 | 0.039 |
| A_70_P006811 | RPS4Y1 | ribosomal protein S4, Y-linked 1 | 1.93 | 0.001 | 2.28 | 0.000 | 1.68 | 0.007 | -1.12 | 0.502 |
| A_70_P006901 | GJB2 | gap junction protein, beta 2, 26kDa | 1.60 | 0.096 | 2.45 | 0.005 | 3.61 | 0.000 | 3.05 | 0.001 |
| A_70_P006991 | COL3A1 | collagen, type III, alpha 1 | -1.39 | 0.229 | -1.32 | 0.303 | 2.76 | 0.002 | 5.85 | 0.000 |
| A_70_P007006 | ZEB2 | zinc finger E-box binding homeobox 2 | -2.49 | 0.001 | -1.84 | 0.013 | -1.34 | 0.196 | 1.17 | 0.464 |
| A_70_P007051 | EPB41L4A | erythrocyte membrane protein band 4.1 like 4A | -2.15 | 0.003 | -1.45 | 0.093 | -1.53 | 0.058 | -1.30 | 0.225 |
| A_70_P007146 | COL1A2 | collagen, type I, alpha 2 | -1.21 | 0.463 | 1.01 | 0.954 | 3.63 | 0.000 | 5.28 | 0.000 |

| **Probenames** | **Gene Symbol** | **Gene Name** | **6h vs Baseline**  **Fold**  **Change P** | | **d1 vs Baseline**  **Fold**  **Change P** | | **d3 vs Baseline**  **Fold**  **Change P** | | **d7 vs Baseline**  **Fold**  **Change P** | |
| --- | --- | --- | --- | --- | --- | --- | --- | --- | --- | --- |
| A_70_P007201 | COL12A1 | collagen, type XII, alpha 1 | -1.62 | 0.011 | -1.03 | 0.869 | 1.35 | 0.086 | 2.39 | 0.000 |
| A_70_P007211 | GFPT1 | glutamine--fructose-6-phosphate transaminase 1 | 1.98 | 0.005 | 3.28 | 0.000 | 2.68 | 0.000 | 1.58 | 0.041 |
| A_70_P007226 | MREG | melanoregulin | -1.93 | 0.005 | 1.03 | 0.895 | 2.19 | 0.002 | 2.80 | 0.000 |
| A_70_P007316 | SLC40A1 | solute carrier family 40 (iron-regulated transporter), member 1 | -3.29 | 0.000 | -2.12 | 0.010 | -1.52 | 0.111 | -1.14 | 0.592 |
| A_70_P007396 | C21orf2 | chromosome 21 open reading frame 2 | -2.01 | 0.004 | -1.53 | 0.054 | -1.56 | 0.045 | -1.33 | 0.175 |
| A_70_P007546 | TMEM140 | transmembrane protein 140 | -3.68 | 0.001 | -2.18 | 0.024 | -1.42 | 0.271 | 1.21 | 0.535 |
| A_70_P007551 | CENPM | centromere protein M | -1.24 | 0.094 | 1.93 | 0.000 | 2.11 | 0.000 | 1.61 | 0.002 |
| A_70_P007556 | SUMF1 | sulfatase modifying factor 1 | -1.56 | 0.057 | -1.43 | 0.114 | -2.14 | 0.004 | -1.53 | 0.066 |
| A_70_P007586 | IFT80 | intraflagellar transport 80 homolog (Chlamydomonas) | -2.26 | 0.019 | -1.46 | 0.232 | -1.71 | 0.103 | -1.16 | 0.628 |
| A_70_P007666 | KIF2C | kinesin family member 2C (KIF2C) | -1.45 | 0.034 | 5.15 | 0.000 | 5.63 | 0.000 | 2.17 | 0.000 |
| A_70_P007736 | LRRC33 | leucine rich repeat containing 33 | -2.03 | 0.028 | -1.42 | 0.242 | -1.22 | 0.496 | 1.48 | 0.191 |
| A_70_P007906 | BEND5 | BEN domain containing 5 | -2.42 | 0.000 | -2.01 | 0.001 | -2.23 | 0.000 | -1.62 | 0.009 |
| A_70_P007991 | BCL2L11 | BCL2-like 11 (apoptosis facilitator) | 1.65 | 0.018 | 2.21 | 0.001 | 3.38 | 0.000 | 1.75 | 0.010 |
| A_70_P008031 | KPNA2 | karyopherin alpha 2 (RAG cohort 1, importin alpha 1) | 1.20 | 0.346 | 2.12 | 0.001 | 1.94 | 0.003 | 1.48 | 0.053 |
| A_70_P008066 | POR | P450 (cytochrome) oxidoreductase | -2.13 | 0.035 | -1.42 | 0.291 | -1.59 | 0.169 | -1.27 | 0.474 |
| A_70_P008081 | JUN | jun proto-oncogene | -1.59 | 0.073 | -1.58 | 0.076 | -2.68 | 0.001 | -2.34 | 0.004 |
| A_70_P008096 | COL12A1 | collagen, type XII, alpha 1 | -1.64 | 0.036 | -1.04 | 0.869 | 1.23 | 0.344 | 3.07 | 0.000 |
| A_70_P008196 | IL1RN | interleukin 1 receptor antagonist | 2.24 | 0.065 | 1.67 | 0.222 | 3.34 | 0.010 | 3.61 | 0.007 |
| A_70_P008211 | CLEC6A | C-type lectin domain family 6, member A | 1.16 | 0.517 | 1.53 | 0.074 | 2.39 | 0.002 | 2.60 | 0.001 |

| **Probenames** | **Gene Symbol** | **Gene Name** | **6h vs Baseline**  **Fold**  **Change P** | | **d1 vs Baseline**  **Fold**  **Change P** | | **d3 vs Baseline**  **Fold**  **Change P** | | **d7 vs Baseline**  **Fold**  **Change P** | |
| --- | --- | --- | --- | --- | --- | --- | --- | --- | --- | --- |
| A_70_P008212 | CLEC6A | C-type lectin domain family 6, member A | 1.06 | 0.773 | 1.40 | 0.123 | 2.18 | 0.002 | 2.38 | 0.001 |
| A_70_P008251 | EGLN3 | egl nine homolog 3 (C. elegans) | 1.97 | 0.007 | 2.66 | 0.001 | 2.69 | 0.000 | 2.35 | 0.001 |
| A_70_P008306 | HYOU1 | hypoxia up-regulated 1 | 2.38 | 0.003 | 2.16 | 0.006 | 1.18 | 0.490 | 1.26 | 0.332 |
| A_70_P008336 | SNAP25 | synaptosomal-associated protein, 25kDa | -1.20 | 0.516 | -1.23 | 0.465 | -1.19 | 0.529 | 2.01 | 0.024 |
| A_70_P008371 | NDRG4 | NDRG family member 4 | -1.86 | 0.020 | -1.99 | 0.011 | -2.43 | 0.002 | -2.26 | 0.004 |
| A_70_P008486 | RTN1 | reticulon 1 | -2.27 | 0.010 | -2.43 | 0.006 | -4.23 | 0.000 | -1.39 | 0.239 |
| A_70_P008511 | CHRDL1 | chordin-like 1 | -1.12 | 0.682 | -2.48 | 0.007 | -1.48 | 0.189 | -1.14 | 0.639 |
| A_70_P008596 | TTK | TTK protein kinase | -1.41 | 0.035 | 6.29 | 0.000 | 4.66 | 0.000 | 2.44 | 0.000 |
| A_70_P008661 | ZWILCH | Zwilch, kinetochore associated, homolog (Drosophila) | -1.15 | 0.417 | 2.04 | 0.001 | 1.50 | 0.029 | 1.13 | 0.462 |
| A_70_P008671 | SLCO3A1 | solute carrier organic anion transporter family, member 3A1 | -2.09 | 0.000 | -1.46 | 0.023 | -1.39 | 0.040 | -1.23 | 0.179 |
| A_70_P008776 | SLCO4A1 | solute carrier organic anion transporter family member 4A1 | 6.23 | 0.000 | 4.49 | 0.000 | 3.10 | 0.003 | 1.82 | 0.078 |
| A_70_P008871 | POU2AF1 | POU class 2 associating factor 1 | -1.73 | 0.050 | 1.52 | 0.124 | 2.56 | 0.003 | 1.44 | 0.176 |
| A_70_P009046 | VRK1 | vaccinia related kinase 1 | -1.64 | 0.004 | 2.11 | 0.000 | 1.79 | 0.001 | 1.13 | 0.396 |
| A_70_P009296 | ERG | v-ets erythroblastosis virus E26 oncogene homolog (avian) | -2.04 | 0.005 | -1.48 | 0.087 | -1.06 | 0.783 | 1.30 | 0.234 |
| A_70_P009411 | PSPH | phosphoserine phosphatase | 1.44 | 0.025 | 2.34 | 0.000 | 1.65 | 0.004 | 1.39 | 0.039 |
| A_70_P009416 | SKA1 | spindle and kinetochore associated complex subunit 1 | -1.59 | 0.014 | 3.49 | 0.000 | 3.61 | 0.000 | 1.37 | 0.072 |
| A_70_P009466 | PSAT1 | phosphoserine aminotransferase 1 | 1.94 | 0.050 | 2.46 | 0.012 | 1.30 | 0.404 | 1.23 | 0.515 |
| A_70_P009551 | NCAPG | non-SMC condensin I complex, subunit G | -1.43 | 0.042 | 7.12 | 0.000 | 7.58 | 0.000 | 3.04 | 0.000 |

| **Probenames** | **Gene Symbol** | **Gene Name** | **6h vs Baseline**  **Fold**  **Change P** | | **d1 vs Baseline**  **Fold**  **Change P** | | **d3 vs Baseline**  **Fold**  **Change P** | | **d7 vs Baseline**  **Fold**  **Change P** | |
| --- | --- | --- | --- | --- | --- | --- | --- | --- | --- | --- |
| A_70_P009666 | MT-2 | metallothionein 2A | 13.99 | 0.000 | 14.72 | 0.000 | 11.74 | 0.000 | 2.56 | 0.020 |
| A_70_P009856 | CACHD1 | cache domain containing 1 | -2.40 | 0.003 | -1.77 | 0.034 | -2.12 | 0.008 | -1.56 | 0.088 |
| A_70_P009981 | MRTO4 | mRNA turnover 4 homolog (S. cerevisiae) | 1.90 | 0.004 | 2.11 | 0.001 | 1.43 | 0.070 | -1.02 | 0.913 |
| A_70_P009991 | MYH10 | myosin, heavy chain 10, non-muscle | -1.67 | 0.036 | -2.01 | 0.007 | -2.18 | 0.004 | -1.13 | 0.577 |
| A_70_P009996 | DDX39A | DEAD (Asp-Glu-Ala-Asp) box polypeptide 39A | 1.69 | 0.014 | 2.17 | 0.001 | 1.65 | 0.018 | 1.06 | 0.744 |
| A_70_P010021 | RHBDL1 | rhomboid, veinlet-like 1 (Drosophila) | -2.02 | 0.002 | -1.79 | 0.006 | -1.08 | 0.661 | -1.13 | 0.508 |
| A_70_P010041 | GRAP | GRB2-related adaptor protein | -2.62 | 0.001 | -1.69 | 0.033 | -1.20 | 0.414 | 1.19 | 0.440 |
| A_70_P010056 | RNASEH2A | ribonuclease H2, subunit A | -1.71 | 0.010 | 1.61 | 0.020 | 2.08 | 0.001 | 1.00 | 0.990 |
| A_70_P010106 | AIF1 | allograft inflammatory factor 1 | -2.53 | 0.007 | 1.00 | 0.994 | 1.19 | 0.558 | -1.01 | 0.973 |
| A_70_P010156 | RAPGEF3 | Rap guanine nucleotide exchange factor (GEF) 3 | -2.14 | 0.005 | -1.41 | 0.145 | -1.20 | 0.431 | -1.12 | 0.612 |
| A_70_P010196 | CERCAM | cerebral endothelial cell adhesion molecule | -1.10 | 0.733 | -1.02 | 0.934 | 2.29 | 0.008 | 2.08 | 0.016 |
| A_70_P010201 | S100A9 | S100 calcium binding protein A9 | 2.74 | 0.000 | 3.04 | 0.000 | 3.34 | 0.000 | 1.32 | 0.174 |
| A_70_P010216 | CFP | complement factor properdin | -1.03 | 0.930 | 2.22 | 0.023 | 1.17 | 0.618 | 1.12 | 0.729 |
| A_70_P010226 | PLTP | phospholipid transfer protein | -2.01 | 0.020 | -1.44 | 0.189 | 1.55 | 0.119 | 1.48 | 0.160 |
| A_70_P010236 | PDGFRL | platelet-derived growth factor receptor-like | 1.48 | 0.289 | 1.43 | 0.338 | 3.25 | 0.006 | 2.04 | 0.069 |
| A_70_P010241 | MANF | mesencephalic astrocyte-derived neurotrophic factor | 3.07 | 0.000 | 3.15 | 0.000 | 2.27 | 0.001 | 1.26 | 0.274 |
| A_70_P010321 | TACC3 | transforming, acidic coiled-coil containing protein 3 | 1.24 | 0.380 | 1.04 | 0.872 | 2.11 | 0.009 | 1.53 | 0.100 |
| A_70_P010336 | SCPEP1 | serine carboxypeptidase 1 | -2.00 | 0.002 | -1.41 | 0.079 | 1.18 | 0.379 | 1.56 | 0.028 |
| A_70_P010436 | LOC786490 | similar to epithelial membrane protein 1 | 2.27 | 0.005 | 1.51 | 0.109 | 1.20 | 0.448 | 1.44 | 0.154 |

| **Probenames** | **Gene Symbol** | **Gene Name** | **6h vs Baseline**  **Fold**  **Change P** | | **d1 vs Baseline**  **Fold**  **Change P** | | **d3 vs Baseline**  **Fold**  **Change P** | | **d7 vs Baseline**  **Fold**  **Change P** | |
| --- | --- | --- | --- | --- | --- | --- | --- | --- | --- | --- |
| A_70_P010476 | PDGFRB | platelet-derived growth factor receptor, beta polypeptide | -2.72 | 0.001 | -1.60 | 0.060 | 1.03 | 0.911 | 1.83 | 0.020 |
| A_70_P010501 | TROAP | trophinin associated protein (tastin) | 1.03 | 0.848 | 1.73 | 0.003 | 2.00 | 0.001 | 1.15 | 0.370 |
| A_70_P010556 | NTRK2 | neurotrophic tyrosine kinase, receptor, type 2 | -1.84 | 0.027 | -2.18 | 0.007 | -2.00 | 0.014 | -2.25 | 0.006 |
| A_70_P010561 | F13A1 | coagulation factor XIII, A1 polypeptide | 1.86 | 0.042 | 2.05 | 0.022 | 2.12 | 0.017 | 2.06 | 0.021 |
| A_70_P010646 | FBXO32 | F-box protein 32 | -2.36 | 0.006 | -3.40 | 0.000 | -1.91 | 0.028 | -1.22 | 0.454 |
| A_70_P010666 | ZNF613 | zinc finger protein 613 | -2.04 | 0.007 | -1.43 | 0.126 | -1.54 | 0.069 | -1.15 | 0.536 |
| A_70_P010681 | SLMAP | sarcolemma associated protein | -2.07 | 0.019 | -2.27 | 0.010 | -3.06 | 0.001 | -2.15 | 0.014 |
| A_70_P010806 | CDCA7 | cell division cycle associated 7 | -1.30 | 0.300 | 2.77 | 0.001 | 1.71 | 0.048 | 1.20 | 0.480 |
| A_70_P010821 | SVIL | supervillin | -2.37 | 0.002 | -2.01 | 0.007 | -2.07 | 0.005 | -1.72 | 0.027 |
| A_70_P010836 | PTX3 | pentraxin 3, long | 10.80 | 0.000 | 6.93 | 0.000 | 2.75 | 0.024 | 1.85 | 0.141 |
| A_70_P010837 | PTX3 | pentraxin 3, long | 10.92 | 0.000 | 7.09 | 0.000 | 2.67 | 0.031 | 1.80 | 0.168 |
| A_70_P010906 | CDH3 | cadherin 3, type 1, P-cadherin (placental) | -1.18 | 0.342 | 1.56 | 0.020 | 2.00 | 0.001 | 1.42 | 0.059 |
| A_70_P010976 | SDR16C5 | short chain dehydrogenase/reductase family 16C, member 5 | -2.39 | 0.021 | -1.65 | 0.155 | -1.11 | 0.758 | -1.38 | 0.353 |
| A_70_P011056 | TCF19 | transcription factor 19 | -1.54 | 0.017 | 4.16 | 0.000 | 2.93 | 0.000 | 1.69 | 0.006 |
| A_70_P011071 | KIAA1370 | KIAA1370 | -3.24 | 0.000 | -2.13 | 0.005 | -1.95 | 0.010 | -1.41 | 0.144 |
| A_70_P011076 | CLEC14A | C-type lectin domain family 14, member A | -3.88 | 0.000 | -2.45 | 0.007 | -1.75 | 0.065 | 1.04 | 0.892 |
| A_70_P011116 | NFIL3 | nuclear factor, interleukin 3 regulated | 3.32 | 0.000 | 2.58 | 0.000 | 1.89 | 0.008 | 1.33 | 0.173 |
| A_70_P011146 | AEBP1 | AE binding protein 1 | 1.56 | 0.052 | 2.14 | 0.003 | 2.97 | 0.000 | 1.92 | 0.008 |
| A_70_P011216 | EPHX1 | epoxide hydrolase 1, microsomal (xenobiotic) | -2.37 | 0.002 | -1.87 | 0.016 | -2.46 | 0.002 | -1.79 | 0.022 |
| A_70_P011266 | C1QTNF5 | C1q and tumor necrosis factor related protein 5 | 1.74 | 0.022 | 2.18 | 0.003 | 2.77 | 0.000 | 2.05 | 0.005 |

| **Probenames** | **Gene Symbol** | **Gene Name** | **6h vs Baseline**  **Fold**  **Change P** | | **d1 vs Baseline**  **Fold**  **Change P** | | **d3 vs Baseline**  **Fold**  **Change P** | | **d7 vs Baseline**  **Fold**  **Change P** | |
| --- | --- | --- | --- | --- | --- | --- | --- | --- | --- | --- |
| A_70_P011346 | STAB1 | stabilin 1 | -1.55 | 0.051 | 1.93 | 0.007 | 3.32 | 0.000 | 2.05 | 0.004 |
| A_70_P011476 | BMP1 | bone morphogenetic protein 1 | -1.31 | 0.161 | 1.01 | 0.962 | 1.95 | 0.003 | 2.66 | 0.000 |
| A_70_P011536 | FN1 | fibronectin 1 | 1.24 | 0.341 | 1.21 | 0.397 | 1.60 | 0.055 | 2.33 | 0.002 |
| A_70_P011546 | MCM4 | minichromosome maintenance complex component 4 | -1.25 | 0.133 | 2.70 | 0.000 | 1.63 | 0.004 | 1.29 | 0.092 |
| A_70_P011556 | GAS6 | growth arrest-specific 6 | -2.01 | 0.002 | -1.34 | 0.120 | -1.61 | 0.019 | -1.29 | 0.174 |
| A_70_P011561 | ANGPTL2 | angiopoietin-like 2 | -2.39 | 0.002 | -1.87 | 0.012 | 1.12 | 0.602 | 2.68 | 0.001 |
| A_70_P011601 | IFRD2 | interferon-related developmental regulator 2 | 2.72 | 0.001 | 2.58 | 0.002 | 1.53 | 0.100 | -1.05 | 0.843 |
| A_70_P011611 | CYB5B | cytochrome b5 type B (outer mitochondrial membrane) | 1.02 | 0.909 | 2.07 | 0.001 | 1.96 | 0.002 | 1.02 | 0.907 |
| A_70_P011691 | ADAMTS2 | ADAM metallopeptidase with thrombospondin type 1 motif, 2 | 1.10 | 0.667 | 1.13 | 0.585 | 2.81 | 0.001 | 3.74 | 0.000 |
| A_70_P011771 | LOC617255 | similar to Nucleolar protein 5A (56kDa with KKE/D repeat) | 2.00 | 0.002 | 2.18 | 0.001 | 1.24 | 0.243 | -1.03 | 0.885 |
| A_70_P011891 | MND1 | meiotic nuclear divisions 1 homolog (S. cerevisiae) | -1.48 | 0.041 | 4.26 | 0.000 | 5.72 | 0.000 | 1.79 | 0.005 |
| A_70_P012151 | LHX3 | LIM homeobox 3 | -3.48 | 0.001 | -2.25 | 0.018 | -3.69 | 0.001 | -2.03 | 0.033 |
| A_70_P012261 | GJB3 | gap junction protein, beta 3, 31kDa | 3.60 | 0.001 | 3.66 | 0.000 | 4.87 | 0.000 | 2.71 | 0.003 |
| A_70_P012341 | TP53BP2 | tumor protein p53 binding protein, 2 | -1.73 | 0.013 | -1.50 | 0.050 | -2.16 | 0.001 | -1.44 | 0.075 |
| A_70_P012356 | C6orf105 | chromosome 6 open reading frame 105 | 1.56 | 0.076 | 2.33 | 0.003 | 1.46 | 0.129 | 1.33 | 0.243 |
| A_70_P012436 | CXCL5 | chemokine (C-X-C motif) ligand 5 | 2.66 | 0.000 | 2.99 | 0.000 | 3.00 | 0.000 | 1.80 | 0.005 |
| A_70_P012536 | LGALS15 | galectin 15 | 2.77 | 0.003 | 1.14 | 0.652 | 1.36 | 0.294 | 1.20 | 0.528 |

| **Probenames** | **Gene Symbol** | **Gene Name** | **6h vs Baseline**  **Fold**  **Change P** | | **d1 vs Baseline**  **Fold**  **Change P** | | **d3 vs Baseline**  **Fold**  **Change P** | | **d7 vs Baseline**  **Fold**  **Change P** | |
| --- | --- | --- | --- | --- | --- | --- | --- | --- | --- | --- |
| A_70_P012537 | LGALS15 | galectin 15 | 2.38 | 0.004 | 1.04 | 0.868 | 1.27 | 0.355 | 1.11 | 0.681 |
| A_70_P012566 | BEGAIN | brain-enriched guanylate kinase-associated homolog (rat) | -2.07 | 0.001 | -1.68 | 0.011 | -1.68 | 0.011 | -1.86 | 0.004 |
| A_70_P012567 | BEGAIN | brain-enriched guanylate kinase-associated homolog (rat) | -2.11 | 0.001 | -1.63 | 0.012 | -1.62 | 0.013 | -1.88 | 0.002 |
| A_70_P012581 | SPA17 | sperm autoantigenic protein 17 | -2.33 | 0.017 | -1.60 | 0.148 | -1.69 | 0.109 | -2.08 | 0.033 |
| A_70_P012586 | EDNRA | endothelin receptor type A | -2.61 | 0.001 | -1.96 | 0.012 | -2.41 | 0.002 | -1.77 | 0.027 |
| A_70_P012596 | PTGS1 | prostaglandin-endoperoxide synthase 1 (prostaglandin G/H synthas | -3.61 | 0.000 | -2.16 | 0.015 | -2.51 | 0.005 | -1.59 | 0.114 |
| A_70_P012711 | FADS2 | fatty acid desaturase 2 | -2.07 | 0.002 | -1.02 | 0.906 | -1.12 | 0.550 | 1.06 | 0.769 |
| A_70_P012881 | KIAA0101 | KIAA0101 protein | -2.44 | 0.000 | 7.78 | 0.000 | 6.33 | 0.000 | 2.48 | 0.000 |
| A_70_P013006 | CXCL14 | chemokine (C-X-C motif) ligand 14 | -1.91 | 0.054 | -2.26 | 0.019 | 1.03 | 0.917 | -1.52 | 0.191 |
| A_70_P013071 | LSM14A | LSM14A, SCD6 homolog A (S. cerevisiae) | 1.26 | 0.547 | 1.54 | 0.269 | 2.89 | 0.015 | -1.02 | 0.952 |
| A_70_P013091 | RBM38 | RNA binding motif protein 38 | -1.69 | 0.027 | -1.46 | 0.092 | -1.72 | 0.022 | -2.02 | 0.005 |
| A_70_P013261 | LAMA4 | laminin, alpha 4 | -2.11 | 0.007 | -1.78 | 0.026 | -1.41 | 0.157 | 1.11 | 0.648 |
| A_70_P013266 | ELN | elastin | -1.93 | 0.006 | -2.21 | 0.002 | -1.47 | 0.075 | -1.33 | 0.172 |
| A_70_P013366 | ABHD11 | abhydrolase domain containing 11 | 1.57 | 0.031 | 2.26 | 0.001 | 1.81 | 0.007 | 1.00 | 0.995 |
| A_70_P013386 | LOX | lysyl oxidase | -1.07 | 0.814 | 1.26 | 0.414 | 1.87 | 0.038 | 2.44 | 0.006 |
| A_70_P013421 | SLC7A7 | solute carrier family 7 (cationic amino acid transporter, y+ sys | -2.50 | 0.002 | -1.80 | 0.027 | 1.05 | 0.842 | 1.69 | 0.046 |
| A_70_P013461 | GNG11 | guanine nucleotide binding protein (G protein), gamma 11" | -2.31 | 0.002 | -2.04 | 0.005 | -1.26 | 0.292 | -1.82 | 0.014 |
| A_70_P013471 | LMNA | lamin A/C | 1.83 | 0.008 | 2.34 | 0.001 | 2.23 | 0.001 | 1.67 | 0.020 |

| **Probenames** | **Gene Symbol** | **Gene Name** | **6h vs Baseline**  **Fold**  **Change P** | | **d1 vs Baseline**  **Fold**  **Change P** | | **d3 vs Baseline**  **Fold**  **Change P** | | **d7 vs Baseline**  **Fold**  **Change P** | |
| --- | --- | --- | --- | --- | --- | --- | --- | --- | --- | --- |
| A_70_P013486 | CLDN5 | claudin 5 | -4.04 | 0.001 | -3.13 | 0.004 | -2.16 | 0.035 | -1.37 | 0.353 |
| A_70_P013551 | RUSC1 | RUN and SH3 domain containing 1 | 1.01 | 0.977 | 2.00 | 0.002 | 1.32 | 0.150 | -1.25 | 0.234 |
| A_70_P013571 | CNP | 2',3'-cyclic nucleotide 3' phosphodiesterase | 2.15 | 0.000 | 1.96 | 0.001 | 1.52 | 0.016 | 1.10 | 0.539 |
| A_70_P013591 | PBXIP1 | pre-B-cell leukemia homeobox interacting protein 1 | -2.07 | 0.011 | -1.88 | 0.024 | -1.60 | 0.077 | -1.18 | 0.513 |
| A_70_P013681 | FCGR1A | Fc fragment of IgG, high affinity Ia, receptor (CD64)" | 1.07 | 0.820 | 1.50 | 0.199 | 5.68 | 0.000 | 3.87 | 0.001 |
| A_70_P013696 | NM23A | non-metastatic cells 1 | 1.44 | 0.056 | 2.16 | 0.001 | 1.72 | 0.008 | -1.14 | 0.447 |
| A_70_P013726 | RNF13 | ring finger protein 13 | -2.26 | 0.003 | -1.54 | 0.076 | -1.63 | 0.049 | -1.18 | 0.467 |
| A_70_P013731 | NSMCE1 | non-SMC element 1 homolog (S. cerevisiae) | -2.05 | 0.000 | -1.43 | 0.028 | -1.32 | 0.080 | -1.71 | 0.003 |
| A_70_P013771 | ACVRL1 | activin A receptor type II-like 1 | -2.23 | 0.017 | -1.73 | 0.083 | -1.51 | 0.182 | 1.05 | 0.867 |
| A_70_P013886 | KIAA1804 | mixed lineage kinase 4 (KIAA1804) | -2.23 | 0.000 | -1.70 | 0.008 | -1.72 | 0.007 | -1.52 | 0.027 |
| A_70_P013936 | MCM10 | minichromosome maintenance complex component 10 | -1.29 | 0.157 | 3.53 | 0.000 | 2.40 | 0.000 | 1.50 | 0.031 |
| A_70_P013986 | SLC46A3 | solute carrier family 46, member 3 | -2.56 | 0.000 | -1.61 | 0.019 | -1.36 | 0.104 | -1.21 | 0.294 |
| A_70_P014156 | UHRF1 | ubiquitin-like with PHD and ring finger domains 1 | -1.18 | 0.235 | 6.25 | 0.000 | 3.49 | 0.000 | 1.73 | 0.001 |
| A_70_P014246 | SYNE1 | spectrin repeat containing, nuclear envelope 1 | -2.59 | 0.001 | -2.05 | 0.007 | -2.57 | 0.001 | -1.71 | 0.033 |
| A_70_P014421 | MTHFD1L | methylenetetrahydrofolate dehydrogenase (NADP+dependent) | 1.74 | 0.006 | 2.13 | 0.001 | 2.02 | 0.001 | 1.83 | 0.004 |
| A_70_P014566 | C13orf27 | chromosome 13 open reading frame 27 | -1.30 | 0.316 | 1.46 | 0.156 | 2.24 | 0.007 | 1.04 | 0.868 |

| **Probenames** | **Gene Symbol** | **Gene Name** | **6h vs Baseline**  **Fold**  **Change P** | | **d1 vs Baseline**  **Fold**  **Change P** | | **d3 vs Baseline**  **Fold**  **Change P** | | **d7 vs Baseline**  **Fold**  **Change P** | |
| --- | --- | --- | --- | --- | --- | --- | --- | --- | --- | --- |
| A_70_P014576 | TPX2 | TPX2, microtubule-associated, homolog (Xenopus laevis) | -1.33 | 0.097 | 2.97 | 0.000 | 2.87 | 0.000 | 2.02 | 0.001 |
| A_70_P014586 | IQCB1 | IQ motif containing B1 | -1.50 | 0.130 | -1.45 | 0.157 | -2.01 | 0.016 | -1.23 | 0.421 |
| A_70_P014636 | AC011498.2 | NULL | -2.42 | 0.000 | -1.92 | 0.004 | -2.14 | 0.001 | -1.71 | 0.013 |
| A_70_P014637 | AC011498.2 | NULL | -2.58 | 0.000 | -2.03 | 0.003 | -2.24 | 0.001 | -1.75 | 0.013 |
| A_70_P014652 | EPAS1 | endothelial PAS domain protein 1 | -2.10 | 0.003 | -1.79 | 0.012 | -1.63 | 0.030 | -1.38 | 0.132 |
| A_70_P014656 | PPP1R12A | protein phosphatase 1, regulatory (inhibitor) subunit 12A | -1.70 | 0.021 | -1.71 | 0.020 | -2.17 | 0.002 | -1.69 | 0.023 |
| A_70_P014657 | PPP1R12A | protein phosphatase 1, regulatory (inhibitor) subunit 12A | -1.74 | 0.020 | -1.69 | 0.026 | -2.07 | 0.004 | -1.71 | 0.024 |
| A_70_P014786 | SCG3 | secretogranin III | -1.85 | 0.001 | -2.01 | 0.000 | -2.22 | 0.000 | -1.80 | 0.001 |
| A_70_P014846 | RUNDC3B | RUN domain containing 3B | -3.33 | 0.000 | -2.05 | 0.005 | -1.55 | 0.060 | -1.79 | 0.018 |
| A_70_P014861 | SLC1A3 | solute carrier family 1 (glial high affinity glutamate transport | 1.25 | 0.284 | 2.23 | 0.002 | 1.95 | 0.006 | 1.49 | 0.071 |
| A_70_P014901 | IGLL1 | immunoglobulin lambda-like polypeptide 1 | -1.12 | 0.757 | 1.20 | 0.617 | 2.07 | 0.062 | 2.37 | 0.031 |
| A_70_P015051 | PCMTD1 | protein-L-isoaspartate (D-aspartate) O-methyltransferase domain | -2.24 | 0.000 | -1.73 | 0.006 | -1.55 | 0.020 | -1.41 | 0.058 |
| A_70_P015121 | ABI2 | abl-interactor 2 | -2.20 | 0.001 | -1.63 | 0.022 | -1.76 | 0.011 | -1.31 | 0.175 |
| A_70_P015461 | FAM54A | family with sequence similarity 54, member A | -1.16 | 0.324 | 2.89 | 0.000 | 2.47 | 0.000 | 1.44 | 0.028 |
| A_70_P015466 | EIF2C4 | eukaryotic translation initiation factor 2C, 4 | -2.76 | 0.000 | -1.94 | 0.002 | -1.57 | 0.018 | -1.34 | 0.096 |
| A_70_P015751 | DIAPH3 | diaphanous homolog 3 (Drosophila) | 1.25 | 0.215 | 5.73 | 0.000 | 5.78 | 0.000 | 2.87 | 0.000 |
| A_70_P015971 | MARCKSL1 | MARCKS-like 1 | 1.23 | 0.222 | 1.32 | 0.105 | 2.45 | 0.000 | 1.87 | 0.002 |
| A_70_P016081 | EIF5A | eukaryotic translation initiation factor 5A | 1.41 | 0.112 | 2.03 | 0.004 | 1.99 | 0.005 | 1.10 | 0.644 |

| **Probenames** | **Gene Symbol** | **Gene Name** | **6h vs Baseline**  **Fold**  **Change P** | | **d1 vs Baseline**  **Fold**  **Change P** | | **d3 vs Baseline**  **Fold**  **Change P** | | **d7 vs Baseline**  **Fold**  **Change P** | |
| --- | --- | --- | --- | --- | --- | --- | --- | --- | --- | --- |
| A_70_P016091 | EIF5A | eukaryotic translation initiation factor 5A | 1.45 | 0.167 | 2.34 | 0.005 | 2.19 | 0.009 | 1.22 | 0.447 |
| A_70_P016181 | TXNIP | thioredoxin interacting protein | -3.05 | 0.002 | -2.64 | 0.006 | -3.49 | 0.001 | -1.71 | 0.089 |
| A_70_P016306 | COL3A1 | collagen, type III, alpha 1 | -1.00 | 0.985 | 1.31 | 0.073 | 2.16 | 0.000 | 1.81 | 0.001 |
| A_70_P016311 | JUN | jun proto-oncogene | -1.34 | 0.319 | -1.85 | 0.048 | -2.33 | 0.010 | -2.11 | 0.021 |
| A_70_P016331 | MMP7 | matrix metallopeptidase 7 | 5.23 | 0.001 | 8.37 | 0.000 | 7.49 | 0.000 | 5.74 | 0.000 |
| A_70_P016332 | MMP7 | matrix metallopeptidase 7 (matrilysin, uterine) | 5.85 | 0.000 | 8.72 | 0.000 | 7.86 | 0.000 | 5.72 | 0.000 |
| A_70_P016336 | HMMR | hyaluronan-mediated motility receptor (RHAMM) | -1.62 | 0.001 | 3.69 | 0.000 | 3.96 | 0.000 | 2.12 | 0.000 |
| A_70_P016337 | HMMR | hyaluronan-mediated motility receptor (RHAMM) | -1.39 | 0.012 | 3.49 | 0.000 | 3.53 | 0.000 | 1.99 | 0.000 |
| A_70_P016351 | GSTA1-1 | microsomal glutathione-S-transferase 1-1 | -7.46 | 0.004 | -3.94 | 0.031 | -7.61 | 0.004 | -3.12 | 0.065 |
| A_70_P016352 | GSTA1-1 | microsomal glutathione-S-transferase 1-1 | -7.23 | 0.003 | -3.90 | 0.028 | -8.31 | 0.002 | -2.97 | 0.068 |
| A_70_P016371 | CONNEXIN 43 | CONNEXIN 43 protein | -1.13 | 0.525 | -1.16 | 0.449 | 1.12 | 0.570 | 2.11 | 0.002 |
| A_70_P016372 | CONNEXIN 43 | CONNEXIN 43 protein | -1.12 | 0.574 | -1.20 | 0.377 | 1.05 | 0.808 | 2.08 | 0.004 |
| A_70_P016376 | COL1A1 | collagen, type I, alpha 1 | 1.08 | 0.715 | 1.15 | 0.522 | 2.18 | 0.003 | 1.97 | 0.008 |
| A_70_P016386 | LDHA | lactate dehydrogenase A | 2.41 | 0.001 | 2.38 | 0.001 | 2.10 | 0.002 | 1.36 | 0.140 |
| A_70_P016387 | LDHA | lactate dehydrogenase A | 2.62 | 0.001 | 2.72 | 0.000 | 2.47 | 0.001 | 1.42 | 0.117 |
| A_70_P016396 | LOC443070 | ribosomal protein L32 | -2.93 | 0.004 | -2.12 | 0.027 | -2.41 | 0.012 | -1.42 | 0.262 |
| A_70_P016471 | NDRG1 | N-myc downstream regulated 1 | 1.66 | 0.116 | 1.47 | 0.225 | 2.64 | 0.007 | 1.92 | 0.050 |
| A_70_P016516 | CAV1 | caveolin 1, caveolae protein, 22kDa | -3.09 | 0.003 | -1.68 | 0.107 | -1.40 | 0.276 | 1.32 | 0.367 |

| **Probenames** | **Gene Symbol** | **Gene Name** | **6h vs Baseline**  **Fold**  **Change P** | | **d1 vs Baseline**  **Fold**  **Change P** | | **d3 vs Baseline**  **Fold**  **Change P** | | **d7 vs Baseline**  **Fold**  **Change P** | |
| --- | --- | --- | --- | --- | --- | --- | --- | --- | --- | --- |
| A_70_P016521 | ATP6V1B2 | ATPase, H+ transporting, lysosomal 56/58kDa, V1 subunit B2 | -1.44 | 0.126 | -1.39 | 0.166 | -2.78 | 0.001 | -1.83 | 0.018 |
| A_70_P016522 | ATP6V1B2 | ATPase, H+ transporting, lysosomal 56/58kDa, V1 subunit B2 | -1.55 | 0.071 | -1.44 | 0.126 | -2.73 | 0.001 | -1.87 | 0.015 |
| A_70_P016526 | HSPA5 | heat shock 70kDa protein 5 (glucose-regulated protein, 78kDa) | 2.69 | 0.001 | 2.51 | 0.001 | 1.43 | 0.135 | 1.27 | 0.304 |
| A_70_P016527 | HSPA5 | heat shock 70kDa protein 5 (glucose-regulated protein, 78kDa) | 2.36 | 0.002 | 2.35 | 0.002 | 1.32 | 0.232 | 1.24 | 0.340 |
| A_70_P016576 | LOC100037679 | KDEL receptor 2 | 1.50 | 0.028 | 2.04 | 0.001 | 1.82 | 0.003 | 1.28 | 0.158 |
| A_70_P016651 | MYOC | myocilin, trabecular meshwork inducible glucocorticoid response | -1.11 | 0.771 | -2.35 | 0.034 | -1.18 | 0.648 | -1.04 | 0.923 |
| A_70_P016656 | GSTA1 | glutathione S-transferase alpha 1 | -2.85 | 0.031 | -2.11 | 0.108 | 1.26 | 0.598 | -2.08 | 0.114 |
| A_70_P016657 | GSTA1 | glutathione S-transferase alpha 1 | -2.58 | 0.029 | -2.11 | 0.075 | 1.25 | 0.578 | -2.01 | 0.093 |
| A_70_P016711 | LOC100037688 | kappa light chain | -1.50 | 0.202 | 1.27 | 0.449 | 2.86 | 0.004 | 2.00 | 0.041 |
| A_70_P016731 | MBP | myelin basic protein | -2.04 | 0.006 | -1.82 | 0.016 | -2.44 | 0.001 | -1.66 | 0.035 |
| A_70_P016771 | CYP21A2 | cytochrome P450, family 21, subfamily A, polypeptide 2 | 2.56 | 0.021 | 2.29 | 0.037 | 3.20 | 0.006 | -1.24 | 0.545 |
| A_70_P016781 | ALOX5AP | arachidonate 5-lipoxygenase-activating protein | -2.34 | 0.002 | 1.00 | 0.997 | -1.10 | 0.661 | -1.42 | 0.136 |
| A_70_P016782 | ALOX5AP | arachidonate 5-lipoxygenase-activating protein | -2.53 | 0.000 | -1.04 | 0.847 | -1.12 | 0.561 | -1.36 | 0.135 |
| A_70_P016808 | LOC443322 | lysozyme 3a precursor | 2.29 | 0.049 | -1.50 | 0.305 | -1.24 | 0.586 | -3.54 | 0.006 |
| A_70_P016809 | LOC443322 | lysozyme 3a precursor | 2.33 | 0.044 | -1.52 | 0.287 | -1.25 | 0.561 | -3.59 | 0.005 |

| **Probenames** | **Gene Symbol** | **Gene Name** | **6h vs Baseline**  **Fold**  **Change P** | | **d1 vs Baseline**  **Fold**  **Change P** | | **d3 vs Baseline**  **Fold**  **Change P** | | **d7 vs Baseline**  **Fold**  **Change P** | |
| --- | --- | --- | --- | --- | --- | --- | --- | --- | --- | --- |
| A_70_P016821 | MHCII-DRB1 | MHC DR beta chain | -2.10 | 0.002 | -1.75 | 0.012 | -1.26 | 0.249 | -1.09 | 0.652 |
| A_70_P016851 | TRAC | T cell receptor alpha constant | -3.36 | 0.001 | -2.17 | 0.017 | -1.56 | 0.138 | -1.10 | 0.730 |
| A_70_P016856 | GJB2 | gap junction protein, beta 2, 26kDa | 1.54 | 0.177 | 2.46 | 0.011 | 4.33 | 0.000 | 3.39 | 0.002 |
| A_70_P016857 | GJB2 | gap junction protein, beta 2, 26kDa | 1.51 | 0.189 | 2.39 | 0.012 | 4.05 | 0.000 | 3.27 | 0.002 |
| A_70_P016861 | CSF3 | colony stimulating factor 3 (granulocyte) | 3.85 | 0.001 | 2.63 | 0.006 | 1.76 | 0.079 | 1.00 | 0.990 |
| A_70_P016862 | CSF3 | colony stimulating factor 3 (granulocyte) | 3.48 | 0.001 | 2.36 | 0.009 | 1.73 | 0.072 | 1.06 | 0.843 |
| A_70_P016866 | CALR | calreticulin | 1.59 | 0.050 | 2.06 | 0.006 | 1.31 | 0.234 | 1.31 | 0.234 |
| A_70_P016891 | CD3G | CD3g molecule, gamma (CD3-TCR complex) | -3.25 | 0.001 | -2.22 | 0.015 | -1.56 | 0.139 | -1.06 | 0.827 |
| A_70_P016916 | LPL | lipoprotein lipase | -3.39 | 0.000 | -3.11 | 0.000 | -1.69 | 0.019 | -1.44 | 0.088 |
| A_70_P016951 | TCRD19 | T-cell receptor delta 19 | -2.97 | 0.005 | -1.75 | 0.102 | -2.07 | 0.039 | 1.26 | 0.477 |
| A_70_P016952 | TCRD19 | T-cell receptor delta 19 | -2.90 | 0.006 | -1.72 | 0.118 | -2.13 | 0.038 | 1.29 | 0.443 |
| A_70_P017026 | CYP3A24 | cytochrome P450 CYP3A24 | -2.10 | 0.011 | -1.69 | 0.057 | -1.95 | 0.020 | -1.47 | 0.151 |
| A_70_P017036 | HOXA4 | homeobox A4 | -2.08 | 0.001 | -1.44 | 0.060 | -1.45 | 0.055 | -1.51 | 0.035 |
| A_70_P017037 | HOXA4 | homeobox A4 | -2.12 | 0.001 | -1.43 | 0.066 | -1.49 | 0.045 | -1.57 | 0.027 |
| A_70_P017041 | HOXA5 | homeobox A5 | -2.17 | 0.001 | -1.91 | 0.003 | -1.65 | 0.015 | -1.29 | 0.174 |
| A_70_P017042 | HOXA5 | homeobox A5 | -2.21 | 0.001 | -1.91 | 0.004 | -1.69 | 0.013 | -1.31 | 0.161 |
| A_70_P017047 | HOXA7 | homeobox A7 | -1.96 | 0.004 | -2.01 | 0.003 | -1.86 | 0.006 | -1.20 | 0.361 |
| A_70_P017096 | IGFBP4 | insulin-like growth factor binding protein 4 | -1.04 | 0.793 | 1.94 | 0.001 | 2.19 | 0.000 | 1.36 | 0.065 |
| A_70_P017097 | IGFBP4 | insulin-like growth factor binding protein 4 | -1.06 | 0.723 | 1.86 | 0.002 | 2.07 | 0.001 | 1.34 | 0.086 |
| A_70_P017111 | NOS3 | nitric oxide synthase 3 (endothelial cell) | 2.79 | 0.005 | 2.94 | 0.003 | 2.60 | 0.007 | 1.81 | 0.069 |
| A_70_P017136 | AREG | amphiregulin | 2.16 | 0.028 | 2.18 | 0.026 | 1.86 | 0.066 | 2.40 | 0.015 |
| A_70_P017321 | DHFR | dihydrofolate reductase | -1.65 | 0.018 | 2.04 | 0.002 | 1.06 | 0.768 | 1.07 | 0.730 |

| **Probenames** | **Gene Symbol** | **Gene Name** | **6h vs Baseline**  **Fold**  **Change P** | | **d1 vs Baseline**  **Fold**  **Change P** | | **d3 vs Baseline**  **Fold**  **Change P** | | **d7 vs Baseline**  **Fold**  **Change P** | |
| --- | --- | --- | --- | --- | --- | --- | --- | --- | --- | --- |
| A_70_P017516 | SMAD6 | SMAD family member 6 | -4.11 | 0.001 | -2.54 | 0.011 | -2.62 | 0.009 | -1.49 | 0.224 |
| A_70_P017561 | DHRS3 | dehydrogenase/reductase (SDR family) member 3 | -1.80 | 0.025 | -2.05 | 0.009 | -1.98 | 0.011 | -1.53 | 0.088 |
| A_70_P017566 | DTYMK | deoxythymidylate kinase (thymidylate kinase) | 1.16 | 0.373 | 2.93 | 0.000 | 2.12 | 0.001 | -1.04 | 0.815 |
| A_70_P017641 | C1QTNF3 | C1q and tumor necrosis factor related protein 3 | 1.23 | 0.470 | -1.40 | 0.241 | 1.41 | 0.234 | 4.15 | 0.000 |
| A_70_P017646 | LPXN | leupaxin | -2.34 | 0.000 | -1.72 | 0.003 | -1.48 | 0.019 | -1.08 | 0.604 |
| A_70_P017776 | LRRC25 | leucine rich repeat containing 25 | -2.00 | 0.020 | -1.20 | 0.498 | 1.11 | 0.683 | 1.58 | 0.100 |
| A_70_P017786 | MEF2C | myocyte enhancer factor 2C | -2.55 | 0.000 | -1.32 | 0.163 | 1.02 | 0.930 | 1.12 | 0.567 |
| A_70_P017866 | PLAUR | plasminogen activator, urokinase receptor | 2.02 | 0.059 | 2.80 | 0.010 | 5.51 | 0.000 | 6.62 | 0.000 |
| A_70_P017881 | GPNMB | glycoprotein (transmembrane) nmb | -1.40 | 0.188 | -1.37 | 0.215 | 2.38 | 0.004 | 1.70 | 0.047 |
| A_70_P017891 | LCP1 | lymphocyte cytosolic protein 1 (L-plastin) | -2.16 | 0.020 | -1.34 | 0.325 | 1.06 | 0.847 | 1.71 | 0.086 |
| A_70_P017916 | S100A11 | S100 calcium binding protein A11 | 1.66 | 0.013 | 2.06 | 0.001 | 1.45 | 0.056 | 1.32 | 0.142 |
| A_70_P017926 | TSKU | tsukushi small leucine rich proteoglycan homolog (Xenopus laevis | -3.06 | 0.001 | -1.95 | 0.017 | -1.68 | 0.054 | -2.01 | 0.014 |
| A_70_P017946 | RFC2 | replication factor C (activator 1) 2, 40kDa | 1.03 | 0.850 | 2.34 | 0.000 | 1.52 | 0.018 | 1.05 | 0.737 |
| A_70_P017971 | HADH | hydroxyacyl-CoA dehydrogenase | -2.03 | 0.001 | -1.35 | 0.084 | -1.47 | 0.033 | -1.42 | 0.051 |
| A_70_P018001 | DDAH2 | dimethylarginine dimethylaminohydrolase 2 | -2.02 | 0.001 | -1.49 | 0.034 | 1.09 | 0.633 | -1.26 | 0.196 |
| A_70_P018051 | C6orf115 | chromosome 6 open reading frame 115 | 1.23 | 0.260 | 1.95 | 0.003 | 2.28 | 0.001 | 1.25 | 0.224 |
| A_70_P018071 | NPC2 | Niemann-Pick disease, type C2 | -2.20 | 0.002 | -1.19 | 0.376 | -1.09 | 0.679 | 1.11 | 0.610 |
| A_70_P018086 | MYL6 | myosin, light chain 6, alkali, smooth muscle and non-muscle | -1.54 | 0.011 | 1.13 | 0.416 | 2.36 | 0.000 | -1.38 | 0.043 |

| **Probenames** | **Gene Symbol** | **Gene Name** | **6h vs Baseline**  **Fold**  **Change P** | | **d1 vs Baseline**  **Fold**  **Change P** | | **d3 vs Baseline**  **Fold**  **Change P** | | **d7 vs Baseline**  **Fold**  **Change P** | |
| --- | --- | --- | --- | --- | --- | --- | --- | --- | --- | --- |
| A_70_P018186 | TNC | tenascin C | 1.33 | 0.321 | 2.47 | 0.006 | 1.87 | 0.041 | 3.44 | 0.001 |
| A_70_P018201 | CYB5R3 | cytochrome b5 reductase 3 | -2.03 | 0.018 | -1.25 | 0.402 | -1.30 | 0.328 | -1.19 | 0.524 |
| A_70_P018251 | FOLR1 | folate receptor 1 (adult) | -1.02 | 0.938 | 2.58 | 0.008 | 4.05 | 0.001 | 1.83 | 0.067 |
| A_70_P018256 | S100A4 | S100 calcium binding protein A4 | 1.39 | 0.121 | 1.78 | 0.013 | 2.60 | 0.000 | 1.14 | 0.511 |
| A_70_P018331 | CKS2 | CDC28 protein kinase regulatory subunit 2 | -1.86 | 0.004 | 4.53 | 0.000 | 6.43 | 0.000 | 1.78 | 0.006 |
| A_70_P018361 | MRPL17 | mitochondrial ribosomal protein L17 | 1.49 | 0.041 | 2.26 | 0.001 | 1.81 | 0.005 | 1.01 | 0.945 |
| A_70_P018376 | FAP | fibroblast activation protein, alpha | -1.06 | 0.797 | 1.57 | 0.064 | 2.91 | 0.000 | 3.11 | 0.000 |
| A_70_P018381 | C9orf16 | chromosome 9 open reading frame 16 | 1.02 | 0.932 | 1.88 | 0.007 | 2.31 | 0.001 | -1.09 | 0.669 |
| A_70_P018436 | CRABP2 | cellular retinoic acid binding protein 2 | 3.10 | 0.005 | 3.54 | 0.002 | 6.94 | 0.000 | 5.27 | 0.000 |
| A_70_P018606 | OLFM4 | olfactomedin 4 | 6.56 | 0.000 | 7.87 | 0.000 | 2.54 | 0.020 | 1.10 | 0.780 |
| A_70_P018671 | JUN | jun proto-oncogene | -1.41 | 0.222 | -1.71 | 0.066 | -2.25 | 0.010 | -2.18 | 0.013 |
| A_70_P018716 | CIRBP | cold inducible RNA binding protein | -2.60 | 0.002 | -2.56 | 0.002 | -2.77 | 0.001 | -1.94 | 0.016 |
| A_70_P018816 | WDR6 | WD repeat domain 6 | -2.10 | 0.018 | -1.12 | 0.672 | -1.01 | 0.967 | -1.19 | 0.527 |
| A_70_P018866 | IGFBP3 | insulin-like growth factor binding protein 3 | 1.20 | 0.399 | 2.35 | 0.001 | 2.96 | 0.000 | 2.49 | 0.001 |
| A_70_P018906 | KIAA0528 | KIAA0528 | -2.09 | 0.002 | -1.31 | 0.191 | -1.49 | 0.059 | -1.23 | 0.298 |
| A_70_P018971 | CDCA7 | cell division cycle associated 7 | -1.41 | 0.206 | 2.72 | 0.002 | 1.81 | 0.040 | 1.03 | 0.909 |
| A_70_P019046 | MPV17L2 | MPV17 mitochondrial membrane protein-like 2 | 2.11 | 0.001 | 2.29 | 0.001 | 1.93 | 0.003 | 1.18 | 0.378 |
| A_70_P019121 | SUN2 | Sad1 and UNC84 domain containing 2 | -1.73 | 0.002 | -2.00 | 0.000 | -1.27 | 0.108 | -1.65 | 0.003 |
| A_70_P019126 | GREM1 | gremlin 1, cysteine knot superfamily, homolog (Xenopus laevis) | 1.25 | 0.366 | 1.84 | 0.026 | 3.92 | 0.000 | 4.10 | 0.000 |
| A_70_P019201 | RRP9 | ribosomal RNA processing 9, small subunit (SSU) processome compo | 2.43 | 0.000 | 1.74 | 0.010 | -1.17 | 0.411 | -1.13 | 0.508 |

| **Probenames** | **Gene Symbol** | **Gene Name** | **6h vs Baseline**  **Fold**  **Change P** | | **d1 vs Baseline**  **Fold**  **Change P** | | **d3 vs Baseline**  **Fold**  **Change P** | | **d7 vs Baseline**  **Fold**  **Change P** | |
| --- | --- | --- | --- | --- | --- | --- | --- | --- | --- | --- |
| A_70_P019226 | GCAT | glycine C-acetyltransferase | -1.21 | 0.415 | 1.21 | 0.401 | -1.68 | 0.037 | -2.36 | 0.002 |
| A_70_P019426 | ANKH | ankylosis, progressive homolog (mouse) | -1.32 | 0.108 | -1.08 | 0.644 | 1.56 | 0.016 | 2.30 | 0.000 |
| A_70_P019461 | WBSCR27 | Williams Beuren syndrome chromosome region 27 | -1.73 | 0.001 | -1.49 | 0.011 | -1.55 | 0.006 | -2.17 | 0.000 |
| A_70_P019501 | C1QTNF7 | C1q and tumor necrosis factor related protein 7 | -2.54 | 0.002 | -3.25 | 0.000 | -1.83 | 0.026 | -1.48 | 0.124 |
| A_70_P019536 | TNS3 | tensin 3 | -2.22 | 0.008 | -1.64 | 0.073 | -1.96 | 0.021 | -1.23 | 0.422 |
| A_70_P019556 | CXCL12 | chemokine (C-X-C motif) ligand 12 | -2.61 | 0.001 | -1.80 | 0.017 | 1.72 | 0.025 | 1.63 | 0.041 |
| A_70_P019621 | TLK2 | tousled-like kinase 2 | 1.77 | 0.166 | 1.74 | 0.178 | 2.51 | 0.035 | -1.34 | 0.464 |
| A_70_P019641 | SYNE2 | spectrin repeat containing, nuclear envelope 2 | -2.56 | 0.002 | -1.65 | 0.059 | -1.57 | 0.083 | -1.36 | 0.220 |
| A_70_P019656 | ADH1C | alcohol dehydrogenase 1C (class I), gamma polypeptide | -9.67 | 0.002 | -9.59 | 0.002 | -13.72 | 0.001 | -2.89 | 0.087 |
| A_70_P019681 | SAA3 | serum amyloid A 3 (SAA3) | 14.97 | 0.000 | 20.68 | 0.000 | 14.74 | 0.000 | 9.68 | 0.000 |
| A_70_P019721 | C4BPA | component 4 binding protein, alpha (C4BPA) | 3.70 | 0.000 | 4.89 | 0.000 | 3.80 | 0.000 | 2.41 | 0.004 |
| A_70_P019746 | HSD17B13 | hydroxysteroid (17-beta) dehydrogenase 13 | -8.23 | 0.015 | -4.62 | 0.060 | -10.80 | 0.007 | -1.63 | 0.520 |
| A_70_P019811 | ATG4C | ATG4 autophagy related 4 homolog C (S. cerevisiae) | -2.06 | 0.001 | -1.22 | 0.215 | -1.21 | 0.236 | -1.24 | 0.184 |
| A_70_P019866 | DTNA | dystrobrevin, alpha | -2.65 | 0.015 | -2.74 | 0.012 | -4.68 | 0.001 | -2.34 | 0.029 |
| A_70_P020086 | LMO2 | LIM domain only 2 (rhombotin-like 1) | -2.51 | 0.000 | -1.56 | 0.025 | -1.06 | 0.724 | -1.14 | 0.471 |
| A_70_P020236 | C19orf61 | chromosome 19 open reading frame 61 | -14.03 | 0.003 | -7.55 | 0.014 | -11.52 | 0.005 | -2.90 | 0.157 |
| A_70_P020271 | CRELD2 | cysteine-rich with EGF-like domains 2 | 3.08 | 0.000 | 3.84 | 0.000 | 1.64 | 0.027 | 1.31 | 0.198 |
| A_70_P020286 | CLDN2 | claudin 2 | 2.94 | 0.001 | 2.83 | 0.001 | 2.57 | 0.002 | 1.10 | 0.683 |

| **Probenames** | **Gene Symbol** | **Gene Name** | **6h vs Baseline**  **Fold**  **Change P** | | **d1 vs Baseline**  **Fold**  **Change P** | | **d3 vs Baseline**  **Fold**  **Change P** | | **d7 vs Baseline**  **Fold**  **Change P** | |
| --- | --- | --- | --- | --- | --- | --- | --- | --- | --- | --- |
| A_70_P020351 | NAP1L5 | nucleosome assembly protein 1-like 5 | -2.56 | 0.001 | -2.31 | 0.003 | -2.21 | 0.005 | -1.39 | 0.172 |
| A_70_P020361 | NEIL2 | nei endonuclease VIII-like 2 (E. coli) | -2.17 | 0.002 | -1.33 | 0.163 | -1.23 | 0.309 | -1.48 | 0.065 |
| A_70_P020476 | SLC7A5 | solute carrier family 7 (cationic amino acid transporter, y+ system), member 5 | 1.99 | 0.003 | 2.14 | 0.002 | 1.41 | 0.088 | 1.23 | 0.287 |
| A_70_P020536 | FKBP11 | FK506 binding protein 11, 19 kDa | 2.06 | 0.003 | 2.75 | 0.000 | 2.21 | 0.002 | -1.01 | 0.962 |
| A_70_P020661 | MGST1 | microsomal glutathione S-transferase 1 | -2.21 | 0.000 | -1.27 | 0.107 | -1.24 | 0.149 | -1.69 | 0.003 |
| A_70_P020761 | LOC789682 | similar to Mast cell antigen 32 precursor (Mast cell Ag-32) (MCA-32) | -1.23 | 0.439 | 1.22 | 0.447 | 2.58 | 0.003 | 1.91 | 0.027 |
| A_70_P020856 | LRRC59 | leucine rich repeat containing 59 | 1.98 | 0.017 | 2.34 | 0.005 | 1.51 | 0.121 | 1.33 | 0.262 |
| A_70_P020861 | OCIAD2 | OCIA domain containing 2 | -2.72 | 0.000 | -1.51 | 0.053 | -1.82 | 0.009 | -1.77 | 0.011 |
| A_70_P020866 | IFI30 | interferon, gamma-inducible protein 30 | 1.05 | 0.827 | 1.41 | 0.122 | 2.68 | 0.000 | 1.74 | 0.020 |
| A_70_P020946 | CCL2 | chemokine (C-C motif) ligand 2 | 9.23 | 0.000 | 8.73 | 0.000 | 5.52 | 0.000 | 2.86 | 0.004 |
| A_70_P020951 | CDC25B | cell division cycle 25 homolog B (S. pombe) | -2.02 | 0.003 | 1.43 | 0.081 | 1.49 | 0.054 | 1.64 | 0.022 |
| A_70_P021026 | STMN1 | stathmin 1 | -2.22 | 0.000 | 1.93 | 0.000 | 2.73 | 0.000 | 1.45 | 0.006 |
| A_70_P021121 | THBS4 | thrombospondin 4 | 1.31 | 0.516 | 1.42 | 0.404 | 2.42 | 0.050 | 4.49 | 0.003 |
| A_70_P021136 | DMPK | dystrophia myotonica-protein kinase | -2.11 | 0.021 | -1.58 | 0.132 | -1.60 | 0.123 | -1.33 | 0.335 |
| A_70_P021206 | PRDX4 | peroxiredoxin 4 | 1.10 | 0.570 | 1.75 | 0.005 | 2.48 | 0.000 | 1.05 | 0.753 |
| A_70_P021211 | OGN | osteoglycin | -2.46 | 0.007 | -4.37 | 0.000 | -1.80 | 0.058 | -1.19 | 0.553 |
| A_70_P021236 | CUEDC1 | CUE domain containing 1 | -2.03 | 0.006 | -1.31 | 0.219 | -1.54 | 0.062 | -1.36 | 0.168 |
| A_70_P021321 | EMCN | endomucin | -2.47 | 0.001 | -1.37 | 0.174 | -1.08 | 0.732 | -1.11 | 0.628 |
| A_70_P021346 | USP11 | ubiquitin specific peptidase 11 | -2.09 | 0.003 | -1.57 | 0.043 | -1.86 | 0.008 | -1.15 | 0.500 |
| A_70_P021361 | SORBS3 | sorbin and SH3 domain containing 3 | -2.10 | 0.009 | -1.34 | 0.245 | -1.36 | 0.223 | -1.21 | 0.452 |
| A_70_P021371 | SMTN | smoothelin | -1.72 | 0.025 | -1.60 | 0.049 | -1.66 | 0.035 | -2.04 | 0.006 |

| **Probenames** | **Gene Symbol** | **Gene Name** | **6h vs Baseline**  **Fold**  **Change P** | | **d1 vs Baseline**  **Fold**  **Change P** | | **d3 vs Baseline**  **Fold**  **Change P** | | **d7 vs Baseline**  **Fold**  **Change P** | |
| --- | --- | --- | --- | --- | --- | --- | --- | --- | --- | --- |
| A_70_P021381 | LOC505941 | similar to KIAA1398 protein | 1.86 | 0.005 | 2.15 | 0.001 | 1.44 | 0.066 | 1.33 | 0.139 |
| A_70_P021426 | GALM | galactose mutarotase (aldose 1-epimerase) | -2.08 | 0.007 | -1.27 | 0.311 | -1.38 | 0.180 | -1.32 | 0.246 |
| A_70_P021436 | SMOC2 | SPARC related modular calcium binding 2 | 1.40 | 0.249 | 1.27 | 0.407 | 2.53 | 0.006 | 3.51 | 0.001 |
| A_70_P021491 | C1QTNF6 | C1q and tumor necrosis factor related protein 6 | 1.27 | 0.226 | 1.62 | 0.024 | 3.37 | 0.000 | 1.76 | 0.011 |
| A_70_P021596 | ADAM12 | ADAM metallopeptidase domain 12 | 2.26 | 0.039 | 3.78 | 0.003 | 11.08 | 0.000 | 16.37 | 0.000 |
| A_70_P021636 | CPZ | carboxypeptidase Z | 1.15 | 0.369 | 1.08 | 0.613 | 2.16 | 0.000 | 1.72 | 0.003 |
| A_70_P021666 | FTSJ3 | FtsJ homolog 3 (E. coli) | 2.10 | 0.004 | 1.79 | 0.018 | 1.05 | 0.837 | -1.07 | 0.760 |
| A_70_P021711 | COL1A1 | collagen, type I, alpha 1 | -1.01 | 0.955 | 1.69 | 0.050 | 3.21 | 0.000 | 2.99 | 0.001 |
| A_70_P021761 | TYMS | thymidylate synthetase | -1.14 | 0.300 | 5.92 | 0.000 | 5.53 | 0.000 | 2.36 | 0.000 |
| A_70_P021766 | UBE2J1 | ubiquitin-conjugating enzyme E2, J1 (UBC6 homolog, yeast) | 1.49 | 0.042 | 2.37 | 0.000 | 2.29 | 0.000 | 1.51 | 0.035 |
| A_70_P021781 | DSG3 | desmoglein 3 (pemphigus vulgaris antigen) | 1.41 | 0.248 | 2.72 | 0.004 | 5.02 | 0.000 | 3.71 | 0.001 |
| A_70_P021796 | CAMK2G | calcium/calmodulin-dependent protein kinase II gamma | -2.65 | 0.001 | -1.95 | 0.009 | -1.97 | 0.008 | -1.49 | 0.087 |
| A_70_P021836 | SYNCRIP | synaptotagmin binding, cytoplasmic RNA interacting protein | 1.70 | 0.007 | 2.02 | 0.001 | 1.53 | 0.022 | 1.09 | 0.605 |
| A_70_P021871 | NNAT | neuronatin | 1.45 | 0.379 | 1.08 | 0.851 | 1.20 | 0.664 | -2.44 | 0.047 |
| A_70_P022026 | LOC533307 | tubulin, beta 5 | 1.04 | 0.777 | 2.15 | 0.000 | 1.66 | 0.003 | 1.44 | 0.021 |
| A_70_P022106 | TRMT1L | TRM1 tRNA methyltransferase 1-like | -2.19 | 0.000 | -1.36 | 0.092 | -1.30 | 0.142 | -1.50 | 0.031 |
| A_70_P022161 | NOLC1 | nucleolar and coiled-body phosphoprotein 1 | 2.32 | 0.002 | 2.01 | 0.007 | 1.01 | 0.982 | -1.08 | 0.716 |
| A_70_P022226 | CYB5 | CYB5 protein (CYB5) | -2.46 | 0.000 | -1.65 | 0.010 | -1.70 | 0.007 | -1.92 | 0.002 |

| **Probenames** | **Gene Symbol** | **Gene Name** | **6h vs Baseline**  **Fold**  **Change P** | | **d1 vs Baseline**  **Fold**  **Change P** | | **d3 vs Baseline**  **Fold**  **Change P** | | **d7 vs Baseline**  **Fold**  **Change P** | |
| --- | --- | --- | --- | --- | --- | --- | --- | --- | --- | --- |
| A_70_P022366 | TCF7 | transcription factor 7 (T-cell specific, HMG-box) | -2.19 | 0.001 | -1.58 | 0.031 | 1.01 | 0.962 | 1.38 | 0.111 |
| A_70_P022436 | CENPT | centromere protein T | -1.04 | 0.721 | 2.45 | 0.000 | 2.38 | 0.000 | 1.44 | 0.006 |
| A_70_P022451 | SPTBN1 | spectrin, beta, non-erythrocytic 1 | -1.45 | 0.103 | -1.70 | 0.028 | -2.61 | 0.001 | -1.61 | 0.044 |
| A_70_P022511 | FGD3 | FYVE, RhoGEF and PH domain containing 3 | -2.59 | 0.005 | -1.58 | 0.125 | -1.67 | 0.091 | 1.15 | 0.631 |
| A_70_P022621 | FUT1 | fucosyltransferase 1 (galactoside 2-alpha-L-fucosyltransferase, H blood group) | 1.40 | 0.016 | 2.05 | 0.000 | 1.51 | 0.005 | 1.13 | 0.339 |
| A_70_P022691 | FAM107A | family with sequence similarity 107, member A | -1.87 | 0.026 | -1.49 | 0.133 | -1.93 | 0.021 | -2.13 | 0.010 |
| A_70_P022696 | 37681 | membrane-associated ring finger (C3HC4) 3 | 1.94 | 0.006 | 2.18 | 0.002 | 1.94 | 0.006 | 1.62 | 0.031 |
| A_70_P022726 | SH2D2A | SH2 domain containing 2A | -2.01 | 0.003 | -1.55 | 0.036 | -1.70 | 0.014 | 1.14 | 0.492 |
| A_70_P022736 | CC2D1B | coiled-coil and C2 domain containing 1B | -2.30 | 0.003 | -1.46 | 0.119 | -1.80 | 0.022 | -1.28 | 0.297 |
| A_70_P022796 | COCH | coagulation factor C homolog, cochlin (Limulus polyphemus) | -1.59 | 0.278 | -3.11 | 0.017 | -2.12 | 0.090 | -1.62 | 0.260 |
| A_70_P022876 | SH3D20 | SH3 domain containing 20 | -2.18 | 0.002 | -1.32 | 0.194 | -1.11 | 0.606 | -1.12 | 0.593 |
| A_70_P022951 | FMO2 | flavin containing monooxygenase 2 (non-functional) | -4.51 | 0.001 | -2.71 | 0.011 | -3.08 | 0.005 | -2.11 | 0.043 |
| A_70_P022976 | LY6G6E | lymphocyte antigen 6 complex, locus G6E | -1.59 | 0.193 | -2.21 | 0.036 | -1.01 | 0.981 | -1.06 | 0.857 |
| A_70_P023156 | DOCK4 | dedicator of cytokinesis 4 (DOCK4) | -3.02 | 0.000 | -1.83 | 0.001 | -1.14 | 0.371 | -1.01 | 0.929 |
| A_70_P023186 | FBXO15 | F-box protein 15 | -2.72 | 0.017 | -1.78 | 0.140 | -2.88 | 0.013 | -1.80 | 0.130 |
| A_70_P023641 | CDCA7L | cell division cycle associated 7-like | -2.34 | 0.036 | -1.80 | 0.130 | -3.38 | 0.006 | -1.71 | 0.164 |
| A_70_P024096 | PPL | periplakin | -2.03 | 0.003 | -1.62 | 0.026 | -1.46 | 0.071 | -1.49 | 0.059 |
| A_70_P024116 | NOX4 | NADPH oxidase 4 | 1.02 | 0.902 | 1.80 | 0.001 | 2.38 | 0.000 | 2.09 | 0.000 |

| **Probenames** | **Gene Symbol** | **Gene Name** | **6h vs Baseline**  **Fold**  **Change P** | | **d1 vs Baseline**  **Fold**  **Change P** | | **d3 vs Baseline**  **Fold**  **Change P** | | **d7 vs Baseline**  **Fold**  **Change P** | |
| --- | --- | --- | --- | --- | --- | --- | --- | --- | --- | --- |
| A_70_P024117 | NOX4 | NADPH oxidase 4 | 1.04 | 0.785 | 1.81 | 0.001 | 2.13 | 0.000 | 1.87 | 0.001 |
| A_70_P024121 | NOX1 | NADPH oxidase 1 | 1.10 | 0.732 | 1.26 | 0.415 | 1.54 | 0.135 | 2.78 | 0.003 |
| A_70_P024122 | NOX1 | NADPH oxidase 1 | -1.02 | 0.941 | 1.25 | 0.465 | 1.49 | 0.195 | 3.15 | 0.002 |
| A_70_P024221 | COL1A2 | collagen, type I, alpha 2 | -1.13 | 0.657 | -1.02 | 0.953 | 3.75 | 0.000 | 6.99 | 0.000 |
| A_70_P024331 | TNMD | tenomodulin | -1.73 | 0.086 | -3.90 | 0.001 | -2.28 | 0.016 | -1.93 | 0.045 |
| A_70_P024332 | TNMD | tenomodulin | -1.67 | 0.077 | -3.71 | 0.000 | -2.31 | 0.008 | -2.11 | 0.016 |
| A_70_P024476 | COL3A1 | collagen, type III, alpha 1 | -1.28 | 0.391 | -1.16 | 0.609 | 3.33 | 0.001 | 5.99 | 0.000 |
| A_70_P024496 | COL1A1 | collagen, type I, alpha 1 | 1.33 | 0.275 | 1.17 | 0.548 | 3.59 | 0.000 | 4.37 | 0.000 |
| A_70_P024506 | COL1A1 | collagen, type I, alpha 1 | -1.07 | 0.702 | -1.08 | 0.674 | 1.85 | 0.004 | 2.26 | 0.000 |
| A_70_P024546 | NOX5 | NADPH oxidase, EF-hand calcium binding domain 5 | 2.04 | 0.030 | 2.29 | 0.015 | 3.59 | 0.001 | 1.16 | 0.610 |
| A_70_P024601 | COL1A2 | collagen, type I, alpha 2 | -1.27 | 0.214 | -1.17 | 0.392 | 2.17 | 0.001 | 3.39 | 0.000 |
| A_70_P024621 | COL1A1 | collagen, type I, alpha 1 | 1.31 | 0.279 | 1.08 | 0.764 | 4.19 | 0.000 | 6.55 | 0.000 |
| A_70_P024641 | SFRP1 | secreted frizzled-related protein 1 | 1.35 | 0.227 | 2.00 | 0.013 | 2.03 | 0.012 | 1.96 | 0.015 |
| A_70_P024651 | CITED2 | Cbp/p300-interacting transactivator, with Glu/Asp-rich carboxy-t | -2.25 | 0.003 | -1.94 | 0.012 | -2.15 | 0.005 | -1.68 | 0.039 |
| A_70_P024652 | CITED2 | Cbp/p300-interacting transactivator, with Glu/Asp-rich carboxy-t | -2.23 | 0.003 | -1.91 | 0.012 | -2.22 | 0.003 | -1.71 | 0.030 |
| A_70_P024826 | C13orf15 | chromosome 13 open reading frame 15 | -3.55 | 0.000 | -2.43 | 0.006 | -1.84 | 0.040 | -1.28 | 0.373 |
| A_70_P024896 | ST3GAL2 | ST3 beta-galactoside alpha-2,3-sialyltransferase 2 | -2.55 | 0.000 | -1.62 | 0.025 | -1.36 | 0.131 | 1.16 | 0.459 |
| A_70_P024916 | CEP110 | centrosomal protein 110kDa | -2.01 | 0.013 | -1.59 | 0.080 | -1.77 | 0.036 | -1.40 | 0.188 |
| A_70_P024971 | NCAPH | non-SMC condensin I complex, subunit H | -1.96 | 0.002 | 2.12 | 0.001 | 1.93 | 0.003 | 1.50 | 0.039 |

| **Probenames** | **Gene Symbol** | **Gene Name** | **6h vs Baseline**  **Fold**  **Change P** | | **d1 vs Baseline**  **Fold**  **Change P** | | **d3 vs Baseline**  **Fold**  **Change P** | | **d7 vs Baseline**  **Fold**  **Change P** | |
| --- | --- | --- | --- | --- | --- | --- | --- | --- | --- | --- |
| A_70_P024986 | CD8A | CD8a molecule | -1.78 | 0.031 | -1.83 | 0.026 | -2.18 | 0.006 | 1.06 | 0.801 |
| A_70_P025016 | KCNN4 | potassium intermediate/small conductance calcium-activated chann | 2.89 | 0.001 | 2.23 | 0.008 | -1.01 | 0.979 | -1.26 | 0.380 |
| A_70_P025086 | GRK5 | G protein-coupled receptor kinase 5 | -2.42 | 0.003 | -1.96 | 0.017 | -2.10 | 0.010 | -1.27 | 0.349 |
| A_70_P025121 | PLA2G2A | phospholipase A2, group IIA (platelets, synovial fluid) | 2.22 | 0.049 | 1.46 | 0.318 | 2.69 | 0.019 | -1.41 | 0.370 |
| A_70_P025211 | SPOCK2 | sparc/osteonectin, cwcv and kazal-like domains proteoglycan (tes | -2.07 | 0.004 | -1.89 | 0.008 | -1.80 | 0.013 | -1.01 | 0.945 |
| A_70_P025276 | ADA | adenosine deaminase | 1.54 | 0.052 | 2.18 | 0.002 | 1.93 | 0.006 | 1.53 | 0.056 |
| A_70_P025536 | GRM8 | glutamate receptor, metabotropic 8 | -3.02 | 0.002 | -2.55 | 0.006 | -2.60 | 0.006 | -1.23 | 0.483 |
| A_70_P025736 | TXK | TXK tyrosine kinase | -2.05 | 0.003 | -1.42 | 0.086 | -1.26 | 0.248 | 1.30 | 0.191 |
| A_70_P025991 | PHAX | phosphorylated adaptor for RNA export | 2.86 | 0.012 | 2.30 | 0.037 | 2.32 | 0.036 | -1.37 | 0.391 |
| A_70_P026001 | KIAA1826 | KIAA1826 | 1.32 | 0.278 | 1.44 | 0.163 | 2.21 | 0.007 | -1.07 | 0.795 |
| A_70_P026056 | ITGAV | integrin, alpha V (vitronectin receptor, alpha polypeptide, antigen CD51)" | 1.13 | 0.483 | 1.31 | 0.127 | 2.61 | 0.000 | 2.58 | 0.000 |
| A_70_P026057 | ITGAV | integrin, alpha V (vitronectin receptor, alpha polypeptide, antigen CD51)" | 1.04 | 0.810 | 1.28 | 0.170 | 2.48 | 0.000 | 2.55 | 0.000 |
| A_70_P026066 | AQP3 | aquaporin 3 | -2.46 | 0.019 | -1.85 | 0.091 | -1.11 | 0.765 | -2.33 | 0.027 |
| A_70_P026067 | AQP3 | aquaporin 3 (Gill blood group) | -2.46 | 0.018 | -1.89 | 0.077 | -1.10 | 0.773 | -2.25 | 0.030 |
| A_70_P026096 | OCLN | occludin | -1.97 | 0.007 | -1.75 | 0.020 | -2.01 | 0.006 | -1.36 | 0.163 |
| A_70_P026156 | EM4e | EM4e protein | -2.18 | 0.004 | -1.44 | 0.118 | -1.50 | 0.087 | -1.27 | 0.298 |
| A_70_P026157 | EM4e | EM4e protein | -2.08 | 0.004 | -1.40 | 0.128 | -1.51 | 0.070 | -1.27 | 0.276 |
| A_70_P026161 | EP4a | EP4a protein | -2.26 | 0.002 | -1.53 | 0.070 | -1.57 | 0.058 | -1.30 | 0.247 |

| **Probenames** | **Gene Symbol** | **Gene Name** | **6h vs Baseline**  **Fold**  **Change P** | | **d1 vs Baseline**  **Fold**  **Change P** | | **d3 vs Baseline**  **Fold**  **Change P** | | **d7 vs Baseline**  **Fold**  **Change P** | |
| --- | --- | --- | --- | --- | --- | --- | --- | --- | --- | --- |
| A_70_P026162 | EP4a | EP4a protein | -2.18 | 0.002 | -1.47 | 0.082 | -1.49 | 0.073 | -1.22 | 0.354 |
| A_70_P026166 | EM4b | EM4b protein | -2.02 | 0.002 | -1.44 | 0.063 | -1.50 | 0.041 | -1.56 | 0.027 |
| A_70_P026171 | EP4d | EP4d protein | -3.31 | 0.001 | -1.86 | 0.035 | -2.02 | 0.020 | -1.90 | 0.030 |
| A_70_P026172 | EP4d | EP4d protein | -3.34 | 0.001 | -1.89 | 0.036 | -2.01 | 0.023 | -1.84 | 0.042 |
| A_70_P026196 | ART4 | ADP-ribosyltransferase 4 (Dombrock blood group) | -2.89 | 0.000 | -1.53 | 0.039 | -1.15 | 0.448 | -1.34 | 0.135 |
| A_70_P026197 | ART4 | ADP-ribosyltransferase 4 (Dombrock blood group) | -3.44 | 0.000 | -1.59 | 0.048 | -1.19 | 0.422 | -1.41 | 0.129 |
| A_70_P026206 | PCNA | proliferating cell nuclear antigen | -1.02 | 0.893 | 3.06 | 0.000 | 1.83 | 0.005 | 1.10 | 0.599 |
| A_70_P026207 | PCNA | proliferating cell nuclear antigen | 1.01 | 0.966 | 3.23 | 0.000 | 1.84 | 0.003 | 1.11 | 0.545 |
| A_70_P026211 | SPP1 | secreted phosphoprotein 1 | 8.30 | 0.000 | 5.79 | 0.001 | 6.45 | 0.001 | 7.51 | 0.000 |
| A_70_P026331 | LOC100138798 | similar to Vl1a protein | -1.53 | 0.226 | 1.38 | 0.349 | 2.37 | 0.024 | 1.89 | 0.080 |
| A_70_P026336 | LOC100138798 | similar to Vl1a protein | -1.50 | 0.252 | 1.41 | 0.322 | 2.20 | 0.037 | 1.95 | 0.070 |
| A_70_P026351 | LOC100138798 | similar to Vl1a protein | -1.32 | 0.432 | 1.46 | 0.283 | 2.29 | 0.031 | 2.08 | 0.052 |
| A_70_P026374 | LOC100138798 | similar to Vl1a protein | -1.33 | 0.358 | 1.36 | 0.316 | 2.28 | 0.016 | 1.96 | 0.042 |
| A_70_P026376 | GGT5 | gamma-glutamyltransferase 5 | -1.55 | 0.208 | 1.38 | 0.349 | 2.29 | 0.028 | 1.96 | 0.066 |
| A_70_P026386 | LOC100138798 | similar to Vl1a protein | -1.47 | 0.276 | 1.43 | 0.308 | 2.21 | 0.037 | 1.88 | 0.086 |
| A_70_P026391 | LOC100138798 | similar to Vl1a protein | -1.37 | 0.362 | 1.37 | 0.359 | 2.19 | 0.035 | 1.91 | 0.073 |
| A_70_P026401 | LOC100138798 | similar to Vl1a protein | -1.23 | 0.498 | 1.36 | 0.324 | 2.18 | 0.024 | 2.03 | 0.036 |
| A_70_P026411 | LOC789490 | similar to Ig lambda chain V-I region BL2 | -1.49 | 0.252 | 1.39 | 0.339 | 2.16 | 0.038 | 1.77 | 0.110 |
| A_70_P026421 | LOC511354 | immunoglobulin light chain variable region | -1.59 | 0.173 | 1.29 | 0.446 | 2.26 | 0.025 | 1.69 | 0.125 |

| **Probenames** | **Gene Symbol** | **Gene Name** | **6h vs Baseline**  **Fold**  **Change P** | | **d1 vs Baseline**  **Fold**  **Change P** | | **d3 vs Baseline**  **Fold**  **Change P** | | **d7 vs Baseline**  **Fold**  **Change P** | |
| --- | --- | --- | --- | --- | --- | --- | --- | --- | --- | --- |
| A_70_P026536 | MMP1 | matrix metallopeptidase 1 (interstitial collagenase) | 3.51 | 0.007 | 3.68 | 0.006 | 20.75 | 0.000 | 3.20 | 0.011 |
| A_70_P026537 | MMP1 | matrix metallopeptidase 1 (interstitial collagenase) | 3.51 | 0.008 | 3.84 | 0.005 | 20.84 | 0.000 | 3.27 | 0.011 |
| A_70_P026546 | MMP2 | matrix metallopeptidase 2 (gelatinase A, 72kDa gelatinase, 72kDa | -1.38 | 0.195 | -1.09 | 0.728 | 2.61 | 0.002 | 3.62 | 0.000 |
| A_70_P026547 | MMP2 | matrix metallopeptidase 2 (gelatinase A, 72kDa gelatinase, 72kDa | -1.37 | 0.212 | -1.02 | 0.932 | 2.71 | 0.001 | 3.55 | 0.000 |
| A_70_P026661 | RECEPTOR | neuropilin-1 | -2.37 | 0.002 | -1.64 | 0.049 | -1.13 | 0.598 | 1.02 | 0.922 |
| A_70_P026746 | AQP9 | aquaporin 9 | -2.07 | 0.005 | -1.35 | 0.180 | -1.37 | 0.160 | 1.12 | 0.611 |
| A_70_P026761 | MMP13 | matrix metallopeptidase 13 (collagenase 3) | 1.41 | 0.475 | 1.36 | 0.522 | 15.38 | 0.000 | 10.84 | 0.000 |
| A_70_P026976 | STMN2 | stathmin-like 2 | -1.54 | 0.033 | -2.01 | 0.002 | -1.21 | 0.305 | -1.02 | 0.933 |
| A_70_P027026 | ACAP1 | ArfGAP with coiled-coil, ankyrin repeat and PH domains 1 | -2.25 | 0.006 | -1.40 | 0.199 | -1.07 | 0.788 | 1.15 | 0.584 |
| A_70_P027046 | PIM2 | pim-2 oncogene | -2.28 | 0.004 | -1.72 | 0.039 | -1.93 | 0.015 | -1.45 | 0.141 |
| A_70_P027061 | ZC4H2 | zinc finger, C4H2 domain containing | -2.52 | 0.000 | -1.38 | 0.109 | -1.37 | 0.115 | -1.11 | 0.599 |
| A_70_P027176 | PHF15 | PHD finger protein 15 | -2.52 | 0.002 | -2.51 | 0.002 | -2.14 | 0.007 | -1.69 | 0.043 |
| A_70_P027266 | MCAM | melanoma cell adhesion molecule | -2.73 | 0.001 | -2.48 | 0.002 | -2.52 | 0.002 | -1.91 | 0.019 |
| A_70_P027296 | FGFR2 | fibroblast growth factor receptor 2 | -2.01 | 0.012 | -1.75 | 0.035 | -1.64 | 0.059 | -1.51 | 0.108 |
| A_70_P027331 | WDR77 | WD repeat domain 77 | 2.15 | 0.003 | 2.31 | 0.001 | 1.23 | 0.330 | 1.21 | 0.359 |
| A_70_P027426 | LOC526046 | surfeit 2 | 1.79 | 0.006 | 2.05 | 0.002 | 1.41 | 0.076 | 1.29 | 0.179 |

| **Probenames** | **Gene Symbol** | **Gene Name** | **6h vs Baseline**  **Fold**  **Change P** | | **d1 vs Baseline**  **Fold**  **Change P** | | **d3 vs Baseline**  **Fold**  **Change P** | | **d7 vs Baseline**  **Fold**  **Change P** | |
| --- | --- | --- | --- | --- | --- | --- | --- | --- | --- | --- |
| A_70_P027436 | SLC16A3 | solute carrier family 16, member 3 (monocarboxylic acid transpor | 1.03 | 0.834 | 1.46 | 0.030 | 2.17 | 0.000 | 1.60 | 0.010 |
| A_70_P027441 | CARHSP1 | calcium regulated heat stable protein 1, 24kDa | 1.00 | 0.996 | 1.75 | 0.006 | 2.70 | 0.000 | 1.14 | 0.433 |
| A_70_P027491 | SFXN2 | sideroflexin 2 | 1.30 | 0.353 | 2.33 | 0.009 | 1.49 | 0.171 | 1.39 | 0.255 |
| A_70_P027526 | GPC1 | glypican 1 | 1.26 | 0.346 | 1.34 | 0.239 | 2.18 | 0.006 | 1.07 | 0.774 |
| A_70_P027536 | BRB | brain ribonuclease | -1.72 | 0.121 | -1.11 | 0.761 | 2.98 | 0.006 | 1.74 | 0.116 |
| A_70_P027576 | EMP3 | epithelial membrane protein 3 | 1.46 | 0.314 | 2.50 | 0.026 | 4.21 | 0.002 | 2.31 | 0.039 |
| A_70_P027591 | H1FX | H1 histone family, member X | -2.71 | 0.000 | 1.06 | 0.774 | 1.14 | 0.531 | -1.12 | 0.585 |
| A_70_P027656 | UCP2 | uncoupling protein 2 (mitochondrial, proton carrier) | -2.96 | 0.001 | -1.76 | 0.031 | -1.37 | 0.204 | 1.04 | 0.884 |
| A_70_P027666 | SFRP1 | secreted frizzled-related protein 1 | 1.36 | 0.225 | 2.63 | 0.002 | 3.20 | 0.000 | 2.65 | 0.002 |
| A_70_P027706 | YKT6 | YKT6 v-SNARE homolog (S. cerevisiae) | 1.53 | 0.024 | 2.09 | 0.001 | 1.67 | 0.008 | 1.05 | 0.761 |
| A_70_P027721 | SRM | spermidine synthase | 3.85 | 0.000 | 4.07 | 0.000 | 2.43 | 0.002 | 1.32 | 0.240 |
| A_70_P027756 | LOC407171 | Fc gamma 2 receptor | 1.56 | 0.155 | 1.57 | 0.148 | 2.34 | 0.013 | 1.90 | 0.048 |
| A_70_P027781 | CD37 | CD37 molecule | -2.03 | 0.001 | -1.67 | 0.011 | 1.21 | 0.274 | 1.37 | 0.087 |
| A_70_P027811 | COL5A2 | collagen, type V, alpha 2 | -1.50 | 0.077 | 1.07 | 0.763 | 2.98 | 0.000 | 4.50 | 0.000 |
| A_70_P027826 | SLC35B1 | solute carrier family 35, member B1 | 1.78 | 0.004 | 2.20 | 0.000 | 1.67 | 0.008 | 1.23 | 0.235 |
| A_70_P027841 | CYGB | cytoglobin | 1.15 | 0.526 | 1.73 | 0.023 | 2.29 | 0.002 | 2.13 | 0.004 |
| A_70_P027966 | CDCA3 | cell division cycle associated 3 | -1.62 | 0.002 | 7.15 | 0.000 | 6.60 | 0.000 | 2.19 | 0.000 |
| A_70_P027971 | PRRX1 | paired related homeobox 1 | 1.09 | 0.653 | 1.76 | 0.011 | 3.23 | 0.000 | 1.82 | 0.008 |
| A_70_P027986 | PLXND1 | plexin D1 | -2.06 | 0.002 | -1.18 | 0.394 | 1.21 | 0.324 | 1.22 | 0.315 |

| **Probenames** | **Gene Symbol** | **Gene Name** | **6h vs Baseline**  **Fold**  **Change P** | | **d1 vs Baseline**  **Fold**  **Change P** | | **d3 vs Baseline**  **Fold**  **Change P** | | **d7 vs Baseline**  **Fold**  **Change P** | |
| --- | --- | --- | --- | --- | --- | --- | --- | --- | --- | --- |
| A_70_P028011 | NUF2 | NUF2, NDC80 kinetochore complex component, homolog (S. cerevisia | -1.55 | 0.009 | 2.70 | 0.000 | 2.69 | 0.000 | 1.51 | 0.013 |
| A_70_P028026 | EIF6 | eukaryotic translation initiation factor 6 | 1.11 | 0.659 | 1.41 | 0.163 | 3.54 | 0.000 | 2.94 | 0.001 |
| A_70_P028061 | SPHK1 | sphingosine kinase 1 | 2.43 | 0.002 | 2.20 | 0.005 | 2.43 | 0.002 | 1.97 | 0.012 |
| A_70_P028096 | TRAF3IP3 | TRAF3 interacting protein 3 | -2.16 | 0.001 | -1.44 | 0.078 | -1.07 | 0.727 | 1.28 | 0.219 |
| A_70_P028111 | BUB1 | budding uninhibited by benzimidazoles 1 homolog (yeast) | -1.18 | 0.094 | 2.17 | 0.000 | 2.15 | 0.000 | 1.43 | 0.002 |
| A_70_P028191 | CKAP2L | cytoskeleton associated protein 2-like | -1.69 | 0.004 | 2.44 | 0.000 | 2.55 | 0.000 | 1.55 | 0.010 |
| A_70_P028241 | C12orf24 | chromosome 12 open reading frame 24 | -2.00 | 0.005 | -1.52 | 0.058 | -1.21 | 0.360 | -1.35 | 0.160 |
| A_70_P028261 | ABHD4 | abhydrolase domain containing 4 | -2.07 | 0.001 | -1.55 | 0.028 | -1.18 | 0.376 | 1.18 | 0.362 |
| A_70_P028306 | LMNB1 | lamin B1 | -1.23 | 0.213 | 3.11 | 0.000 | 2.17 | 0.000 | 1.58 | 0.013 |
| A_70_P028307 | LMNB1 | lamin B1 | -1.26 | 0.217 | 3.18 | 0.000 | 2.28 | 0.001 | 1.64 | 0.018 |
| A_70_P028341 | SPCS3 | signal peptidase complex subunit 3 homolog (S.cerevisiae) | 1.51 | 0.040 | 2.04 | 0.002 | 1.79 | 0.007 | 1.01 | 0.964 |
| A_70_P028386 | AURKB | aurora kinase B | -1.84 | 0.000 | 6.83 | 0.000 | 6.50 | 0.000 | 2.67 | 0.000 |
| A_70_P028446 | ASF1B | ASF1 anti-silencing function 1 homolog B | -1.18 | 0.262 | 5.69 | 0.000 | 4.27 | 0.000 | 1.81 | 0.001 |
| A_70_P028486 | NEK2 | NIMA (never in mitosis gene a)-related kinase 2 | -1.20 | 0.623 | 2.49 | 0.026 | 3.69 | 0.003 | 1.63 | 0.200 |
| A_70_P028491 | ACOT7 | acyl-CoA thioesterase 7 | 1.53 | 0.043 | 2.79 | 0.000 | 2.26 | 0.001 | 1.88 | 0.006 |
| A_70_P028546 | ISYNA1 | inositol-3-phosphate synthase 1 | -2.38 | 0.007 | -1.32 | 0.322 | -1.26 | 0.399 | -1.76 | 0.055 |
| A_70_P028616 | IGFBP3 | insulin-like growth factor binding protein 3 | 1.39 | 0.063 | 1.96 | 0.001 | 2.20 | 0.000 | 2.54 | 0.000 |
| A_70_P028681 | TBXAS1 | thromboxane A synthase 1 (platelet) | -2.31 | 0.045 | -1.12 | 0.759 | -1.45 | 0.340 | 1.58 | 0.243 |
| A_70_P028696 | Mynn | myoneurin | 1.52 | 0.027 | 2.07 | 0.001 | 1.53 | 0.025 | 1.04 | 0.808 |

| **Probenames** | **Gene Symbol** | **Gene Name** | **6h vs Baseline**  **Fold**  **Change P** | | **d1 vs Baseline**  **Fold**  **Change P** | | **d3 vs Baseline**  **Fold**  **Change P** | | **d7 vs Baseline**  **Fold**  **Change P** | |
| --- | --- | --- | --- | --- | --- | --- | --- | --- | --- | --- |
| A_70_P028801 | NUMA1 | nuclear mitotic apparatus protein 1 | -2.06 | 0.001 | -1.42 | 0.053 | -1.19 | 0.311 | -1.35 | 0.094 |
| A_70_P028841 | GRRP1 | glycine/arginine rich protein 1 | 1.53 | 0.065 | 2.06 | 0.005 | 1.42 | 0.123 | 1.31 | 0.224 |
| A_70_P028906 | AC104852.1 | NULL | -1.33 | 0.067 | 3.09 | 0.000 | 3.19 | 0.000 | 1.77 | 0.002 |
| A_70_P028931 | LOC781493 | similar to Collagen alpha-1(XIV) chain precursor (Undulin) | -1.01 | 0.963 | 1.04 | 0.864 | 2.48 | 0.003 | 2.15 | 0.009 |
| A_70_P029186 | TEAD4 | TEA domain family member 4 | 1.57 | 0.024 | 2.29 | 0.000 | 1.80 | 0.006 | 1.51 | 0.037 |
| A_70_P029341 | CPXM1 | carboxypeptidase X (M14 family), member 1 | 2.28 | 0.008 | 3.27 | 0.001 | 2.73 | 0.002 | 2.01 | 0.020 |
| A_70_P029376 | P4HB | prolyl 4-hydroxylase, beta polypeptide | 1.43 | 0.144 | 2.01 | 0.010 | 1.22 | 0.397 | 1.22 | 0.390 |
| A_70_P029411 | MXRA8 | matrix-remodelling associated 8 | 1.41 | 0.243 | 2.30 | 0.012 | 2.34 | 0.010 | 1.65 | 0.101 |
| A_70_P029426 | EIF4EBP1 | eukaryotic translation initiation factor 4E binding protein 1 | 1.40 | 0.225 | 2.13 | 0.014 | 2.22 | 0.010 | -1.04 | 0.889 |
| A_70_P029481 | CEBPD | CCAAT/enhancer binding protein (C/EBP), delta | 2.18 | 0.002 | 1.64 | 0.028 | -1.12 | 0.565 | -1.52 | 0.056 |
| A_70_P029501 | CRTAP | cartilage associated protein | -1.29 | 0.323 | 1.43 | 0.174 | 2.48 | 0.003 | 2.12 | 0.010 |
| A_70_P029511 | TMEM119 | transmembrane protein 119 | -1.33 | 0.193 | 1.47 | 0.089 | 5.32 | 0.000 | 6.02 | 0.000 |
| A_70_P029571 | RASGRP3 | RAS guanyl releasing protein 3 (calcium and DAG-regulated) | -2.36 | 0.002 | -1.36 | 0.183 | -1.34 | 0.207 | 1.16 | 0.517 |
| A_70_P029596 | ITGA5 | integrin, alpha 5 (fibronectin receptor, alpha polypeptide) | 1.60 | 0.105 | 2.17 | 0.014 | 2.81 | 0.002 | 2.17 | 0.014 |
| A_70_P029611 | CTHRC1 | collagen triple helix repeat containing 1 | -1.16 | 0.689 | 1.08 | 0.835 | 2.43 | 0.028 | 6.42 | 0.000 |
| A_70_P029656 | RANBP1 | RAN binding protein 1 | 1.52 | 0.033 | 2.12 | 0.001 | 1.56 | 0.025 | -1.19 | 0.342 |
| A_70_P029666 | BLVRB | biliverdin reductase B (flavin reductase (NADPH)) | -2.15 | 0.001 | -1.09 | 0.631 | -1.05 | 0.785 | -1.77 | 0.007 |

| **Probenames** | **Gene Symbol** | **Gene Name** | **6h vs Baseline**  **Fold**  **Change P** | | **d1 vs Baseline**  **Fold**  **Change P** | | **d3 vs Baseline**  **Fold**  **Change P** | | **d7 vs Baseline**  **Fold**  **Change P** | |
| --- | --- | --- | --- | --- | --- | --- | --- | --- | --- | --- |
| A_70_P029686 | TK1 | thymidine kinase 1, soluble | -1.22 | 0.123 | 5.68 | 0.000 | 3.25 | 0.000 | 1.84 | 0.000 |
| A_70_P029691 | SPSB3 | splA/ryanodine receptor domain and SOCS box containing 3 | -2.07 | 0.000 | -1.69 | 0.004 | -1.95 | 0.001 | -1.46 | 0.027 |
| A_70_P029716 | FBLN2 | fibulin 2 | -1.49 | 0.111 | -1.03 | 0.897 | 1.90 | 0.017 | 2.37 | 0.003 |
| A_70_P029731 | OBTP | overexpressed breast tumor protein homolog | 3.02 | 0.000 | 3.30 | 0.000 | 1.70 | 0.003 | 1.03 | 0.850 |
| A_70_P029746 | GSTA4 | glutathione S-transferase alpha 4 | -2.80 | 0.000 | -1.62 | 0.011 | 1.05 | 0.768 | -1.04 | 0.812 |
| A_70_P029796 | TGFBI | transforming growth factor, beta-induced, 68kDa | -1.35 | 0.333 | -1.16 | 0.631 | 3.30 | 0.002 | 8.14 | 0.000 |
| A_70_P029806 | CMTM3 | CKLF-like MARVEL transmembrane domain containing 3 | -1.39 | 0.060 | 1.17 | 0.346 | 2.05 | 0.001 | 2.29 | 0.000 |
| A_70_P029836 | MRC2 | mannose receptor, C type 2 | -1.34 | 0.114 | -1.33 | 0.121 | 2.48 | 0.000 | 2.05 | 0.001 |
| A_70_P029861 | CAPG | capping protein (actin filament), gelsolin-like | 1.07 | 0.774 | 1.79 | 0.028 | 2.62 | 0.001 | 1.94 | 0.015 |
| A_70_P029886 | CYP4V2 | cytochrome P450, family 4, subfamily V, polypeptide 2 | -2.19 | 0.001 | -1.68 | 0.014 | -1.21 | 0.314 | 1.02 | 0.914 |
| A_70_P029896 | MMP19 | matrix metallopeptidase 19 | -3.36 | 0.005 | -1.70 | 0.159 | 1.15 | 0.697 | 1.79 | 0.123 |
| A_70_P029911 | SDS | serine dehydratase | 1.47 | 0.451 | 1.74 | 0.281 | 3.02 | 0.044 | 3.02 | 0.043 |
| A_70_P029921 | LPL | lipoprotein lipase | -2.20 | 0.001 | -2.23 | 0.001 | -1.81 | 0.004 | 1.01 | 0.971 |
| A_70_P029922 | LPL | lipoprotein lipase | -2.14 | 0.001 | -2.26 | 0.001 | -1.82 | 0.005 | 1.02 | 0.895 |
| A_70_P029966 | PDXK | pyridoxal (pyridoxine, vitamin B6) kinase" | 2.16 | 0.001 | 2.77 | 0.000 | 2.67 | 0.000 | 1.23 | 0.294 |
| A_70_P030001 | ITLN2 | intelectin 2 | -1.89 | 0.160 | -1.50 | 0.358 | -2.95 | 0.026 | -1.97 | 0.135 |
| A_70_P030011 | ABCB1 | ATP-binding cassette, sub-family B (MDR/TAP), member 1" | -3.06 | 0.000 | -1.86 | 0.015 | -1.43 | 0.132 | -1.48 | 0.101 |

| **Probenames** | **Gene Symbol** | **Gene Name** | **6h vs Baseline**  **Fold**  **Change P** | | **d1 vs Baseline**  **Fold**  **Change P** | | **d3 vs Baseline**  **Fold**  **Change P** | | **d7 vs Baseline**  **Fold**  **Change P** | |
| --- | --- | --- | --- | --- | --- | --- | --- | --- | --- | --- |
| A_70_P030012 | ABCB1 | ATP-binding cassette, sub-family B (MDR/TAP), member 1" | -2.52 | 0.000 | -1.75 | 0.011 | -1.29 | 0.198 | -1.41 | 0.095 |
| A_70_P030056 | C1orf116 | chromosome 1 open reading frame 116 | -2.22 | 0.047 | -1.09 | 0.814 | -1.36 | 0.406 | 1.24 | 0.557 |
| A_70_P030086 | GJB2 | gap junction protein, beta 2, 26kDa | 1.53 | 0.182 | 2.53 | 0.009 | 4.35 | 0.000 | 3.36 | 0.002 |
| A_70_P030087 | GJB2 | gap junction protein, beta 2, 26kDa | 1.54 | 0.171 | 2.46 | 0.010 | 4.27 | 0.000 | 3.45 | 0.001 |
| A_70_P030096 | AGTR1 | angiotensin II receptor, type 1 | -5.77 | 0.000 | -4.00 | 0.001 | -2.18 | 0.022 | -1.45 | 0.234 |
| A_70_P030107 | CPT1B | carnitine palmitoyltransferase 1B (muscle) | -2.12 | 0.026 | -1.46 | 0.223 | -1.99 | 0.039 | -1.18 | 0.580 |
| A_70_P030112 | IL15 | interleukin 15 | -2.05 | 0.001 | -1.56 | 0.013 | 1.00 | 0.995 | 1.33 | 0.091 |
| A_70_P030126 | CP | ceruloplasmin (ferroxidase) | 6.17 | 0.001 | 24.16 | 0.000 | 13.98 | 0.000 | 3.77 | 0.006 |
| A_70_P030127 | CP | ceruloplasmin (ferroxidase) | 8.35 | 0.000 | 32.22 | 0.000 | 19.14 | 0.000 | 4.86 | 0.004 |
| A_70_P030146 | PRND | prion protein 2 (dublet) | -1.20 | 0.286 | 1.08 | 0.655 | 1.94 | 0.002 | 2.30 | 0.000 |
| A_70_P030151 | AQP5 | aquaporin 5 | 1.25 | 0.329 | 1.24 | 0.345 | -1.38 | 0.166 | -2.42 | 0.002 |
| A_70_P030152 | AQP5 | aquaporin 5 | 1.20 | 0.427 | 1.20 | 0.412 | -1.40 | 0.151 | -2.43 | 0.002 |
| A_70_P030171 | ITGB2 | integrin, beta 2 (complement component 3 receptor 3 and 4 subuni | -2.76 | 0.014 | -1.47 | 0.294 | -1.33 | 0.428 | 1.43 | 0.332 |
| A_70_P030172 | ITGB2 | integrin, beta 2 (complement component 3 receptor 3 and 4 subuni | -2.73 | 0.014 | -1.44 | 0.311 | -1.29 | 0.476 | 1.38 | 0.369 |
| A_70_P030181 | SLPI | secretory leukocyte peptidase inhibitor | -1.10 | 0.640 | 2.13 | 0.002 | 1.32 | 0.174 | 1.66 | 0.020 |
| A_70_P030182 | SLPI | secretory leukocyte peptidase inhibitor | -1.02 | 0.927 | 2.28 | 0.002 | 1.26 | 0.289 | 1.76 | 0.019 |
| A_70_P030191 | TTR | transthyretin | -2.43 | 0.005 | -1.72 | 0.056 | -2.02 | 0.018 | -1.34 | 0.275 |
| A_70_P030231 | TAC1 | tachykinin, precursor 1 | -1.09 | 0.698 | -2.48 | 0.001 | -1.73 | 0.027 | -1.26 | 0.302 |
| A_70_P030232 | TAC1 | tachykinin, precursor 1 | -1.18 | 0.493 | -2.77 | 0.001 | -1.84 | 0.023 | -1.33 | 0.250 |
| A_70_P030261 | LOC100037688 | kappa light chain | -1.66 | 0.099 | 1.19 | 0.550 | 2.68 | 0.005 | 1.51 | 0.169 |

| **Probenames** | **Gene Symbol** | **Gene Name** | **6h vs Baseline**  **Fold**  **Change P** | | **d1 vs Baseline**  **Fold**  **Change P** | | **d3 vs Baseline**  **Fold**  **Change P** | | **d7 vs Baseline**  **Fold**  **Change P** | |
| --- | --- | --- | --- | --- | --- | --- | --- | --- | --- | --- |
| A_70_P030262 | LOC100037688 | kappa light chain | -1.54 | 0.148 | 1.22 | 0.494 | 2.56 | 0.006 | 1.49 | 0.184 |
| A_70_P030336 | CYP2J | cytochrome P450, family 2, subfamily J" | -2.05 | 0.003 | -1.43 | 0.091 | -1.58 | 0.038 | -1.56 | 0.042 |
| A_70_P030339 | CYP2J | cytochrome P450, family 2, subfamily J" | -2.21 | 0.004 | -1.52 | 0.080 | -1.80 | 0.020 | -1.69 | 0.034 |
| A_70_P030341 | CD86 | CD86 molecule | -1.04 | 0.906 | 1.39 | 0.350 | 2.26 | 0.033 | 1.47 | 0.278 |
| A_70_P030361 | PAEP | progestagen-associated endometrial protein | 6.77 | 0.003 | 15.18 | 0.000 | 15.64 | 0.000 | 3.47 | 0.031 |
| A_70_P030367 | ATP5G1 | ATP synthase, H+ transporting, mitochondrial Fo complex, subunit | 1.23 | 0.329 | 1.28 | 0.239 | 1.20 | 0.378 | -2.00 | 0.005 |
| A_70_P030376 | UGT1A1 | UDP glucuronosyltransferase 1 family, polypeptide A1 | -10.10 | 0.005 | -7.10 | 0.012 | -9.15 | 0.006 | -2.30 | 0.235 |
| A_70_P030377 | UGT1A1 | UDP glucuronosyltransferase 1 family, polypeptide A1 | -10.26 | 0.004 | -7.50 | 0.009 | -9.94 | 0.004 | -2.21 | 0.248 |
| A_70_P030386 | TTP | tristetraprolin | 2.12 | 0.011 | 1.42 | 0.186 | -1.06 | 0.829 | 1.02 | 0.951 |
| A_70_P030387 | TTP | tristetraprolin | 2.25 | 0.001 | 1.47 | 0.060 | 1.21 | 0.336 | -1.13 | 0.531 |
| A_70_P030396 | ITGAL | integrin, alpha L (antigen CD11A (p180), lymphocyte function-ass | -2.06 | 0.014 | -1.81 | 0.036 | -1.77 | 0.043 | 1.32 | 0.291 |
| A_70_P030397 | ITGAL | integrin, alpha L (antigen CD11A (p180), lymphocyte function-ass | -2.01 | 0.014 | -1.83 | 0.028 | -1.77 | 0.037 | 1.31 | 0.289 |
| A_70_P030411 | MYC | v-myc myelocytomatosis viral oncogene homolog (avian) | 2.49 | 0.001 | 2.09 | 0.003 | 1.56 | 0.045 | 1.18 | 0.420 |
| A_70_P030412 | MYC | v-myc myelocytomatosis viral oncogene homolog (avian) | 2.62 | 0.000 | 2.30 | 0.001 | 1.81 | 0.008 | 1.32 | 0.161 |
| A_70_P030446 | HMMR | hyaluronan-mediated motility receptor (RHAMM) | -1.21 | 0.212 | 2.72 | 0.000 | 2.77 | 0.000 | 1.86 | 0.001 |

| **Probenames** | **Gene Symbol** | **Gene Name** | **6h vs Baseline**  **Fold**  **Change P** | | **d1 vs Baseline**  **Fold**  **Change P** | | **d3 vs Baseline**  **Fold**  **Change P** | | **d7 vs Baseline**  **Fold**  **Change P** | |
| --- | --- | --- | --- | --- | --- | --- | --- | --- | --- | --- |
| A_70_P030506 | UPP1 | uridine phosphorylase 1 | 6.85 | 0.000 | 8.73 | 0.000 | 7.39 | 0.000 | 3.40 | 0.005 |
| A_70_P030596 | MFAP4 | microfibrillar-associated protein 4 | -2.22 | 0.015 | -3.72 | 0.001 | -1.55 | 0.148 | 1.06 | 0.832 |
| A_70_P030616 | ESAM | endothelial cell adhesion molecule | -2.40 | 0.001 | -1.42 | 0.098 | -1.14 | 0.504 | 1.32 | 0.183 |
| A_70_P030636 | ANGPTL4 | angiopoietin-like 4 | 1.73 | 0.027 | 2.13 | 0.005 | 1.27 | 0.299 | 1.24 | 0.345 |
| A_70_P030666 | HEYL | hairy/enhancer-of-split related with YRPW motif-like | -3.20 | 0.000 | -2.23 | 0.001 | -1.30 | 0.176 | 1.04 | 0.837 |
| A_70_P030736 | GPX7 | glutathione peroxidase 7 | 1.05 | 0.829 | 1.86 | 0.010 | 4.07 | 0.000 | 4.14 | 0.000 |
| A_70_P030776 | SFRP2 | secreted frizzled-related protein 2 | -1.61 | 0.249 | -1.49 | 0.331 | 2.43 | 0.043 | 1.48 | 0.336 |
| A_70_P030790 | CD93 | CD93 molecule | -2.28 | 0.005 | 1.36 | 0.220 | 1.45 | 0.146 | 1.67 | 0.052 |
| A_70_P030811 | PLVAP | plasmalemma vesicle associated protein | -1.59 | 0.132 | 1.54 | 0.153 | 2.45 | 0.008 | 2.45 | 0.008 |
| A_70_P030831 | HOMER3 | homer homolog 3 (Drosophila) | -2.10 | 0.001 | -1.02 | 0.914 | 1.19 | 0.317 | 1.60 | 0.016 |
| A_70_P030871 | MCM5 | minichromosome maintenance complex component 5 | -1.44 | 0.060 | 2.97 | 0.000 | 1.41 | 0.074 | 1.18 | 0.367 |
| A_70_P030886 | ACP5 | acid phosphatase 5, tartrate resistant | -2.45 | 0.001 | -1.64 | 0.028 | -1.48 | 0.071 | -1.10 | 0.632 |
| A_70_P030896 | FAM125A | family with sequence similarity 125, member A | -2.12 | 0.000 | -1.22 | 0.134 | -1.43 | 0.013 | -1.72 | 0.001 |
| A_70_P030971 | HLA-DMB | major histocompatibility complex, class II, DM beta | -2.67 | 0.001 | -2.33 | 0.004 | -2.42 | 0.003 | -1.34 | 0.250 |
| A_70_P030996 | COL18A1 | collagen, type XVIII, alpha 1 | -1.57 | 0.129 | 1.04 | 0.899 | 2.37 | 0.009 | 1.99 | 0.028 |
| A_70_P031011 | HM13 | histocompatibility (minor) 13 | 1.44 | 0.118 | 2.03 | 0.006 | 1.71 | 0.028 | 1.24 | 0.344 |
| A_70_P031231 | TGFB3 | transforming growth factor, beta 3 | -1.04 | 0.886 | -1.01 | 0.974 | 1.42 | 0.186 | 2.44 | 0.004 |
| A_70_P031232 | TGFB3 | transforming growth factor, beta 3 | -1.06 | 0.818 | -1.02 | 0.937 | 1.41 | 0.189 | 2.33 | 0.005 |

| **Probenames** | **Gene Symbol** | **Gene Name** | **6h vs Baseline**  **Fold**  **Change P** | | **d1 vs Baseline**  **Fold**  **Change P** | | **d3 vs Baseline**  **Fold**  **Change P** | | **d7 vs Baseline**  **Fold**  **Change P** | |
| --- | --- | --- | --- | --- | --- | --- | --- | --- | --- | --- |
| A_70_P031411 | SERPINH1 | serpin peptidase inhibitor, clade H (heat shock protein 47), mem | 1.03 | 0.872 | 2.09 | 0.001 | 1.90 | 0.002 | 2.42 | 0.000 |
| A_70_P031436 | H1F0 | H1 histone family, member 0 | -2.46 | 0.001 | -1.03 | 0.898 | 1.39 | 0.148 | 1.08 | 0.722 |
| A_70_P031481 | LASP1 | LIM and SH3 protein 1 | 1.51 | 0.490 | 1.82 | 0.319 | 3.64 | 0.045 | -1.06 | 0.916 |
| A_70_P031486 | AC079061.1 | NULL | -2.05 | 0.014 | -1.85 | 0.029 | -2.24 | 0.007 | -2.02 | 0.015 |
| A_70_P031521 | HOPX | HOP homeobox (HOPX) | -3.60 | 0.001 | -2.51 | 0.008 | -3.57 | 0.001 | -2.09 | 0.026 |
| A_70_P031611 | PLP1 | proteolipid protein 1 | -2.00 | 0.006 | -2.62 | 0.001 | -3.46 | 0.000 | -1.61 | 0.040 |
| A_70_P031751 | DAPP1 | dual adaptor of phosphotyrosine and 3-phosphoinositides | -2.03 | 0.000 | -1.33 | 0.047 | 1.16 | 0.275 | 1.14 | 0.329 |
| A_70_P031756 | YPEL3 | yippee-like 3 (Drosophila) | -2.39 | 0.001 | -1.78 | 0.009 | -1.14 | 0.500 | -1.51 | 0.048 |
| A_70_P031861 | THBD | thrombomodulin | 2.00 | 0.013 | 1.14 | 0.599 | -1.17 | 0.524 | -1.08 | 0.758 |
| A_70_P031976 | LYZ1 | lysozyme 1 | 2.72 | 0.005 | 3.20 | 0.002 | 3.30 | 0.002 | 1.82 | 0.065 |
| A_70_P032021 | MGST1 | microsomal glutathione S-transferase 1 | -2.47 | 0.001 | -1.51 | 0.052 | -1.60 | 0.030 | -1.81 | 0.009 |
| A_70_P032026 | KLHL24 | kelch-like 24 (Drosophila) | -1.84 | 0.014 | -2.15 | 0.004 | -1.85 | 0.013 | -1.30 | 0.240 |
| A_70_P032086 | GPATCH4 | G patch domain containing 4 | 2.09 | 0.000 | 1.88 | 0.001 | -1.13 | 0.377 | -1.30 | 0.079 |
| A_70_P032211 | PANK1 | pantothenate kinase 1 | -2.37 | 0.001 | -1.14 | 0.514 | -1.24 | 0.286 | -1.52 | 0.051 |
| A_70_P032216 | GABBR1 | gamma-aminobutyric acid (GABA) B receptor, 1 | -1.78 | 0.006 | -1.70 | 0.010 | -2.11 | 0.001 | -2.00 | 0.002 |
| A_70_P032271 | DLGAP5 | discs, large (Drosophila) homolog-associated protein 5 | 2.23 | 0.141 | 4.51 | 0.012 | 6.51 | 0.003 | 1.34 | 0.574 |
| A_70_P032296 | PKM2 | pyruvate kinase, muscle | 1.31 | 0.374 | 2.40 | 0.011 | 3.33 | 0.001 | 1.87 | 0.054 |
| A_70_P032311 | C4orf31 | chromosome 4 open reading frame 31 | -4.51 | 0.002 | -4.29 | 0.003 | -4.24 | 0.003 | -1.42 | 0.388 |

| **Probenames** | **Gene Symbol** | **Gene Name** | **6h vs Baseline**  **Fold**  **Change P** | | **d1 vs Baseline**  **Fold**  **Change P** | | **d3 vs Baseline**  **Fold**  **Change P** | | **d7 vs Baseline**  **Fold**  **Change P** | |
| --- | --- | --- | --- | --- | --- | --- | --- | --- | --- | --- |
| A_70_P032341 | HMMR | hyaluronan-mediated motility receptor (RHAMM) | -1.90 | 0.000 | 4.53 | 0.000 | 5.38 | 0.000 | 2.31 | 0.000 |
| A_70_P032371 | COL6A3 | collagen, type VI, alpha 3 | -1.00 | 0.997 | 1.34 | 0.203 | 3.15 | 0.000 | 3.52 | 0.000 |
| A_70_P032386 | SEC24D | SEC24 family, member D (S. cerevisiae) | 1.70 | 0.014 | 2.28 | 0.001 | 1.74 | 0.011 | 1.53 | 0.041 |
| A_70_P032476 | MFAP5 | microfibrillar associated protein 5 | 1.18 | 0.629 | -1.08 | 0.827 | 2.31 | 0.030 | 2.08 | 0.052 |
| A_70_P032596 | EMP1 | similar to epithelial membrane protein 1 | 2.62 | 0.002 | 1.76 | 0.037 | 1.80 | 0.031 | 1.61 | 0.071 |
| A_70_P032631 | C5orf13 | chromosome 5 open reading frame 13 | -2.75 | 0.000 | -2.09 | 0.002 | 1.56 | 0.030 | 2.03 | 0.002 |
| A_70_P032816 | SLC25A5 | solute carrier family 25 (mitochondrial carrier; adenine nucleotide translocator), member 5" | 1.99 | 0.001 | 2.34 | 0.000 | 1.81 | 0.004 | 1.07 | 0.677 |
| A_70_P033121 | C16orf5 | chromosome 16 open reading frame 5 | -2.27 | 0.000 | -1.70 | 0.004 | -1.72 | 0.003 | -1.30 | 0.104 |
| A_70_P033126 | FXYD1 | FXYD domain containing ion transport regulator 1 | -2.00 | 0.002 | -2.18 | 0.001 | -1.32 | 0.145 | -1.97 | 0.002 |
| A_70_P033141 | SLC25A37 | solute carrier family 25, member 37 | -1.73 | 0.030 | -1.61 | 0.054 | -2.05 | 0.007 | -1.22 | 0.392 |
| A_70_P033241 | C12orf44 | chromosome 12 open reading frame 44 | -1.72 | 0.043 | -1.46 | 0.137 | -2.00 | 0.013 | -2.00 | 0.013 |
| A_70_P033311 | SLC26A11 | solute carrier family 26, member 11 | -2.15 | 0.001 | -1.90 | 0.002 | -1.97 | 0.001 | -1.32 | 0.114 |
| A_70_P033331 | ZRANB3 | zinc finger, RAN-binding domain containing 3 | -2.21 | 0.001 | -1.24 | 0.225 | -1.50 | 0.033 | -1.14 | 0.438 |
| A_70_P033336 | S100A12 | S100 calcium binding protein A12 | 2.59 | 0.003 | 2.60 | 0.003 | 4.64 | 0.000 | 1.09 | 0.733 |
| A_70_P033351 | RB1 | retinoblastoma 1 | -3.19 | 0.000 | -1.92 | 0.009 | -1.89 | 0.010 | -1.07 | 0.745 |
| A_70_P033401 | KDELR1 | KDEL (Lys-Asp-Glu-Leu) endoplasmic reticulum protein retention r | -1.07 | 0.710 | 1.59 | 0.018 | 2.01 | 0.001 | 1.24 | 0.228 |
| A_70_P033441 | MEST | mesoderm specific transcript homolog (mouse) | -1.65 | 0.027 | -1.61 | 0.034 | -1.04 | 0.831 | 2.68 | 0.000 |
| A_70_P033476 | NHP2 | NHP2 ribonucleoprotein homolog (yeast) | 2.23 | 0.003 | 2.52 | 0.001 | 1.77 | 0.024 | -1.25 | 0.328 |

| **Probenames** | **Gene Symbol** | **Gene Name** | **6h vs Baseline**  **Fold**  **Change P** | | **d1 vs Baseline**  **Fold**  **Change P** | | **d3 vs Baseline**  **Fold**  **Change P** | | **d7 vs Baseline**  **Fold**  **Change P** | |
| --- | --- | --- | --- | --- | --- | --- | --- | --- | --- | --- |
| A_70_P033491 | EZH2 | enhancer of zeste homolog 2 (Drosophila) | 1.07 | 0.697 | 2.62 | 0.000 | 2.09 | 0.001 | 1.50 | 0.025 |
| A_70_P033571 | SYDE1 | synapse defective 1, Rho GTPase, homolog 1 (C. elegans) | 1.91 | 0.112 | 2.00 | 0.092 | 2.52 | 0.031 | -1.35 | 0.448 |
| A_70_P033626 | SLC23A2 | solute carrier family 23 (nucleobase transporters), member 2 | -3.22 | 0.000 | -2.58 | 0.002 | -4.12 | 0.000 | -2.20 | 0.007 |
| A_70_P033681 | SPRR1B | small proline-rich protein 1B | 2.53 | 0.032 | 1.60 | 0.243 | 3.24 | 0.010 | 1.22 | 0.609 |
| A_70_P033686 | KRT17 | keratin 17 | 1.05 | 0.823 | 1.73 | 0.033 | 2.49 | 0.002 | 1.58 | 0.070 |
| A_70_P033696 | TIMM9 | translocase of inner mitochondrial membrane 9 homolog (yeast) | 1.53 | 0.020 | 2.05 | 0.001 | 1.65 | 0.008 | -1.05 | 0.768 |
| A_70_P033761 | POSTN | periostin, osteoblast specific factor | 1.03 | 0.890 | 1.27 | 0.232 | 4.44 | 0.000 | 6.21 | 0.000 |
| A_70_P034351 | CCNA2 | cyclin A2 | -2.06 | 0.001 | 4.54 | 0.000 | 3.82 | 0.000 | 1.93 | 0.002 |
| A_70_P034381 | CXCR7 | chemokine (C-X-C motif) receptor 7 | -1.06 | 0.813 | -1.13 | 0.610 | 2.42 | 0.002 | 2.44 | 0.002 |
| A_70_P034406 | INPP5B | inositol polyphosphate-5-phosphatase, 75kDa | -2.20 | 0.001 | -1.51 | 0.049 | -1.53 | 0.042 | -1.15 | 0.461 |
| A_70_P034456 | MED25 | mediator complex subunit 25 | -2.26 | 0.010 | -1.64 | 0.085 | -1.78 | 0.051 | -1.28 | 0.368 |
| A_70_P034521 | TOMM5 | translocase of outer mitochondrial membrane 5 homolog (yeast) | 1.26 | 0.286 | 2.11 | 0.004 | 2.26 | 0.002 | 1.00 | 0.983 |
| A_70_P034531 | CENP-E | Centromeric protein E (CENP-E) | -1.24 | 0.091 | 3.92 | 0.000 | 5.08 | 0.000 | 2.40 | 0.000 |
| A_70_P034691 | IMPA2 | inositol(myo)-1(or 4)-monophosphatase 2 | -1.22 | 0.213 | 1.81 | 0.002 | 2.35 | 0.000 | -1.03 | 0.851 |
| A_70_P034696 | SMARCA2 | SWI/SNF related, matrix associated, actin dependent regulator of | -2.66 | 0.000 | -1.83 | 0.005 | -1.48 | 0.045 | -1.47 | 0.048 |
| A_70_P034706 | TSPYL5 | TSPY-like 5 | -2.24 | 0.002 | -1.58 | 0.046 | -1.76 | 0.018 | -1.33 | 0.192 |
| A_70_P034751 | STX7 | syntaxin 7 | -2.10 | 0.006 | -1.47 | 0.107 | -1.53 | 0.080 | -1.12 | 0.620 |
| A_70_P034821 | SLC25A37 | solute carrier family 25, member 37 | -1.91 | 0.012 | -1.60 | 0.052 | -2.04 | 0.007 | -1.30 | 0.247 |

| **Probenames** | **Gene Symbol** | **Gene Name** | **6h vs Baseline**  **Fold**  **Change P** | | **d1 vs Baseline**  **Fold**  **Change P** | | **d3 vs Baseline**  **Fold**  **Change P** | | **d7 vs Baseline**  **Fold**  **Change P** | |
| --- | --- | --- | --- | --- | --- | --- | --- | --- | --- | --- |
| A_70_P034931 | SERF1B | small EDRK-rich factor 1A (telomeric) | -1.04 | 0.858 | 1.42 | 0.092 | 2.30 | 0.001 | 1.12 | 0.559 |
| A_70_P035036 | PPRC1 | peroxisome proliferator-activated receptor gamma, coactivator-re | 2.02 | 0.002 | 1.90 | 0.003 | 1.07 | 0.715 | 1.01 | 0.968 |
| A_70_P035126 | CDC6 | cell division cycle 6 homolog (S. cerevisiae) | -1.15 | 0.510 | 9.84 | 0.000 | 4.82 | 0.000 | 2.90 | 0.000 |
| A_70_P035131 | BATF3 | basic leucine zipper transcription factor, ATF-like 3 | 2.96 | 0.001 | 2.43 | 0.003 | 1.85 | 0.026 | 1.23 | 0.404 |
| A_70_P035141 |  | septin 6 (SEPT6) | 1.15 | 0.609 | 2.09 | 0.019 | 1.60 | 0.110 | -1.12 | 0.688 |
| A_70_P035241 | TRA2A | transformer 2 alpha homolog (Drosophila) | -2.15 | 0.001 | -2.08 | 0.002 | -1.85 | 0.005 | -1.38 | 0.100 |
| A_70_P035341 | ZNF362 | zinc finger protein 362 | -2.74 | 0.000 | -2.06 | 0.005 | -1.30 | 0.244 | -1.32 | 0.219 |
| A_70_P035536 | ANKRD1 | ankyrin repeat domain 1 (cardiac muscle) | 7.31 | 0.000 | 2.82 | 0.009 | -1.14 | 0.691 | 1.17 | 0.652 |
| A_70_P035556 | PDIA5 | protein disulfide isomerase family A, member 5 | 1.52 | 0.045 | 2.28 | 0.001 | 1.91 | 0.005 | 1.89 | 0.005 |
| A_70_P035601 | CLEC4A | C-type lectin domain family 4, member A | -1.10 | 0.691 | 1.31 | 0.284 | 1.97 | 0.016 | 2.07 | 0.011 |
| A_70_P035611 | SELM | NULL | 1.44 | 0.195 | 2.11 | 0.016 | 1.74 | 0.061 | -1.15 | 0.613 |
| A_70_P035686 | GALE | UDP-galactose-4-epimerase | 1.91 | 0.016 | 2.41 | 0.002 | 1.21 | 0.424 | -1.08 | 0.743 |
| A_70_P035841 | DDX5 | DEAD (Asp-Glu-Ala-Asp) box polypeptide 5 | 1.38 | 0.283 | 1.46 | 0.215 | 2.57 | 0.007 | 1.07 | 0.808 |
| A_70_P035871 | SERPINE1 | serpin peptidase inhibitor, clade E (nexin, plasminogen activato | 4.75 | 0.000 | 2.09 | 0.023 | 3.35 | 0.001 | 3.32 | 0.001 |
| A_70_P035891 | SNX10 | sorting nexin 10 | -1.80 | 0.077 | -1.72 | 0.100 | -2.22 | 0.023 | 1.27 | 0.443 |
| A_70_P035901 | ACSF2 | acyl-CoA synthetase family member 2 | -2.32 | 0.000 | -1.53 | 0.029 | -1.27 | 0.188 | -1.23 | 0.246 |
| A_70_P035981 | TREM2 | triggering receptor expressed on myeloid cells 2 | -1.12 | 0.390 | 1.16 | 0.279 | 2.11 | 0.000 | 1.39 | 0.025 |
| A_70_P035986 | SFRP4 | secreted frizzled-related protein 4 | 1.19 | 0.567 | -1.19 | 0.569 | 2.16 | 0.025 | 2.51 | 0.010 |

| **Probenames** | **Gene Symbol** | **Gene Name** | **6h vs Baseline**  **Fold**  **Change P** | | **d1 vs Baseline**  **Fold**  **Change P** | | **d3 vs Baseline**  **Fold**  **Change P** | | **d7 vs Baseline**  **Fold**  **Change P** | |
| --- | --- | --- | --- | --- | --- | --- | --- | --- | --- | --- |
| A_70_P035996 | VEGFB | vascular endothelial growth factor B | -2.12 | 0.002 | -1.46 | 0.066 | -1.06 | 0.767 | -1.24 | 0.276 |
| A_70_P036016 | FBN1 | fibrillin 1 | 1.11 | 0.609 | 1.40 | 0.127 | 2.29 | 0.002 | 2.79 | 0.000 |
| A_70_P036066 | GJA5 | gap junction protein, alpha 5, 40kDa | -2.17 | 0.046 | -1.44 | 0.315 | -1.97 | 0.074 | 1.24 | 0.547 |
| A_70_P036071 | MMP9 | matrix metallopeptidase 9 (gelatinase B, 92kDa gelatinase, 92kDa | 1.61 | 0.108 | 2.14 | 0.017 | 5.80 | 0.000 | 4.22 | 0.000 |
| A_70_P036076 | METTL5 | methyltransferase like 5 | -2.11 | 0.000 | -1.52 | 0.015 | -1.35 | 0.066 | -1.84 | 0.001 |
| A_70_P036081 | FABP5 | fatty acid binding protein 5 (psoriasis-associated) | -1.63 | 0.061 | 1.34 | 0.238 | 2.54 | 0.002 | 1.42 | 0.164 |
| A_70_P036182 | CYR61 | cysteine-rich, angiogenic inducer, 61 | 1.01 | 0.980 | -1.31 | 0.314 | -2.08 | 0.016 | -1.84 | 0.037 |
| A_70_P036186 | LOXL1 | lysyl oxidase-like 1 | -1.15 | 0.436 | 1.17 | 0.368 | 1.26 | 0.208 | 2.20 | 0.001 |
| A_70_P036196 | NRM | nurim (nuclear envelope membrane protein) | -1.92 | 0.001 | 2.35 | 0.000 | 2.18 | 0.000 | 1.34 | 0.074 |
| A_70_P036226 | P4HA2 | prolyl 4-hydroxylase, alpha polypeptide II | 1.14 | 0.515 | 1.51 | 0.054 | 1.52 | 0.052 | 2.48 | 0.001 |
| A_70_P036386 | UCHL1 | ubiquitin carboxyl-terminal esterase L1 (ubiquitin thiolesterase | -1.09 | 0.781 | -1.64 | 0.119 | 1.48 | 0.207 | 2.75 | 0.005 |
| A_70_P036631 | TGFB1I1 | transforming growth factor beta 1 induced transcript 1 | -2.08 | 0.003 | -1.34 | 0.162 | -1.23 | 0.309 | -1.12 | 0.561 |
| A_70_P036711 | MCAM | melanoma cell adhesion molecule | -2.39 | 0.002 | -1.89 | 0.013 | -1.97 | 0.010 | -1.45 | 0.118 |
| A_70_P036871 | HCLS1 | hematopoietic cell-specific Lyn substrate 1 | -2.32 | 0.007 | -1.05 | 0.849 | 1.20 | 0.491 | 1.48 | 0.157 |
| A_70_P036936 | LRRN1 | leucine rich repeat neuronal 1 | -2.33 | 0.025 | -3.54 | 0.002 | -4.27 | 0.001 | -2.39 | 0.022 |
| A_70_P036951 | PPP1R3A | protein phosphatase 1, regulatory (inhibitor) subunit 3A | -3.87 | 0.000 | -2.44 | 0.001 | -3.77 | 0.000 | -3.38 | 0.000 |
| A_70_P036956 | BRD3 | bromodomain containing 3 | -2.10 | 0.001 | -1.61 | 0.022 | -1.53 | 0.035 | -1.30 | 0.173 |

| **Probenames** | **Gene Symbol** | **Gene Name** | **6h vs Baseline**  **Fold**  **Change P** | | **d1 vs Baseline**  **Fold**  **Change P** | | **d3 vs Baseline**  **Fold**  **Change P** | | **d7 vs Baseline**  **Fold**  **Change P** | |
| --- | --- | --- | --- | --- | --- | --- | --- | --- | --- | --- |
| A_70_P037036 | TTC8 | tetratricopeptide repeat domain 8 | -2.22 | 0.002 | -1.70 | 0.024 | -1.65 | 0.031 | -1.44 | 0.105 |
| A_70_P037071 | PCMTD2 | protein-L-isoaspartate (D-aspartate) O-methyltransferase domain | -2.83 | 0.000 | -2.07 | 0.002 | -1.69 | 0.014 | -1.28 | 0.195 |
| A_70_P037081 | SLC44A2 | solute carrier family 44, member 2 | -2.16 | 0.003 | -1.42 | 0.114 | -1.02 | 0.921 | 1.07 | 0.741 |
| A_70_P037126 | CDCA8 | cell division cycle associated 8 | 1.50 | 0.137 | 3.13 | 0.001 | 3.31 | 0.001 | 1.24 | 0.419 |
| A_70_P037146 | SUMF1 | sulfatase modifying factor 1 | -1.44 | 0.176 | -1.42 | 0.192 | -2.30 | 0.006 | -1.60 | 0.088 |
| A_70_P037256 | RNASE6 | ribonuclease, RNase A family, k6 | -2.11 | 0.004 | -1.06 | 0.802 | 1.95 | 0.008 | 1.69 | 0.029 |
| A_70_P037276 | FBXO5 | F-box protein 5 | 1.08 | 0.570 | 3.57 | 0.000 | 2.89 | 0.000 | 2.00 | 0.000 |
| A_70_P037336 | ENO2 | enolase 2 (gamma, neuronal) | -1.83 | 0.032 | -2.31 | 0.006 | -2.19 | 0.008 | 1.13 | 0.629 |
| A_70_P037366 | PGAM2 | phosphoglycerate mutase 2 (muscle) | 1.69 | 0.002 | 2.17 | 0.000 | 1.77 | 0.001 | 1.24 | 0.141 |
| A_70_P037396 | AFAP1L2 | actin filament associated protein 1-like 2 | -2.08 | 0.001 | -1.55 | 0.025 | 1.13 | 0.500 | -1.02 | 0.891 |
| A_70_P037411 | MT1E | metallothionein 1E (MT1E) | 110.22 | 0.000 | 59.84 | 0.000 | 15.16 | 0.000 | 2.28 | 0.044 |
| A_70_P037461 | PLA2G2A | phospholipase A2 group IIA-like | 3.42 | 0.000 | 2.03 | 0.003 | 1.29 | 0.211 | 1.05 | 0.803 |
| A_70_P037466 | CD52 | CD52 molecule | -2.79 | 0.013 | -1.83 | 0.114 | -1.10 | 0.793 | -1.00 | 0.991 |
| A_70_P037491 | C7 | complement component 7 | -1.17 | 0.534 | 1.96 | 0.016 | 3.18 | 0.000 | 2.10 | 0.009 |
| A_70_P037521 | FGB | fibrinogen beta chain | -2.20 | 0.001 | -2.02 | 0.002 | -2.09 | 0.002 | -1.58 | 0.026 |
| A_70_P037556 | NDC80 | NDC80 homolog, kinetochore complex component (S. cerevisiae) | -1.35 | 0.232 | 3.97 | 0.000 | 6.21 | 0.000 | 2.60 | 0.002 |
| A_70_P037601 | PCYT2 | phosphate cytidylyltransferase 2, ethanolamine | 1.66 | 0.158 | 2.17 | 0.039 | 2.36 | 0.024 | -1.12 | 0.744 |
| A_70_P037756 | DDB2 | damage-specific DNA binding protein 2, 48kDa | -2.17 | 0.001 | -1.22 | 0.274 | -1.37 | 0.092 | -1.29 | 0.160 |
| A_70_P038041 | HP | haptoglobin (HP) | 19.55 | 0.000 | 32.51 | 0.000 | 12.06 | 0.001 | 5.54 | 0.012 |

| **Probenames** | **Gene Symbol** | **Gene Name** | **6h vs Baseline**  **Fold**  **Change P** | | **d1 vs Baseline**  **Fold**  **Change P** | | **d3 vs Baseline**  **Fold**  **Change P** | | **d7 vs Baseline**  **Fold**  **Change P** | |
| --- | --- | --- | --- | --- | --- | --- | --- | --- | --- | --- |
| A_70_P038046 | CA3 | carbonic anhydrase III, muscle specific | -5.08 | 0.001 | -4.92 | 0.001 | -3.39 | 0.007 | -2.33 | 0.045 |
| A_70_P038056 | FN1 | fibronectin 1 | 2.11 | 0.015 | 1.59 | 0.107 | 2.53 | 0.004 | 5.47 | 0.000 |
| A_70_P038146 | C1QTNF6 | C1q and tumor necrosis factor related protein 6 | -1.06 | 0.736 | 1.12 | 0.467 | 2.23 | 0.000 | 1.78 | 0.003 |
| A_70_P038231 | FMOD | fibromodulin | -1.12 | 0.684 | -2.50 | 0.007 | 1.22 | 0.495 | 1.55 | 0.145 |
| A_70_P038236 | TCF4 | transcription factor 4 | -2.23 | 0.002 | -1.29 | 0.228 | -1.05 | 0.818 | 1.31 | 0.206 |
| A_70_P038251 | PIK3IP1 | phosphoinositide-3-kinase interacting protein 1 | -2.38 | 0.001 | -2.39 | 0.001 | -1.93 | 0.004 | 1.00 | 0.988 |
| A_70_P038281 | SERPINF1 | serpin peptidase inhibitor, clade F (alpha-2 antiplasmin, pigmen | 1.94 | 0.079 | 1.37 | 0.375 | 2.99 | 0.008 | 3.92 | 0.002 |
| A_70_P038301 | CCL21 | chemokine (C-C motif) ligand 21 | -3.26 | 0.007 | 1.15 | 0.700 | 4.98 | 0.001 | 1.56 | 0.242 |
| A_70_P038351 | CFI | complement factor I | 1.39 | 0.230 | 2.09 | 0.015 | 1.68 | 0.069 | 1.40 | 0.215 |
| A_70_P038376 | SLCO2B1 | solute carrier organic anion transporter family, member 2B1 | -2.21 | 0.003 | -1.67 | 0.035 | -1.14 | 0.564 | 1.20 | 0.405 |
| A_70_P038421 | MSR1 | macrophage scavenger receptor 1 | -2.06 | 0.026 | -1.47 | 0.200 | 1.44 | 0.219 | 2.46 | 0.008 |
| A_70_P038426 | KDELR3 | KDEL (Lys-Asp-Glu-Leu) endoplasmic reticulum protein retention receptor 3 | 1.21 | 0.297 | 2.25 | 0.001 | 1.98 | 0.002 | 1.53 | 0.034 |
| A_70_P038431 | PLOD2 | procollagen-lysine, 2-oxoglutarate 5-dioxygenase 2 | -1.01 | 0.947 | -1.05 | 0.789 | 1.55 | 0.025 | 2.89 | 0.000 |
| A_70_P038456 | AGT | angiotensinogen (serpin peptidase inhibitor, clade A, member 8) | -3.03 | 0.016 | -3.23 | 0.012 | -5.21 | 0.001 | -1.58 | 0.272 |
| A_70_P038491 | DLGAP5 | discs, large (Drosophila) homolog-associated protein 5 | -1.12 | 0.429 | 2.41 | 0.000 | 2.75 | 0.000 | 1.55 | 0.007 |

| **Probenames** | **Gene Symbol** | **Gene Name** | **6h vs Baseline**  **Fold**  **Change P** | | **d1 vs Baseline**  **Fold**  **Change P** | | **d3 vs Baseline**  **Fold**  **Change P** | | **d7 vs Baseline**  **Fold**  **Change P** | |
| --- | --- | --- | --- | --- | --- | --- | --- | --- | --- | --- |
| A_70_P038496 | FN1 | fibronectin 1 | 1.47 | 0.262 | 1.44 | 0.287 | 1.87 | 0.079 | 3.39 | 0.003 |
| A_70_P038556 | MUSTN1 | musculoskeletal, embryonic nuclear protein 1 | 1.64 | 0.026 | 1.92 | 0.006 | 1.37 | 0.130 | -2.12 | 0.002 |
| A_70_P038631 | PLOD2 | procollagen-lysine, 2-oxoglutarate 5-dioxygenase 2 | 1.13 | 0.484 | 1.08 | 0.679 | 1.85 | 0.004 | 2.65 | 0.000 |
| A_70_P038686 | MFNG | MFNG O-fucosylpeptide 3-beta-N-acetylglucosaminyltransferase | -3.22 | 0.000 | -1.33 | 0.234 | -1.35 | 0.206 | 1.11 | 0.653 |
| A_70_P038836 | CYBB | cytochrome b-245, beta polypeptide | -2.13 | 0.003 | -1.47 | 0.082 | -1.18 | 0.437 | 1.50 | 0.068 |
| A_70_P038861 | PLAC8 | placenta-specific 8 | -9.34 | 0.001 | -2.87 | 0.058 | -1.69 | 0.315 | -1.00 | 0.999 |
| A_70_P038946 | GNL3 | guanine nucleotide binding protein-like 3 (nucleolar) | 2.22 | 0.001 | 2.19 | 0.001 | 1.09 | 0.671 | 1.04 | 0.827 |
| A_70_P039081 | LYAR | Ly1 antibody reactive homolog (mouse) | 2.03 | 0.002 | 2.45 | 0.000 | 1.57 | 0.027 | -1.27 | 0.210 |
| A_70_P039176 | KLHL24 | kelch-like 24 (Drosophila) | -2.76 | 0.000 | -2.22 | 0.001 | -1.37 | 0.093 | -1.29 | 0.168 |
| A_70_P039261 | CA1 | carbonic anhydrase I | -3.07 | 0.006 | -4.09 | 0.001 | -1.96 | 0.070 | -3.68 | 0.002 |
| A_70_P039262 | CA1 | carbonic anhydrase I | -3.06 | 0.009 | -3.76 | 0.003 | -2.17 | 0.053 | -3.58 | 0.004 |
| A_70_P039276 | NPY | neuropeptide Y | -1.62 | 0.023 | -2.21 | 0.001 | -3.07 | 0.000 | -3.69 | 0.000 |
| A_70_P039277 | NPY | neuropeptide Y | -1.57 | 0.028 | -2.22 | 0.001 | -2.76 | 0.000 | -3.32 | 0.000 |
| A_70_P039286 | CAV1 | caveolin 1, caveolae protein, 22kDa | -2.95 | 0.003 | -1.68 | 0.104 | -1.51 | 0.187 | 1.43 | 0.253 |
| A_70_P039301 | SFTPC | surfactant protein C | -23.90 | 0.007 | -10.02 | 0.036 | -7.77 | 0.058 | -3.84 | 0.194 |
| A_70_P039302 | SFTPC | surfactant protein C | -32.46 | 0.006 | -12.20 | 0.033 | -10.58 | 0.042 | -4.44 | 0.177 |
| A_70_P039311 | CD3D | CD3d molecule, delta (CD3-TCR complex) | -2.89 | 0.001 | -1.91 | 0.026 | -1.29 | 0.335 | 1.01 | 0.968 |
| A_70_P039312 | CD3D | CD3d molecule, delta (CD3-TCR complex) | -3.13 | 0.001 | -2.03 | 0.019 | -1.35 | 0.276 | 1.04 | 0.894 |
| A_70_P039316 | MIF | macrophage migration inhibitory factor (glycosylation-inhibiting | -1.03 | 0.896 | 1.76 | 0.014 | 2.12 | 0.002 | -1.35 | 0.152 |

| **Probenames** | **Gene Symbol** | **Gene Name** | **6h vs Baseline**  **Fold**  **Change P** | | **d1 vs Baseline**  **Fold**  **Change P** | | **d3 vs Baseline**  **Fold**  **Change P** | | **d7 vs Baseline**  **Fold**  **Change P** | |
| --- | --- | --- | --- | --- | --- | --- | --- | --- | --- | --- |
| A_70_P039336 | LOC443162 | galectin-14 | -2.03 | 0.007 | -1.48 | 0.095 | -1.21 | 0.405 | -1.85 | 0.015 |
| A_70_P039337 | LOC443162 | galectin-14 | -2.00 | 0.006 | -1.46 | 0.097 | -1.26 | 0.289 | -1.82 | 0.015 |
| A_70_P039351 | CD247 | CD247 molecule | -2.23 | 0.001 | -1.47 | 0.065 | -1.36 | 0.131 | 1.22 | 0.313 |
| A_70_P039352 | CD247 | CD247 molecule | -2.16 | 0.002 | -1.49 | 0.059 | -1.39 | 0.109 | 1.24 | 0.279 |
| A_70_P039366 | CXCL10 | chemokine (C-X-C motif) ligand 10 | -3.78 | 0.013 | -1.89 | 0.187 | 1.37 | 0.503 | 1.60 | 0.326 |
| A_70_P039391 | CR2 | complement component (3d/Epstein Barr virus) receptor 2 | -20.78 | 0.001 | -8.47 | 0.013 | -5.86 | 0.033 | -2.33 | 0.273 |
| A_70_P039392 | CR2 | complement component (3d/Epstein Barr virus) receptor 2 | -15.44 | 0.002 | -7.17 | 0.016 | -5.45 | 0.034 | -1.72 | 0.456 |
| A_70_P039396 | GRO | GRO protein | 1.65 | 0.041 | 1.21 | 0.400 | 2.04 | 0.007 | -1.10 | 0.670 |
| A_70_P039397 | GRO | GRO protein | 1.56 | 0.073 | 1.18 | 0.471 | 2.03 | 0.009 | -1.15 | 0.547 |
| A_70_P039426 | PTGDS | prostaglandin D2 synthase 21kDa (brain) | -1.48 | 0.152 | -1.95 | 0.023 | -1.42 | 0.193 | -2.32 | 0.006 |
| A_70_P039432 | CD40LG | CD40 ligand | -2.15 | 0.002 | -1.87 | 0.006 | -1.71 | 0.014 | -1.08 | 0.699 |
| A_70_P039451 | SFN | stratifin | 2.49 | 0.004 | 4.69 | 0.000 | 5.39 | 0.000 | 2.08 | 0.014 |
| A_70_P039461 | LOC100101238 | regakine 1-like protein | -2.02 | 0.073 | 1.49 | 0.291 | 8.17 | 0.000 | 4.29 | 0.002 |
| A_70_P039462 | LOC100101238 | regakine 1-like protein | -1.93 | 0.091 | 1.48 | 0.295 | 8.48 | 0.000 | 4.49 | 0.001 |
| A_70_P039466 | CD3E | CD3e molecule, epsilon (CD3-TCR complex) | -3.49 | 0.000 | -1.98 | 0.022 | -1.47 | 0.167 | -1.08 | 0.762 |
| A_70_P039467 | CD3E | CD3e molecule, epsilon (CD3-TCR complex) | -2.72 | 0.002 | -1.75 | 0.051 | -1.34 | 0.278 | 1.11 | 0.686 |
| A_70_P039471 | CRYAA | crystallin, alpha A | 2.05 | 0.044 | 1.81 | 0.088 | 2.59 | 0.012 | -1.18 | 0.609 |
| A_70_P039472 | CRYAA | crystallin, alpha A | 2.08 | 0.063 | 2.02 | 0.074 | 2.86 | 0.012 | -1.53 | 0.255 |
| A_70_P039501 | CAPN3 | calpain 3, (p94) | -2.48 | 0.000 | -1.02 | 0.930 | 1.23 | 0.266 | 1.73 | 0.010 |
| A_70_P039502 | CAPN3 | calpain 3, (p94) | -2.08 | 0.001 | 1.03 | 0.853 | 1.36 | 0.095 | 1.89 | 0.003 |

| **Probenames** | **Gene Symbol** | **Gene Name** | **6h vs Baseline**  **Fold**  **Change P** | | **d1 vs Baseline**  **Fold**  **Change P** | | **d3 vs Baseline**  **Fold**  **Change P** | | **d7 vs Baseline**  **Fold**  **Change P** | |
| --- | --- | --- | --- | --- | --- | --- | --- | --- | --- | --- |
| A_70_P039516 | SELP | selectin P (granule membrane protein 140kDa, antigen CD62) | 11.27 | 0.000 | 12.42 | 0.000 | 4.28 | 0.001 | 1.96 | 0.080 |
| A_70_P039517 | SELP | selectin P (granule membrane protein 140kDa, antigen CD62) | 10.98 | 0.000 | 12.07 | 0.000 | 4.47 | 0.001 | 2.10 | 0.041 |
| A_70_P039526 | CRYAB | crystallin, alpha B | 1.27 | 0.403 | 1.09 | 0.759 | -1.76 | 0.062 | -2.57 | 0.005 |
| A_70_P039531 | STAR | steroidogenic acute regulatory protein | -1.37 | 0.110 | -1.11 | 0.569 | 2.34 | 0.001 | 2.34 | 0.001 |
| A_70_P039532 | STAR | steroidogenic acute regulatory protein | -1.30 | 0.187 | -1.04 | 0.840 | 2.34 | 0.001 | 2.36 | 0.001 |
| A_70_P039536 | MX1 | myxovirus (influenza virus) resistance 1, interferon-inducible p | -2.15 | 0.001 | -1.40 | 0.091 | -1.12 | 0.560 | -1.35 | 0.131 |
| A_70_P039576 | APOBEC3H | apolipoprotein B mRNA editing enzyme, catalytic polypeptide-like | -2.17 | 0.000 | -1.33 | 0.087 | -1.05 | 0.773 | -1.15 | 0.386 |
| A_70_P039577 | APOBEC3H | apolipoprotein B mRNA editing enzyme, catalytic polypeptide-like | -2.19 | 0.041 | 1.29 | 0.472 | -1.08 | 0.828 | -1.14 | 0.716 |
| A_70_P039601 | LHB | luteinizing hormone beta polypeptide | -3.39 | 0.005 | -2.07 | 0.065 | -2.82 | 0.013 | -1.31 | 0.464 |
| A_70_P039641 | ABCG2 | ATP-binding cassette, sub-family G (WHITE), member 2 | -4.97 | 0.000 | -2.04 | 0.010 | -1.27 | 0.328 | -1.54 | 0.089 |
| A_70_P039661 | IL8 | interleukin 8 | 10.75 | 0.000 | 6.06 | 0.001 | 27.24 | 0.000 | 8.40 | 0.000 |
| A_70_P039662 | IL8 | interleukin 8 | 9.17 | 0.000 | 5.31 | 0.002 | 25.35 | 0.000 | 7.50 | 0.000 |
| A_70_P039676 | AHSG | alpha-2-HS-glycoprotein | 2.23 | 0.025 | 2.08 | 0.037 | 3.29 | 0.002 | 1.19 | 0.592 |
| A_70_P039677 | AHSG | alpha-2-HS-glycoprotein | 2.31 | 0.018 | 2.14 | 0.029 | 3.35 | 0.002 | 1.14 | 0.687 |
| A_70_P039696 | OXT | oxytocin, prepropeptide | 2.44 | 0.036 | 2.28 | 0.050 | 4.17 | 0.003 | -1.30 | 0.505 |
| A_70_P039697 | OXT | oxytocin, prepropeptide | 2.38 | 0.048 | 2.25 | 0.062 | 4.16 | 0.004 | -1.21 | 0.637 |
| A_70_P039701 | SCD5 | stearoyl-CoA desaturase 5 | -2.16 | 0.003 | -1.78 | 0.016 | -1.80 | 0.015 | -1.79 | 0.015 |

| **Probenames** | **Gene Symbol** | **Gene Name** | **6h vs Baseline**  **Fold**  **Change P** | | **d1 vs Baseline**  **Fold**  **Change P** | | **d3 vs Baseline**  **Fold**  **Change P** | | **d7 vs Baseline**  **Fold**  **Change P** | |
| --- | --- | --- | --- | --- | --- | --- | --- | --- | --- | --- |
| A_70_P039702 | SCD5 | stearoyl-CoA desaturase 5 | -2.02 | 0.003 | -1.70 | 0.016 | -1.78 | 0.011 | -1.78 | 0.010 |
| A_70_P039706 | IL6 | interleukin 6 (interferon, beta 2) | 54.41 | 0.000 | 54.73 | 0.000 | 23.65 | 0.000 | 4.40 | 0.029 |
| A_70_P039721 | SERPINC1 | serpin peptidase inhibitor, clade C (antithrombin), member 1 | -2.21 | 0.000 | -1.53 | 0.025 | -1.74 | 0.006 | -1.63 | 0.013 |
| A_70_P039766 | ATP7B | ATPase, Cu++ transporting, beta polypeptide" | -2.14 | 0.012 | -2.22 | 0.009 | -1.51 | 0.138 | -1.61 | 0.089 |
| A_70_P039916 | UCK2 | uridine-cytidine kinase 2 | 2.40 | 0.000 | 3.42 | 0.000 | 2.70 | 0.000 | 1.46 | 0.034 |
| A_70_P039986 | SLC27A2 | solute carrier family 27 (fatty acid transporter), member 2 | -2.94 | 0.005 | -1.49 | 0.223 | -2.24 | 0.024 | -2.24 | 0.024 |
| A_70_P040136 | TACC3 | transforming, acidic coiled-coil containing protein 3 | -1.81 | 0.007 | 2.42 | 0.000 | 2.68 | 0.000 | 1.28 | 0.204 |
| A_70_P040161 | MRPS27 | mitochondrial ribosomal protein S27 | 1.71 | 0.180 | 2.09 | 0.075 | 2.42 | 0.038 | -1.42 | 0.376 |
| A_70_P040181 | GRB7 | growth factor receptor-bound protein 7 | -2.65 | 0.001 | -1.66 | 0.043 | -1.75 | 0.027 | -1.53 | 0.081 |
| A_70_P040211 | TBX3 | T-box 3 | -2.99 | 0.003 | -2.45 | 0.010 | -2.44 | 0.010 | -1.35 | 0.321 |
| A_70_P040316 | KANK3 | KN motif and ankyrin repeat domains 3 | -2.07 | 0.002 | -1.79 | 0.008 | -1.54 | 0.037 | 1.10 | 0.625 |
| A_70_P040406 | TRIP13 | thyroid hormone receptor interactor 13 | 1.46 | 0.033 | 3.59 | 0.000 | 2.44 | 0.000 | 1.58 | 0.014 |
| A_70_P040436 | PRC1 | protein regulator of cytokinesis 1 | -1.13 | 0.598 | 4.24 | 0.000 | 4.91 | 0.000 | 2.28 | 0.003 |
| A_70_P040616 | NTRK2 | neurotrophic tyrosine kinase, receptor, type 2 | -1.82 | 0.020 | -2.36 | 0.002 | -2.41 | 0.002 | -2.29 | 0.003 |
| A_70_P040641 | ARL4A | ADP-ribosylation factor-like 4A | -2.04 | 0.000 | -1.01 | 0.914 | 1.19 | 0.229 | -1.02 | 0.897 |
| A_70_P040646 | TRIM45 | tripartite motif containing 45 | -2.94 | 0.001 | -2.23 | 0.005 | -1.80 | 0.028 | -1.14 | 0.594 |
| A_70_P040681 | RPS6KA5 | ribosomal protein S6 kinase, 90kDa, polypeptide 5 | -2.62 | 0.000 | -2.01 | 0.001 | -1.70 | 0.007 | -1.52 | 0.024 |

| **Probenames** | **Gene Symbol** | **Gene Name** | **6h vs Baseline**  **Fold**  **Change P** | | **d1 vs Baseline**  **Fold**  **Change P** | | **d3 vs Baseline**  **Fold**  **Change P** | | **d7 vs Baseline**  **Fold**  **Change P** | |
| --- | --- | --- | --- | --- | --- | --- | --- | --- | --- | --- |
| A_70_P040786 | CIITA | class II, major histocompatibility complex, transactivator | -2.47 | 0.002 | -1.83 | 0.019 | -1.69 | 0.038 | -1.17 | 0.504 |
| A_70_P040826 | TRPM2 | transient receptor potential cation channel, subfamily M, member | -2.48 | 0.011 | -2.02 | 0.039 | -2.03 | 0.038 | 1.25 | 0.476 |
| A_70_P040876 | MLEC | malectin | 1.63 | 0.016 | 2.56 | 0.000 | 2.07 | 0.001 | 1.15 | 0.427 |
| A_70_P040916 | ORC1L | origin recognition complex, subunit 1-like (ORC1L) | -1.16 | 0.224 | 4.64 | 0.000 | 2.73 | 0.000 | 1.65 | 0.001 |
| A_70_P040956 | GZMB | granzyme B (granzyme 2, cytotoxic T-lymphocyte-associated serine | -2.94 | 0.009 | -1.98 | 0.070 | -2.39 | 0.027 | -1.02 | 0.947 |
| A_70_P041046 | PPP1R12A | protein phosphatase 1, regulatory (inhibitor) subunit 12A | -1.70 | 0.030 | -1.77 | 0.021 | -2.14 | 0.004 | -1.57 | 0.057 |
| A_70_P041221 | SLC2A4 | solute carrier family 2 (facilitated glucose transporter), membe | -1.89 | 0.045 | -1.93 | 0.039 | -2.55 | 0.006 | -3.17 | 0.002 |
| A_70_P041222 | SLC2A4 | solute carrier family 2 (facilitated glucose transporter), membe | -1.99 | 0.032 | -1.92 | 0.041 | -2.59 | 0.006 | -3.28 | 0.001 |
| A_70_P041226 | LOC443338 | secretory protein | -1.34 | 0.096 | 1.56 | 0.019 | 2.10 | 0.001 | -1.02 | 0.916 |
| A_70_P041227 | PLP2 | proteolipid protein 2 (colonic epithelium-enriched) | -1.34 | 0.118 | 1.60 | 0.019 | 2.16 | 0.001 | 1.06 | 0.751 |
| A_70_P041246 | SCNN1B | sodium channel, nonvoltage-gated 1, beta | -2.46 | 0.001 | -2.07 | 0.005 | -2.57 | 0.001 | -2.10 | 0.004 |
| A_70_P041256 | INSR | insulin receptor | -1.69 | 0.021 | -1.64 | 0.028 | -2.11 | 0.003 | -1.59 | 0.037 |
| A_70_P041257 | INSR | insulin receptor | -1.73 | 0.017 | -1.59 | 0.037 | -2.02 | 0.004 | -1.61 | 0.034 |
| A_70_P041276 | CPE | carboxypeptidase E | -1.79 | 0.004 | -2.42 | 0.000 | -1.05 | 0.781 | 1.27 | 0.175 |
| A_70_P041277 | CPE | carboxypeptidase E | -1.83 | 0.004 | -2.45 | 0.000 | -1.05 | 0.798 | 1.27 | 0.183 |

| **Probenames** | **Gene Symbol** | **Gene Name** | **6h vs Baseline**  **Fold**  **Change P** | | **d1 vs Baseline**  **Fold**  **Change P** | | **d3 vs Baseline**  **Fold**  **Change P** | | **d7 vs Baseline**  **Fold**  **Change P** | |
| --- | --- | --- | --- | --- | --- | --- | --- | --- | --- | --- |
| A_70_P041326 | NOS2 | nitric oxide synthase 2, inducible | -1.86 | 0.003 | -2.14 | 0.001 | -1.63 | 0.014 | 1.25 | 0.217 |
| A_70_P041327 | NOS2 | nitric oxide synthase 2, inducible | -1.89 | 0.000 | -2.14 | 0.000 | -1.47 | 0.013 | 1.11 | 0.461 |
| A_70_P041351 | BCAT2 | branched chain amino-acid transaminase 2, mitochondrial | 2.54 | 0.004 | 2.37 | 0.006 | 1.61 | 0.088 | -1.17 | 0.562 |
| A_70_P041352 | BCAT2 | branched chain amino-acid transaminase 2, mitochondrial | 2.42 | 0.005 | 2.22 | 0.009 | 1.58 | 0.101 | -1.13 | 0.637 |
| A_70_P041476 | EM4c | EM4c protein | -2.62 | 0.003 | -1.66 | 0.074 | -1.68 | 0.067 | -1.59 | 0.099 |
| A_70_P041511 | SFTPB | surfactant protein B | -7.27 | 0.002 | -1.32 | 0.587 | -1.48 | 0.451 | -1.59 | 0.376 |
| A_70_P041512 | SFTPB | surfactant protein B | -6.21 | 0.003 | -1.15 | 0.784 | -1.33 | 0.583 | -1.40 | 0.513 |
| A_70_P041516 | SP-D | surfactant protein D | -2.94 | 0.035 | -1.09 | 0.855 | -2.79 | 0.044 | -1.56 | 0.348 |
| A_70_P041517 | SP-D | surfactant protein D | -2.90 | 0.038 | -1.07 | 0.878 | -3.04 | 0.032 | -1.55 | 0.357 |
| A_70_P041626 | ENO3 | enolase 3 (beta, muscle) | -1.60 | 0.030 | 1.06 | 0.750 | 1.09 | 0.674 | 2.10 | 0.002 |
| A_70_P041627 | ENO3 | enolase 3 (beta, muscle) | -1.68 | 0.025 | 1.07 | 0.753 | 1.04 | 0.839 | 2.13 | 0.003 |
| A_70_P041632 | KDR | kinase insert domain receptor (a type III receptor tyrosine kina | -2.12 | 0.010 | -1.09 | 0.735 | -1.20 | 0.476 | 1.31 | 0.288 |
| A_70_P041641 | LDHA | lactate dehydrogenase A | 2.50 | 0.000 | 2.69 | 0.000 | 2.66 | 0.000 | 1.41 | 0.073 |
| A_70_P041671 | JUNB | jun B proto-oncogene | 2.52 | 0.002 | 1.80 | 0.027 | 2.03 | 0.010 | 1.50 | 0.105 |
| A_70_P041672 | JUNB | jun B proto-oncogene | 2.59 | 0.001 | 1.81 | 0.024 | 2.08 | 0.008 | 1.51 | 0.096 |
| A_70_P041761 | IL18 | interleukin 18 (interferon-gamma-inducing factor) | -2.05 | 0.000 | -1.54 | 0.012 | -1.47 | 0.022 | -1.08 | 0.589 |
| A_70_P041786 | PGFS | prostaglandin F synthase | -5.51 | 0.002 | -3.45 | 0.012 | -3.63 | 0.010 | -2.35 | 0.064 |
| A_70_P041789 | PGFS | prostaglandin F synthase | -4.73 | 0.008 | -3.27 | 0.033 | -1.93 | 0.208 | 1.20 | 0.716 |
| A_70_P041806 | IGFBP3 | insulin-like growth factor binding protein 3 | 1.30 | 0.229 | 2.38 | 0.001 | 2.58 | 0.001 | 2.85 | 0.000 |

| **Probenames** | **Gene Symbol** | **Gene Name** | **6h vs Baseline**  **Fold**  **Change P** | | **d1 vs Baseline**  **Fold**  **Change P** | | **d3 vs Baseline**  **Fold**  **Change P** | | **d7 vs Baseline**  **Fold**  **Change P** | |
| --- | --- | --- | --- | --- | --- | --- | --- | --- | --- | --- |
| A_70_P041807 | IGFBP3 | insulin-like growth factor binding protein 3 | 1.23 | 0.311 | 2.39 | 0.001 | 2.47 | 0.001 | 2.94 | 0.000 |
| A_70_P041816 | IGFBP6 | insulin-like growth factor binding protein 6 | 1.13 | 0.661 | 1.07 | 0.814 | 2.53 | 0.006 | 1.48 | 0.182 |
| A_70_P041817 | IGFBP6 | insulin-like growth factor binding protein 6 | 1.10 | 0.735 | 1.04 | 0.889 | 2.43 | 0.006 | 1.46 | 0.183 |
| A_70_P041841 | CYP1A1 | cytochrome P450, family 1, subfamily A, polypeptide 1 | -13.38 | 0.003 | -9.08 | 0.009 | -16.74 | 0.002 | -3.33 | 0.117 |
| A_70_P041846 | NR3C2 | nuclear receptor subfamily 3, group C, member 2 | -2.48 | 0.002 | -1.81 | 0.024 | -1.92 | 0.015 | -1.85 | 0.020 |
| A_70_P041847 | NR3C2 | nuclear receptor subfamily 3, group C, member 2 | -2.10 | 0.006 | -1.64 | 0.046 | -1.73 | 0.030 | -1.78 | 0.024 |
| A_70_P042051 | C7orf23 | chromosome 7 open reading frame 23 | -2.04 | 0.003 | -1.44 | 0.080 | -1.47 | 0.066 | -1.22 | 0.323 |
| A_70_P042176 | RRS1 | RRS1 ribosome biogenesis regulator homolog (S. cerevisiae) | 2.16 | 0.002 | 1.81 | 0.009 | 1.13 | 0.537 | -1.06 | 0.747 |
| A_70_P042206 | PCK2 | phosphoenolpyruvate carboxykinase 2 (mitochondrial) | 2.58 | 0.002 | 3.46 | 0.000 | 2.36 | 0.003 | 1.23 | 0.395 |
| A_70_P042311 | WWC2 | WW and C2 domain containing 2 | 2.29 | 0.005 | 1.50 | 0.114 | 1.04 | 0.877 | -1.19 | 0.484 |
| A_70_P042346 | NPHP1 | nephronophthisis 1 (juvenile) | -2.34 | 0.006 | -1.44 | 0.173 | -1.49 | 0.141 | -1.17 | 0.552 |
| A_70_P042406 | DNAJB11 | DnaJ (Hsp40) homolog, subfamily B, member 11 | 2.35 | 0.000 | 2.54 | 0.000 | 1.46 | 0.048 | 1.24 | 0.238 |
| A_70_P042481 | ALDH18A1 | aldehyde dehydrogenase 18 family, member A1 | 2.10 | 0.009 | 2.62 | 0.002 | 1.59 | 0.075 | 1.44 | 0.156 |
| A_70_P042501 | HSD17B11 | hydroxysteroid (17-beta) dehydrogenase 11 | -2.67 | 0.003 | -1.56 | 0.120 | -1.88 | 0.035 | -1.08 | 0.775 |
| A_70_P042531 | GYPC | glycophorin C (Gerbich blood group) | -2.86 | 0.000 | -1.74 | 0.009 | 1.13 | 0.496 | -1.15 | 0.458 |
| A_70_P042561 | PRRC1 | proline-rich coiled-coil 1 | 1.14 | 0.572 | 2.07 | 0.008 | 1.76 | 0.031 | 1.68 | 0.043 |

| **Probenames** | **Gene Symbol** | **Gene Name** | **6h vs Baseline**  **Fold**  **Change P** | | **d1 vs Baseline**  **Fold**  **Change P** | | **d3 vs Baseline**  **Fold**  **Change P** | | **d7 vs Baseline**  **Fold**  **Change P** | |
| --- | --- | --- | --- | --- | --- | --- | --- | --- | --- | --- |
| A_70_P042661 | BBS4 | Bardet-Biedl syndrome 4 | -1.90 | 0.023 | -1.56 | 0.097 | -2.06 | 0.013 | -1.36 | 0.240 |
| A_70_P042706 | GINS4 | GINS complex subunit 4 (Sld5 homolog) | 1.01 | 0.951 | 2.48 | 0.000 | 1.71 | 0.004 | 1.24 | 0.182 |
| A_70_P042771 | MYBL2 | v-myb myeloblastosis viral oncogene homolog (avian)-like 2 | -2.16 | 0.000 | 2.35 | 0.000 | 1.65 | 0.007 | 1.20 | 0.258 |
| A_70_P042821 | ASGR1 | asialoglycoprotein receptor 1 | -1.61 | 0.274 | -1.22 | 0.646 | 1.60 | 0.279 | 2.49 | 0.048 |
| A_70_P042831 | MAP2K6 | mitogen-activated protein kinase kinase 6 | -3.39 | 0.000 | -1.61 | 0.019 | -1.19 | 0.344 | -1.41 | 0.074 |
| A_70_P042941 | ECE2 | endothelin converting enzyme 2 | 3.25 | 0.000 | 3.54 | 0.000 | 2.86 | 0.000 | 1.34 | 0.153 |
| A_70_P042956 | SDF2L1 | stromal cell-derived factor 2-like 1 | 2.27 | 0.001 | 4.73 | 0.000 | 2.63 | 0.000 | 1.21 | 0.357 |
| A_70_P043006 | TNFAIP8L1 | tumor necrosis factor, alpha-induced protein 8-like 1 | -2.00 | 0.004 | -1.45 | 0.076 | -1.53 | 0.048 | -1.44 | 0.084 |
| A_70_P043146 | KIF22 | kinesin family member 22 | -1.13 | 0.231 | 2.77 | 0.000 | 3.04 | 0.000 | 1.61 | 0.000 |
| A_70_P043161 | IMP4 | IMP4, U3 small nucleolar ribonucleoprotein, homolog (yeast) | 2.05 | 0.001 | 1.88 | 0.002 | 1.03 | 0.840 | -1.23 | 0.229 |
| A_70_P043236 | FZD1 | frizzled homolog 1 (Drosophila) | -2.24 | 0.000 | -1.60 | 0.005 | 1.24 | 0.144 | 1.46 | 0.017 |
| A_70_P043321 | DKK3 | dickkopf homolog 3 (Xenopus laevis) | -1.63 | 0.067 | -2.19 | 0.007 | -2.34 | 0.004 | -1.40 | 0.187 |
| A_70_P043346 | PPM1K | protein phosphatase, Mg2+/Mn2+ dependent, 1K | -2.52 | 0.000 | -1.49 | 0.056 | -1.65 | 0.022 | -1.22 | 0.323 |
| A_70_P043411 | CXCL12 | chemokine (C-X-C motif) ligand 12 | -2.11 | 0.018 | -1.24 | 0.449 | -1.01 | 0.966 | 1.71 | 0.072 |
| A_70_P043686 | IVL | involucrin | 2.25 | 0.076 | 2.60 | 0.041 | 3.59 | 0.010 | 1.03 | 0.952 |
| A_70_P043726 | MAPT | microtubule-associated protein tau | -2.76 | 0.001 | -2.16 | 0.004 | -4.06 | 0.000 | -1.70 | 0.030 |
| A_70_P043836 | PPP1R16B | protein phosphatase 1, regulatory (inhibitor) subunit 16B | -2.36 | 0.000 | -1.48 | 0.039 | -1.07 | 0.681 | 1.04 | 0.842 |
| A_70_P044061 | MAN1C1 | mannosidase, alpha, class 1C, member 1 | -2.36 | 0.002 | -1.79 | 0.021 | -1.40 | 0.150 | -1.01 | 0.951 |

| **Probenames** | **Gene Symbol** | **Gene Name** | **6h vs Baseline**  **Fold**  **Change P** | | **d1 vs Baseline**  **Fold**  **Change P** | | **d3 vs Baseline**  **Fold**  **Change P** | | **d7 vs Baseline**  **Fold**  **Change P** | |
| --- | --- | --- | --- | --- | --- | --- | --- | --- | --- | --- |
| A_70_P044241 | SMC1A | structural maintenance of chromosomes 1A | 1.06 | 0.716 | 2.04 | 0.001 | 1.50 | 0.020 | 1.23 | 0.194 |
| A_70_P044301 | TMEM154 | transmembrane protein 154 | 1.91 | 0.007 | 2.13 | 0.002 | 2.31 | 0.001 | 1.56 | 0.044 |
| A_70_P044346 | NEXN | nexilin (F actin binding protein) | -1.28 | 0.308 | -1.79 | 0.029 | -2.24 | 0.005 | -1.83 | 0.025 |
| A_70_P044391 | MRPL40 | mitochondrial ribosomal protein L40 | 1.04 | 0.826 | 1.26 | 0.229 | 3.00 | 0.000 | 1.62 | 0.022 |
| A_70_P044681 | TMPRSS4 | transmembrane protease, serine 4 | -2.26 | 0.024 | -2.20 | 0.028 | 1.32 | 0.400 | -1.33 | 0.387 |
| A_70_P044686 | PHLDB2 | pleckstrin homology-like domain, family B, member 2 | -2.15 | 0.004 | -1.48 | 0.090 | -1.24 | 0.331 | 1.09 | 0.687 |
| A_70_P044736 | SDPR | serum deprivation response | -5.90 | 0.002 | -4.23 | 0.007 | -3.29 | 0.020 | -1.31 | 0.555 |
| A_70_P044801 | CCL26 | chemokine (C-C motif) ligand 26 | -1.98 | 0.119 | -4.65 | 0.003 | -2.70 | 0.031 | -3.35 | 0.012 |
| A_70_P044911 | USP7 | ubiquitin specific peptidase 7 (herpes virus-associated) | 2.25 | 0.053 | 1.63 | 0.219 | 2.34 | 0.044 | -1.53 | 0.285 |
| A_70_P044986 | SCAPER | S-phase cyclin A-associated protein in the ER | 2.13 | 0.107 | 1.76 | 0.216 | 2.72 | 0.040 | -1.43 | 0.426 |
| A_70_P045016 | WNT2B | wingless-type MMTV integration site family, member 2B | -2.52 | 0.000 | -1.88 | 0.004 | -1.30 | 0.154 | -1.27 | 0.190 |
| A_70_P045141 | MTHFD2 | methylenetetrahydrofolate dehydrogenase (NADP+ dependent) 2, met | 1.83 | 0.015 | 2.15 | 0.003 | 1.52 | 0.070 | 1.59 | 0.050 |
| A_70_P045142 | MTHFD2 | methylenetetrahydrofolate dehydrogenase (NADP+ dependent) 2, met | 1.86 | 0.013 | 2.27 | 0.002 | 1.58 | 0.054 | 1.59 | 0.051 |
| A_70_P045231 | LBP | lipopolysaccharide binding protein (LBP) | 44.82 | 0.000 | 49.21 | 0.000 | 13.41 | 0.000 | 4.52 | 0.005 |
| A_70_P045236 | IVD | isovaleryl-CoA dehydrogenase | -2.15 | 0.002 | -1.33 | 0.157 | -1.43 | 0.084 | -1.41 | 0.095 |
| A_70_P045256 | GLYAT | glycine-N-acyltransferase | -3.73 | 0.000 | -1.75 | 0.017 | -1.42 | 0.108 | -1.81 | 0.012 |
| A_70_P045261 | MARCO | macrophage receptor with collagenous structure | -19.40 | 0.008 | -10.76 | 0.025 | -23.17 | 0.005 | -2.34 | 0.378 |

| **Probenames** | **Gene Symbol** | **Gene Name** | **6h vs Baseline**  **Fold**  **Change P** | | **d1 vs Baseline**  **Fold**  **Change P** | | **d3 vs Baseline**  **Fold**  **Change P** | | **d7 vs Baseline**  **Fold**  **Change P** | |
| --- | --- | --- | --- | --- | --- | --- | --- | --- | --- | --- |
| A_70_P045271 | PON1 | paraoxonase 1 | -3.78 | 0.006 | -1.97 | 0.113 | -2.25 | 0.063 | -3.07 | 0.015 |
| A_70_P045431 | CROT | carnitine O-octanoyltransferase | -2.25 | 0.001 | -1.48 | 0.069 | -1.19 | 0.387 | -1.55 | 0.044 |
| A_70_P045486 | CYP2B7P1 | cytochrome P450, family 2, subfamily B, polypeptide 7 pseudogene | -5.23 | 0.032 | -4.60 | 0.045 | -13.47 | 0.003 | -2.33 | 0.239 |
| A_70_P045551 | C1orf38 | chromosome 1 open reading frame 38 | -2.61 | 0.004 | -1.34 | 0.305 | -1.21 | 0.498 | 1.43 | 0.216 |
| A_70_P045711 | HES1 | hairy and enhancer of split 1, (Drosophila) | -2.10 | 0.003 | -1.57 | 0.046 | -1.54 | 0.054 | -1.18 | 0.427 |
| A_70_P045746 | SASH3 | SAM and SH3 domain containing 3 | -2.27 | 0.005 | -1.12 | 0.633 | 1.22 | 0.426 | 1.44 | 0.149 |
| A_70_P045761 | SNX20 | sorting nexin 20 | -2.63 | 0.002 | -1.68 | 0.050 | -1.26 | 0.356 | 1.13 | 0.629 |
| A_70_P045921 | MXD3 | MAX dimerization protein 3 | -1.39 | 0.020 | 1.94 | 0.000 | 2.27 | 0.000 | 1.37 | 0.026 |
| A_70_P045991 | SLAMF7 | SLAM family member 7 | -2.12 | 0.005 | -1.31 | 0.249 | 1.10 | 0.663 | 1.63 | 0.047 |
| A_70_P046016 | ZNF277 | zinc finger protein 277 | -2.30 | 0.000 | -1.42 | 0.055 | 1.06 | 0.751 | -1.27 | 0.175 |
| A_70_P046041 | NTHL1 | nth endonuclease III-like 1 (E. coli) | -2.10 | 0.000 | -1.41 | 0.045 | -1.23 | 0.198 | -1.63 | 0.008 |
| A_70_P046061 | GNPDA1 | glucosamine-6-phosphate deaminase 1 | -2.06 | 0.000 | -1.23 | 0.089 | 1.10 | 0.428 | -1.18 | 0.175 |
| A_70_P046111 | SELENBP1 | selenium binding protein 1 | -4.05 | 0.000 | -3.12 | 0.002 | -3.78 | 0.001 | -2.15 | 0.019 |
| A_70_P046136 | NAA50 | N(alpha)-acetyltransferase 50, NatE catalytic subunit | 1.31 | 0.185 | 2.06 | 0.003 | 1.48 | 0.060 | -1.17 | 0.433 |
| A_70_P046151 | DOCK10 | dedicator of cytokinesis 10 | -2.36 | 0.001 | -1.86 | 0.012 | -1.01 | 0.980 | 1.56 | 0.056 |
| A_70_P046246 | TIMM8A | translocase of inner mitochondrial membrane 8 homolog A (yeast) | 2.28 | 0.000 | 2.08 | 0.000 | 1.35 | 0.073 | -1.07 | 0.656 |
| A_70_P046306 | MLST8 | MTOR associated protein, LST8 homolog (S. cerevisiae) | 1.99 | 0.105 | 1.63 | 0.233 | 2.71 | 0.026 | -1.31 | 0.505 |
| A_70_P046361 | CAV3 | caveolin 3 | -2.13 | 0.048 | -1.51 | 0.252 | -2.32 | 0.030 | -3.65 | 0.003 |
| A_70_P046406 | CYB5 | CYB5 protein | -3.35 | 0.000 | -1.96 | 0.010 | -2.96 | 0.000 | -2.00 | 0.009 |

| **Probenames** | **Gene Symbol** | **Gene Name** | **6h vs Baseline**  **Fold**  **Change P** | | **d1 vs Baseline**  **Fold**  **Change P** | | **d3 vs Baseline**  **Fold**  **Change P** | | **d7 vs Baseline**  **Fold**  **Change P** | |
| --- | --- | --- | --- | --- | --- | --- | --- | --- | --- | --- |
| A_70_P046516 | CAPN6 | calpain 6 | 1.19 | 0.508 | 2.71 | 0.002 | 3.65 | 0.000 | 2.88 | 0.002 |
| A_70_P046591 | TNFAIP6 | tumor necrosis factor, alpha-induced protein 6 | 3.92 | 0.004 | 2.33 | 0.052 | 3.26 | 0.011 | 3.12 | 0.013 |
| A_70_P046651 | S100A14 | S100 calcium binding protein A14 | 1.93 | 0.003 | 2.29 | 0.001 | 2.29 | 0.001 | 1.21 | 0.297 |
| A_70_P046731 | CDC42EP3 | CDC42 effector protein (Rho GTPase binding) 3 | -2.07 | 0.002 | -1.58 | 0.029 | -1.78 | 0.009 | -1.36 | 0.119 |
| A_70_P046746 | PHACTR1 | phosphatase and actin regulator 1 | -2.96 | 0.004 | -2.41 | 0.013 | -2.00 | 0.039 | -1.07 | 0.818 |
| A_70_P046781 | SOCS3 | suppressor of cytokine signaling 3 | 6.05 | 0.000 | 5.45 | 0.000 | 4.65 | 0.000 | 2.82 | 0.001 |
| A_70_P046796 | ERRFI1 | ERBB receptor feedback inhibitor 1 | 2.04 | 0.006 | 1.40 | 0.139 | 1.34 | 0.187 | 1.35 | 0.183 |
| A_70_P046851 | TM7SF2 | transmembrane 7 superfamily member 2 | -2.42 | 0.003 | -1.18 | 0.506 | -1.69 | 0.046 | -1.24 | 0.373 |
| A_70_P046852 | TM7SF2 | transmembrane 7 superfamily member 2 | -2.46 | 0.002 | -1.19 | 0.472 | -1.76 | 0.031 | -1.27 | 0.321 |
| A_70_P046981 | SYNCRIP | synaptotagmin binding, cytoplasmic RNA interacting protein | 1.86 | 0.005 | 2.52 | 0.000 | 1.81 | 0.007 | 1.41 | 0.084 |
| A_70_P046986 | ARL4D | ADP-ribosylation factor-like 4D | 1.50 | 0.146 | 1.43 | 0.191 | 2.17 | 0.012 | -1.04 | 0.893 |
| A_70_P047026 | PPIF | peptidylprolyl isomerase F | 1.63 | 0.052 | 2.18 | 0.005 | 1.22 | 0.406 | 1.07 | 0.775 |
| A_70_P047071 | C1orf93 | chromosome 1 open reading frame 93 | -1.73 | 0.013 | -1.37 | 0.120 | -1.49 | 0.056 | -2.61 | 0.000 |
| A_70_P047136 | ETF1 | eukaryotic translation termination factor 1 | 1.93 | 0.012 | 2.34 | 0.003 | 1.53 | 0.081 | 1.23 | 0.379 |
| A_70_P047191 | APBB3 | amyloid beta (A4) precursor protein-binding, family B, member 3 | -2.06 | 0.001 | -1.57 | 0.023 | -1.46 | 0.051 | -1.27 | 0.189 |
| A_70_P047346 | TMEM176B | transmembrane protein 176B | -1.24 | 0.189 | 1.39 | 0.059 | 2.51 | 0.000 | 1.98 | 0.001 |
| A_70_P047446 | CDC2 | NULL | -1.35 | 0.218 | 5.29 | 0.000 | 7.40 | 0.000 | 2.47 | 0.002 |
| A_70_P047541 | LRMP | lymphoid-restricted membrane protein | -3.60 | 0.001 | -2.02 | 0.030 | -1.79 | 0.065 | -1.45 | 0.223 |
| A_70_P047586 | BBS1 | Bardet-Biedl syndrome 1 | 1.62 | 0.036 | 2.08 | 0.004 | 1.34 | 0.173 | 1.34 | 0.181 |

| **Probenames** | **Gene Symbol** | **Gene Name** | **6h vs Baseline**  **Fold**  **Change P** | | **d1 vs Baseline**  **Fold**  **Change P** | | **d3 vs Baseline**  **Fold**  **Change P** | | **d7 vs Baseline**  **Fold**  **Change P** | |
| --- | --- | --- | --- | --- | --- | --- | --- | --- | --- | --- |
| A_70_P047651 | KIF20A | kinesin family member 20A | -1.11 | 0.678 | 3.10 | 0.001 | 3.29 | 0.000 | 3.12 | 0.001 |
| A_70_P047666 | FXYD5 | FXYD domain containing ion transport regulator 5 | 1.02 | 0.936 | 1.80 | 0.035 | 3.28 | 0.000 | 2.40 | 0.004 |
| A_70_P047726 | COL3A1 | collagen, type III, alpha 1 | -1.32 | 0.305 | -1.21 | 0.486 | 3.02 | 0.001 | 4.98 | 0.000 |
| A_70_P047731 | WISP1 | WNT1 inducible signaling pathway protein 1 | -1.81 | 0.154 | -1.03 | 0.946 | 6.67 | 0.000 | 6.66 | 0.000 |
| A_70_P047741 | TMEM119 | transmembrane protein 119 | -1.45 | 0.085 | 1.24 | 0.299 | 3.58 | 0.000 | 4.54 | 0.000 |
| A_70_P047751 | ARHGEF3 | Rho guanine nucleotide exchange factor (GEF) 3 | -2.02 | 0.002 | -1.51 | 0.039 | -1.29 | 0.178 | -1.05 | 0.789 |
| A_70_P047796 | FBLN7 | fibulin 7 | 1.56 | 0.128 | 1.04 | 0.884 | 1.86 | 0.043 | 2.95 | 0.002 |
| A_70_P047801 | LRRC57 | leucine rich repeat containing 57 | -2.50 | 0.001 | -2.08 | 0.003 | -2.52 | 0.001 | -1.77 | 0.013 |
| A_70_P047816 | DYNC1LI2 | dynein, cytoplasmic 1, light intermediate chain 2 | 1.31 | 0.086 | 2.12 | 0.000 | 2.05 | 0.000 | 1.30 | 0.093 |
| A_70_P047821 | C6orf173 | hypothetical protein LOC787125 | -1.39 | 0.049 | 3.74 | 0.000 | 4.33 | 0.000 | 1.71 | 0.004 |
| A_70_P047846 | COL5A2 | collagen, type V, alpha 2 | -1.33 | 0.225 | -1.07 | 0.755 | 1.99 | 0.010 | 3.56 | 0.000 |
| A_70_P047921 | COL1A1 | collagen, type I, alpha 1 | 1.33 | 0.278 | 1.10 | 0.705 | 3.38 | 0.000 | 4.10 | 0.000 |
| A_70_P047940 | COL4A1 | collagen, type IV, alpha 1 | -1.19 | 0.390 | 1.27 | 0.239 | 1.89 | 0.006 | 2.42 | 0.001 |
| A_70_P047941 | AC004223.3 | NULL | 1.52 | 0.033 | 2.14 | 0.001 | 1.75 | 0.007 | 1.19 | 0.344 |
| A_70_P047961 | COL4A1 | collagen, type IV, alpha 1 | -1.20 | 0.342 | 1.30 | 0.168 | 1.81 | 0.007 | 2.51 | 0.000 |
| A_70_P047966 | CYFIP1 | cytoplasmic FMR1 interacting protein 1 | 1.40 | 0.048 | 2.00 | 0.001 | 2.53 | 0.000 | 1.39 | 0.052 |
| A_70_P047991 | COL1A1 | collagen, type I, alpha 1 | 1.13 | 0.576 | 1.04 | 0.860 | 3.12 | 0.000 | 3.97 | 0.000 |
| A_70_P048049 | COL3A1 | collagen, type III, alpha 1 | -1.53 | 0.148 | -1.35 | 0.295 | 2.99 | 0.002 | 5.22 | 0.000 |
| A_70_P048111 | CSRP1 | cysteine and glycine-rich protein 1 | 2.00 | 0.039 | 2.15 | 0.025 | 1.38 | 0.306 | -1.79 | 0.076 |

| **Probenames** | **Gene Symbol** | **Gene Name** | **6h vs Baseline**  **Fold**  **Change P** | | **d1 vs Baseline**  **Fold**  **Change P** | | **d3 vs Baseline**  **Fold**  **Change P** | | **d7 vs Baseline**  **Fold**  **Change P** | |
| --- | --- | --- | --- | --- | --- | --- | --- | --- | --- | --- |
| A_70_P048131 | GPC3 | glypican 3 | -1.98 | 0.023 | -2.49 | 0.004 | -2.32 | 0.007 | -1.52 | 0.136 |
| A_70_P048151 | BUB1 | budding uninhibited by benzimidazoles 1 homolog (yeast) | -3.68 | 0.000 | -1.31 | 0.332 | -1.25 | 0.417 | -1.56 | 0.120 |
| A_70_P048191 | COL3A1 | collagen, type III, alpha 1 | -1.21 | 0.331 | -1.19 | 0.391 | 2.30 | 0.001 | 4.48 | 0.000 |
| A_70_P048201 | COL1A1 | collagen, type I, alpha 1 | 1.41 | 0.168 | 1.44 | 0.142 | 3.46 | 0.000 | 2.24 | 0.005 |
| A_70_P048246 | TCEA3 | transcription elongation factor A (SII), 3 | -2.15 | 0.004 | -1.16 | 0.511 | -1.05 | 0.820 | -1.67 | 0.038 |
| A_70_P048271 | GNA14 | guanine nucleotide binding protein (G protein), alpha 14 | -2.08 | 0.008 | -1.77 | 0.029 | -1.84 | 0.021 | -1.54 | 0.085 |
| A_70_P048311 | GNA13 | guanine nucleotide binding protein (G protein), alpha 13 | 2.10 | 0.041 | 1.73 | 0.117 | 2.34 | 0.022 | -1.31 | 0.417 |
| A_70_P048321 | ADRBK2 | adrenergic, beta, receptor kinase 2 | -2.11 | 0.002 | -1.30 | 0.199 | -1.57 | 0.039 | -1.26 | 0.253 |
| A_70_P048401 | JUN | jun proto-oncogene | -1.78 | 0.058 | -2.01 | 0.026 | -1.57 | 0.125 | -2.14 | 0.017 |
| A_70_P048421 | CD79B | CD79b molecule, immunoglobulin-associated beta | -2.78 | 0.010 | -1.75 | 0.118 | 1.23 | 0.542 | -1.03 | 0.940 |
| A_70_P048451 | RRM2 | ribonucleotide reductase M2 | -1.46 | 0.036 | 8.39 | 0.000 | 6.15 | 0.000 | 2.75 | 0.000 |
| A_70_P048491 | C9 | complement component 9 | 1.18 | 0.585 | 3.20 | 0.002 | 2.92 | 0.003 | 2.05 | 0.030 |
| A_70_P048636 | ZNFX1 | zinc finger, NFX1-type containing 1 | -1.92 | 0.007 | -1.58 | 0.040 | -2.10 | 0.003 | -1.22 | 0.337 |
| A_70_P048841 | LOC529036 | hypothetical LOC529036 | 3.07 | 0.000 | 3.79 | 0.000 | 3.01 | 0.000 | 2.33 | 0.003 |
| A_70_P048891 | DUSP5 | similar to dual specificity phosphatase 5 | 3.45 | 0.000 | 2.00 | 0.014 | 1.23 | 0.409 | -1.18 | 0.510 |
| A_70_P048896 | TNS4 | tensin 4 | 2.26 | 0.036 | 2.17 | 0.045 | 1.24 | 0.552 | -1.26 | 0.516 |
| A_70_P048901 | ATXN2 | ataxin 2 | -2.03 | 0.004 | -1.64 | 0.027 | -1.99 | 0.004 | -1.26 | 0.258 |
| A_70_P048926 | PSORS1C2 | psoriasis susceptibility 1 candidate 2 | 1.53 | 0.139 | 1.57 | 0.123 | 2.11 | 0.017 | 1.01 | 0.984 |
| A_70_P049006 | DDX21 | DEAD (Asp-Glu-Ala-Asp) box polypeptide 21 | 2.20 | 0.002 | 1.89 | 0.007 | 1.06 | 0.760 | 1.11 | 0.604 |

| **Probenames** | **Gene Symbol** | **Gene Name** | **6h vs Baseline**  **Fold**  **Change P** | | **d1 vs Baseline**  **Fold**  **Change P** | | **d3 vs Baseline**  **Fold**  **Change P** | | **d7 vs Baseline**  **Fold**  **Change P** | |
| --- | --- | --- | --- | --- | --- | --- | --- | --- | --- | --- |
| A_70_P049081 | PDLIM3 | PDZ and LIM domain 3 | -2.54 | 0.001 | -2.11 | 0.005 | -2.35 | 0.002 | -2.50 | 0.001 |
| A_70_P049106 | CALCOCO1 | calcium binding and coiled-coil domain 1 | -2.21 | 0.011 | -1.73 | 0.060 | -1.12 | 0.689 | -1.07 | 0.797 |
| A_70_P049181 | SRSF5 | serine/arginine-rich splicing factor 5 | -2.10 | 0.009 | -1.98 | 0.015 | -2.11 | 0.009 | -1.35 | 0.236 |
| A_70_P049406 | RPL10 | ribosomal protein L10 | 2.34 | 0.042 | 1.47 | 0.328 | 1.52 | 0.285 | -1.62 | 0.223 |
| A_70_P049492 | SULF2 | sulfatase 2 | 3.03 | 0.000 | 2.70 | 0.001 | 2.38 | 0.002 | 1.58 | 0.064 |
| A_70_P049631 | TLR6 | toll-like receptor 6 | -2.21 | 0.002 | -1.49 | 0.065 | -1.62 | 0.031 | -1.31 | 0.195 |
| A_70_P049632 | TLR6 | toll-like receptor 6 | -2.11 | 0.002 | -1.44 | 0.075 | -1.41 | 0.087 | -1.15 | 0.480 |
| A_70_P049636 | TLR5 | toll-like receptor 5 | -2.39 | 0.001 | -1.80 | 0.009 | -2.41 | 0.001 | -1.46 | 0.066 |
| A_70_P049641 | TLR10 | toll-like receptor 10 | -3.40 | 0.002 | -2.26 | 0.023 | -1.75 | 0.099 | -1.15 | 0.669 |
| A_70_P049642 | TLR10 | toll-like receptor 10 | -3.69 | 0.001 | -2.55 | 0.011 | -1.89 | 0.063 | -1.25 | 0.488 |
| A_70_P049647 | TLR7 | toll-like receptor 7 | -1.69 | 0.012 | -1.11 | 0.581 | 1.56 | 0.028 | 2.15 | 0.001 |
| A_70_P049651 | NKX2-2 | NK2 homeobox 2 | -2.23 | 0.003 | -1.56 | 0.065 | -2.17 | 0.004 | -1.50 | 0.091 |
| A_70_P049652 | NKX2-2 | NK2 homeobox 2 | -2.19 | 0.004 | -1.59 | 0.058 | -2.21 | 0.004 | -1.52 | 0.080 |
| A_70_P049681 | angpt2 | angiopoietin 2 | -2.27 | 0.010 | -1.41 | 0.219 | -1.19 | 0.535 | 1.05 | 0.862 |
| A_70_P049682 | ANGPT2 | angiopoietin 2 | -2.06 | 0.016 | -1.48 | 0.156 | -1.23 | 0.438 | 1.03 | 0.922 |
| A_70_P049691 | ANGPT1 | angiopoietin 1 | -2.97 | 0.000 | -2.05 | 0.003 | -1.26 | 0.261 | 1.24 | 0.295 |
| A_70_P049921 | BCL2L15 | BCL2-like 15 | -1.29 | 0.411 | 1.71 | 0.101 | 2.42 | 0.013 | 2.45 | 0.012 |
| A_70_P050111 | VCAN | versican | 2.59 | 0.011 | 2.56 | 0.012 | 3.94 | 0.001 | 5.52 | 0.000 |
| A_70_P050131 | ADM | adrenomedullin | -1.41 | 0.230 | -1.38 | 0.258 | -2.13 | 0.016 | -1.43 | 0.211 |
| A_70_P050181 | C1orf110 | chromosome 1 open reading frame 110 | -2.48 | 0.012 | -1.72 | 0.100 | -5.49 | 0.000 | -2.86 | 0.005 |
| A_70_P050246 | HMGA1 | high mobility group AT-hook 1 | 1.51 | 0.089 | 1.56 | 0.072 | 2.31 | 0.003 | -1.33 | 0.228 |

| **Probenames** | **Gene Symbol** | **Gene Name** | **6h vs Baseline**  **Fold**  **Change P** | | **d1 vs Baseline**  **Fold**  **Change P** | | **d3 vs Baseline**  **Fold**  **Change P** | | **d7 vs Baseline**  **Fold**  **Change P** | |
| --- | --- | --- | --- | --- | --- | --- | --- | --- | --- | --- |
| A_70_P050286 | ITGA5 | integrin, alpha 5 (fibronectin receptor, alpha polypeptide) | 1.44 | 0.219 | 1.68 | 0.088 | 2.15 | 0.019 | 2.58 | 0.006 |
| A_70_P050366 | ACTC1 | actin, alpha, cardiac muscle 1 | -2.26 | 0.017 | -2.08 | 0.029 | -2.56 | 0.008 | -2.16 | 0.022 |
| A_70_P050396 | COL12A1 | collagen, type XII, alpha 1 | -1.42 | 0.216 | 1.06 | 0.834 | 1.31 | 0.333 | 3.56 | 0.000 |
| A_70_P050436 | BGN | biglycan | 1.22 | 0.551 | -1.28 | 0.456 | 3.84 | 0.001 | 5.21 | 0.000 |
| A_70_P050491 | ACTC1 | actin, alpha, cardiac muscle 1 | -2.91 | 0.025 | -2.46 | 0.053 | -3.35 | 0.014 | -3.63 | 0.009 |
| A_70_P050591 | COL1A1 | collagen, type I, alpha 1 | 1.33 | 0.414 | 1.21 | 0.582 | 6.38 | 0.000 | 9.35 | 0.000 |
| A_70_P050651 | COL5A2 | collagen, type V, alpha 2 | -1.42 | 0.164 | -1.03 | 0.889 | 2.37 | 0.003 | 3.98 | 0.000 |
| A_70_P050821 | CDKN1A | cyclin-dependent kinase inhibitor 1A (p21, Cip1) | 2.59 | 0.004 | 1.47 | 0.177 | 1.69 | 0.074 | 1.41 | 0.223 |
| A_70_P050827 | APOA1 | apolipoprotein A-I | -3.58 | 0.001 | -1.84 | 0.067 | -1.49 | 0.211 | 1.17 | 0.614 |
| A_70_P050861 | COL3A1 | collagen, type III, alpha 1 | -1.21 | 0.351 | -1.02 | 0.929 | 2.85 | 0.000 | 3.42 | 0.000 |
| A_70_P050917 | TLR2 | toll-like receptor 2 | 2.03 | 0.031 | 1.65 | 0.109 | 1.57 | 0.148 | 1.40 | 0.265 |
| A_70_P050926 | BGN | biglycan | 1.14 | 0.594 | -1.18 | 0.504 | 2.20 | 0.007 | 3.35 | 0.000 |
| A_70_P050966 | IGFBP2 | insulin-like growth factor binding protein 2, 36kDa | -1.51 | 0.235 | 1.46 | 0.274 | 4.43 | 0.001 | 3.60 | 0.002 |
| A_70_P050967 | IGFBP2 | insulin-like growth factor binding protein 2, 36kDa | -1.62 | 0.160 | 1.45 | 0.265 | 4.35 | 0.001 | 3.65 | 0.002 |
| A_70_P050991 | GNLY | granulysin | -7.18 | 0.004 | -4.11 | 0.025 | -4.32 | 0.021 | -1.75 | 0.331 |
| A_70_P050992 | GNLY | granulysin | -7.50 | 0.003 | -4.01 | 0.025 | -4.29 | 0.020 | -1.79 | 0.308 |
| A_70_P051002 | ALB | albumin | -2.16 | 0.015 | -1.43 | 0.214 | -1.36 | 0.274 | -1.59 | 0.113 |

| **Probenames** | **Gene Symbol** | **Gene Name** | **6h vs Baseline**  **Fold**  **Change P** | | **d1 vs Baseline**  **Fold**  **Change P** | | **d3 vs Baseline**  **Fold**  **Change P** | | **d7 vs Baseline**  **Fold**  **Change P** | |
| --- | --- | --- | --- | --- | --- | --- | --- | --- | --- | --- |
| A_70_P051036 | SLC2A5 | solute carrier family 2 (facilitated glucose/fructose transporter), member 5" | -1.37 | 0.049 | 1.18 | 0.264 | 1.58 | 0.008 | 2.24 | 0.000 |
| A_70_P051037 | SLC2A5 | solute carrier family 2 (facilitated glucose/fructose transporter), member 5" | -1.37 | 0.086 | 1.21 | 0.282 | 1.62 | 0.014 | 2.47 | 0.000 |
| A_70_P051056 | INHBA | inhibin, beta A | 2.63 | 0.001 | 2.14 | 0.006 | 2.09 | 0.008 | 1.56 | 0.078 |
| A_70_P051057 | INHBA | inhibin, beta A | 2.61 | 0.002 | 2.13 | 0.008 | 2.11 | 0.009 | 1.57 | 0.084 |
| A_70_P051091 | IGF1 | insulin-like growth factor 1 (somatomedin C) | 1.37 | 0.152 | 2.56 | 0.001 | 5.55 | 0.000 | 2.40 | 0.001 |
| A_70_P051096 | TOM | elafin | -1.15 | 0.667 | -1.13 | 0.703 | 5.26 | 0.000 | 3.11 | 0.004 |
| A_70_P051097 | TOM | elafin | -1.16 | 0.635 | -1.11 | 0.727 | 4.91 | 0.000 | 2.89 | 0.004 |
| A_70_P051106 | SLC2A3 | solute carrier family 2 (facilitated glucose transporter), member 3" | 2.48 | 0.001 | 2.41 | 0.001 | 2.30 | 0.002 | 1.35 | 0.181 |
| A_70_P051107 | SLC2A3 | solute carrier family 2 (facilitated glucose transporter), member 3" | 2.23 | 0.005 | 2.35 | 0.003 | 2.16 | 0.006 | 1.35 | 0.220 |
| A_70_P051126 | PTPLA | protein tyrosine phosphatase-like (proline instead of catalytic | -2.00 | 0.005 | -1.43 | 0.102 | -1.20 | 0.375 | -1.67 | 0.024 |
| A_70_P051127 | PTPLA | protein tyrosine phosphatase-like (proline instead of catalytic | -2.06 | 0.004 | -1.50 | 0.068 | -1.22 | 0.356 | -1.62 | 0.035 |
| A_70_P051176 | IL1A | interleukin 1, alpha | 1.02 | 0.941 | -1.28 | 0.445 | 1.78 | 0.094 | 4.03 | 0.001 |
| A_70_P051177 | IL1A | interleukin 1, alpha | 1.10 | 0.722 | -1.21 | 0.472 | 1.68 | 0.068 | 3.37 | 0.001 |
| A_70_P051181 | HSD11B2 | hydroxysteroid (11-beta) dehydrogenase 2 | -2.07 | 0.002 | -1.90 | 0.004 | -1.18 | 0.382 | -1.12 | 0.533 |
| A_70_P051196 | CSN3 | casein kappa | 2.96 | 0.000 | 3.09 | 0.000 | 1.19 | 0.400 | -1.19 | 0.414 |
| A_70_P051201 | GHRL | ghrelin/obestatin prepropeptide | -2.22 | 0.154 | -2.95 | 0.061 | -4.32 | 0.016 | -12.99 | 0.000 |
| A_70_P051202 | GHRL | ghrelin/obestatin prepropeptide | -2.56 | 0.141 | -3.15 | 0.079 | -5.16 | 0.018 | -16.04 | 0.001 |

| **Probenames** | **Gene Symbol** | **Gene Name** | **6h vs Baseline**  **Fold**  **Change P** | | **d1 vs Baseline**  **Fold**  **Change P** | | **d3 vs Baseline**  **Fold**  **Change P** | | **d7 vs Baseline**  **Fold**  **Change P** | |
| --- | --- | --- | --- | --- | --- | --- | --- | --- | --- | --- |
| A_70_P051231 | LGALS1 | lectin, galactoside-binding, soluble, 1 | -1.18 | 0.412 | 1.22 | 0.334 | 2.20 | 0.002 | 1.02 | 0.912 |
| A_70_P051236 | LOC443348 | pregnancy-specific antigen | -1.64 | 0.013 | -2.38 | 0.000 | -1.92 | 0.002 | -1.76 | 0.006 |
| A_70_P051237 | LOC443348 | pregnancy-specific antigen | -1.67 | 0.012 | -2.83 | 0.000 | -2.05 | 0.001 | -1.78 | 0.006 |
| A_70_P051261 | HBB | hemoglobin, beta" | -2.26 | 0.030 | -1.98 | 0.062 | -2.64 | 0.013 | -1.36 | 0.372 |
| A_70_P051276 | SP-A | pulmonary surfactant-associated protein A | -32.34 | 0.006 | -7.99 | 0.072 | -12.61 | 0.033 | -4.63 | 0.171 |
| A_70_P051277 | SP-A | pulmonary surfactant-associated protein A | -37.37 | 0.005 | -9.50 | 0.056 | -14.51 | 0.027 | -4.85 | 0.163 |
| A_70_P051291 | IL12A | interleukin 12A (natural killer cell stimulatory factor 1, cytotoxic lymphocyte maturation factor 1, p35)" | -1.97 | 0.013 | -1.95 | 0.014 | -2.18 | 0.006 | -1.17 | 0.514 |
| A_70_P051292 | IL12A | interleukin 12A (natural killer cell stimulatory factor 1, cytotoxic lymphocyte maturation factor 1, p35)" | -1.98 | 0.017 | -1.97 | 0.018 | -2.09 | 0.011 | -1.19 | 0.500 |
| A_70_P051322 | SERT | serotonin transporter | -1.63 | 0.056 | -1.70 | 0.039 | -2.07 | 0.008 | -1.03 | 0.890 |
| A_70_P051351 | PYGM | phosphorylase, glycogen, muscle | -2.31 | 0.002 | 1.10 | 0.672 | -1.11 | 0.649 | -1.37 | 0.173 |
| A_70_P051356 | AQP1 | aquaporin 1 (Colton blood group) | -1.55 | 0.043 | -1.73 | 0.015 | -2.11 | 0.002 | -1.49 | 0.062 |
| A_70_P051357 | AQP1 | aquaporin 1 (Colton blood group) | -1.54 | 0.058 | -1.70 | 0.023 | -2.15 | 0.003 | -1.49 | 0.073 |
| A_70_P051411 | TIMP-1 | TIMP-1 protein | 3.31 | 0.000 | 5.33 | 0.000 | 5.36 | 0.000 | 1.88 | 0.021 |
| A_70_P051412 | TIMP-1 | TIMP-1 protein | 3.19 | 0.000 | 4.87 | 0.000 | 4.89 | 0.000 | 1.78 | 0.025 |
| A_70_P051421 | TGFB1 | transforming growth factor, beta 1 | -1.24 | 0.447 | 1.09 | 0.760 | 2.36 | 0.008 | 2.39 | 0.007 |
| A_70_P051471 | ADAM19 | ADAM metallopeptidase domain 19 (meltrin beta) | -1.28 | 0.170 | 1.12 | 0.522 | 2.50 | 0.000 | 2.62 | 0.000 |
| A_70_P051486 | CDC45 | cell division cycle 45 homolog (S. cerevisiae) | -1.23 | 0.213 | 4.51 | 0.000 | 2.36 | 0.000 | 1.53 | 0.018 |

| **Probenames** | **Gene Symbol** | **Gene Name** | **6h vs Baseline**  **Fold**  **Change P** | | **d1 vs Baseline**  **Fold**  **Change P** | | **d3 vs Baseline**  **Fold**  **Change P** | | **d7 vs Baseline**  **Fold**  **Change P** | |
| --- | --- | --- | --- | --- | --- | --- | --- | --- | --- | --- |
| A_70_P051546 | SLC7A1 | solute carrier family 7 (cationic amino acid transporter, y+ system), member 1 | 2.58 | 0.006 | 2.63 | 0.005 | 2.19 | 0.017 | 1.25 | 0.441 |
| A_70_P051571 | CDT1 | chromatin licensing and DNA replication factor 1 | -1.47 | 0.031 | 2.31 | 0.000 | 1.72 | 0.005 | 1.19 | 0.290 |
| A_70_P051601 | CDKN2D | cyclin-dependent kinase inhibitor 2D (p19, inhibits CDK4) | -1.36 | 0.136 | -1.12 | 0.581 | 2.00 | 0.004 | 1.16 | 0.465 |
| A_70_P051636 | MMD | monocyte to macrophage differentiation-associated | -2.11 | 0.002 | -1.58 | 0.030 | -1.01 | 0.945 | 1.51 | 0.045 |
| A_70_P051681 | TWISTNB | TWIST neighbor | 2.02 | 0.022 | 1.75 | 0.058 | 1.75 | 0.058 | -1.93 | 0.030 |
| A_70_P051776 | EPHX2 | epoxide hydrolase 2, cytoplasmic | -1.96 | 0.008 | -2.00 | 0.007 | -2.01 | 0.007 | -1.66 | 0.036 |
| A_70_P051846 | PDLIM3 | PDZ and LIM domain 3 | -2.22 | 0.014 | -2.17 | 0.016 | -2.29 | 0.011 | -2.39 | 0.008 |
| A_70_P051851 | CLIC4 | chloride intracellular channel 4 | 2.11 | 0.001 | 2.04 | 0.001 | 1.51 | 0.025 | 1.28 | 0.148 |
| A_70_P051896 | HCLS1 | hematopoietic cell-specific Lyn substrate 1 | -2.03 | 0.010 | -1.29 | 0.295 | -1.00 | 1.000 | 1.62 | 0.061 |
| A_70_P051961 | NEIL2 | nei endonuclease VIII-like 2 (E. coli) | -2.22 | 0.004 | -1.41 | 0.154 | -1.53 | 0.084 | -1.48 | 0.104 |
| A_70_P051991 | TCF19 | transcription factor 19 | -1.28 | 0.091 | 3.16 | 0.000 | 2.12 | 0.000 | 1.66 | 0.003 |
| A_70_P052081 | RACGAP1P | Rac GTPase activating protein 1 pseudogene | -1.38 | 0.047 | 3.41 | 0.000 | 3.76 | 0.000 | 2.17 | 0.000 |
| A_70_P052086 | CEP72 | centrosomal protein 72kDa | -1.22 | 0.454 | 2.59 | 0.003 | 2.56 | 0.003 | 1.25 | 0.408 |
| A_70_P052096 | DCTPP1 | dCTP pyrophosphatase 1 | 1.77 | 0.031 | 3.01 | 0.001 | 2.03 | 0.011 | -1.19 | 0.479 |
| A_70_P052281 | DOCK8 | dedicator of cytokinesis 8 | -2.26 | 0.000 | -1.51 | 0.016 | -1.40 | 0.041 | -1.06 | 0.687 |
| A_70_P052336 | MCM3 | minichromosome maintenance complex component 3 | -1.24 | 0.364 | 3.29 | 0.000 | 1.60 | 0.061 | 1.37 | 0.191 |
| A_70_P052371 | CRADD | CASP2 and RIPK1 domain containing adaptor with death domain | -2.06 | 0.001 | -1.31 | 0.122 | -1.33 | 0.108 | -1.39 | 0.066 |

| **Probenames** | **Gene Symbol** | **Gene Name** | **6h vs Baseline**  **Fold**  **Change P** | | **d1 vs Baseline**  **Fold**  **Change P** | | **d3 vs Baseline**  **Fold**  **Change P** | | **d7 vs Baseline**  **Fold**  **Change P** | |
| --- | --- | --- | --- | --- | --- | --- | --- | --- | --- | --- |
| A_70_P052381 | MAPK11 | mitogen-activated protein kinase 11 | -2.10 | 0.001 | -1.17 | 0.366 | 1.20 | 0.279 | 1.10 | 0.578 |
| A_70_P052456 | AMPD2 | adenosine monophosphate deaminase 2 | 2.08 | 0.004 | 2.18 | 0.002 | 1.61 | 0.038 | 1.37 | 0.149 |
| A_70_P052486 | TSPAN7 | tetraspanin 7 | -2.00 | 0.023 | -1.45 | 0.191 | -1.53 | 0.135 | -1.05 | 0.852 |
| A_70_P052506 | APOOL | apolipoprotein O-like | -2.00 | 0.001 | -1.15 | 0.415 | 1.08 | 0.661 | -1.22 | 0.243 |
| A_70_P052556 | RELB | v-rel reticuloendotheliosis viral oncogene homolog B | 2.01 | 0.003 | 1.52 | 0.047 | 1.62 | 0.025 | 1.11 | 0.592 |
| A_70_P052566 | IQCD | IQ motif containing D | -2.58 | 0.019 | -1.74 | 0.138 | -2.40 | 0.027 | -1.26 | 0.526 |
| A_70_P052601 | EPAS1 | endothelial PAS domain protein 1 | -2.20 | 0.013 | -2.27 | 0.011 | -1.78 | 0.056 | -1.62 | 0.102 |
| A_70_P052621 | PPA1 | pyrophosphatase (inorganic) 1 | 1.96 | 0.001 | 2.11 | 0.001 | 1.45 | 0.041 | -1.06 | 0.708 |
| A_70_P052661 | CDH5 | cadherin 5, type 2 (vascular endothelium) | -2.83 | 0.003 | -1.30 | 0.359 | -1.10 | 0.732 | 1.54 | 0.146 |
| A_70_P052691 | LIX1L | Lix1 homolog (mouse)-like | -3.29 | 0.000 | -1.98 | 0.003 | -1.26 | 0.231 | 1.02 | 0.895 |
| A_70_P052721 | PTPN13 | protein tyrosine phosphatase, non-receptor type 13 (APO-1/CD95 ( | -2.01 | 0.022 | -1.53 | 0.136 | -1.54 | 0.132 | -1.20 | 0.507 |
| A_70_P052746 | ARHGEF2 | Rho/Rac guanine nucleotide exchange factor (GEF) 2 | -2.20 | 0.004 | -1.68 | 0.037 | -1.35 | 0.197 | 1.10 | 0.676 |
| A_70_P052821 | RBP4 | retinol binding protein 4, plasma | 1.03 | 0.891 | 1.13 | 0.634 | 2.47 | 0.003 | 1.52 | 0.114 |
| A_70_P052866 | ID2 | inhibitor of DNA binding 2, dominant negative helix-loop-helix p | -2.56 | 0.000 | -2.00 | 0.003 | -1.32 | 0.155 | -1.14 | 0.498 |
| A_70_P052906 | CCDC69 | coiled-coil domain containing 69 | -2.17 | 0.001 | -1.61 | 0.025 | -1.68 | 0.016 | -1.47 | 0.059 |
| A_70_P052916 | COL4A6 | collagen, type IV, alpha 6 | -2.19 | 0.007 | -1.99 | 0.015 | -1.46 | 0.143 | -1.74 | 0.040 |
| A_70_P052991 | ERLIN2 | ER lipid raft associated 2 | -2.37 | 0.001 | -1.44 | 0.108 | -1.33 | 0.195 | -1.25 | 0.314 |
| A_70_P053031 | UNC119 | unc-119 homolog (C. elegans) | -2.35 | 0.000 | -1.66 | 0.006 | -1.14 | 0.410 | -1.25 | 0.178 |

| **Probenames** | **Gene Symbol** | **Gene Name** | **6h vs Baseline**  **Fold**  **Change P** | | **d1 vs Baseline**  **Fold**  **Change P** | | **d3 vs Baseline**  **Fold**  **Change P** | | **d7 vs Baseline**  **Fold**  **Change P** | |
| --- | --- | --- | --- | --- | --- | --- | --- | --- | --- | --- |
| A_70_P053051 | STK19 | serine/threonine kinase 19 | -2.29 | 0.001 | -2.00 | 0.004 | -2.03 | 0.003 | -1.50 | 0.062 |
| A_70_P053151 | NR2F1 | nuclear receptor subfamily 2, group F, member 1 | -3.83 | 0.000 | -2.34 | 0.003 | -1.72 | 0.039 | -1.14 | 0.583 |
| A_70_P053281 | GNB4 | guanine nucleotide binding protein (G protein), beta polypeptide | -2.28 | 0.000 | -1.14 | 0.455 | 1.37 | 0.090 | 1.73 | 0.007 |
| A_70_P053306 | NOSTRIN | nitric oxide synthase trafficker | -2.37 | 0.001 | -1.51 | 0.073 | -1.49 | 0.082 | -1.52 | 0.069 |
| A_70_P053386 | MAGI1 | membrane associated guanylate kinase, WW and PDZ domain containi | -2.70 | 0.000 | -1.73 | 0.006 | -1.69 | 0.008 | -1.40 | 0.063 |
| A_70_P053411 | PLAT | plasminogen activator, tissue | 2.11 | 0.003 | 1.62 | 0.036 | 2.98 | 0.000 | 1.75 | 0.018 |
| A_70_P053451 | FAM35B2 | Family with sequence similarity 35, member A | -2.29 | 0.000 | -1.59 | 0.010 | -1.68 | 0.005 | -1.43 | 0.037 |
| A_70_P053551 | TMEM126A | transmembrane protein 126A | 1.67 | 0.020 | 2.08 | 0.002 | 1.63 | 0.025 | -1.30 | 0.193 |
| A_70_P053751 | ZBED5 | zinc finger, BED-type containing 5 | -2.20 | 0.000 | -1.65 | 0.010 | -1.35 | 0.098 | -1.24 | 0.221 |
| A_70_P053771 | RASL11B | RAS-like, family 11, member B | -1.31 | 0.241 | -1.64 | 0.045 | 2.20 | 0.004 | 1.47 | 0.109 |
| A_70_P053921 | ESPL1 | extra spindle pole bodies homolog 1 (S. cerevisiae) | -1.96 | 0.008 | 3.25 | 0.000 | 3.36 | 0.000 | 1.55 | 0.062 |
| A_70_P053936 | CCNB3 | cyclin B3 | -1.77 | 0.000 | 2.32 | 0.000 | 2.61 | 0.000 | 1.40 | 0.014 |
| A_70_P054071 | RASGRP2 | RAS guanyl releasing protein 2 (calcium and DAG-regulated) | -2.14 | 0.002 | -1.70 | 0.018 | -1.50 | 0.059 | -1.22 | 0.327 |
| A_70_P054151 | MAP4K2 | mitogen-activated protein kinase kinase kinase kinase 2 | -2.07 | 0.001 | -1.35 | 0.082 | -1.15 | 0.403 | 1.03 | 0.843 |
| A_70_P054236 | LTBP4 | latent transforming growth factor beta binding protein 4 | -1.63 | 0.152 | -3.43 | 0.002 | -2.47 | 0.015 | -1.08 | 0.825 |

| **Probenames** | **Gene Symbol** | **Gene Name** | **6h vs Baseline**  **Fold**  **Change P** | | **d1 vs Baseline**  **Fold**  **Change P** | | **d3 vs Baseline**  **Fold**  **Change P** | | **d7 vs Baseline**  **Fold**  **Change P** | |
| --- | --- | --- | --- | --- | --- | --- | --- | --- | --- | --- |
| A_70_P054421 | CCNB1 | cyclin B1 | -1.21 | 0.255 | 7.78 | 0.000 | 7.78 | 0.000 | 2.87 | 0.000 |
| A_70_P054441 | LPCAT4 | lysophosphatidylcholine acyltransferase 4 | -2.20 | 0.005 | -1.38 | 0.184 | -1.63 | 0.057 | -1.26 | 0.334 |
| A_70_P054546 | BYSL | bystin-like | 2.03 | 0.001 | 1.79 | 0.004 | 1.10 | 0.561 | -1.03 | 0.860 |
| A_70_P054641 | DPYS | dihydropyrimidinase | -1.99 | 0.009 | -1.70 | 0.034 | -3.48 | 0.000 | -2.44 | 0.002 |
| A_70_P054651 | NPNT | nephronectin | -2.35 | 0.005 | -1.91 | 0.023 | -1.68 | 0.059 | -1.37 | 0.231 |
| A_70_P054661 | AMBP | alpha-1-microglobulin/bikunin precursor | 2.14 | 0.030 | 1.44 | 0.263 | 1.66 | 0.126 | -1.26 | 0.470 |
| A_70_P054711 | AGP | alpha-1 acid glycoprotein | 1.16 | 0.659 | 1.93 | 0.070 | 8.20 | 0.000 | 13.60 | 0.000 |
| A_70_P054756 | APOC2 | apolipoprotein C-II | -2.48 | 0.003 | -1.43 | 0.167 | -1.77 | 0.036 | -1.98 | 0.016 |
| A_70_P054781 | LAYN | layilin | -2.92 | 0.000 | -1.82 | 0.006 | -2.12 | 0.001 | -1.48 | 0.052 |
| A_70_P054871 | DYNLL2 | dynein, light chain, LC8-type 2 | -1.35 | 0.124 | -1.41 | 0.084 | -2.36 | 0.000 | -1.95 | 0.003 |
| A_70_P054881 | TCP11L2 | t-complex 11 (mouse)-like 2 | -2.62 | 0.001 | -2.72 | 0.001 | -1.99 | 0.011 | -1.31 | 0.265 |
| A_70_P054926 | AC135457.2 | NULL | -1.15 | 0.547 | 2.43 | 0.002 | 3.37 | 0.000 | 1.57 | 0.069 |
| A_70_P054951 | NEK6 | NIMA (never in mitosis gene a)-related kinase 6 | 1.98 | 0.049 | 2.09 | 0.035 | 2.20 | 0.026 | 1.26 | 0.468 |
| A_70_P054966 | SH2D1A | SH2 domain containing 1A | -3.21 | 0.001 | -2.01 | 0.023 | -1.32 | 0.319 | 1.31 | 0.341 |
| A_70_P054986 | LCK | lymphocyte-specific protein tyrosine kinase | -3.09 | 0.001 | -1.97 | 0.023 | -1.63 | 0.084 | 1.10 | 0.722 |
| A_70_P055006 | HACL1 | 2-hydroxyacyl-CoA lyase 1 | -2.13 | 0.005 | -1.31 | 0.240 | -1.37 | 0.179 | -1.34 | 0.203 |
| A_70_P055096 | SNRPG | small nuclear ribonucleoprotein polypeptide G | 1.86 | 0.009 | 2.05 | 0.004 | 2.20 | 0.002 | -1.27 | 0.262 |
| A_70_P055161 | RAD51 | RAD51 homolog (RecA homolog, E. coli) (S. cerevisiae) | -1.26 | 0.054 | 4.87 | 0.000 | 3.59 | 0.000 | 1.85 | 0.000 |
| A_70_P055171 | IVD | isovaleryl-CoA dehydrogenase | -2.31 | 0.001 | -1.48 | 0.059 | -1.57 | 0.034 | -1.38 | 0.108 |

| **Probenames** | **Gene Symbol** | **Gene Name** | **6h vs Baseline**  **Fold**  **Change P** | | **d1 vs Baseline**  **Fold**  **Change P** | | **d3 vs Baseline**  **Fold**  **Change P** | | **d7 vs Baseline**  **Fold**  **Change P** | |
| --- | --- | --- | --- | --- | --- | --- | --- | --- | --- | --- |
| A_70_P055236 | SYCP3 | kinetochore associated 1 | -3.06 | 0.000 | -2.32 | 0.002 | -2.59 | 0.001 | -1.70 | 0.028 |
| A_70_P055261 | C1QTNF3 | C1q and tumor necrosis factor related protein 3 | 1.50 | 0.289 | -2.10 | 0.067 | 2.16 | 0.058 | 6.95 | 0.000 |
| A_70_P055316 | EXOSC4 | exosome component 4 | 1.79 | 0.008 | 2.26 | 0.001 | 1.76 | 0.009 | -1.07 | 0.730 |
| A_70_P055351 | CD36 | CD36 molecule (thrombospondin receptor) | -3.60 | 0.000 | -1.40 | 0.099 | -1.51 | 0.046 | -1.65 | 0.020 |
| A_70_P055361 | TCTN2 | tectonic family member 2 | -2.28 | 0.002 | -1.76 | 0.018 | -2.10 | 0.004 | -1.58 | 0.048 |
| A_70_P055661 | CKS1B | CDC28 protein kinase regulatory subunit 1B | 1.13 | 0.483 | 2.32 | 0.000 | 2.09 | 0.001 | -1.03 | 0.850 |
| A_70_P055736 | PLAU | plasminogen activator, urokinase | 1.75 | 0.125 | 2.79 | 0.010 | 2.94 | 0.008 | 3.45 | 0.003 |
| A_70_P055766 | MXRA7 | matrix-remodelling associated 7 | -1.89 | 0.004 | -2.10 | 0.001 | -1.61 | 0.022 | -1.55 | 0.031 |
| A_70_P055806 | AURKA | aurora kinase A | -1.04 | 0.797 | 4.63 | 0.000 | 5.02 | 0.000 | 2.21 | 0.000 |
| A_70_P055821 | JAM3 | junctional adhesion molecule 3 | -2.13 | 0.002 | -1.94 | 0.004 | -1.87 | 0.006 | -1.42 | 0.086 |
| A_70_P055871 | GRINL1A | glutamate receptor, ionotropic, N-methyl D-aspartate-like 1A | -2.53 | 0.013 | -1.69 | 0.124 | -2.22 | 0.028 | -1.12 | 0.728 |
| A_70_P055911 | ALOX5 | arachidonate 5-lipoxygenase | -2.20 | 0.001 | -1.44 | 0.084 | -2.09 | 0.002 | -1.07 | 0.727 |
| A_70_P055976 | IL20RB | interleukin 20 receptor beta | -2.61 | 0.001 | -2.13 | 0.003 | -2.04 | 0.005 | -1.99 | 0.006 |
| A_70_P056061 | GFRA2 | GDNF family receptor alpha 2 | -2.07 | 0.004 | -2.64 | 0.001 | -1.79 | 0.016 | -1.67 | 0.030 |
| A_70_P056106 | WDR76 | WD repeat domain 76 | -2.08 | 0.003 | 1.12 | 0.554 | -1.02 | 0.903 | 1.01 | 0.963 |
| A_70_P056166 | MEF2C | myocyte enhancer factor 2C (MEF2C) | -3.16 | 0.000 | -2.09 | 0.001 | -1.46 | 0.034 | -1.11 | 0.535 |
| A_70_P056186 | UTRN | utrophin | -2.24 | 0.000 | -1.57 | 0.011 | -1.43 | 0.033 | -1.32 | 0.089 |
| A_70_P056251 | PALMD | palmdelphin | -2.32 | 0.003 | -1.69 | 0.036 | -1.76 | 0.026 | -1.69 | 0.035 |
| A_70_P056261 | SERPINB2 | serpin peptidase inhibitor, clade B (ovalbumin) member 2 | 3.20 | 0.021 | 3.80 | 0.010 | 3.86 | 0.010 | 4.15 | 0.007 |
| A_70_P056296 | ITGB4 | integrin, beta 4 | -1.60 | 0.159 | 1.43 | 0.279 | 2.23 | 0.025 | 1.25 | 0.486 |

| **Probenames** | **Gene Symbol** | **Gene Name** | **6h vs Baseline**  **Fold**  **Change P** | | **d1 vs Baseline**  **Fold**  **Change P** | | **d3 vs Baseline**  **Fold**  **Change P** | | **d7 vs Baseline**  **Fold**  **Change P** | |
| --- | --- | --- | --- | --- | --- | --- | --- | --- | --- | --- |
| A_70_P056456 | PDZD2 | PDZ domain containing 2 | -3.24 | 0.003 | -2.64 | 0.009 | -2.91 | 0.005 | -1.37 | 0.330 |
| A_70_P056531 | ZNF34 | zinc finger protein 34 | -2.03 | 0.008 | -1.90 | 0.014 | -2.21 | 0.004 | -1.42 | 0.142 |
| A_70_P056566 | PAX8 | paired box 8 | 1.07 | 0.664 | 1.28 | 0.151 | 2.52 | 0.000 | 1.63 | 0.011 |
| A_70_P056956 | ENOSF1 | enolase superfamily member 1 | -2.02 | 0.001 | -1.39 | 0.069 | -1.33 | 0.105 | -1.36 | 0.089 |
| A_70_P057016 | PCMTD1 | protein-L-isoaspartate (D-aspartate) O-methyltransferase domain | -2.76 | 0.001 | -2.14 | 0.005 | -1.84 | 0.017 | -1.21 | 0.406 |
| A_70_P057096 | CTSS | cathepsin S | -2.33 | 0.003 | -1.06 | 0.811 | 1.67 | 0.046 | 1.57 | 0.073 |
| A_70_P057131 | TXNIP | thioredoxin interacting protein | -1.39 | 0.167 | -1.55 | 0.071 | -2.29 | 0.003 | -1.22 | 0.393 |
| A_70_P057151 | MYLK | myosin light chain kinase | -1.28 | 0.259 | -1.27 | 0.274 | -1.74 | 0.023 | -2.21 | 0.003 |
| A_70_P057156 | CYBA | cytochrome b-245, alpha polypeptide | -2.03 | 0.006 | -1.30 | 0.245 | -1.43 | 0.114 | -1.87 | 0.012 |
| A_70_P057157 | CYBA | cytochrome b-245, alpha polypeptide | -2.01 | 0.007 | -1.30 | 0.243 | -1.38 | 0.157 | -1.84 | 0.014 |
| A_70_P057161 | CTSF | cathepsin F | -2.07 | 0.002 | -2.33 | 0.001 | -1.74 | 0.011 | -1.28 | 0.200 |
| A_70_P057162 | CTSF | cathepsin F | -2.07 | 0.001 | -2.27 | 0.001 | -1.72 | 0.010 | -1.28 | 0.187 |
| A_70_P057166 | NKG7 | natural killer cell group 7 sequence | -3.08 | 0.006 | -2.21 | 0.036 | -1.57 | 0.201 | 1.42 | 0.316 |
| A_70_P057201 | KNTC1 | kinetochore associated 1 | -1.86 | 0.001 | 6.75 | 0.000 | 4.92 | 0.000 | 2.32 | 0.000 |
| A_70_P057311 | CXCL13 | chemokine (C-X-C motif) ligand 13 | -2.31 | 0.048 | -1.08 | 0.852 | 4.35 | 0.002 | 1.68 | 0.197 |
| A_70_P057361 | INPP5A | inositol polyphosphate-5-phosphatase, 40kDa | -2.11 | 0.003 | -1.88 | 0.009 | -2.04 | 0.004 | -1.85 | 0.010 |
| A_70_P057421 | MAK16 | MAK16 homolog (S. cerevisiae) | 2.21 | 0.001 | 2.03 | 0.003 | 1.58 | 0.032 | -1.29 | 0.204 |
| A_70_P057521 | ITGB7 | integrin, beta 7 | -2.26 | 0.006 | -1.54 | 0.104 | -1.46 | 0.154 | -1.08 | 0.753 |
| A_70_P057616 | UQCR11 | ubiquinol-cytochrome c reductase, complex III subunit XI | 1.21 | 0.459 | 1.92 | 0.020 | 2.11 | 0.010 | -1.52 | 0.110 |
| A_70_P057651 | CCDC109B | coiled-coil domain containing 109B | 1.31 | 0.034 | 2.47 | 0.000 | 2.77 | 0.000 | 2.23 | 0.000 |
| A_70_P057656 | LOC524694 | Paladin (LOC524694) | -3.69 | 0.000 | -2.72 | 0.002 | -1.52 | 0.124 | -1.10 | 0.716 |

| **Probenames** | **Gene Symbol** | **Gene Name** | **6h vs Baseline**  **Fold**  **Change P** | | **d1 vs Baseline**  **Fold**  **Change P** | | **d3 vs Baseline**  **Fold**  **Change P** | | **d7 vs Baseline**  **Fold**  **Change P** | |
| --- | --- | --- | --- | --- | --- | --- | --- | --- | --- | --- |
| A_70_P057756 | SIRT3 | sirtuin 3 | -2.50 | 0.001 | -1.70 | 0.021 | -1.82 | 0.011 | -1.53 | 0.055 |
| A_70_P057786 | RCSD1 | RCSD domain containing 1 | -3.98 | 0.000 | -2.11 | 0.016 | -1.23 | 0.456 | 1.15 | 0.600 |
| A_70_P057871 | TMEM80 | transmembrane protein 80 | -2.19 | 0.000 | -1.66 | 0.003 | -1.77 | 0.001 | -2.02 | 0.000 |
| A_70_P057946 | PRODH | proline dehydrogenase (oxidase) 1 (PRODH), nuclear gene encoding mitochondrial protein | -2.95 | 0.000 | -2.96 | 0.000 | -4.08 | 0.000 | -3.88 | 0.000 |
| A_70_P057961 | RGS10 | regulator of G-protein signaling 10 | -2.12 | 0.011 | -1.15 | 0.590 | 1.57 | 0.096 | 1.50 | 0.126 |
| A_70_P057981 | GATA3 | GATA binding protein 3 | -3.32 | 0.002 | -2.75 | 0.007 | -2.86 | 0.006 | -1.70 | 0.117 |
| A_70_P058076 | CCDC34 | coiled-coil domain containing 34 | -1.32 | 0.132 | 1.53 | 0.029 | 2.01 | 0.002 | -1.39 | 0.081 |
| A_70_P058166 | ITGA8 | integrin, alpha 8 | -2.67 | 0.008 | -2.85 | 0.005 | -3.72 | 0.001 | -2.04 | 0.038 |
| A_70_P058171 | FAM171A1 | family with sequence similarity 171, member A1 | -2.14 | 0.003 | -1.30 | 0.217 | -1.35 | 0.158 | -1.53 | 0.057 |
| A_70_P058276 | KRT15 | keratin 15 | -2.52 | 0.010 | -1.77 | 0.082 | -1.41 | 0.273 | -1.93 | 0.049 |
| A_70_P058311 | GPR116 | G protein-coupled receptor 116 transcript variant 3 | -6.02 | 0.000 | -3.33 | 0.003 | -1.92 | 0.068 | -1.34 | 0.382 |
| A_70_P058376 | FAM105A | family with sequence similarity 105, member A | -2.19 | 0.000 | -1.99 | 0.000 | -1.17 | 0.263 | 1.01 | 0.961 |
| A_70_P058491 | ENPP1 | ectonucleotide pyrophosphatase/phosphodiesterase 1 | 1.31 | 0.135 | 1.65 | 0.011 | 2.02 | 0.001 | 2.30 | 0.000 |
| A_70_P058566 | PYCR1 | pyrroline-5-carboxylate reductase 1 | 2.30 | 0.025 | 3.03 | 0.005 | 2.95 | 0.006 | 2.44 | 0.018 |
| A_70_P058626 | VWF | von Willebrand factor | -1.57 | 0.039 | -1.03 | 0.869 | 2.13 | 0.002 | 1.42 | 0.098 |
| A_70_P058651 | CRABP1 | cellular retinoic acid binding protein 1 | -1.21 | 0.586 | 1.26 | 0.507 | 3.26 | 0.005 | 1.87 | 0.090 |
| A_70_P058701 | CD74 | CD74 molecule, major histocompatibility complex, class II invari | -2.13 | 0.003 | -1.52 | 0.066 | -1.02 | 0.918 | 1.09 | 0.669 |

| **Probenames** | **Gene Symbol** | **Gene Name** | **6h vs Baseline**  **Fold**  **Change P** | | **d1 vs Baseline**  **Fold**  **Change P** | | **d3 vs Baseline**  **Fold**  **Change P** | | **d7 vs Baseline**  **Fold**  **Change P** | |
| --- | --- | --- | --- | --- | --- | --- | --- | --- | --- | --- |
| A_70_P058706 | CXCL5 | chemokine (C-X-C motif) ligand 5 (CXCL5) | 11.26 | 0.000 | 14.57 | 0.000 | 18.55 | 0.000 | 6.18 | 0.001 |
| A_70_P058771 | ECM1 | extracellular matrix protein 1 | 1.10 | 0.682 | 1.50 | 0.098 | 2.46 | 0.002 | 1.86 | 0.019 |
| A_70_P058876 | NDUFA4L2 | NADH dehydrogenase (ubiquinone) 1 alpha subcomplex, 4-like 2 | -2.23 | 0.006 | -1.83 | 0.028 | -1.00 | 0.989 | 1.66 | 0.058 |
| A_70_P058951 | TMEM45A | transmembrane protein 45A | 3.12 | 0.001 | 4.16 | 0.000 | 2.89 | 0.002 | 2.35 | 0.007 |
| A_70_P059016 | GM2A | GM2 ganglioside activator | -2.02 | 0.001 | -1.27 | 0.183 | 1.05 | 0.790 | 1.40 | 0.066 |
| A_70_P059061 | LOXL2 | lysyl oxidase-like 2 | -1.37 | 0.202 | 1.09 | 0.727 | 2.35 | 0.003 | 3.14 | 0.000 |
| A_70_P059141 | ENPP2 | ectonucleotide pyrophosphatase/phosphodiesterase 2 | -2.73 | 0.000 | -1.45 | 0.086 | -1.06 | 0.765 | 1.03 | 0.867 |
| A_70_P059301 | APOA1 | apolipoprotein A-I | -3.13 | 0.001 | -1.71 | 0.074 | -1.33 | 0.315 | 1.14 | 0.635 |
| A_70_P059481 | NDRG1 | N-myc downstream regulated 1 | 1.75 | 0.032 | 1.49 | 0.111 | 2.45 | 0.002 | 1.47 | 0.121 |
| A_70_P059526 | FKBP10 | FK506 binding protein 10, 65 kDa | 1.56 | 0.176 | 2.59 | 0.009 | 5.52 | 0.000 | 2.34 | 0.017 |
| A_70_P059556 | SIPA1 | signal-induced proliferation-associated 1 | -2.34 | 0.001 | -1.22 | 0.347 | -1.01 | 0.960 | 1.24 | 0.309 |
| A_70_P059611 | DLL4 | delta-like 4 (Drosophila) | -3.24 | 0.005 | -2.20 | 0.037 | -2.51 | 0.018 | 1.01 | 0.979 |
| A_70_P059651 | PDIA4 | protein disulfide isomerase family A, member 4 | 1.88 | 0.034 | 2.74 | 0.002 | 1.51 | 0.147 | 1.50 | 0.152 |
| A_70_P059676 | CD14 | CD14 molecule | 1.36 | 0.189 | 1.49 | 0.093 | 2.12 | 0.005 | 1.76 | 0.025 |
| A_70_P059686 | CTSS | cathepsin S | -2.20 | 0.010 | -1.41 | 0.207 | 1.67 | 0.071 | 2.15 | 0.012 |
| A_70_P059701 | COL1A2 | collagen, type I, alpha 2 | -1.24 | 0.411 | -1.08 | 0.782 | 3.68 | 0.000 | 6.34 | 0.000 |
| A_70_P059716 | ACTN2 | actinin, alpha 2 | -1.83 | 0.050 | -2.78 | 0.003 | -3.79 | 0.000 | -2.39 | 0.009 |
| A_70_P059756 | COL1A1 | collagen, type I, alpha 1 | 1.18 | 0.577 | 1.08 | 0.807 | 5.04 | 0.000 | 8.21 | 0.000 |
| A_70_P059786 | COL1A2 | collagen, type I, alpha 2 | -1.14 | 0.647 | 1.01 | 0.971 | 4.37 | 0.000 | 6.91 | 0.000 |

| **Probenames** | **Gene Symbol** | **Gene Name** | **6h vs Baseline**  **Fold**  **Change P** | | **d1 vs Baseline**  **Fold**  **Change P** | | **d3 vs Baseline**  **Fold**  **Change P** | | **d7 vs Baseline**  **Fold**  **Change P** | |
| --- | --- | --- | --- | --- | --- | --- | --- | --- | --- | --- |
| A_70_P059801 | DES | desmin | -1.22 | 0.531 | -1.10 | 0.756 | -2.28 | 0.019 | -2.18 | 0.025 |
| A_70_P059811 | HSPA5 | heat shock 70kDa protein 5 (glucose-regulated protein, 78kDa) | 2.68 | 0.004 | 2.62 | 0.004 | 1.24 | 0.450 | 1.25 | 0.426 |
| A_70_P059822 | TUBB | tubulin, beta | 1.04 | 0.858 | 2.27 | 0.001 | 1.47 | 0.073 | 1.46 | 0.077 |
| A_70_P059826 | COL1A2 | collagen, type I, alpha 2 | -1.09 | 0.615 | 1.18 | 0.338 | 3.21 | 0.000 | 2.94 | 0.000 |
| A_70_P059836 | TSKU | tsukushi small leucine rich proteoglycan homolog (Xenopus laevis | -2.74 | 0.001 | -1.91 | 0.017 | -1.52 | 0.097 | -2.19 | 0.006 |
| A_70_P059841 | COL1A2 | collagen, type I, alpha 2 | -1.15 | 0.553 | 1.11 | 0.646 | 3.44 | 0.000 | 4.13 | 0.000 |
| A_70_P059876 | COL5A1 | collagen, type V, alpha 1 | -1.39 | 0.224 | 1.08 | 0.769 | 3.61 | 0.000 | 5.08 | 0.000 |
| A_70_P059886 | COL1A1 | collagen, type I, alpha 1 | 1.25 | 0.546 | 1.45 | 0.320 | 9.39 | 0.000 | 13.36 | 0.000 |
| A_70_P059931 | VCAM1 | vascular cell adhesion molecule 1 | -1.32 | 0.311 | 1.13 | 0.643 | 5.46 | 0.000 | 2.99 | 0.001 |
| A_70_P059936 | PRSS23 | protease, serine, 23 | -2.99 | 0.001 | -2.58 | 0.003 | 1.00 | 0.996 | 3.61 | 0.000 |
| A_70_P059986 | THBS1 | thrombospondin 1 | 2.09 | 0.028 | 1.93 | 0.045 | 1.14 | 0.670 | 1.64 | 0.120 |
| A_70_P060076 | NPC2 | Niemann-Pick disease, type C2 | -2.22 | 0.000 | -1.13 | 0.460 | -1.03 | 0.867 | 1.03 | 0.840 |
| A_70_P060116 | THBS2 | thrombospondin 2 | 1.82 | 0.129 | 2.03 | 0.077 | 5.09 | 0.001 | 10.53 | 0.000 |
| A_70_P060121 | UMPS | uridine monophosphate synthetase | 1.81 | 0.030 | 2.07 | 0.011 | 1.71 | 0.046 | 1.26 | 0.363 |
| A_70_P060136 | CGREF1 | cell growth regulator with EF-hand domain 1 | 1.51 | 0.115 | 3.63 | 0.000 | 3.09 | 0.001 | 1.73 | 0.042 |
| A_70_P060146 | AC005393.1 | NULL | 2.22 | 0.001 | 2.15 | 0.001 | 1.52 | 0.035 | -1.27 | 0.202 |
| A_70_P060186 | HYOU1 | hypoxia up-regulated 1 | 2.73 | 0.001 | 2.75 | 0.001 | 1.30 | 0.280 | 1.14 | 0.594 |
| A_70_P060231 | LEPROT | leptin receptor overlapping transcript | -2.19 | 0.002 | -1.21 | 0.357 | -1.22 | 0.329 | 1.14 | 0.522 |
| A_70_P060351 | TXNDC17 | thioredoxin domain containing 17 | 1.08 | 0.690 | 1.77 | 0.008 | 2.02 | 0.002 | -1.10 | 0.596 |
| A_70_P060406 | RBM5 | RNA binding motif protein 5 | -2.16 | 0.010 | -1.82 | 0.034 | -2.31 | 0.006 | -1.45 | 0.166 |
| A_70_P060511 | KRT79 | keratin 79 | -1.01 | 0.989 | 1.46 | 0.420 | 3.99 | 0.010 | 3.06 | 0.030 |

| **Probenames** | **Gene Symbol** | **Gene Name** | **6h vs Baseline**  **Fold**  **Change P** | | **d1 vs Baseline**  **Fold**  **Change P** | | **d3 vs Baseline**  **Fold**  **Change P** | | **d7 vs Baseline**  **Fold**  **Change P** | |
| --- | --- | --- | --- | --- | --- | --- | --- | --- | --- | --- |
| A_70_P060731 | GLUL | glutamate-ammonia ligase | -1.66 | 0.147 | -1.61 | 0.172 | -2.08 | 0.044 | -2.17 | 0.035 |
| A_70_P060791 | PEG3-AS1 | PEG3 antisense RNA 1 (non-protein coding) | -1.88 | 0.007 | -2.26 | 0.001 | -2.15 | 0.002 | -1.91 | 0.006 |
| A_70_P060846 | SMARCD3 | SWI/SNF related, matrix associated, actin dependent regulator of | -2.10 | 0.002 | -1.79 | 0.009 | -1.93 | 0.004 | -1.63 | 0.021 |
| A_70_P060871 | PI15 | peptidase inhibitor 15 | 1.56 | 0.169 | 2.80 | 0.005 | 1.53 | 0.182 | 2.58 | 0.009 |
| A_70_P060931 | KDR | kinase insert domain receptor (a type III receptor tyrosine kina | -2.21 | 0.002 | -1.16 | 0.481 | -1.18 | 0.449 | 1.22 | 0.352 |
| A_70_P060971 | THY1 | Thy-1 cell surface antigen | -1.29 | 0.291 | -1.72 | 0.038 | 1.57 | 0.078 | 2.76 | 0.001 |
| A_70_P060996 | BGN | biglycan | 1.04 | 0.888 | -1.36 | 0.265 | 2.55 | 0.004 | 2.44 | 0.006 |
| A_70_P061026 | BIRC5 | baculoviral IAP repeat containing 5 | -1.59 | 0.046 | 7.18 | 0.000 | 8.49 | 0.000 | 2.74 | 0.000 |
| A_70_P061031 | COL5A1 | collagen, type V, alpha 1 | 1.00 | 0.984 | 1.12 | 0.632 | 3.23 | 0.000 | 4.27 | 0.000 |
| A_70_P061046 | HBB | hemoglobin, beta | -2.89 | 0.010 | -2.69 | 0.015 | -3.14 | 0.006 | -1.74 | 0.136 |
| A_70_P061066 | CKAP2 | cytoskeleton associated protein 2 | -1.44 | 0.078 | 5.92 | 0.000 | 6.36 | 0.000 | 3.27 | 0.000 |
| A_70_P061211 | DDIT4 | DNA-damage-inducible transcript 4 | -2.23 | 0.003 | -1.86 | 0.015 | -1.72 | 0.029 | -1.76 | 0.023 |
| A_70_P061236 | KIAA0101 | KIAA0101 protein (KIAA0101) | -2.66 | 0.000 | 8.49 | 0.000 | 6.39 | 0.000 | 2.63 | 0.000 |
| A_70_P061256 | NT5DC2 | 5'-nucleotidase domain containing 2 | -1.27 | 0.192 | 1.64 | 0.013 | 2.19 | 0.001 | 2.29 | 0.000 |
| A_70_P061281 | NME7 | non-metastatic cells 7, protein expressed in (nucleoside-diphosp | -2.24 | 0.001 | -1.26 | 0.208 | -1.27 | 0.198 | -1.03 | 0.872 |
| A_70_P061331 | CXCL2 | chemokine (C-X-C motif) ligand 2 | 3.34 | 0.001 | 1.87 | 0.053 | 2.14 | 0.023 | -1.26 | 0.440 |
| A_70_P061341 | C29H11orf10 | chromosome 11 open reading frame 10 ortholog | 1.46 | 0.025 | 2.10 | 0.000 | 1.44 | 0.030 | -1.31 | 0.090 |
| A_70_P061401 | MARCKSL1 | MARCKS-like 1 | 1.12 | 0.591 | 1.18 | 0.437 | 2.42 | 0.001 | 2.16 | 0.003 |
| A_70_P061431 | CKAP4 | cytoskeleton-associated protein 4 | 2.05 | 0.005 | 2.38 | 0.001 | 2.28 | 0.002 | 1.36 | 0.168 |

| **Probenames** | **Gene Symbol** | **Gene Name** | **6h vs Baseline**  **Fold**  **Change P** | | **d1 vs Baseline**  **Fold**  **Change P** | | **d3 vs Baseline**  **Fold**  **Change P** | | **d7 vs Baseline**  **Fold**  **Change P** | |
| --- | --- | --- | --- | --- | --- | --- | --- | --- | --- | --- |
| A_70_P061456 | HMGA1 | high mobility group AT-hook 1 | 1.45 | 0.223 | 1.40 | 0.268 | 2.15 | 0.020 | -1.01 | 0.986 |
| A_70_P061466 | COL1A2 | collagen, type I, alpha 2 | -1.05 | 0.800 | -1.00 | 0.997 | 1.99 | 0.005 | 2.81 | 0.000 |
| A_70_P061496 | MMP2 | matrix metallopeptidase 2 (gelatinase A, 72kDa gelatinase, 72kDa | -1.51 | 0.087 | -1.04 | 0.865 | 2.75 | 0.001 | 2.44 | 0.002 |
| A_70_P061526 | SOD2 | superoxide dismutase 2, mitochondrial | 3.63 | 0.000 | 2.83 | 0.002 | 2.34 | 0.006 | 1.31 | 0.306 |
| A_70_P061556 | CTSF | cathepsin F | -1.94 | 0.002 | -2.19 | 0.001 | -1.68 | 0.011 | -1.27 | 0.184 |
| A_70_P061601 | CENP-A | similar to Histone H3-like centromeric protein A (Centromere protein A) (CENP-A) | 1.05 | 0.657 | 2.09 | 0.000 | 2.04 | 0.000 | 1.50 | 0.004 |
| A_70_P061621 | PDZK1 | PDZ domain containing 1 | -2.23 | 0.039 | -1.47 | 0.290 | -1.18 | 0.637 | -1.57 | 0.220 |
| A_70_P061691 | HPGD | hydroxyprostaglandin dehydrogenase 15-(NAD) | -4.74 | 0.016 | -4.56 | 0.018 | -5.78 | 0.008 | -1.57 | 0.430 |
| A_70_P061696 | CXCR6 | chemokine (C-X-C motif) receptor 6 | -2.95 | 0.005 | -2.24 | 0.025 | -1.94 | 0.057 | 1.16 | 0.645 |
| A_70_P061716 | PSEN1 | presenilin 1 | -1.90 | 0.022 | -1.48 | 0.134 | -2.19 | 0.008 | -1.36 | 0.232 |
| A_70_P061721 | NT5C | 5', 3'-nucleotidase, cytosolic | 1.27 | 0.057 | 2.34 | 0.000 | 1.83 | 0.000 | 1.18 | 0.183 |
| A_70_P061801 | UBE2C | ubiquitin-conjugating enzyme E2C | -1.76 | 0.002 | 8.18 | 0.000 | 9.22 | 0.000 | 2.55 | 0.000 |
| A_70_P061841 | VKORC1L1 | vitamin K epoxide reductase complex, subunit 1-like 1 | 1.35 | 0.028 | 2.13 | 0.000 | 2.14 | 0.000 | 1.42 | 0.012 |
| A_70_P061876 | LRRN4CL | LRRN4 C-terminal like | -2.06 | 0.003 | -1.66 | 0.025 | -1.94 | 0.006 | -1.15 | 0.501 |
| A_70_P061941 | METTL11A | methyltransferase like 11A | -2.02 | 0.001 | -1.49 | 0.033 | -1.76 | 0.005 | -1.55 | 0.021 |
| A_70_P061956 | S100G | S100 calcium binding protein G | -1.35 | 0.277 | 1.37 | 0.263 | 2.52 | 0.005 | 1.03 | 0.918 |
| A_70_P061996 | CD79A | CD79a molecule, immunoglobulin-associated alpha | -2.59 | 0.006 | -1.79 | 0.067 | 1.36 | 0.303 | 1.04 | 0.883 |

| **Probenames** | **Gene Symbol** | **Gene Name** | **6h vs Baseline**  **Fold**  **Change P** | | **d1 vs Baseline**  **Fold**  **Change P** | | **d3 vs Baseline**  **Fold**  **Change P** | | **d7 vs Baseline**  **Fold**  **Change P** | |
| --- | --- | --- | --- | --- | --- | --- | --- | --- | --- | --- |
| A_70_P062431 | RBP1 | retinol binding protein 1, cellular | -2.02 | 0.003 | 1.07 | 0.722 | 2.37 | 0.001 | 1.80 | 0.010 |
| A_70_P062896 | MTHFD2 | methylenetetrahydrofolate dehydrogenase (NADP+ dependent) 2, met | 2.01 | 0.002 | 2.34 | 0.000 | 1.82 | 0.006 | 1.51 | 0.040 |
| A_70_P062971 | STX19 | syntaxin 19 | -2.58 | 0.000 | -1.81 | 0.006 | -1.80 | 0.006 | -1.89 | 0.004 |
| A_70_P063011 | GLRX2 | glutaredoxin 2 | 1.38 | 0.105 | 2.03 | 0.002 | 1.87 | 0.005 | -1.08 | 0.675 |
| A_70_P063116 | LAMA2 | laminin, alpha 2 | -2.04 | 0.017 | -1.63 | 0.083 | 1.52 | 0.127 | 1.99 | 0.020 |
| A_70_P063186 | CA2 | carbonic anhydrase II | -2.73 | 0.021 | -1.88 | 0.122 | -1.09 | 0.815 | 1.59 | 0.245 |
| A_70_P063191 | POLD2 | polymerase (DNA directed), delta 2, regulatory subunit 50kDa | 1.25 | 0.357 | 2.37 | 0.003 | 1.14 | 0.590 | -1.07 | 0.773 |
| A_70_P063396 | MXRA5 | matrix-remodelling associated 5 | 2.30 | 0.003 | 1.92 | 0.014 | 3.53 | 0.000 | 4.02 | 0.000 |
| A_70_P063431 | MAGI3 | membrane associated guanylate kinase, WW and PDZ domain containi | -2.36 | 0.000 | -1.68 | 0.013 | -1.59 | 0.023 | -1.37 | 0.100 |
| A_70_P063441 | CAP2 | CAP, adenylate cyclase-associated protein, 2 (yeast) | -2.33 | 0.002 | -1.79 | 0.016 | -1.89 | 0.010 | -1.82 | 0.014 |
| A_70_P063556 | CCRL2 | chemokine (C-C motif) receptor-like 2 | -3.11 | 0.002 | -1.95 | 0.035 | -1.69 | 0.087 | -1.29 | 0.382 |
| A_70_P063606 | HPGD | hydroxyprostaglandin dehydrogenase 15-(NAD) | -1.66 | 0.027 | -1.64 | 0.031 | -2.02 | 0.004 | -1.21 | 0.369 |
| A_70_P063646 | RAD54L | RAD54-like (S. cerevisiae) | -2.78 | 0.001 | 1.39 | 0.175 | -1.12 | 0.623 | -1.14 | 0.565 |
| A_70_P063686 | SCG2 | secretogranin II | -1.38 | 0.083 | -2.03 | 0.001 | -2.27 | 0.000 | -1.13 | 0.501 |
| A_70_P063836 | FANCD2 | Fanconi anemia, complementation group D2 | -1.17 | 0.093 | 3.42 | 0.000 | 2.95 | 0.000 | 1.75 | 0.000 |
| A_70_P063861 | CDH17 | cadherin 17, LI cadherin (liver-intestine) | -1.04 | 0.891 | 1.72 | 0.055 | 1.76 | 0.047 | 2.71 | 0.002 |
| A_70_P063921 | S100A14 | S100 calcium binding protein A14 | 1.91 | 0.001 | 2.18 | 0.000 | 2.33 | 0.000 | 1.19 | 0.281 |
| A_70_P063941 | METTL1 | methyltransferase like 1 | 2.79 | 0.000 | 2.72 | 0.000 | 1.36 | 0.126 | -1.03 | 0.896 |

| **Probenames** | **Gene Symbol** | **Gene Name** | **6h vs Baseline**  **Fold**  **Change P** | | **d1 vs Baseline**  **Fold**  **Change P** | | **d3 vs Baseline**  **Fold**  **Change P** | | **d7 vs Baseline**  **Fold**  **Change P** | |
| --- | --- | --- | --- | --- | --- | --- | --- | --- | --- | --- |
| A_70_P063996 | IRF8 | interferon regulatory factor 8 | -1.95 | 0.008 | -1.13 | 0.581 | 2.04 | 0.006 | 1.71 | 0.026 |
| A_70_P064011 | BCAS1 | breast carcinoma amplified sequence 1 | -2.48 | 0.005 | -1.67 | 0.075 | -2.39 | 0.006 | -1.96 | 0.024 |
| A_70_P064061 | DBP | D site of albumin promoter (albumin D-box) binding protein | -2.98 | 0.001 | -2.10 | 0.014 | -1.42 | 0.196 | -1.76 | 0.048 |
| A_70_P064081 | CA4 | carbonic anhydrase IV | -2.81 | 0.019 | -3.12 | 0.012 | -3.69 | 0.005 | -1.86 | 0.130 |
| A_70_P064091 | FLI1 | Friend leukemia virus integration 1 | -2.42 | 0.001 | -1.48 | 0.076 | -1.18 | 0.428 | 1.19 | 0.409 |
| A_70_P064136 | NXF1 | nuclear RNA export factor 1 | 2.12 | 0.094 | 2.10 | 0.098 | 2.92 | 0.023 | -1.15 | 0.737 |
| A_70_P064186 | CDC6 | cell division cycle 6 homolog (S. cerevisiae) | 1.00 | 0.981 | 6.01 | 0.000 | 3.47 | 0.000 | 2.08 | 0.003 |
| A_70_P064211 | CENPL | centromere protein L | -1.25 | 0.360 | 2.28 | 0.004 | 2.66 | 0.001 | 1.53 | 0.091 |
| A_70_P064246 | ERI2 | ERI1 exoribonuclease family member 2 | -1.50 | 0.007 | 1.99 | 0.000 | 2.89 | 0.000 | 1.39 | 0.022 |
| A_70_P064276 | SPC25 | SPC25, NDC80 kinetochore complex component, homolog | -1.10 | 0.515 | 5.40 | 0.000 | 5.11 | 0.000 | 1.83 | 0.001 |
| A_70_P064331 | CYBB | cytochrome b-245, beta polypeptide | -2.22 | 0.007 | -1.66 | 0.061 | -1.17 | 0.531 | 1.56 | 0.098 |
| A_70_P064366 | BCL11A | B-cell CLL/lymphoma 11A (zinc finger protein) | -2.41 | 0.000 | -1.59 | 0.006 | 1.14 | 0.355 | -1.20 | 0.216 |
| A_70_P064391 | RAB30 | RAB30, member RAS oncogene family | -2.74 | 0.000 | -1.80 | 0.003 | -1.46 | 0.036 | -1.23 | 0.225 |
| A_70_P064431 | SBNO2 | strawberry notch homolog 2 (Drosophila) | 3.35 | 0.002 | 3.18 | 0.002 | 2.46 | 0.011 | 1.80 | 0.071 |
| A_70_P064521 | PIM2 | pim-2 oncogene | -2.27 | 0.007 | -1.71 | 0.056 | -1.89 | 0.028 | -1.18 | 0.531 |
| A_70_P064561 | CBX5 | chromobox homolog 5 | 1.07 | 0.664 | 2.05 | 0.000 | 1.83 | 0.001 | 1.15 | 0.346 |
| A_70_P064576 | CENPH | centromere protein H | -1.57 | 0.003 | 3.31 | 0.000 | 2.62 | 0.000 | 1.42 | 0.015 |
| A_70_P064581 | ICAM3 | intercellular adhesion molecule 3 | -2.05 | 0.004 | -1.48 | 0.080 | -1.58 | 0.044 | -1.13 | 0.551 |
| A_70_P064756 | YPEL3 | yippee-like 3 (Drosophila) | -2.37 | 0.001 | -1.79 | 0.012 | -1.07 | 0.725 | -1.47 | 0.072 |
| A_70_P064811 | LRMP | lymphoid-restricted membrane protein | -2.45 | 0.002 | -1.83 | 0.017 | -2.08 | 0.006 | -1.34 | 0.207 |

| **Probenames** | **Gene Symbol** | **Gene Name** | **6h vs Baseline**  **Fold**  **Change P** | | **d1 vs Baseline**  **Fold**  **Change P** | | **d3 vs Baseline**  **Fold**  **Change P** | | **d7 vs Baseline**  **Fold**  **Change P** | |
| --- | --- | --- | --- | --- | --- | --- | --- | --- | --- | --- |
| A_70_P064826 | OLFM4 | olfactomedin 4 (OLFM4) | 14.22 | 0.000 | 22.43 | 0.000 | 4.83 | 0.002 | 1.39 | 0.436 |
| A_70_P064866 | P2RY10 | purinergic receptor P2Y, G-protein coupled, 10 | -2.36 | 0.001 | -1.71 | 0.024 | 1.04 | 0.852 | 1.14 | 0.533 |
| A_70_P064901 | C19orf33 | chromosome 19 open reading frame 33 | 1.14 | 0.495 | 1.29 | 0.186 | 2.40 | 0.000 | -1.12 | 0.550 |
| A_70_P064926 | CD19 | CD19 molecule | -3.01 | 0.006 | -1.32 | 0.415 | 1.69 | 0.134 | 1.06 | 0.855 |
| A_70_P065036 | CCDC28A | coiled-coil domain containing 28A | -2.20 | 0.001 | -1.74 | 0.009 | -1.54 | 0.033 | -1.90 | 0.004 |
| A_70_P065076 | C4BPA | component 4 binding protein, alpha (C4BPA) | 3.70 | 0.001 | 5.02 | 0.000 | 3.47 | 0.002 | 2.71 | 0.008 |
| A_70_P065106 | LOC785220 | similar to Putative TRAF4-associated factor 1 | -1.71 | 0.002 | 1.90 | 0.000 | 2.26 | 0.000 | 1.30 | 0.070 |
| A_70_P065141 | PRKD2 | protein kinase D2 | -2.08 | 0.006 | -1.29 | 0.267 | -1.22 | 0.380 | -1.06 | 0.787 |
| A_70_P065376 | FBXO32 | F-box protein 32 | -2.16 | 0.005 | -3.19 | 0.000 | -2.35 | 0.002 | -1.47 | 0.106 |
| A_70_P065386 | METTL7A | methyltransferase like 7A | -2.43 | 0.000 | -1.55 | 0.028 | -1.65 | 0.014 | -1.70 | 0.011 |
| A_70_P065446 | BCL2A1 | BCL2-related protein A1 | -2.46 | 0.012 | -1.32 | 0.379 | 1.27 | 0.446 | 1.18 | 0.590 |
| A_70_P065531 | FAR2 | fatty acyl CoA reductase 2 | 1.02 | 0.875 | 1.41 | 0.012 | 2.06 | 0.000 | 2.33 | 0.000 |
| A_70_P065566 | C1orf54 | chromosome 1 open reading frame 54 | -3.00 | 0.000 | -1.21 | 0.424 | 1.31 | 0.252 | -1.04 | 0.860 |
| A_70_P065596 | MAF | v-maf musculoaponeurotic fibrosarcoma oncogene homolog (avian) | -2.19 | 0.000 | -1.45 | 0.037 | 2.10 | 0.001 | 2.28 | 0.000 |
| A_70_P065626 | ATP5S | ATP synthase, H+ transporting, mitochondrial Fo complex, subunit | -2.05 | 0.001 | -1.39 | 0.059 | -1.36 | 0.079 | -1.52 | 0.021 |
| A_70_P065746 | PFDN1 | prefoldin subunit 1 | 1.39 | 0.136 | 1.90 | 0.009 | 2.09 | 0.004 | -1.35 | 0.173 |
| A_70_P065876 | QKI | quaking homolog, KH domain RNA binding (mouse) | -2.42 | 0.011 | -2.10 | 0.027 | -1.56 | 0.155 | 1.34 | 0.344 |
| A_70_P065896 | CMBL | carboxymethylenebutenolidase homolog (Pseudomonas) | -2.65 | 0.001 | -1.44 | 0.102 | -1.25 | 0.305 | -1.82 | 0.013 |

| **Probenames** | **Gene Symbol** | **Gene Name** | **6h vs Baseline**  **Fold**  **Change P** | | **d1 vs Baseline**  **Fold**  **Change P** | | **d3 vs Baseline**  **Fold**  **Change P** | | **d7 vs Baseline**  **Fold**  **Change P** | |
| --- | --- | --- | --- | --- | --- | --- | --- | --- | --- | --- |
| A_70_P065941 | TPST2 | tyrosylprotein sulfotransferase 2 | 1.37 | 0.269 | 2.05 | 0.022 | 1.71 | 0.075 | -1.04 | 0.891 |
| A_70_P065971 | BBS2 | Bardet-Biedl syndrome 2 | -2.50 | 0.001 | -1.69 | 0.022 | -1.73 | 0.017 | -1.58 | 0.041 |
| A_70_P066271 | UGT2A3 | UDP glucuronosyltransferase 2 family, polypeptide A3 | -2.23 | 0.053 | -2.15 | 0.063 | -2.34 | 0.042 | -1.79 | 0.145 |
| A_70_P066316 | PVRL3 | poliovirus receptor-related 3 | -2.73 | 0.000 | -1.72 | 0.019 | -2.32 | 0.001 | -1.62 | 0.032 |
| A_70_P066321 | C14orf135 | chromosome 14 open reading frame 135 | -2.57 | 0.003 | -1.90 | 0.030 | -2.42 | 0.005 | -1.46 | 0.168 |
| A_70_P066506 | TRA2A | transformer 2 alpha homolog (Drosophila) | -2.09 | 0.011 | -2.12 | 0.010 | -2.02 | 0.014 | -1.26 | 0.356 |
| A_70_P066716 | HLA-DMA | major histocompatibility complex, class II, DM alpha | -2.51 | 0.003 | -2.07 | 0.011 | -1.97 | 0.016 | -1.38 | 0.205 |
| A_70_P066736 | SMPX | small muscle protein, X-linked | -2.25 | 0.048 | -3.61 | 0.004 | -4.41 | 0.002 | -4.49 | 0.002 |
| A_70_P066846 | FAM109B | family with sequence similarity 109, member B | -2.11 | 0.000 | -1.29 | 0.077 | 1.39 | 0.030 | 1.41 | 0.025 |
| A_70_P066871 | BIRC5 | baculoviral IAP repeat-containing 5 (BIRC5) | -2.60 | 0.000 | -1.46 | 0.010 | -1.65 | 0.002 | -1.73 | 0.001 |
| A_70_P066981 | ACTN2 | actinin, alpha 2 | -1.63 | 0.083 | -2.28 | 0.008 | -3.27 | 0.001 | -2.35 | 0.006 |
| A_70_P067006 | CSPP1 | centrosome and spindle pole associated protein 1 | -2.51 | 0.002 | -1.77 | 0.035 | -2.26 | 0.005 | -1.52 | 0.106 |
| A_70_P067306 | PRSS23 | protease, serine, 23 | -4.04 | 0.001 | -3.29 | 0.003 | 1.01 | 0.970 | 3.90 | 0.001 |
| A_70_P067396 | PEAR1 | platelet endothelial aggregation receptor 1 | -2.42 | 0.001 | -1.31 | 0.209 | 1.43 | 0.102 | 1.41 | 0.114 |
| A_70_P067446 | TSPAN15 | tetraspanin 15 | -2.06 | 0.006 | -1.49 | 0.088 | -2.32 | 0.002 | -1.84 | 0.015 |
| A_70_P067461 | TNFRSF12A | tumor necrosis factor receptor superfamily, member 12A | 2.87 | 0.001 | 1.68 | 0.048 | 1.54 | 0.093 | -1.07 | 0.782 |
| A_70_P067476 | ZC3H11A | zinc finger CCCH-type containing 11A | 1.04 | 0.867 | -1.17 | 0.548 | -2.11 | 0.011 | -1.99 | 0.016 |

| **Probenames** | **Gene Symbol** | **Gene Name** | **6h vs Baseline**  **Fold**  **Change P** | | **d1 vs Baseline**  **Fold**  **Change P** | | **d3 vs Baseline**  **Fold**  **Change P** | | **d7 vs Baseline**  **Fold**  **Change P** | |
| --- | --- | --- | --- | --- | --- | --- | --- | --- | --- | --- |
| A_70_P067581 | SAA | serum amyloid A-like | 2.81 | 0.001 | 3.47 | 0.000 | 2.37 | 0.003 | 1.41 | 0.161 |
| A_70_P067861 | NUDT2 | nudix (nucleoside diphosphate linked moiety X)-type motif 2 | -2.27 | 0.000 | -1.29 | 0.057 | 1.33 | 0.036 | -1.36 | 0.025 |
| A_70_P067986 | SOD2 | manganous superoxide dismutase | 4.15 | 0.000 | 2.92 | 0.002 | 2.30 | 0.008 | 1.40 | 0.224 |
| A_70_P067987 | SOD2 | manganous superoxide dismutase | 4.14 | 0.000 | 2.94 | 0.001 | 2.39 | 0.006 | 1.49 | 0.155 |
| A_70_P068026 | TNFSF13B | tumor necrosis factor (ligand) superfamily, member 13b | -1.39 | 0.199 | -1.01 | 0.977 | 1.36 | 0.232 | 2.25 | 0.006 |
| A_70_P068027 | TNFSF13B | tumor necrosis factor (ligand) superfamily, member 13b | -1.30 | 0.302 | -1.03 | 0.909 | 1.36 | 0.233 | 2.19 | 0.007 |
| A_70_P068046 | CLDN3 | claudin 3 | -1.19 | 0.476 | -1.15 | 0.566 | -1.44 | 0.154 | -2.13 | 0.008 |
| A_70_P068052 | AP000926.2 | NULL | 1.22 | 0.395 | 1.10 | 0.668 | 1.74 | 0.030 | 2.32 | 0.003 |
| A_70_P068061 | CYBB | cytochrome b-245, beta polypeptide | -2.41 | 0.002 | -1.47 | 0.112 | -1.05 | 0.830 | 1.41 | 0.151 |
| A_70_P068091 | FZD2 | frizzled homolog 2 (Drosophila) | -2.27 | 0.000 | -1.33 | 0.118 | 1.09 | 0.602 | 1.10 | 0.580 |
| A_70_P068092 | FZD2 | frizzled homolog 2 (Drosophila) | -2.28 | 0.000 | -1.34 | 0.096 | 1.09 | 0.618 | 1.06 | 0.737 |
| A_70_P068101 | GATA3 | GATA binding protein 3 | -3.33 | 0.001 | -2.73 | 0.004 | -2.75 | 0.004 | -1.77 | 0.068 |
| A_70_P068102 | GATA3 | GATA binding protein 3 | -3.32 | 0.001 | -2.62 | 0.005 | -2.72 | 0.004 | -1.73 | 0.073 |
| A_70_P068141 | CDH2 | cadherin 2, type 1, N-cadherin (neuronal) | -1.31 | 0.163 | -1.12 | 0.539 | 1.01 | 0.942 | 2.11 | 0.002 |
| A_70_P068146 | NTN4 | netrin 4 | -2.36 | 0.000 | -1.50 | 0.047 | 1.14 | 0.493 | 1.45 | 0.062 |
| A_70_P068147 | NTN4 | netrin 4 | -2.34 | 0.001 | -1.53 | 0.040 | 1.15 | 0.466 | 1.43 | 0.079 |
| A_70_P068206 | WNT5A | wingless-type MMTV integration site family, member 5A | -1.15 | 0.482 | -1.05 | 0.823 | 1.97 | 0.004 | 2.18 | 0.002 |
| A_70_P068211 | XDH | xanthine dehydrogenase | 1.76 | 0.127 | 1.55 | 0.225 | 3.97 | 0.002 | 3.38 | 0.004 |
| A_70_P068212 | XDH | xanthine dehydrogenase | 1.83 | 0.122 | 1.77 | 0.143 | 4.47 | 0.001 | 4.03 | 0.002 |

| **Probenames** | **Gene Symbol** | **Gene Name** | **6h vs Baseline**  **Fold**  **Change P** | | **d1 vs Baseline**  **Fold**  **Change P** | | **d3 vs Baseline**  **Fold**  **Change P** | | **d7 vs Baseline**  **Fold**  **Change P** | |
| --- | --- | --- | --- | --- | --- | --- | --- | --- | --- | --- |
| A_70_P068216 | ITLN | intelectin | -1.84 | 0.164 | -1.64 | 0.250 | -3.11 | 0.017 | -2.52 | 0.044 |
| A_70_P068217 | ITLN | intelectin | -1.79 | 0.164 | -1.60 | 0.254 | -3.00 | 0.016 | -2.41 | 0.045 |
| A_70_P068301 | PPP2R5C | protein phosphatase 2, regulatory subunit B', gamma | -2.87 | 0.000 | -2.02 | 0.007 | -2.57 | 0.001 | -1.49 | 0.088 |
| A_70_P068316 | CNP | 2',3'-cyclic nucleotide 3' phosphodiesterase | 2.16 | 0.000 | 1.78 | 0.004 | 1.11 | 0.535 | 1.30 | 0.135 |
| A_70_P068346 | SGCE | sarcoglycan, epsilon | -2.30 | 0.000 | -1.99 | 0.001 | 1.33 | 0.093 | 1.30 | 0.124 |
| A_70_P068426 | RBMS2P1 | RNA binding motif, single stranded interacting protein 2 pseudog | -2.02 | 0.001 | -1.31 | 0.131 | 1.05 | 0.758 | 1.08 | 0.659 |
| A_70_P068476 | TPPP3 | tubulin polymerization-promoting protein family member 3 | -2.95 | 0.002 | -1.80 | 0.048 | -2.02 | 0.021 | -1.73 | 0.062 |
| A_70_P068531 | PDIA4 | protein disulfide isomerase family A, member 4 | 1.94 | 0.040 | 2.76 | 0.004 | 1.50 | 0.188 | 1.49 | 0.193 |
| A_70_P068541 | CKAP4 | cytoskeleton-associated protein 4 | 2.19 | 0.007 | 2.47 | 0.003 | 1.64 | 0.065 | 2.16 | 0.008 |
| A_70_P068551 | ELN | elastin | -1.87 | 0.025 | -1.21 | 0.445 | 2.75 | 0.001 | 2.86 | 0.001 |
| A_70_P068856 | APOE | apolipoprotein E | -2.30 | 0.001 | -1.72 | 0.019 | -1.33 | 0.177 | -1.17 | 0.443 |
| A_70_P068966 | KRT6A | keratin 6A (KRT6A) | 24.30 | 0.000 | 55.51 | 0.000 | 75.88 | 0.000 | 43.47 | 0.000 |
| A_70_P068981 | CST6 | cystatin E/M | 1.76 | 0.014 | 1.78 | 0.013 | 3.84 | 0.000 | 1.40 | 0.116 |
| A_70_P068996 | CCDC80 | coiled-coil domain containing 80 | 1.02 | 0.952 | -1.14 | 0.643 | 2.25 | 0.012 | 2.97 | 0.002 |
| A_70_P069001 | MYLPF | myosin light chain, phosphorylatable, fast skeletal muscle | 1.77 | 0.074 | 1.53 | 0.172 | 2.54 | 0.008 | -1.26 | 0.446 |
| A_70_P069006 | EPS8L1 | EPS8-like 1 | -2.06 | 0.003 | -1.58 | 0.035 | -1.55 | 0.041 | -1.66 | 0.021 |
| A_70_P069196 | CYP4X1 | cytochrome P450, family 4, subfamily X, polypeptide 1 | 1.73 | 0.009 | 3.62 | 0.000 | 1.22 | 0.282 | -1.06 | 0.739 |

| **Probenames** | **Gene Symbol** | **Gene Name** | **6h vs Baseline**  **Fold**  **Change P** | | **d1 vs Baseline**  **Fold**  **Change P** | | **d3 vs Baseline**  **Fold**  **Change P** | | **d7 vs Baseline**  **Fold**  **Change P** | |
| --- | --- | --- | --- | --- | --- | --- | --- | --- | --- | --- |
| A_70_P069376 | Kif26a | kinesin family member 26A (Kif26a) | -3.70 | 0.000 | -1.69 | 0.025 | -1.25 | 0.304 | -1.10 | 0.637 |
| A_70_P069501 | IRF4 | interferon regulatory factor 4 | -2.29 | 0.006 | -1.93 | 0.022 | -2.23 | 0.007 | -1.88 | 0.026 |
| A_70_P069571 | FIBIN | fin bud initiation factor homolog (zebrafish) | -2.32 | 0.000 | -2.92 | 0.000 | -1.37 | 0.058 | 1.24 | 0.188 |
| A_70_P069606 | CD3EAP | CD3e molecule, epsilon associated protein | -2.04 | 0.000 | -1.36 | 0.057 | -1.38 | 0.049 | -1.33 | 0.074 |
| A_70_P069626 | ARHGEF25 | Rho guanine nucleotide exchange factor (GEF) 25 | -2.25 | 0.000 | -1.74 | 0.004 | -1.25 | 0.178 | -1.06 | 0.709 |
| A_70_P069751 | PDGFRA | platelet-derived growth factor receptor, alpha polypeptide | -1.02 | 0.957 | 1.21 | 0.521 | 2.09 | 0.026 | 2.05 | 0.029 |
| A_70_P069771 | SMC2 | structural maintenance of chromosomes 2-like 1 | -1.23 | 0.173 | 3.02 | 0.000 | 2.30 | 0.000 | 1.63 | 0.005 |
| A_70_P069866 | FAM83D | family with sequence similarity 83, member D | -3.16 | 0.002 | -2.46 | 0.010 | -2.02 | 0.035 | -2.69 | 0.006 |
| A_70_P069881 | FBLIM1 | filamin binding LIM protein 1 | -1.09 | 0.659 | 1.44 | 0.077 | 1.93 | 0.004 | 2.06 | 0.002 |
| A_70_P069901 | FEN1 | flap structure-specific endonuclease 1 | -1.10 | 0.465 | 2.11 | 0.000 | 1.35 | 0.038 | 1.24 | 0.119 |
| A_70_P069951 | MYBPH | myosin binding protein H | 3.32 | 0.000 | 3.87 | 0.000 | 2.41 | 0.004 | 1.82 | 0.033 |
| A_70_P070026 | HDAC5 | histone deacetylase 5 | -2.09 | 0.002 | -1.61 | 0.028 | -1.38 | 0.112 | -1.32 | 0.166 |
| A_70_P070106 | BCO2 | beta-carotene oxygenase 2 | -2.34 | 0.003 | -1.19 | 0.454 | -1.26 | 0.325 | -1.05 | 0.843 |
| A_70_P070121 | DST | dystonin | -1.98 | 0.013 | -2.08 | 0.009 | -2.30 | 0.004 | -1.39 | 0.184 |
| A_70_P070131 | DNAJB5 | DnaJ (Hsp40) homolog, subfamily B, member 5 | -1.54 | 0.162 | -1.95 | 0.039 | -3.39 | 0.001 | -2.91 | 0.003 |
| A_70_P070226 | AMICA1 | adhesion molecule, interacts with CXADR antigen 1 | -1.27 | 0.148 | -1.04 | 0.790 | 1.95 | 0.001 | 2.25 | 0.000 |
| A_70_P070231 | LOC540011 | similar to spindle and KT associated 2 | -1.83 | 0.000 | 2.07 | 0.000 | 2.78 | 0.000 | 1.52 | 0.005 |

| **Probenames** | **Gene Symbol** | **Gene Name** | **6h vs Baseline**  **Fold**  **Change P** | | **d1 vs Baseline**  **Fold**  **Change P** | | **d3 vs Baseline**  **Fold**  **Change P** | | **d7 vs Baseline**  **Fold**  **Change P** | |
| --- | --- | --- | --- | --- | --- | --- | --- | --- | --- | --- |
| A_70_P070351 | CCNB2 | cyclin B2 | 1.05 | 0.816 | 3.78 | 0.000 | 4.56 | 0.000 | 2.21 | 0.003 |
| A_70_P070391 | MCM4 | minichromosome maintenance complex component 4 | -1.31 | 0.093 | 2.33 | 0.000 | 1.39 | 0.048 | 1.19 | 0.265 |
| A_70_P070554 | TYMS | thymidylate synthetase | -1.10 | 0.445 | 5.87 | 0.000 | 4.90 | 0.000 | 2.23 | 0.000 |
| A_70_P070561 | ACSM3 | acyl-CoA synthetase medium-chain family member 3 | -2.87 | 0.000 | -1.61 | 0.044 | -1.97 | 0.008 | -2.05 | 0.005 |
| A_70_P070611 | CREBL2 | cAMP responsive element binding protein-like 2 | -2.28 | 0.001 | -1.68 | 0.019 | -1.78 | 0.011 | -1.35 | 0.148 |
| A_70_P070661 | TM6SF1 | transmembrane 6 superfamily member 1 | -2.35 | 0.000 | -1.92 | 0.003 | -1.96 | 0.002 | -1.21 | 0.295 |
| A_70_P070801 | PDZK1IP1 | PDZK1 interacting protein 1 | 2.24 | 0.003 | 2.04 | 0.006 | 1.85 | 0.013 | -1.46 | 0.101 |
| A_70_P070831 | GGCT | gamma-glutamylcyclotransferase | -1.23 | 0.167 | 2.28 | 0.000 | 1.56 | 0.009 | 1.23 | 0.168 |
| A_70_P070981 | GADD45A | growth arrest and DNA-damage-inducible, alpha | 2.16 | 0.003 | 1.67 | 0.030 | 1.49 | 0.078 | 1.42 | 0.119 |
| A_70_P071086 | DNAJB9 | DnaJ (Hsp40) homolog, subfamily B, member 9 | 1.90 | 0.010 | 2.16 | 0.003 | 1.37 | 0.159 | 1.15 | 0.526 |
| A_70_P071096 | STX10 | syntaxin 10 | -2.14 | 0.000 | -1.39 | 0.047 | -1.27 | 0.131 | -1.61 | 0.008 |
| A_70_P071116 | TSR1 | TSR1, 20S rRNA accumulation, homolog (S. cerevisiae) | 2.03 | 0.004 | 2.02 | 0.004 | 1.14 | 0.529 | 1.03 | 0.902 |
| A_70_P071216 | CTSW | cathepsin W | -2.88 | 0.010 | -2.70 | 0.014 | -2.69 | 0.014 | 1.10 | 0.786 |
| A_70_P071286 | DCXR | dicarbonyl/L-xylulose reductase | -2.27 | 0.004 | -1.54 | 0.088 | -1.48 | 0.116 | -1.72 | 0.038 |
| A_70_P071416 | EEF2K | eukaryotic elongation factor-2 kinase | -2.06 | 0.000 | -1.81 | 0.001 | -1.96 | 0.001 | -1.64 | 0.005 |
| A_70_P071436 | ATF3 | activating transcription factor 3 | -1.29 | 0.528 | -2.02 | 0.094 | -2.54 | 0.032 | -2.51 | 0.035 |

| **Probenames** | **Gene Symbol** | **Gene Name** | **6h vs Baseline**  **Fold**  **Change P** | | **d1 vs Baseline**  **Fold**  **Change P** | | **d3 vs Baseline**  **Fold**  **Change P** | | **d7 vs Baseline**  **Fold**  **Change P** | |
| --- | --- | --- | --- | --- | --- | --- | --- | --- | --- | --- |
| A_70_P071471 | NPM3 | nucleophosmin/nucleoplasmin 3 | 5.99 | 0.000 | 5.34 | 0.000 | 2.12 | 0.004 | 1.02 | 0.944 |
| A_70_P071481 | SLC40A1 | solute carrier family 40 (iron-regulated transporter), member 1 | -3.02 | 0.003 | -2.50 | 0.010 | -2.84 | 0.005 | -1.14 | 0.680 |
| A_70_P071516 | ABHD4 | abhydrolase domain containing 4 | -2.13 | 0.000 | -1.62 | 0.011 | -1.05 | 0.761 | 1.12 | 0.489 |
| A_70_P071566 | RPE | ribulose-5-phosphate-3-epimerase | 1.07 | 0.629 | 1.92 | 0.001 | 2.07 | 0.000 | 1.21 | 0.190 |
| A_70_P072181 | PLCB2 | phospholipase C, beta 2 | -2.68 | 0.002 | -1.53 | 0.104 | -1.66 | 0.059 | -1.09 | 0.716 |
| A_70_P072266 | MXRA8 | matrix-remodelling associated 8 | -2.66 | 0.000 | -2.16 | 0.001 | 1.59 | 0.021 | 1.58 | 0.022 |
| Keratin14_TC3860.f1 | KRT14 | Keratin 14 | 1.94 | 0.048 | 3.24 | 0.002 | 4.32 | 0.000 | 2.33 | 0.016 |
| Keratin5_TC8502.f1 | KRT5 | Keratin 5 | 1.83 | 0.082 | 2.77 | 0.008 | 4.61 | 0.000 | 2.40 | 0.018 |
| TubulinB_TC2191.f1 | TUBB | Tubulin beta | 1.81 | 0.031 | 2.74 | 0.001 | 2.15 | 0.009 | 1.28 | 0.327 |
