# Supplementary material for: Gene Expression Changes Associated with the Airway Wall Response to Injury
Source: PLoS One. 2013 Apr 9;8(4):e58930. doi: 10.1371/journal.pone.0058930 (PMC3621906; doi:10.1371/journal.pone.0058930)
Supplement: Table S2 — a: The results of functional annotation clustering analysis, using the DAVID knowledge database (http://david.abcc.ncifcrf.gov; version 2008), applied to the significantly down-regulated annotated genes showing a greater than two-fold change in expression at 6 h (n = 561). Only clusters with an enrichment score of 4.0 or above, and a significance level <0.05 are tabulated. The complete GO gene-associations database for the human genome was used as a reference list. DAVID determined all the annotated biologic process GO terms that existed both in the background gene list and those associated with our genes of interest. The GO FAT option was chosen to select a subset of the GO term set relating only to biological process. The number of appearances of each GO term was counted and compared between the groups of interest and for the reference genes. A modified Fisher exact test was calculated for all analyses and the Benjamini-Hochberg method was used to control the false discovery rate for the enrichment p-values for the given individual term members. b: The results of functional annotation clustering analysis, using the DAVID knowledge database (http://david.abcc.ncifcrf.gov; version 2008), applied to the significantly up-regulated annotated genes showing a greater than two-fold change in expression at 6 h (n = 154). See legend for table 1a for description of table derivation. (DOC) [file pone.0058930.s003.doc]

| Direction of change | Cluster | Enrichment score | Term | Count | PValue | Genes | List Total | Pop Hits | Fold Enrichment | Benjamini | FDR |
| --- | --- | --- | --- | --- | --- | --- | --- | --- | --- | --- | --- |
| Down | 1 | 4.4 | GO:0001568~blood vessel development | 23 | 2.56E-06 | ACVRL1, AGT, ANGPT1, ANGPT2, APOE, CAV1, CDH5, CITED2, CXCL12, DLL4, EDNRA, EMCN, EPAS1, GJA5, IL18, KDR, LAMA4, LMO2, MMP19, PLXND1, QKI, SH2D2A, TBX3 | 392 | 245 | 3.2 | 0.006 | 0.004 |
|  |  |  | GO:0001944~vasculature development | 23 | 3.81E-06 | ACVRL1, AGT, ANGPT1, ANGPT2, APOE, CAV1, CDH5, CITED2, CXCL12, DLL4, EDNRA, EMCN, EPAS1, GJA5, IL18, KDR, LAMA4, LMO2, MMP19, PLXND1, QKI, SH2D2A, TBX3 | 392 | 251 | 3.2 | 0.005 | 0.007 |
|  |  |  | GO:0048514~blood vessel morphogenesis | 18 | 1.37E-04 | ACVRL1, AGT, ANGPT1, ANGPT2, APOE, CAV1, CITED2, CXCL12, DLL4, EDNRA, EMCN, EPAS1, IL18, KDR, MMP19, PLXND1, QKI, SH2D2A | 392 | 211 | 2.9 | 0.053 | 0.240 |
|  |  |  | GO:0001525~angiogenesis | 13 | 0.001 | ACVRL1, ANGPT1, ANGPT2, CXCL12, DLL4, EDNRA, EMCN, EPAS1, IL18, KDR, MMP19, PLXND1, SH2D2A | 392 | 148 | 3.0 | 0.087 | 2.122 |

Table S2a

| Direction of change | Cluster | Enrichment score | Term | Count | PValue | Genes | List Total | Pop Hits | Fold Enrichment | Benjamini | FDR |
| --- | --- | --- | --- | --- | --- | --- | --- | --- | --- | --- | --- |
| Up | 1 | 9.4 | GO:0009611~response to wounding | 26 | 2.72E-12 | AHSG, C4BPA, CCL2, CXCL2, FN1, FOS, GAL, GNA13, IL6, IL8, LBP, PLAT, PTX3, S100A12, S100A9, SELP, SERPINB2, SERPINE1, SOD2, SPP1, TFPI2, THBD, THBS1, TLR2, TNFAIP6, VCAN | 118 | 530 | 5.6 | 4.11E-09 | 4.53E-09 |
|  |  |  | GO:0006954~inflammatory response | 18 | 2.65E-09 | AHSG, C4BPA, CCL2, CXCL2, FN1, FOS, GAL, IL6, IL8, LBP, PTX3, S100A12, S100A9, SELP, SPP1, THBS1, TLR2, TNFAIP6 | 118 | 325 | 6.3 | 2.00E-06 | 4.41E-06 |
|  |  |  | GO:0006952~defense response | 23 | 1.19E-08 | AHSG, ANKRD1, C4BPA, CCL2, CXCL2, FN1, FOS, GAL, HP, IL6, IL8, INHBA, KCNN4, LBP, PLA2G2A, PTX3, S100A12, S100A9, SELP, SPP1, THBS1, TLR2, TNFAIP6 | 118 | 615 | 4.3 | 5.99E-06 | 1.98E-05 |
| Up | 2 | 4.5 | GO:0002237~response to molecule of bacterial origin | 9 | 7.44E-07 | CCL2, FOS, IL6, LBP, NOS3, SELP, SOCS3, THBD, TLR2 | 118 | 86 | 12.0 | 2.25E-04 | 0.001 |
|  |  |  | GO:0009617~response to bacterium | 11 | 6.58E-06 | CCL2, FOS, IL6, LBP, NOS3, PLA2G2A, S100A12, SELP, SOCS3, THBD, TLR2 | 118 | 193 | 6.5 | 0.001 | 0.011 |
|  |  |  | GO:0010033~response to organic substance | 19 | 4.49E-05 | AHSG, CCL2, CDKN1A, FOS, GAL, IL6, JUNB, KRT19, LBP, MANF, MYC, NOS3, OXT, SELP, SOCS3, SPP1, THBD, THBS1, TLR2 | 118 | 721 | 3.0 | 0.006 | 0.075 |
|  |  |  | GO:0032496~response to lipopolysaccharide | 7 | 5.29E-05 | CCL2, FOS, LBP, NOS3, SELP, SOCS3, THBD | 118 | 77 | 10.4 | 0.006 | 0.088 |
|  |  |  | GO:0042742~defense response to bacterium | 6 | 0.003 | IL6, LBP, PLA2G2A, S100A12, SELP, TLR2 | 118 | 112 | 6.1 | 0.074 | 4.6 |

Table S2b
