# Supplementary material for: Gene Expression Changes Associated with the Airway Wall Response to Injury
Source: PLoS One. 2013 Apr 9;8(4):e58930. doi: 10.1371/journal.pone.0058930 (PMC3621906; doi:10.1371/journal.pone.0058930)
Supplement: Table S3 — a: The results of functional annotation clustering analysis, using the DAVID knowledge database (http://david.abcc.ncifcrf.gov; version 2008), applied to the significantly up-regulated annotated genes showing a greater than two-fold change in expression at d1 (n = 324). See legend for table 1a for description of table derivation. b: The results of functional annotation clustering analysis, using the DAVID knowledge database (http://david.abcc.ncifcrf.gov; version 2008), applied to the significantly up-regulated annotated genes showing a greater than two-fold change in expression at d1 (n = 324). See legend for table 1a for description of table derivation. (DOC) [file pone.0058930.s004.doc]

| Direction of change | Cluster | Enrichment score | Term | Count | % | PValue | Genes | List Total | Pop Hits | Fold Enrichment | Benjamini | FDR |
| --- | --- | --- | --- | --- | --- | --- | --- | --- | --- | --- | --- | --- |
| Up | 1 | 21.1 | GO:0000279~ M phase | 47 | 15.8 | 1.28E-27 | ASPM, AURKA, AURKB, BIRC5, BUB1, CCNA2, CCNB1, CCNB2, CCNB3, CDC20, CDC6, CDCA3, CDCA5, CDCA8, CKS2, DLGAP5, ESPL1, FANCD2, FBXO5, KIF22, KIF2C, KNTC1, KPNA2, MND1, NCAPG, NCAPH, NDC80, NEK2, NEK6, NOLC1, NUF2, PRC1, PTTG1, RAD51, RANBP1, SKA1, SMC1A, SMC2, SPC25, TACC3, TPX2, TRIP13, TTK, TUBB, UBE2C, ZWILCH, ZWINT | 251 | 329 | 7.7 | 2.64E-24 | 2.20E-24 |
|  |  |  | GO:0022403~ cell cycle phase | 48 | 16.2 | 3.30E-24 | ASPM, AURKA, AURKB, BIRC5, BUB1, CCNA2, CCNB1, CCNB2, CCNB3, CDC20, CDC6, CDCA3, CDCA5, CDCA8, CKS2, DLGAP5, ESPL1, FANCD2, FBXO5, INHBA, KIF22, KIF2C, KNTC1, KPNA2, MND1, NCAPG, NCAPH, NDC80, NEK2, NEK6, NOLC1, NUF2, PRC1, PTTG1, RAD51, RANBP1, SKA1, SMC1A, SMC2, SPC25, TACC3, TPX2, TRIP13, TTK, TUBB, UBE2C, ZWILCH, ZWINT | 251 | 414 | 6.2 | 3.42E-21 | 5.70E-21 |
|  |  |  | GO:0022402~ cell cycle process | 54 | 18.2 | 2.47E-23 | ASPM, AURKA, AURKB, BIRC5, BUB1, CALR, CCNA2, CCNB1, CCNB2, CCNB3, CDC20, CDC6, CDCA3, CDCA5, CDCA8, CEP72, CGREF1, CKS2, DLGAP5, ESPL1, FANCD2, FBXO5, IL8, INHBA, KIF22, KIF2C, KNTC1, KPNA2, MND1, MYC, NCAPG, NCAPH, NDC80, NEK2, NEK6, NOLC1, NUF2, PRC1, PTTG1, RACGAP1P, RAD51, RANBP1, SKA1, SMC1A, SMC2, SPC25, TACC3, TPX2, TRIP13, TTK, TUBB, UBE2C, ZWILCH,ZWINT | 251 | 565 | 5.2 | 1.71E-20 | 4.27E-20 |
|  |  |  | GO:0000280~ nuclear division | 36 | 12.1 | 5.00E-23 | ASPM, AURKA, AURKB, BIRC5, BUB1, CCNA2, CCNB1, CCNB2, CDC20, CDC6, CDCA3, CDCA5, CDCA8, DLGAP5, ESPL1, FBXO5, KIF22, KIF2C, KNTC1, NCAPG, NCAPH, NDC80, NEK2, NEK6, NOLC1, NUF2, PTTG1, SKA1, SMC1A, SMC2, SPC25, TPX2, TUBB, UBE2C, ZWILCH, ZWINT | 251 | 220 | 8.8 | 2.59E-20 | 8.63E-20 |
|  |  |  | GO:0007067~ mitosis | 36 | 12.1 | 5.00E-23 | ASPM, AURKA, AURKB, BIRC5, BUB1, CCNA2, CCNB1, CCNB2, CDC20, CDC6, CDCA3, CDCA5, CDCA8, DLGAP5, ESPL1, FBXO5, KIF22, KIF2C, KNTC1, NCAPG, NCAPH, NDC80, NEK2, NEK6, NOLC1, NUF2, PTTG1, SKA1, SMC1A, SMC2, SPC25, TPX2, TUBB, UBE2C, ZWILCH, ZWINT | 251 | 220 | 8.8 | 2.59E-20 | 8.63E-20 |

Table S3a

| Direction of change | Cluster | Enrichment score | Term | Count | % | PValue | Genes | List Total | Pop Hits | Fold Enrichment | Benjamini | FDR |
| --- | --- | --- | --- | --- | --- | --- | --- | --- | --- | --- | --- | --- |
|  |  |  | GO:0000087~M phase of mitotic cell cycle | 36 | 12.1 | 9.29E-23 | ASPM, AURKA, AURKB, BIRC5, BUB1, CCNA2, CCNB1, CCNB2, CDC20, CDC6, CDCA3, CDCA5, CDCA8, DLGAP5, ESPL1, FBXO5, KIF22, KIF2C, KNTC1, NCAPG, NCAPH, NDC80, NEK2, NEK6, NOLC1, NUF2, PTTG1, SKA1, SMC1A, SMC2, SPC25, TPX2, TUBB, UBE2C, ZWILCH, ZWINT | 251 | 224 | 8.7 | 3.85E-20 | 1.60E-19 |
|  |  |  | GO:0048285~ organelle fission | 36 | 12.1 | 1.98E-22 | ASPM, AURKA, AURKB, BIRC5, BUB1, CCNA2, CCNB1, CCNB2, CDC20, CDC6, CDCA3, CDCA5, CDCA8, DLGAP5, ESPL1, FBXO5, KIF22, KIF2C, KNTC1, NCAPG, NCAPH, NDC80, NEK2, NEK6, NOLC1, NUF2, PTTG1, SKA1, SMC1A, SMC2, SPC25, TPX2, TUBB, UBE2C, ZWILCH, ZWINT | 251 | 229 | 8.5 | 6.82E-20 | 3.41E-19 |
|  |  |  | GO:0007049~ cell cycle | 61 | 20.5 | 4.17E-22 | ASPM, AURKA, AURKB, BIRC5, BUB1, CALR, CCNA2, CCNB1, CCNB2, CCNB3, CDC20, CDC45, CDC6, CDCA3, CDCA5, CDCA8, CDT1, CEP72, CGREF1, CKAP2, CKS1B, CKS2, DLGAP5, DTYMK, ESPL1, FANCD2, FBXO5, IL8, INHBA, KIF22, KIF2C, KNTC1, KPNA2, MCM3, MND1, MYC, NCAPG, NCAPH, NDC80, NEK2, NEK6, NOLC1, NUF2, PRC1, PTTG1, RACGAP1P, RAD51, RANBP1, SKA1, SMC1A, SMC2, SPC25, TACC3, TPX2, TRIP13, TTK, TUBB, UBE2C, UHRF1, ZWILCH, ZWINT | 251 | 776 | 4.2 | 1.23E-19 | 7.21E-19 |
|  |  |  | GO:0051301~ cell division | 37 | 12.5 | 1.08E-19 | ASPM, AURKB, BIRC5, BUB1, CCNA2, CCNB1, CCNB2, CCNB3, CDC20, CDC6, CDCA3, CDCA5, CDCA8, CENPH, CKS1B, CKS2, ESPL1, FBXO5, KNTC1, MCM5, NCAPG, NCAPH, NDC80, NEK2, NEK6, NOX5, NUF2, PRC1, PTTG1, RACGAP1P, SKA1, SMC1A, SMC2, SPC25, UBE2C, ZWILCH, ZWINT | 251 | 295 | 6.8 | 2.78E-17 | 1.86E-16 |
|  |  |  | GO:0000278~mitotic cell cycle | 40 | 13.5 | 5.25E-19 | ASPM, AURKA, AURKB, BIRC5, BUB1, CCNA2, CCNB1, CCNB2, CDC20, CDC6, CDCA3, CDCA5, CDCA8, DLGAP5, ESPL1, FBXO5, INHBA, KIF22, KIF2C, KNTC1, KPNA2, NCAPG, NCAPH, NDC80, NEK2, NEK6, NOLC1, NUF2, PRC1, PTTG1, SKA1, SMC1A, SMC2, SPC25, TPX2, TTK, TUBB, UBE2C, ZWILCH, ZWINT | 251 | 370 | 5.8 | 1.21E-16 | 9.07E-16 |
|  |  |  | GO:0007059~ chromosome segregation | 17 | 5.7 | 1.32E-12 | BIRC5, CDCA5, CENPH, DLGAP5, ESPL1, NCAPG, NCAPH, NDC80, NEK2, NEK6, NUF2, PTTG1, SKA1, SMC1A, SMC2, SPC25, ZWINT | 251 | 81 | 11.3 | 2.48E-10 | 2.28E-09 |

Table S3b

| Direction of change | Cluster | Enrichment score | Term | Count | % | PValue | Genes | List Total | Pop Hits | Fold  Enrichment | Benjamini | FDR |
| --- | --- | --- | --- | --- | --- | --- | --- | --- | --- | --- | --- | --- |
| Up | 2 | 8.3 | GO:0007051~ spindle organization | 14 | 4.7 | 9.27E-13 | AURKA, CKS2, ESPL1, FBXO5, NDC80, PRC1, RANBP1, SMC1A, SPC25, TACC3, TTK, TUBB, UBE2C, ZWINT | 251 | 45 | 16.8 | 1.92E-10 | 1.60E-09 |
|  |  |  | GO:0000226~ microtubule cytoskeleton organization | 18 | 6.1 | 1.76E-09 | AURKA, BBS1, CEP72, CKS2, ESPL1, FBXO5, KIF2C, NDC80, NEK2, PRC1, RANBP1, SMC1A, SPC25, TACC3, TTK, TUBB, UBE2C, ZWINT | 251 | 147 | 6.6 | 2.60E-07 | 3.03E-06 |
|  |  |  | GO:0007017~ microtubule-based process | 21 | 7.1 | 4.81E-08 | AURKA, BBS1, CEP72, CKS2, ESPL1, FBXO5, KIF20A, KIF22, KIF2C, KPNA2, NDC80, NEK2, PRC1, RANBP1, SMC1A, SPC25, TACC3, TTK, TUBB, UBE2C, ZWINT | 251 | 253 | 4.5 | 5.86E-06 | 8.31E-05 |
|  |  |  | GO:0007010~ cytoskeleton organization | 24 | 8.1 | 6.22E-06 | AURKA, BBS1, CALR, CEP72, CKS2, DIAPH3, ESPL1, FBXO5, KIF2C, KRT14, KRT19, NDC80, NEK2, PRC1, RACGAP1P, RANBP1, S100A9, SMC1A, SPC25, TACC3, TTK, TUBB, UBE2C, ZWINT | 251 | 436 | 3.0 | 5.85E-04 | 0.011 |
| Up | 3 | 5.0 | GO:0007059~ chromosome segregation | 17 | 5.7 | 1.32E-12 | BIRC5, CDCA5, CENPH, DLGAP5, ESPL1, NCAPG, NCAPH, NDC80, NEK2, NEK6, NUF2, PTTG1, SKA1, SMC1A, SMC2, SPC25, ZWINT | 251 | 81 | 11.3 | 2.48E-10 | 2.28E-09 |
|  |  |  | GO:0000070~ mitotic sister chromatid segregation | 10 | 3.4 | 1.33E-08 | CDCA5, DLGAP5, ESPL1, NCAPG, NCAPH, NDC80, NEK2, SMC1A, SMC2, ZWINT | 251 | 36 | 15.0 | 1.83E-06 | 2.29E-05 |
|  |  |  | GO:0000819~sister chromatid segregation | 10 | 3.4 | 1.72E-08 | CDCA5, DLGAP5, ESPL1, NCAPG, NCAPH, NDC80, NEK2, SMC1A, SMC2, ZWINT | 251 | 37 | 14.6 | 2.23E-06 | 2.98E-05 |
|  |  |  | GO:0007076~ mitotic chromosome condensation | 4 | 1.3 | 0.002 | CDCA5, NCAPG, NCAPH, SMC2 | 251 | 13 | 16.6 | 0.072 | 2.650 |
|  |  |  | GO:0030261~ chromosome condensation | 4 | 1.3 | 0.011 | CDCA5, NCAPG, NCAPH, SMC2 | 251 | 25 | 8.6 | 0.252 | 16.8 |
|  |  |  | GO:0051276~ chromosome organization | 17 | 5.7 | 0.018 | ASF1B, CBX5, CDCA5, CDCA8, CENPH, DLGAP5, ESPL1, EZH2, FANCD2, NCAPG, NCAPH, NDC80, NEK2, PTTG1, SMC1A, SMC2, ZWINT | 251 | 485 | 1.9 | 0.328 | 27.3 |
|  |  |  | GO:0006323~DNA packaging | 5 | 1.7 | 0.171 | ASF1B, CDCA5, NCAPG, NCAPH, SMC2 | 251 | 117 | 2.3 | 0.811 | 96.1 |

Table S3b contd.
