# Supplementary material for: Gene Expression Changes Associated with the Airway Wall Response to Injury
Source: PLoS One. 2013 Apr 9;8(4):e58930. doi: 10.1371/journal.pone.0058930 (PMC3621906; doi:10.1371/journal.pone.0058930)
Supplement: Table S4 — a: The results of functional annotation clustering analysis, using the DAVID knowledge database (http://david.abcc.ncifcrf.gov; version 2008), applied to the significantly up-regulated annotated genes showing a greater than two-fold change in expression at d3 (n = 366). See legend for table 1a for description of table derivation. b: The results of functional annotation clustering analysis, using the DAVID knowledge database (http://david.abcc.ncifcrf.gov; version 2008), applied to the significantly up-regulated annotated genes showing a greater than two-fold change in expression at d3 (n = 366). See legend for table 1a for description of table derivation. c: The results of functional annotation clustering analysis, using the DAVID knowledge database (http://david.abcc.ncifcrf.gov; version 2008), applied to the significantly up-regulated annotated genes showing a greater than two-fold change in expression at d3 (n = 366). See legend for table 1a for description of table derivation. d: The results of functional annotation clustering analysis, using the DAVID knowledge database (http://david.abcc.ncifcrf.gov; version 2008), applied to the significantly up-regulated annotated genes showing a greater than two-fold change in expression at d3 (n = 366). See legend for table 1a for description of table derivation. e: The results of functional annotation clustering analysis, using the DAVID knowledge database (http://david.abcc.ncifcrf.gov; version 2008), applied to the significantly up-regulated annotated genes showing a greater than two-fold change in expression at d3 (n = 366). See legend for table 1a for description of table derivation. (DOC) [file pone.0058930.s005.doc]

| Direction of change | Cluster | Enrichment score | Term | Count | % | PValue | Genes | List Total | Pop Hits | Fold Enrichment | Benjamini | FDR |
| --- | --- | --- | --- | --- | --- | --- | --- | --- | --- | --- | --- | --- |
| Up | 1 | 15.1 | GO:0000279~ M phase | 42 | 12.4 | 1.28E-20 | ASPM, AURKA, AURKB, BIRC5, BUB1, CCNA2, CCNB1, CCNB2, CCNB3, CDC20, CDC6, CDCA3, CDCA5, CDCA8, CKS2, CRYAA, DLGAP5, ESPL1, FANCD2, FBXO5, KIF22, KIF2C, KNTC1, MND1, NCAPG, NDC80, NEK2, NEK6, NUF2, PRC1, PTTG1, RAD51, SKA1, SMC2, SPC25, STMN1, TACC3, TPX2, TRIP13, TTK, TUBB, UBE2C | 284 | 329 | 6.1 | 2.93E-17 | 2.24E-17 |
|  |  |  | GO:0022403~ cell cycle phase | 44 | 13.0 | 1.54E-18 | ASPM, AURKA, AURKB, BIRC5, BUB1, CCNA2, CCNB1, CCNB2, CCNB3, CDC20, CDC6, CDCA3, CDCA5, CDCA8, CDKN2D, CKS2, CRYAA, DLGAP5, ESPL1, FANCD2, FBXO5, INHBA, KIF22, KIF2C, KNTC1, MND1, NCAPG, NDC80, NEK2, NEK6, NUF2, PRC1, PTTG1, RAD51, SKA1, SMC2, SPC25, STMN1, TACC3, TPX2, TRIP13, TTK, TUBB, UBE2C | 284 | 414 | 5.1 | 1.75E-15 | 2.68E-15 |
|  |  |  | GO:0022402~ cell cycle process | 49 | 14.5 | 4.69E-17 | ASPM, AURKA, AURKB, BIRC5, BUB1, CCNA2, CCNB1, CCNB2, CCNB3, CDC20, CDC6, CDCA3, CDCA5, CDCA8, CDKN2D, CEP72, CGREF1, CKS2, CRYAA, DLGAP5, ESPL1, FANCD2, FBXO5, IL8, INHBA, KIF22, KIF2C, KNTC1, MND1, NCAPG, NDC80, NEK2, NEK6, NUF2, PRC1, PTTG1, RACGAP1P, RAD51, SKA1, SMC2, SPC25, STMN1, TACC3, TGFB1, TPX2, TRIP13, TTK, TUBB, UBE2C | 284 | 565 | 4.1 | 3.57E-14 | 8.18E-14 |
|  |  |  | GO:0007067~ mitosis | 31 | 9.1 | 2.07E-16 | ASPM, AURKA, AURKB, BIRC5, BUB1, CCNA2, CCNB1, CCNB2, CDC20, CDC6, CDCA3, CDCA5, CDCA8, DLGAP5, ESPL1, FBXO5, KIF22, KIF2C, KNTC1, NCAPG, NDC80, NEK2, NEK6, NUF2, PTTG1, SKA1, SMC2, SPC25, TPX2, TUBB, UBE2C | 284 | 220 | 6.7 | 1.27E-13 | 3.89E-13 |
|  |  |  | GO:0000280~ nuclear division | 31 | 9.1 | 2.07E-16 | ASPM, AURKA, AURKB, BIRC5, BUB1, CCNA2, CCNB1, CCNB2, CDC20, CDC6, CDCA3, CDCA5, CDCA8, DLGAP5, ESPL1, FBXO5, KIF22, KIF2C, KNTC1, NCAPG, NDC80, NEK2, NEK6, NUF2, PTTG1, SKA1, SMC2, SPC25, TPX2, TUBB, UBE2C | 284 | 220 | 6.7 | 1.27E-13 | 3.89E-13 |

Table S4a

| Direction  of change | Cluster | Enrichment score | Term | Count | % | PValue | Genes | List Total | Pop Hits | Fold Enrichment | Benjamini | FDR |
| --- | --- | --- | --- | --- | --- | --- | --- | --- | --- | --- | --- | --- |
|  |  |  | GO:0000087~M phase of mitotic cell cycle | 31 | 9.1 | 3.42E-16 | ASPM, AURKA, AURKB, BIRC5, BUB1, CCNA2, CCNB1, CCNB2, CDC20, CDC6, CDCA3, CDCA5, CDCA8, DLGAP5, ESPL1, FBXO5, KIF22, KIF2C, KNTC1, NCAPG, NDC80, NEK2, NEK6, NUF2, PTTG1, SKA1, SMC2, SPC25, TPX2, TUBB, UBE2C | 284 | 224 | 6.6 | 1.52E-13 | 5.77E-13 |
|  |  |  | GO:0007049~ cell cycle | 56 | 16.5 | 6.49E-16 | ASPM, AURKA, AURKB, BIRC5, BUB1, CCNA2, CCNB1, CCNB2, CCNB3, CDC20, CDC45, CDC6, CDCA3, CDCA5, CDCA8, CDKN2D, CEP72, CGREF1, CKAP2, CKS1B, CKS2, CRYAA, DLGAP5, DTYMK, ESPL1, FANCD2, FBXO5, IL8, INHBA, KIF22, KIF2C, KNTC1, MND1, NCAPG, NDC80, NEK2, NEK6, NUF2, PFDN1, PRC1, PTTG1, RACGAP1P, RAD51, SKA1, SMC2, SPC25, STMN1, TACC3, TGFB1, TLK2, TPX2, TRIP13, TTK, TUBB, UBE2C, UHRF1 | 284 | 776 | 3.4 | 2.54E-13 | 1.17E-12 |
|  |  |  | GO:0048285~ organelle fission | 31 | 9.1 | 7.41E-16 | ASPM, AURKA, AURKB, BIRC5, BUB1, CCNA2, CCNB1, CCNB2, CDC20, CDC6, CDCA3, CDCA5, CDCA8, DLGAP5, ESPL1, FBXO5, KIF22, KIF2C, KNTC1, NCAPG, NDC80, NEK2, NEK6, NUF2, PTTG1, SKA1, SMC2, SPC25, TPX2, TUBB, UBE2C | 284 | 229 | 6.4 | 2.54E-13 | 1.35E-12 |
|  |  |  | GO:0051301~ cell division | 33 | 9.7 | 1.77E-14 | CKS1B, RBP4, PRC1, NEK2, KNTC1, AURKB, PTTG1, SPC25, CDCA8, NCAPG, BUB1, FBXO5, SKA1, CCNA2, CDCA5, ASPM, CDCA3, CDC6, NOX5, NUF2, CDC20, ESPL1, BIRC5, NDC80, UBE2C, SMC2, CENPH, CCNB1, CCNB3, CCNB2, RACGAP1P, CKS2, NEK6 | 284 | 295 | 5.3 | 4.48E-12 | 3.08E-11 |
|  |  |  | GO:0000278~ mitotic cell cycle | 36 | 10.6 | 6.15E-14 | ASPM, AURKA, AURKB, BIRC5, BUB1, CCNA2, CCNB1, CCNB2, CDC20, CDC6, CDCA3, CDCA5, CDCA8, CDKN2D, DLGAP5, ESPL1, FBXO5, INHBA, KIF22, KIF2C, KNTC1, NCAPG, NDC80, NEK2, NEK6, NUF2, PRC1, PTTG1, SKA1, SMC2, SPC25, STMN1, TPX2, TTK, TUBB, UBE2C | 284 | 370 | 4.6 | 1.41E-11 | 1.07E-10 |
|  |  |  | GO:0007059~ chromosome segregation | 14 | 4.1 | 1.18E-08 | BIRC5, CDCA5, CENPH, DLGAP5, ESPL1, NCAPG, NDC80, NEK2, NEK6, NUF2, PTTG1, SKA1, SMC2, SPC25 | 284 | 81 | 8.2 | 2.07E-06 | 2.05E-05 |

Table S4a contd

| Direction of change | Cluster | Enrichment score | Term | Count | % | PValue | Genes | List Total | Pop Total | Fold Enrichment | Benjamini | FDR |
| --- | --- | --- | --- | --- | --- | --- | --- | --- | --- | --- | --- | --- |
| Up | 2 | 7.7 | GO:0009611~  response to wounding | 44 | 13.0 | 1.52E-14 | AHSG, C1S, C4BPA, C7, C9, CCL2, CCL21, CCNB1, CD14, CDH3, COL3A1, COL5A1, CXCL13, CXCL2, F13A1, FN1, GAL, GNA13, IGF1, IGFBP4, IL1RN, IL6, IL8, ITGA5, LBP, MIF, NOX4, PDGFRA, PLAT, PLAU, PLAUR, PTX3, S100A12, S100A9, SELP, SERPINB2, SERPINE1, SOD2, SPP1, STAB1, TGFB1, TNFAIP6, VCAN, VWF | 284 | 530 | 4.0 | 4.35E-12 | 2.66E-11 |
|  |  |  | GO:0006954~  inflammatory response | 27 | 8.0 | 4.66E-09 | AHSG, C1S, C4BPA, C7, C9, CCL2, CCL21, CD14, CXCL13, CXCL2, FN1, GAL, IGFBP4, IL1RN, IL6, IL8, LBP, MIF, NOX4, PTX3, S100A12, S100A9, SELP, SPP1, STAB1, TGFB1, TNFAIP6 | 284 | 325 | 4.0 | 8.88E-07 | 8.14E-06 |
|  |  |  | GO:0006952~  defense response | 32 | 9.4 | 5.24E-06 | AHSG, C1S, C4BPA, C7, C9, CCL2, CCL21, CD14, CXCL13, CXCL2, FCGR1A, FN1, GAL, HP, IGFBP4, IL1RN, IL6, IL8, INHBA, LBP, MIF, NOX4, PLA2G2A, PTX3, S100A12, S100A9, SELP, SPP1, STAB1, TGFB1, TNFAIP6, TUBB | 284 | 615 | 2.5 | 4.13E-04 | 0.009 |
|  |  |  | GO:0006955~ immune response | 30 | 8.8 | 2.75E-04 | ADA, C1S, C4BPA, C7, C9, CCL2, CCL21, CD14, CD86, CLEC4E, CLEC6A, CXCL13, CXCL2, CXCL5, ENPP1, FCGR1A, IL1R2, IL1RN, IL6, IL8, IRF8, LBP, MIF, MYLPF, POU2AF1, PTX3, SBNO2, TGFB1, TREM2, TUBB | 284 | 690 | 2.1 | 0.012 | 0.479 |
| Up | 3 | 5.9 | GO:0007398~  ectoderm development | 18 | 5.3 | 9.61E-07 | ADAMTS2, COL1A1, COL1A2, COL3A1, COL5A1, COL5A2, CRABP2, CST6, FABP5, GJB3, IVL, KRT14, KRT17, KRT5, KRT6A, MREG, SFN, SPRR1B | 284 | 199 | 4.3 | 1.10E-04 | 0.002 |
|  |  |  | GO:0008544~  epidermis development | 17 | 5.0 | 1.59E-06 | ADAMTS2, COL1A1, COL1A2, COL3A1, COL5A1, COL5A2, CRABP2, CST6, FABP5, GJB3, IVL, KRT14, KRT17, KRT5, MREG, SFN, SPRR1B | 284 | 184 | 4.4 | 1.58E-04 | 0.003 |
|  |  |  | GO:0043588~skin development | 8 | 2.4 | 1.71E-06 | ADAMTS2, COL1A1, COL1A2, COL3A1, COL5A1, COL5A2, GJB3, SFN | 284 | 29 | 13.1 | 1.63E-04 | 0.003 |

Table S4b

| Direction of change | Cluster | Enrichment score | Term | Count | % | PValue | Genes | List Total | Pop Hits | Fold Enrichment | Benjamini | FDR |
| --- | --- | --- | --- | --- | --- | --- | --- | --- | --- | --- | --- | --- |
| Up | 4 | 5.7 | GO:0007051~spindle organization | 12 | 3.5 | 1.49E-09 | AURKA, CKS2, ESPL1, FBXO5, NDC80, PRC1, SPC25, STMN1, TACC3, TTK, TUBB, UBE2C | 284 | 45 | 12.7 | 3.10E-07 | 2.61E-06 |
|  |  |  | GO:0000226~  microtubule cytoskeleton organization | 15 | 4.4 | 2.47E-06 | AURKA, CEP72, CKS2, ESPL1, FBXO5, KIF2C, NDC80, NEK2, PRC1, SPC25, STMN1, TACC3, TTK, TUBB, UBE2C | 284 | 147 | 4.9 | 2.25E-04 | 0.004 |
|  |  |  | GO:0007017~  microtubule-based process | 18 | 5.3 | 2.41E-05 | AURKA, CEP72, CKS2, CRYAA, ESPL1, FBXO5, KIF20A, KIF22, KIF2C, NDC80, NEK2, PRC1, SPC25, STMN1, TACC3, TTK, TUBB, UBE2C | 284 | 253 | 3.4 | 0.002 | 0.042 |
|  |  |  | GO:0007010~  cytoskeleton organization | 23 | 6.8 | 1.28E-04 | AURKA, CEP72, CKS2, CRYAA, DIAPH3, ELN, ESPL1, FBXO5, KIF2C, KRT14, KRT19, LASP1, NDC80, NEK2, PRC1, RACGAP1P, S100A9, SPC25, STMN1, TACC3, TTK, TUBB, UBE2C | 284 | 436 | 2.5 | 0.006 | 0.223 |
| Up | 5 | 5.6 | GO:0032963~ collagen metabolic process | 9 | 2.7 | 7.18E-08 | ADAMTS2, COL1A1, COL3A1, COL5A1, MMP1, MMP13, MMP2, MMP7, MMP9 | 284 | 28 | 15.3 | 1.17E-05 | 1.25E-04 |
|  |  |  | GO:0044259~ multicellular organismal macromolecule metabolic process | 9 | 2.7 | 1.73E-07 | ADAMTS2, COL1A1, COL3A1, COL5A1, MMP1, MMP13, MMP2, MMP7, MMP9 | 284 | 31 | 13.8 | 2.47E-05 | 3.01E-04 |
|  |  |  | GO:0044236~ multicellular organismal metabolic process | 9 | 2.7 | 7.58E-07 | ADAMTS2, COL1A1, COL3A1, COL5A1, MMP1, MMP13, MMP2, MMP7, MMP9 | 284 | 37 | 11.6 | 9.11E-05 | 0.001 |
|  |  |  | GO:0030574~ collagen catabolic process | 6 | 1.8 | 4.63E-05 | ADAMTS2, MMP1, MMP13, MMP2, MMP7, MMP9 | 284 | 20 | 14.3 | 0.003 | 0.081 |
|  |  |  | GO:0044243~ multicellular organismal catabolic process | 6 | 1.8 | 1.77E-04 | ADAMTS2, MMP1, MMP13, MMP2, MMP7, MMP9 | 284 | 26 | 11.0 | 0.009 | 0.309 |

Table S4c

| Direction of change | Cluster | Enrichment score | Term | Count | % | PValue | Genes | List Total | Pop Hits | Fold Enrichment | Benjamini | FDR |
| --- | --- | --- | --- | --- | --- | --- | --- | --- | --- | --- | --- | --- |
| Up | 6 | 4.8 | GO:0030198~ extracellular matrix organization | 14 | 4.1 | 2.49E-07 | ADAMTS2, CCDC80, COL18A1, COL1A1, COL1A2, COL3A1, COL5A1, COL5A2, ELN, LUM, MMP9, PDGFRA, POSTN, TGFBI | 284 | 104 | 6.4 | 3.35E-05 | 4.35E-04 |
|  |  |  | GO:0043588~ skin development | 8 | 2.4 | 1.71E-06 | ADAMTS2, COL1A1, COL1A2, COL3A1, COL5A1, COL5A2, GJB3, SFN | 284 | 29 | 13.1 | 1.63E-04 | 0.003 |
|  |  |  | GO:0030199~ collagen fibril organization | 7 | 2.1 | 2.52E-05 | ADAMTS2, COL1A1, COL1A2, COL3A1, COL5A1, COL5A2, LUM | 284 | 29 | 11.5 | 0.002 | 0.044 |
|  |  |  | GO:0043062~ extracellular structure organization | 14 | 4.1 | 3.83E-05 | ADAMTS2, CCDC80, COL18A1, COL1A1, COL1A2, COL3A1, COL5A1, COL5A2, ELN, LUM, MMP9, PDGFRA, POSTN, TGFBI | 284 | 163 | 4.1 | 0.002 | 0.067 |
|  |  |  | GO:0032964~ collagen biosynthetic process | 3 | 0.9 | 0.004 | COL1A1, COL3A1, COL5A1 | 284 | 5 | 28.6 | 0.098 | 7.1 |

Table S4d

| Direction of change | Cluster | Enrichment score | Term | Count | % | PValue | Genes | List Total | Pop Hits | Fold Enrichment | Benjamini | FDR |
| --- | --- | --- | --- | --- | --- | --- | --- | --- | --- | --- | --- | --- |
| Up | 7 | 4.1 | GO:0048545~response to steroid hormone stimulus | 17 | 5.0 | 2.79E-06 | AURKA, CCL2, CCNA2, COL1A1, GAL, IGFBP2, IL1RN, IL6, JUNB, KRT19, MMP13, NOS3, OXT, PDGFRA, SOCS3, SPP1, TGFB1 | 284 | 192 | 4.2 | 2.45E-04 | 0.005 |
|  |  |  | GO:0009725~response to hormone stimulus | 22 | 6.5 | 3.05E-05 | AHSG, AURKA, CCL2, CCNA2, COL1A1, EIF4EBP1, ENPP1, GAL, IGFBP2, IL1RN, IL6, JUNB, KRT19, MMP13, NOS3, OXT, PDGFRA, RBP4, SOCS3, SPP1, STAR, TGFB1 | 284 | 367 | 2.9 | 0.002 | 0.053 |
|  |  |  | GO:0043627~response to estrogen stimulus | 11 | 3.2 | 6.86E-05 | AURKA, CCNA2, GAL, IGFBP2, KRT19, MMP13, NOS3, OXT, PDGFRA, SOCS3, TGFB1 | 284 | 105 | 5.0 | 0.004 | 0.120 |
|  |  |  | GO:0032355~response to estradiol stimulus | 8 | 2.4 | 1.24E-04 | AURKA, CCNA2, IGFBP2, NOS3, OXT, PDGFRA, SOCS3, TGFB1 | 284 | 54 | 7.1 | 0.006 | 0.217 |
|  |  |  | GO:0009719~response to endogenous stimulus | 22 | 6.5 | 1.25E-04 | AHSG, AURKA, CCL2, CCNA2, COL1A1, EIF4EBP1, ENPP1, GAL, IGFBP2, IL1RN, IL6, JUNB, KRT19, MMP13, NOS3, OXT, PDGFRA, RBP4, SOCS3, SPP1, STAR, TGFB1 | 284 | 405 | 2.6 | 0.006 | 0.218 |
|  |  |  | GO:0043434~response to peptide hormone stimulus | 12 | 3.5 | 3.94E-04 | AHSG, CCNA2, COL1A1, EIF4EBP1, ENPP1, GAL, JUNB, NOS3, OXT, RBP4, SOCS3, STAR | 284 | 154 | 3.7 | 0.016 | 0.686 |
|  |  |  | GO:0010033~response to organic substance | 29 | 8.6 | 0.001 | AHSG, AURKA, C1S, CCL2, CCNA2, CD14, COL1A1, COL3A1, EIF4EBP1, ENPP1, GAL, HSPA6, IGFBP2, IL1RN, IL6, JUNB, KRT19, LBP, MANF, MMP13, NOS3, OXT, PDGFRA, RBP4, SELP, SOCS3, SPP1, STAR, TGFB1 | 284 | 721 | 1.9 | 0.036 | 2.1 |

Table S4e
