# Supplementary material for: Gene Expression Changes Associated with the Airway Wall Response to Injury
Source: PLoS One. 2013 Apr 9;8(4):e58930. doi: 10.1371/journal.pone.0058930 (PMC3621906; doi:10.1371/journal.pone.0058930)
Supplement: Table S5 — a: The results of functional annotation clustering analysis, using the DAVID knowledge database (http://david.abcc.ncifcrf.gov; version 2008), applied to the significantly down-regulated annotated genes showing a greater than two-fold change in expression at d7 (n = 68). See legend for table 1a for description of table derivation. b: The results of functional annotation clustering analysis, using the DAVID knowledge database (http://david.abcc.ncifcrf.gov; version 2008), applied to the significantly up-regulated annotated genes showing a greater than two-fold change in expression at d7 (n = 197). See legend for table 1a for description of table derivation. c: The results of functional annotation clustering analysis, using the DAVID knowledge database (http://david.abcc.ncifcrf.gov; version 2008), applied to the significantly up-regulated annotated genes showing a greater than two-fold change in expression at d7 (n = 197). See legend for table 1a for description of table derivation. d: The results of functional annotation clustering analysis, using the DAVID knowledge database (http://david.abcc.ncifcrf.gov; version 2008), applied to the significantly up-regulated annotated genes showing a greater than two-fold change in expression at d7 (n = 197). See legend for table 1a for description of table derivation. e: The results of functional annotation clustering analysis, using the DAVID knowledge database (http://david.abcc.ncifcrf.gov; version 2008), applied to the significantly up-regulated annotated genes showing a greater than two-fold change in expression at d7 (n = 197). See legend for table 1a for description of table derivation. f: The results of functional annotation clustering analysis, using the DAVID knowledge database (http://david.abcc.ncifcrf.gov; version 2008), applied to the significantly up-regulated annotated genes showing a greater than two-fold change in expression at d7 (n = 197). See legend for table 1a for description of table derivation. (DOC) [file pone.0058930.s006.doc]

| Direction of change | Cluster | Enrichment score | Term | Count | % | PValue | Genes | List Total | Pop Hits | Fold Enrichment | Benjamini | FDR |
| --- | --- | --- | --- | --- | --- | --- | --- | --- | --- | --- | --- | --- |
| Down | 1 | 4.3 | GO:0006936~muscle contraction | 8 | 12.7 | 2.06E-06 | ACTC1, ACTN2, CRYAB, DES, DTNA, SLMAP, SMPX, SMTN | 54 | 153 | 13.1 | 1.68E-03 | 3.17E-03 |
|  |  |  | GO:0003012~muscle system process | 8 | 12.7 | 3.83E-06 | ACTC1, ACTN2, CRYAB, DES, DTNA, SLMAP, SMPX, SMTN | 54 | 168 | 11.9 | 1.57E-03 | 5.90E-03 |
|  |  |  | GO:0006941~ striated muscle contraction | 3 | 4.8 | 1.40E-02 | ACTC1, DTNA, SMPX | 54 | 46 | 16.3 | 9.00E-01 | 1.95E+01 |

Table S5a

| Direction of change | Cluster | Enrichment score | Term | Count | % | PValue | Genes | List Total | Pop Hits | Fold Enrichment | Benjamini | FDR |
| --- | --- | --- | --- | --- | --- | --- | --- | --- | --- | --- | --- | --- |
| Up | 1 | 11.0 | GO:0030198~ extracellular matrix organization | 16 | 0.7 | 2.11E-12 | ADAMTS2, CCDC80, COL12A1, COL1A1, COL1A2, COL3A1, COL5A1, COL5A2, ELN, LOX, LUM, MMP9, PDGFRA, POSTN, SERPINH1, TGFBI | 167 | 104 | 12.5 | 1.81E-09 | 3.56E-09 |
|  |  |  | GO:0043062~ extracellular structure organization | 18 | 0.8 | 1.45E-11 | ADAMTS2, CCDC80, CDH2, COL12A1, COL1A1, COL1A2, COL3A1, COL5A1, COL5A2, ELN, LOX, LUM, MMP9, PDGFRA, POSTN, SERPINH1, TGFBI, TNC | 167 | 163 | 8.9 | 8.30E-09 | 2.45E-08 |
|  |  |  | GO:0030199~ collagen fibril organization | 10 | 0.4 | 4.12E-11 | ADAMTS2, COL12A1, COL1A1, COL1A2, COL3A1, COL5A1, COL5A2, LOX, LUM, SERPINH1 | 167 | 29 | 27.9 | 1.41E-08 | 6.95E-08 |
| Up | 2 | 7.5 | GO:0032963~ collagen metabolic process | 10 | 0.4 | 2.87E-11 | ADAMTS2, COL1A1, COL3A1, COL5A1, MMP1, MMP13, MMP2, MMP7, MMP9, SERPINH1 | 167 | 28 | 28.9 | 1.23E-08 | 4.84E-08 |
|  |  |  | GO:0044259~ multicellular organismal macromolecule metabolic process | 10 | 0.4 | 8.12E-11 | ADAMTS2, COL1A1, COL3A1, COL5A1, MMP1, MMP13, MMP2, MMP7, MMP9, SERPINH1 | 167 | 31 | 26.1 | 2.32E-08 | 1.37E-07 |
|  |  |  | GO:0044236~ multicellular organismal metabolic process | 10 | 0.4 | 4.70E-10 | ADAMTS2, COL1A1, COL3A1, COL5A1, MMP1, MMP13, MMP2, MMP7, MMP9, SERPINH1 | 167 | 37 | 21.9 | 8.06E-08 | 7.94E-07 |
|  |  |  | GO:0030574~ collagen catabolic process | 6 | 0.3 | 3.50E-06 | ADAMTS2, MMP1, MMP13, MMP2, MMP7, MMP9 | 167 | 20 | 24.3 | 3.00E-04 | 5.91E-03 |
|  |  |  | GO:0044243~ multicellular organismal catabolic process | 6 | 0.3 | 1.40E-05 | ADAMTS2, MMP1, MMP13, MMP2, MMP7, MMP9 | 167 | 26 | 18.7 | 8.55E-04 | 2.36E-02 |
|  |  |  | GO:0032964~ collagen biosynthetic process | 4 | 0.2 | 1.78E-05 | COL1A1, COL3A1, COL5A1, SERPINH1 | 167 | 5 | 64.8 | 1.02E-03 | 3.01E-02 |

Table S5b

| Direction of change | Cluster | Enrichment score | Term | Count | % | PValue | Genes | List Total | Pop Hits | Fold Enrichment | Benjamini | FDR |
| --- | --- | --- | --- | --- | --- | --- | --- | --- | --- | --- | --- | --- |
| Up | 3 | 6.9 | GO:0030199~ collagen fibril organization | 10 | 0.4 | 4.12E-11 | ADAMTS2, COL12A1, COL1A1, COL1A2, COL3A1, COL5A1, COL5A2, LOX, LUM, SERPINH1 | 167 | 29 | 27.9 | 1.41E-08 | 6.95E-08 |
|  |  |  | GO:0043588~ skin development | 8 | 0.4 | 4.59E-08 | ADAMTS2, COL1A1, COL1A2, COL3A1, COL5A1, COL5A2, GJB3, SFN | 167 | 29 | 22.3 | 7.15E-06 | 7.75E-05 |
|  |  |  | GO:0007398~ ectoderm development | 13 | 0.6 | 6.19E-06 | ADAMTS2, COL1A1, COL1A2, COL3A1, COL5A1, COL5A2, CRABP2, GJB3, KRT14, KRT5, KRT6A, MREG, SFN | 167 | 199 | 5.3 | 5.05E-04 | 1.05E-02 |
|  |  |  | GO:0008544~ epidermis development | 12 | 0.5 | 1.65E-05 | ADAMTS2, COL1A1, COL1A2, COL3A1, COL5A1, COL5A2, CRABP2, GJB3, KRT14, KRT5, MREG, SFN | 167 | 184 | 5.3 | 9.74E-04 | 2.78E-02 |

Table S5c

| Direction of change | Cluster | Enrichment score | Term | Count | % | PValue | Genes | List Total | Pop Hits | Fold Enrichment | Benjamini | FDR |
| --- | --- | --- | --- | --- | --- | --- | --- | --- | --- | --- | --- | --- |
| Up | 4 | 4.6 | GO:0001501~  skeletal system development | 19 | 0.8 | 7.37E-08 | ANKH, BMP1, COL12A1, COL1A1, COL1A2, COL3A1, COL5A2, FBN1, IGF1, IGFBP3, MMP13, MMP2, MMP9, PDGFRA, POSTN, SPP1, TGFB1, TGFB3, WNT5A | 167 | 319 | 4.8 | 1.05E-05 | 1.24E-04 |
|  |  |  | GO:0001503~ossification | 10 | 0.4 | 1.17E-05 | BMP1, COL1A1, COL5A2, IGF1, IGFBP3, MMP13, MMP2, SPP1, TGFB1, TGFB3 | 167 | 115 | 7.0 | 8.04E-04 | 1.98E-02 |
|  |  |  | GO:0060348~bone development | 10 | 0.4 | 2.02E-05 | BMP1, COL1A1, COL5A2, IGF1, IGFBP3, MMP13, MMP2, SPP1, TGFB1, TGFB3 | 167 | 123 | 6.6 | 1.12E-03 | 3.41E-02 |
|  |  |  | GO:0001649~osteoblast differentiation | 4 | 0.2 | 1.47E-02 | COL1A1, IGF1, IGFBP3, SPP1 | 167 | 42 | 7.7 | 1.77E-01 | 2.21E+01 |
| Up | 5 | 4.6 | GO:0016477~cell migration | 15 | 0.7 | 7.69E-06 | CCL2, CDH2, COL5A1, CTHRC1, FN1, IL6, IL8, ITGA5, NOX1, PLAU, SELP, TGFB1, THBS4, VCAM1, VCAN | 167 | 276 | 4.4 | 5.98E-04 | 1.30E-02 |
|  |  |  | GO:0051674~localization of cell | 15 | 0.7 | 2.54E-05 | CCL2, CDH2, COL5A1, CTHRC1, FN1, IL6, IL8, ITGA5, NOX1, PLAU, SELP, TGFB1, THBS4, VCAM1, VCAN | 167 | 307 | 4.0 | 1.36E-03 | 4.29E-02 |
|  |  |  | GO:0048870~cell motility | 15 | 0.7 | 2.54E-05 | CCL2, CDH2, COL5A1, CTHRC1, FN1, IL6, IL8, ITGA5, NOX1, PLAU, SELP, TGFB1, THBS4, VCAM1, VCAN | 167 | 307 | 4.0 | 1.36E-03 | 4.29E-02 |
|  |  |  | GO:0006928~cell motion | 18 | 0.8 | 7.53E-05 | CCL2, CDH2, CERCAM, COL5A1, CTHRC1, FN1, IGF1, IL6, IL8, ITGA5, NOX1, PLAU, PLAUR, SELP, TGFB1, THBS4, VCAM1, VCAN | 167 | 475 | 3.1 | 3.07E-03 | 1.27E-01 |
| Up | 6 | 4.4 | GO:0031099~regeneration | 10 | 0.4 | 1.53E-07 | CCL2, CCNB1, ENO3, IGF1, PLAU, PLAUR, SERPINE1, SOCS3, TGFB1, VCAN | 167 | 69 | 11.7 | 1.87E-05 | 2.59E-04 |
|  |  |  | GO:0042246~tissue regeneration | 6 | 0.3 | 4.03E-05 | CCNB1, ENO3, IGF1, PLAU, PLAUR, SERPINE1 | 167 | 32 | 15.2 | 2.03E-03 | 6.81E-02 |
|  |  |  | GO:0040007~growth | 11 | 0.5 | 8.63E-05 | CCNB1, CCNB2, EMP3, ENO3, IGF1, MREG, PLAU, PLAUR, SERPINE1, TGFB1, TGFB3 | 167 | 183 | 4.9 | 3.43E-03 | 1.46E-01 |
|  |  |  | GO:0043403~skeletal muscle regeneration | 4 | 0.2 | 2.04E-04 | ENO3, IGF1, PLAU, PLAUR | 167 | 10 | 32.4 | 7.27E-03 | 3.44E-01 |
|  |  |  | GO:0048589~developmental growth | 7 | 0.3 | 6.94E-04 | CCNB1, ENO3, IGF1, MREG, PLAU, PLAUR, SERPINE1 | 167 | 87 | 6.5 | 2.03E-02 | 1.16E+00 |

Table S5d

| irection of change | Cluster | Enrichment score | Term | Count | % | PValue | Genes | List Total | Pop Hits | Fold Enrichment | Benjamini | FDR |
| --- | --- | --- | --- | --- | --- | --- | --- | --- | --- | --- | --- | --- |
| Up | 7 | 4.2 | GO:0000280~nuclear division | 15 | 0.7 | 5.33E-07 | ASPM, AURKA, AURKB, BIRC5, CCNB1, CCNB2, CDC6, CDCA3, FBXO5, KIF2C, KNTC1, NCAPG, NDC80, TPX2, UBE2C | 167 | 220 | 5.5 | 6.09E-05 | 9.00E-04 |
|  |  |  | GO:0007067~mitosis | 15 | 0.7 | 5.33E-07 | ASPM, AURKA, AURKB, BIRC5, CCNB1, CCNB2, CDC6, CDCA3, FBXO5, KIF2C, KNTC1, NCAPG, NDC80, TPX2, UBE2C | 167 | 220 | 5.5 | 6.09E-05 | 9.00E-04 |
|  |  |  | GO:0000087~M phase of mitotic cell cycle | 15 | 0.7 | 6.63E-07 | ASPM, AURKA, AURKB, BIRC5, CCNB1, CCNB2, CDC6, CDCA3, FBXO5, KIF2C, KNTC1, NCAPG, NDC80, TPX2, UBE2C | 167 | 224 | 5.4 | 7.10E-05 | 1.12E-03 |
|  |  |  | GO:0048285~ organelle fission | 15 | 0.7 | 8.64E-07 | ASPM, AURKA, AURKB, BIRC5, CCNB1, CCNB2, CDC6, CDCA3, FBXO5, KIF2C, KNTC1, NCAPG, NDC80, TPX2, UBE2C | 167 | 229 | 5.3 | 8.22E-05 | 1.46E-03 |
|  |  |  | GO:0000279~M phase | 17 | 0.7 | 2.89E-06 | ASPM, AURKA, AURKB, BIRC5, CCNB1, CCNB2, CDC6, CDCA3, FBXO5, KIF2C, KNTC1, NCAPG, NDC80, PRC1, TPX2, TTK, UBE2C | 167 | 329 | 4.2 | 2.61E-04 | 4.88E-03 |
|  |  |  | GO:0000278~mitotic cell cycle | 17 | 0.7 | 1.28E-05 | ASPM, AURKA, AURKB, BIRC5, CCNB1, CCNB2, CDC6, CDCA3, FBXO5, KIF2C, KNTC1, NCAPG, NDC80, PRC1, TPX2, TTK, UBE2C | 167 | 370 | 3.7 | 8.42E-04 | 2.16E-02 |
|  |  |  | GO:0022403~cell cycle phase | 17 | 0.7 | 5.02E-05 | ASPM, AURKA, AURKB, BIRC5, CCNB1, CCNB2, CDC6, CDCA3, FBXO5, KIF2C, KNTC1, NCAPG, NDC80, PRC1, TPX2, TTK, UBE2C | 167 | 414 | 3.3 | 2.39E-03 | 8.47E-02 |
|  |  |  | GO:0022402~cell cycle process | 20 | 0.9 | 6.46E-05 | ASPM, AURKA, AURKB, BIRC5, CCNB1, CCNB2, CDC6, CDCA3, FBXO5, IL8, KIF2C, KNTC1, NCAPG, NDC80, PRC1, RACGAP1P, TGFB1, TPX2, TTK, UBE2C | 167 | 565 | 2.9 | 2.83E-03 | 1.09E-01 |
|  |  |  | GO:0051301~cell division | 14 | 0.6 | 7.08E-05 | ASPM, AURKB, BIRC5, CCNB1, CCNB2, CDC6, CDCA3, FBXO5, KNTC1, NCAPG, NDC80, PRC1, RACGAP1P, UBE2C | 167 | 295 | 3.8 | 2.95E-03 | 1.19E-01 |
|  |  |  | GO:0007051~spindle organization | 6 | 0.3 | 2.15E-04 | AURKA, FBXO5, NDC80, PRC1, TTK, UBE2C | 167 | 45 | 10.8 | 7.50E-03 | 3.63E-01 |

Table S5e

| fs | Cluster | Enrichment score | Term | Count | % | PValue | Genes | List Total | Pop Hits | Fold Enrichment | Benjamini | FDR |
| --- | --- | --- | --- | --- | --- | --- | --- | --- | --- | --- | --- | --- |
|  |  |  | GO:0007049~cell cycle | 21 | 0.9 | 1.32E-03 | ASPM, AURKA, AURKB, BIRC5, CCNB1, CCNB2, CDC6, CDCA3, CKAP2, FBXO5, IL8, KIF2C, KNTC1, NCAPG, NDC80, PRC1, RACGAP1P, TGFB1, TPX2, TTK, UBE2C | 167 | 776 | 2.2 | 3.38E-02 | 2.21E+00 |
|  |  |  | GO:0007010~cytoskeleton organization | 13 | 0.6 | 7.53E-03 | AURKA, DIAPH3, ELN, FBXO5, KIF2C, KRT14, KRT19, NDC80, PRC1, RACGAP1P, THY1, TTK, UBE2C | 167 | 436 | 2.4 | 1.15E-01 | 1.20E+01 |
|  |  |  | GO:0000226~microtubule cytoskeleton organization | 7 | 0.3 | 9.53E-03 | AURKA, FBXO5, KIF2C, NDC80, PRC1, TTK, UBE2C | 167 | 147 | 3.9 | 1.34E-01 | 1.49E+01 |
|  |  |  | GO:0007017~microtubule-based process | 9 | 0.4 | 1.29E-02 | AURKA, FBXO5, KIF20A, KIF2C, NDC80, PRC1, TTK, UBE2C, UCHL1 | 167 | 253 | 2.9 | 1.65E-01 | 1.96E+01 |
|  |  |  | GO:0007052~mitotic spindle organization | 3 | 0.1 | 1.42E-02 | NDC80, PRC1, TTK | 167 | 15 | 16.2 | 1.72E-01 | 2.14E+01 |
| Up | 8 | 4.0 | GO:0042060~wound healing | 18 | 0.8 | 1.82E-10 | C9, CCNB1, COL3A1, COL5A1, ENO3, F13A1, FN1, IGF1, IL6, ITGA5, LOX, PDGFRA, PLAU, PLAUR, SERPINB2, SERPINE1, TGFB1, TGFB3 | 167 | 191 | 7.6 | 3.47E-08 | 3.07E-07 |
|  |  |  | GO:0050817~coagulation | 7 | 0.3 | 1.59E-03 | C9, COL3A1, F13A1, IL6, PLAU, PLAUR, SERPINE1 | 167 | 102 | 5.6 | 3.82E-02 | 2.65E+00 |
|  |  |  | GO:0007596~blood coagulation | 7 | 0.3 | 1.59E-03 | C9, COL3A1, F13A1, IL6, PLAU, PLAUR, SERPINE1 | 167 | 102 | 5.6 | 3.82E-02 | 2.65E+00 |
|  |  |  | GO:0007599~hemostasis | 7 | 0.3 | 2.13E-03 | C9, COL3A1, F13A1, IL6, PLAU, PLAUR, SERPINE1 | 167 | 108 | 5.3 | 4.75E-02 | 3.53E+00 |
|  |  |  | GO:0050878~regulation of body fluid levels | 7 | 0.3 | 7.84E-03 | C9, COL3A1, F13A1, IL6, PLAU, PLAUR, SERPINE1 | 167 | 141 | 4.0 | 1.16E-01 | 1.24E+01 |

Table S5f
